# Global, regional, and national mortality due to unintentional carbon monoxide poisoning, 2000–2021: results from the Global Burden of Disease Study 2021

**DOI:** 10.1016/S2468-2667(23)00185-8

**Published:** 2023-10-06

**Authors:** GBD 2021 Carbon Monoxide Poisoning Collaborators, Madeline E Moberg, Erin B Hamilton, Scott M Zeng, Dana Bryazka, Jeff T Zhao, Rachel Feldman, Yohannes Habtegiorgis Abate, Mohsen Abbasi-Kangevari, Ame Mehadi Abdurehman, Aidin Abedi, Eman Abu-Gharbieh, Isaac Yeboah Addo, Abiola Victor Adepoju, Qorinah Estiningtyas Sakilah Adnani, Saira Afzal, Bright Opoku Ahinkorah, Sajjad Ahmad, Danial Ahmed, Haroon Ahmed, Dejene Tsegaye Alem, Adel Ali Saeed Al-Gheethi, Yousef Alimohamadi, Edward Kwabena Ameyaw, Mohammad Amrollahi-Sharifabadi, Tadele Fentabil Anagaw, Anayochukwu Edward Anyasodor, Jalal Arabloo, Aleksandr Y Aravkin, Seyyed Shamsadin Athari, Alok Atreya, Amirhossein Azari Jafari, Ashish D Badiye, Nayereh Baghcheghi, Sara Bagherieh, Hansi Bansal, Amadou Barrow, Azadeh Bashiri, Nebiyou Simegnew Bayileyegn, Alemshet Yirga Berhie, Akshaya Srikanth Bhagavathula, Pankaj Bhardwaj, Archith Boloor, Luis Alberto Cámera, Felix Carvalho, Márcia Carvalho, Eeshwar K Chandrasekar, Jung-Chen Chang, Vijay Kumar Chattu, Dinh-Toi Chu, Kaleb Coberly, Natália Cruz-Martins, Omid Dadras, Xiaochen Dai, Reza Darvishi Cheshmeh Soltani, Saswati Das, Subasish Das, Sisay Abebe Debela, Berecha Hundessa Demessa, Xinlei Deng, Abebaw Alemayehu Desta, Belay Desye, Meghnath Dhimal, Mahmoud Dibas, Haneil Larson Dsouza, Michael Ekholuenetale, Iman El Sayed, Waseem El-Huneidi, Daniel Berhanie Enyew, Adeniyi Francis Fagbamigbe, Ali Fatehizadeh, Syeda Anum Fatima Fatima, Florian Fischer, Richard Charles Franklin, Tushar Garg, Tilaye Gebru Gebi, Urge Gerema, Melaku Getachew, Motuma Erena Getachew, Farhad Ghamari, Mahaveer Golechha, Pouya Goleij, Sapna Gupta, Veer Bala Gupta, Vivek Kumar Gupta, Mehdi Harorani, Hamidreza Hasani, Abbas M Hassan, Hossein Hassanian-Moghaddam, Mohammed Bheser Hassen, Simon I Hay, Khezar Hayat, Mohammad Heidari, Mahsa Heidari-Foroozan, Demisu Zenbaba Heyi, Ramesh Holla, Praveen Hoogar, Md Shakhaoat Hossain, Mohammad-Salar Hosseini, Sorin Hostiuc, Soodabeh Hoveidamanesh, Olayinka Stephen Ilesanmi, Irena M Ilic, Mustapha Immurana, Chidozie C D Iwu, Umesh Jayarajah, Nitin Joseph, Charity Ehimwenma Joshua, Vidya Kadashetti, Tanuj Kanchan, Himal Kandel, Rami S Kantar, Neeti Kapoor, Ibraheem M Karaye, Patrick DMC Katoto, Himanshu Khajuria, Ejaz Ahmad Khan, Sorour Khateri, Farzad Khodamoradi, Moein Khormali, Jagdish Khubchandani, Grace Kim, Adnan Kisa, Hamid Reza Koohestani, Kewal Krishan, Naveen Kumar, Lucie Laflamme, Iván Landires, Bagher Larijani, Paolo Lauriola, Thao Thi Thu Le, Caterina Ledda, Seung Won Lee, Stephen S Lim, Stany W Lobo, Raimundas Lunevicius, Sandeep B Maharaj, Ritesh G Menezes, Alexios-Fotios A Mentis, Tomislav Mestrovic, Ted R Miller, Seyyedmohammadsadeq Mirmoeeni, Awoke Misganaw, Manish Mishra, Sanjeev Misra, Chaitanya Mittal, Esmaeil Mohammadi, Ali H Mokdad, Mohammad Ali Moni, Ebrahim Mostafavi, Sumaira Mubarik, Francesk Mulita, Jember Azanaw Mulualem, Temesgen Mulugeta, Christopher J L Murray, Isabella Myers, Biswa Prakash Nayak, Vinod C Nayak, Seyed Aria Nejadghaderi, Huong Lan Thi Nguyen, Van Thanh Nguyen, Hasti Nouraei, Ogochukwu Janet Nzoputam, Hassan Okati-Aliabad, Isaac Iyinoluwa Olufadewa, Michal Ordak, Alicia Padron-Monedero, Jagadish Rao Padubidri, Ashok Pandey, Suman Pant, Utsav Parekh, Shrikant Pawar, Amy E Peden, Ionela-Roxana Petcu, Frédéric B Piel, Zahra Zahid Piracha, Ghazaleh Pourali, Ibrahim Qattea, Maryam Faiz Qureshi, Pankaja Raghav Raghav, Mosiur Rahman, Shayan Rahmani, Premkumar Ramasubramani, Sheena Ramazanu, Salman Rawaf, Nazila Rezaei, Negar Rezaei, Mohsen Rezaeian, Basema Saddik, Malihe Sadeghi, Farideh Sadeghian, Umar Saeed, Amirhossein Sahebkar, Zahra Saif, Joseph W Sakshaug, Saina Salahi, Payman Salamati, Abdallah M Samy, Rodrigo Sarmiento-Suárez, David C Schwebel, Subramanian Senthilkumaran, Allen Seylani, Masood Ali Shaikh, Sunder Sham, Bereket Beyene Shashamo, Rahim Ali Sheikhi, B Suresh Kumar Shetty, Pavanchand H Shetty, Migbar Mekonnen Sibhat, Harpreet Singh, Paramdeep Singh, Eskinder Ayalew Sisay, Yonatan Solomon, Majid Taheri, Irfan Ullah, Sana Ullah, Francesco S Violante, Linh Gia Vu, Nuwan Darshana Wickramasinghe, Arzu Yigit, Naohiro Yonemoto, Zabihollah Yousefi, Muhammad Zaman, Mikhail Sergeevich Zastrozhin, Zhi-Jiang Zhang, Peng Zheng, Mohammad Zoladl, Jaimie D Steinmetz, Theo Vos, Mohsen Naghavi, Kanyin Liane Ong

## Abstract

**Background:**

Unintentional carbon monoxide poisoning is a largely preventable cause of death that has received insufficient attention. We aimed to conduct a comprehensive global analysis of the demographic, temporal, and geographical patterns of fatal unintentional carbon monoxide poisoning from 2000 to 2021.

**Methods:**

As part of the latest Global Burden of Diseases, Injuries, and Risk Factors Study (GBD), unintentional carbon monoxide poisoning mortality was quantified using the GBD cause of death ensemble modelling strategy. Vital registration data and covariates with an epidemiological link to unintentional carbon monoxide poisoning informed the estimates of death counts and mortality rates for all locations, sexes, ages, and years included in the GBD. Years of life lost (YLLs) were estimated by multiplying deaths by remaining standard life expectancy at age of death. Population attributable fractions (PAFs) for unintentional carbon monoxide poisoning deaths due to occupational injuries and high alcohol use were estimated.

**Findings:**

In 2021, the global mortality rate due to unintentional carbon monoxide poisoning was 0·366 per 100 000 (95% uncertainty interval 0·276–0·415), with 28 900 deaths (21 700–32 800) and 1·18 million YLLs (0·886–1·35) across all ages. Nearly 70% of deaths occurred in males (20 100 [15 800–24 000]), and the 50–54-year age group had the largest number of deaths (2210 [1660–2590]). The highest mortality rate was in those aged 85 years or older with 1·96 deaths (1·38–2·32) per 100 000. Eastern Europe had the highest age-standardised mortality rate at 2·12 deaths (1·98–2·30) per 100 000. Globally, there was a 53·5% (46·2–63·7) decrease in the age-standardised mortality rate from 2000 to 2021, although this decline was not uniform across regions. The overall PAFs for occupational injuries and high alcohol use were 13·6% (11·9–16·0) and 3·5% (1·4–6·2), respectively.

**Interpretation:**

Improvements in unintentional carbon monoxide poisoning mortality rates have been inconsistent across regions and over time since 2000. Given that unintentional carbon monoxide poisoning is almost entirely preventable, policy-level interventions that lower the risk of carbon monoxide poisoning events should be prioritised, such as those that increase access to improved heating and cooking devices, reduce carbon monoxide emissions from generators, and mandate use of carbon monoxide alarms.

**Funding:**

Bill & Melinda Gates Foundation.

## Introduction

Deaths due to unintentional carbon monoxide poisoning are often preventable.^1^, ^2^ Quantification of mortality from carbon monoxide poisoning is essential to draw attention to this threat and reduce unnecessary deaths.^1^, ^3^ To our knowledge, the Global Burden of Diseases, Injuries, and Risk Factors Study (GBD) is the only study that has estimated carbon monoxide poisoning mortality globally. Other studies have sought to estimate the morbidity and mortality associated with carbon monoxide poisoning at the country or regional level and to identify risk factors for such poisoning events. However, many of these studies have been done only in countries in which high-quality mortality data are available, focused on a single country, or relied on previous iterations of the GBD.^4^, ^5^, ^6^, ^7^, ^8^, ^9^, ^10^, ^11^, ^12^, ^13^, ^14^, ^15^, ^16^, ^17^, ^18^, ^19^ Due to differences in the time periods covered, case definitions, and International Classification of Diseases (ICD) coding systems used, the ability to make comparisons across studies is limited.

Carbon monoxide is a poisonous gas that is odourless, colourless, and tasteless, making it undetectable to the human senses.^20^, ^21^ It is produced by the incomplete combustion of fuels, such as wood, coal, and natural gas. Common household sources include heating and cooking equipment, such as furnaces, wood stoves, charcoal grills, gas stoves and heaters, and generators, as well as vehicle exhaust.^1^, ^2^, ^3^, ^21^ Unintentional fatal carbon monoxide poisonings mostly occur within residences and are more common during the winter months when heating systems are employed, windows are closed, and ventilation is reduced.^2^, ^5^ High levels of carbon monoxide or prolonged periods of exposure to the gas can quickly lead to severe bodily harm through multiple mechanisms, such as interrupting the delivery of oxygen throughout the body, impeding the use of oxygen, causing oxidative stress, or impairing cellular function.^21^, ^22^, ^23^ Mild carbon monoxide poisoning often presents with non-descript symptoms, such as nausea, headache, and fatigue, and more severe poisoning can manifest in several ways, including loss of consciousness, acute cardiac events, and death.^20^, ^21^, ^22^ Although prompt removal from the source of exposure and hyperbaric oxygen therapy have been recommended as two strategies for reversing the effects of, and reducing lasting harm due to, carbon monoxide poisoning, there is currently no true antidote, underscoring the importance of prevention.^22^, ^23^

**Research in context**

**Evidence before this study**

Previous studies have described single or multinational patterns and trends of unintentional carbon monoxide poisoning deaths, and have identified common risk factors such as age, sex, weather, heating source, and type of cooking equipment. Studies commonly found that males and older people were at greater risk of death due to carbon monoxide poisoning. These studies were identified opportunistically, via PubMed and Google Scholar, using terms such as “carbon monoxide poisoning” and “mortality” or “epidemiology” or “risk factors”. Studies were also shared with us by expert collaborators. However, existing studies use inconsistent versions or sets of codes from the International Classification of Diseases (ICD) system to identify carbon monoxide poisoning. Furthermore, most identified studies focused on high-income locations and reported findings for different age groups or time periods, making it difficult to make comparisons between studies or discern broad temporal and spatial trends; although, a few national-level studies have shown decreases in carbon monoxide poisoning mortality over the past 20–30 years. Previous work has shown that increasing public awareness about the sources of carbon monoxide and the use of carbon monoxide alarms can reduce the burden of unintentional carbon monoxide poisoning.

**Added value of this study**

This Global Burden of Diseases, Injuries, and Risk Factors Study (GBD) reports trends in global mortality due to unintentional carbon monoxide poisoning from 2000 to 2021, geographically and by age and sex, using vital registration data gathered from all available mortality databases. Unintentional carbon monoxide poisoning mortality was first estimated as part of the GBD in GBD 2017. Compared with previous iterations of the GBD, these results include new input data sources and improved data processing and mortality estimation methods to better account for miscoding in cause of death data and handling of small numbers. We focus on trends over the past two decades, in which vital registrations in most countries had transitioned to ICD-10 coding. Drawing on more than 3000 data sources, we found approximately a 50% decrease in the global age-standardised mortality rate due to unintentional carbon monoxide poisoning from 2000 to 2021. Mortality due to unintentional carbon monoxide poisoning was greater among males than among females. The percentage of deaths attributable to occupational injuries and high alcohol use was higher for males than females, and varied by age. Although unintentional carbon monoxide poisoning events tend to be more common in places with cold winters, they still occur across all locations and affect people of all ages. We also estimated years of life lost (YLLs) due to carbon monoxide poisoning. We found that while children younger than 5 years have lower mortality rates than adults, this young age group contributes similar numbers of YLLs to those estimated for adults.

**Implications of all the available evidence**

From this analysis, we have identified the locations, ages, and sexes in which mortality due to unintentional carbon monoxide poisoning is highest and most deserving of public health attention. Unintentional carbon monoxide poisoning is a largely preventable cause of death. Future work to increase data collection and public awareness and interventions to reduce carbon monoxide poisoning need to be prioritised, especially in populations most at risk.

Drawing on the most recent iteration of the GBD, we aim to provide a complete time series of mortality estimates for unintentional carbon monoxide poisoning using a common case definition and a standardised modelling approach, globally, for all ages and both sexes, from 2000 to 2021, highlighting countries, regions, and demographic groups at greatest risk of death. This manuscript was produced as part of the GBD Collaborator Network and in accordance with the GBD Protocol.

## Methods

### Overview

The GBD is a comprehensive effort to quantify health loss by age and sex for 371 diseases and injuries and 88 risk factors, across 204 countries and territories, from 1990 to the present. This latest iteration of the GBD includes crucial updates such as new input data sources and improved data processing and mortality estimation methods for miscoding in cause of death data and handling of small numbers. Mortality due to unintentional carbon monoxide poisoning was quantified through a systematic modelling process informed by vital registration records and covariates with a relationship to carbon monoxide poisoning. Details of this framework are described elsewhere,^24^, ^25^ but an overview of methods specific to poisoning by carbon monoxide follows. Regional estimates were computed with estimates from more detailed locations in accordance with the GBD location hierarchy, which consists of seven super-regions, 21 regions, and 204 countries and territories, and was constructed by grouping together countries on the basis of geographical proximity and shared epidemiologically relevant characteristics (appendix pp 6–7).

GBD complies with the Guidelines for Accurate and Transparent Health Estimates Reporting statement (appendix pp 4–5).^26^ Input data sources are available online and in the appendix (pp 58–120). Statistical code used for GBD estimation is available online.

### Input data and data processing

Three main steps were to prepare cause of death data for poisoning by carbon monoxide. First, all available data were identified and assigned to GBD causes of death (appendix pp 7–8). Globally, 3807 data sources were used as input data for the modelling of unintentional carbon monoxide poisoning deaths (appendix pp 58–120). Vital registration data constituted the majority of these sources (97·8%), and the remainder were verbal autopsy studies. However, because this cause of death is not part of the algorithms to classify verbal autopsy data, and most datapoints from these sources reported zero deaths, we marked all verbal autopsy data (40 data sources with 2802 age-specific and sex-specific datapoints) as outliers. The ICD codes mapped to poisoning by carbon monoxide were E862.0–E862.9, E868.0–E868.9, and E869.9 for ICD-9, and X47.0–X47.9 for ICD-10. Hence, this study only captured unintentional non-fire-related carbon monoxide poisoning, and explicitly excludes intentional carbon monoxide poisoning (eg, suicide) and unintentional fire-related carbon monoxide poisoning. This study reports only on 2000–21 data and therefore does not include many years of ICD-9-coded data; this decision was made to emphasise recent trends and reduce the effect of inconsistencies in the coding method. Several countries and geographical regions had scarce data for carbon monoxide poisoning, including much of sub-Saharan Africa, south Asia, and southeast Asia (appendix pp 35–36).

Second, poorly defined causes were reassigned to other underlying causes of death through garbage code redistribution. This process was used when the underlying cause of death in vital registration data was vague or assigned to a cause that should not be considered the true underlying cause of death (eg, heart failure, or injuries of undetermined intent). Garbage code redistribution was done by age, sex, country, year, and ICD system; this method is complex and variable by type of garbage code, but generally requires estimating the proportions of each group of garbage codes that should be reassigned to true underlying causes of death based on available information (eg, patterns in multiple cause of death data; appendix pp 8–11).^25^, ^27^ Approximately 17% of all deaths in the carbon monoxide poisoning input data were redistributed from ICD codes that were considered garbage codes; visualisations for example countries are available in the appendix (pp 12–15).

Third, a Bayesian noise-reduction algorithm was used to apply an adjustment to zero counts and correct for stochasticity in small populations in the cause of death data (appendix pp 16–18).^25^ This method was implemented by cause, sex, and country, and ensured that datapoints of zero deaths were not dropped when modelling in natural logarithm or logit space.

### Modelling

The GBD Cause of Death Ensemble modelling (CODEm) framework was used to produce carbon monoxide poisoning mortality estimates for every age, sex, GBD location, and year.^24^, ^25^ On the basis of previous knowledge of associations with mortality for the cause, covariates were included in the model to help inform estimates in locations with sparse or absent data (appendix pp 35–36). CODEm is designed to create a diverse set of models on the basis of mortality rates or cause fractions, with mixed effect models or spatiotemporal Gaussian process regression and a selection of covariates (appendix pp 20–22).^24^, ^25^ All possible combinations of covariates were tested in a covariate selection process, but only those with statistical significance (p<0·05) and that maintained the covariate priors were chosen; not all covariates were necessarily selected to inform the final model. Of the seven covariates (alcohol consumption [litres per capita]; education [years per capita]; health-care access and quality index; lag-distributed income per capita; sociodemographic index; a summary exposure value of the risk factors for carbon monoxide poisoning; and temperature [mean, population-weighted]) included in CODEm for poisoning by carbon monoxide, two covariates were selected consistently across all models: mean temperature and lag-distributed income. Mean temperature showed the greatest influence on model estimates (appendix p 37). As part of the model testing process, data were held out based on cause-specific patterns of missingness, and out-of-sample predictive validity was assessed for each model and then used to create weighted combinations (ensembles) of models; ultimately, the ensemble with the highest out-of-sample predictive validity was chosen (appendix pp 22–23).^24^ Separate models were run for countries considered to be data-rich and for all countries with all available data (appendix pp 18–19).^25^ Uncertainty was propagated throughout by taking draws from the posterior distribution and computing the mean and 95% uncertainty interval (UI) across the draws at the end of the estimation process. After models were finalised, deaths were first rescaled across poisoning by carbon monoxide and poisoning by other means to sum to the number of deaths from the aggregate poisoning cause, and then with all other causes of death to ensure that the sum of deaths across all causes was equivalent to all-cause mortality estimates.^25^ This ensures consistency and allows for comparisons to be made across ages, sexes, locations, and years, and between GBD causes, where similar methods are applied.

Years of life lost (YLLs) due to unintentional carbon monoxide poisoning were computed by multiplying the number of deaths by the remaining standard life expectancy at the age of death on the basis of the GBD standard model life table.^25^, ^28^ Age-standardised rates were computed with age-specific weights derived from the GBD standard population to calculate a weighted average, enabling cross-country comparison.^25^, ^28^

Occupational injuries and high alcohol use are the only two risk factors currently associated with mortality due to unintentional carbon monoxide poisoning in the GBD risk factor framework. Population attributable fractions (PAFs), or the proportion of unintentional carbon monoxide poisoning deaths attributable to associated risk factors, were calculated for high alcohol use and occupational injuries (appendix pp 24–32).^29^

### Role of the funding source

The funder of the study had no role in study design, data collection, data analysis, data interpretation, or writing of the manuscript.

## Results

### Global mortality

**Figure 1:**
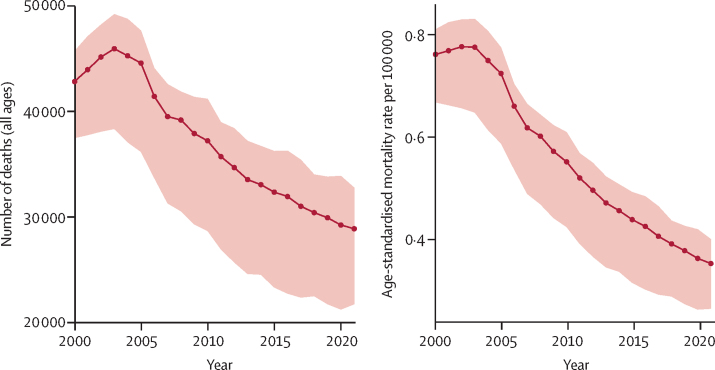
Global age-standardised mortality rate and number of deaths due to unintentional carbon monoxide poisoning, 2000–21 Shading indicates the upper and lower limits of the 95% uncertainty intervals.

In 2021, the global age-standardised carbon monoxide poisoning mortality rate was 0·353 per 100 000 (95% UI 0·265–0·401), with a total of 28 900 deaths (21 700–32 800) and 1 180 000 YLLs (886 000–1 350 000) worldwide (table; figure 1; appendix p 39). Mortality due to carbon monoxide poisoning decreased by 53·5% (46·2–63·7) over the two-decade period, from an age-standardised mortality rate of 0·761 per 100 000 (0·668–0·810) in 2000 (table; figure 1). YLLs also decreased, from 35·8 age-standardised YLLs per 100 000 (29·9–38·1) in 2000 to 14·9 per 100 000 (11·1–17·0) in 2021 (a decrease of 58·1% [52·8–66·4]; appendix p 39).

**Table:** Change in global, super-regional, and regional deaths and age-standardised mortality due to unintentional carbon monoxide poisoning, 2000–21

		**Number of deaths, 95% UI**			**Age-standardised mortality rate per 100 000, 95% UI**		
		2000	2021	Percentage change, 2000–21	2000	2021	Percentage change, 2000–21
Global		42 800 (37 500 to 45 700)	28 900 (21 700 to 32 800)	−32·5% (−47·3 to −21·9)	0·761 (0·668 to 0·810)	0·353 (0·265 to 0·401)	−53·5% (−63·7 to −46·2)
Central Europe, eastern Europe, and central Asia		20 600 (20 300 to 20 900)	7110 (6660 to 7620)	−65·4% (−67·7 to −63·1)	4·55 (4·48 to 4·63)	1·39 (1·30 to 1·49)	−69·4% (−71·3 to −67·4)
	Central Asia	2120 (1950 to 2250)	918 (803 to 1040)	−56·6% (−61·2 to −50·7)	3·29 (3·03 to 3·50)	0·988 (0·865 to 1·12)	−70·0% (−73·0 to −66·0)
	Central Europe	1430 (1390 to 1470)	582 (539 to 626)	−59·3% (−61·6 to −56·5)	1·08 (1·04 to 1·11)	0·360 (0·335 to 0·389)	−66·1% (−68·1 to −63·6)
	Eastern Europe	17 000 (16 800 to 17 200)	5610 (5180 to 6080)	−67·1% (−69·6 to −64·5)	6·83 (6·75 to 6·91)	2·12 (1·98 to 2·30)	−68·9% (−71·0 to −66·6)
High income		2250 (2170 to 2370)	2430 (2320 to 2510)	7·9% (2·5 to 10·8)	0·209 (0·203 to 0·221)	0·186 (0·180 to 0·191)	−11·2% (−15·3 to −8·5)
	Australasia	40·2 (38·6 to 42·2)	28·1 (26·7 to 29·6)	−30·2% (−34·1 to −25·3)	0·171 (0·164 to 0·179)	0·0833 (0·0800 to 0·0874)	−50·9% (−53·8 to −47·0)
	High-income Asia Pacific	430 (397 to 539)	310 (279 to 396)	−27·9% (−36·8 to −21·1)	0·207 (0·191 to 0·259)	0·122 (0·111 to 0·161)	−40·8% (−49·0 to −35·7)
	High-income North America	821 (796 to 838)	1310 (1260 to 1350)	59·4% (52·8 to 64·6)	0·241 (0·235 to 0·246)	0·314 (0·303 to 0·324)	30·4% (24·8 to 34·9)
	Southern Latin America	245 (237 to 251)	247 (236 to 260)	0·9% (−4·6 to 6·9)	0·455 (0·440 to 0·468)	0·342 (0·326 to 0·360)	−24·5% (−28·7 to −20·2)
	Western Europe	715 (689 to 732)	536 (503 to 560)	−25·1% (−27·8 to −23·1)	0·150 (0·145 to 0·153)	0·0860 (0·0825 to 0·0889)	−42·5% (−43·8 to −41·2)
Latin America and Caribbean		540 (514 to 570)	571 (513 to 628)	5·6% (−3·1 to 15·7)	0·129 (0·123 to 0·136)	0·0965 (0·0860 to 0·107)	−25·2% (−31·9 to −18·0)
	Andean Latin America	27·1 (17·1 to 32·3)	46·4 (31·0 to 58·7)	71·3% (40·6 to 114·3)	0·0632 (0·0415 to 0·0736)	0·0715 (0·0477 to 0·0903)	12·8% (−7·3 to 39·9)
	Caribbean	47·7 (35·2 to 63·5)	44·9 (29·8 to 64·7)	−5·8% (−26·2 to 19·6)	0·136 (0·0995 to 0·184)	0·106 (0·0668 to 0·156)	−23·0% (−39·4 to −3·0)
	Central Latin America	405 (392 to 418)	411 (370 to 458)	1·6% (−8·0 to 11·5)	0·231 (0·224 to 0·239)	0·165 (0·148 to 0·184)	−28·7% (−35·6 to −21·8)
	Tropical Latin America	60·7 (58·3 to 63·8)	68·1 (64·6 to 71·6)	12·3% (6·6 to 18·5)	0·0370 (0·0352 to 0·0389)	0·0287 (0·0272 to 0·0302)	−21·6% (−25·1 to −17·6)
North Africa and Middle East		2610 (1470 to 3340)	2780 (1480 to 3630)	6·6% (−14·3 to 29·4)	0·700 (0·409 to 0·889)	0·487 (0·264 to 0·629)	−30·3% (−44·1 to −16·2)
South Asia		1170 (622 to 1640)	1190 (673 to 1530)	1·4% (−19·2 to 33·9)	0·0965 (0·0557 to 0·128)	0·0681 (0·0386 to 0·0879)	−29·8% (−41·8 to −8·5)
Southeast Asia, east Asia, and Oceania		15 200 (11 400 to 17 600)	14 300 (8360 to 17 300)	−6·0% (−43·4 to 20·6)	0·942 (0·694 to 1·08)	0·593 (0·349 to 0·711)	−36·3% (−60·2 to −19·1)
	East Asia	14 500 (11 000 to 17 000)	13 500 (7690 to 16600)	−6·6% (−45·4 to 19·1)	1·30 (0·955 to 1·49)	0·802 (0·465 to 0·962)	−37·6% (−61·7 to −21·8)
	Oceania	17·8 (7·71 to 30·5)	24·6 (11·4 to 45·6)	37·7% (−8·8 to 110·4)	0·245 (0·105 to 0·415)	0·195 (0·0886 to 0·383)	−20·5% (−43·4 to 14·1)
	Southeast Asia	676 (264 to 901)	710 (289 to 925)	5·1% (−20·1 to 45·4)	0·136 (0·0533 to 0·183)	0·101 (0·0413 to 0·131)	−25·8% (−42·6 to 1·6)
Sub-Saharan Africa		502 (339 to 1290)	552 (261 to 1640)	9·9% (−28·8 to 56·0)	0·115 (0·0724 to 0·289)	0·0750 (0·0342 to 0·235)	−35·0% (−59·6 to −10·2)
	Central sub-Saharan Africa	60·5 (21·0 to 229)	75·3 (14·7 to 308)	24·5% (−55·4 to 99·3)	0·117 (0·0427 to 0·432)	0·0905 (0·0178 to 0·368)	−23·2% (−70·1 to 10·7)
	Eastern sub-Saharan Africa	205 (83·8 to 693)	212 (56·2 to 890)	3·3% (−54·2 to 50·8)	0·136 (0·0500 to 0·478)	0·0863 (0·0217 to 0·373)	−36·6% (−69·4 to −8·1)
	Southern sub-Saharan Africa	135 (75·6 to 189)	141 (90·3 to 207)	4·4% (−24·1 to 46·0)	0·214 (0·120 to 0·297)	0·178 (0·118 to 0·256)	−18·6% (−40·4 to 13·6)
	Western sub-Saharan Africa	102 (56·6 to 206)	123 (71·9 to 315)	21·6% (−25·3 to 85·0)	0·0762 (0·0432 to 0·153)	0·0485 (0·0288 to 0·120)	−36·3% (−60·7 to −2·2)

Estimates were computed at the draw level to propagate uncertainty; manual calculations (eg, for percentage change or grouped locations) might result in slight differences. Count and rate data are presented to three significant figures. UI=uncertainty interval.

### Global differences by sex and age

**Figure 2:**
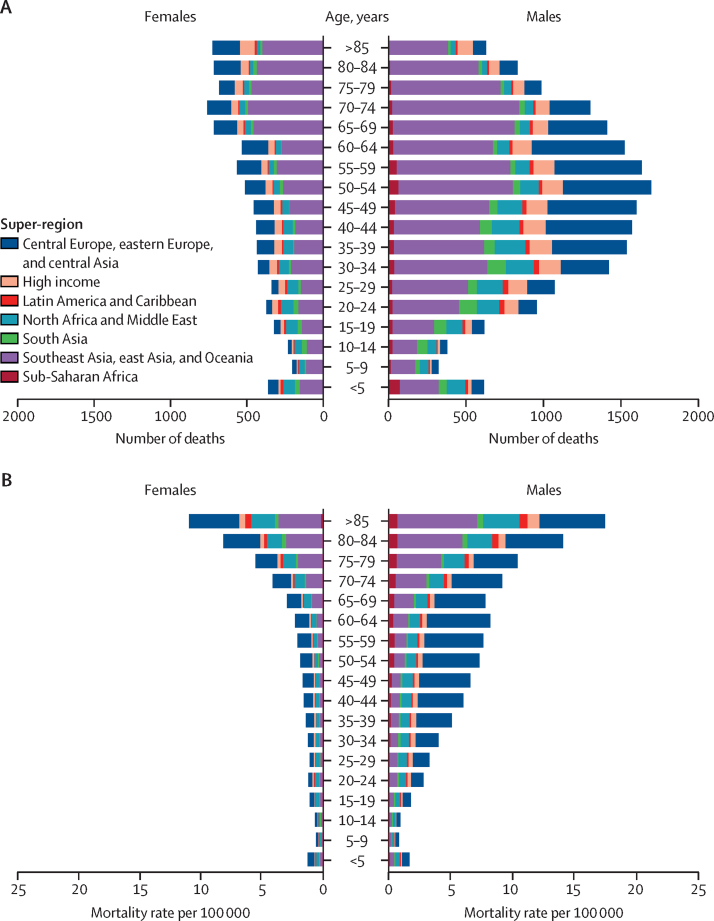
Age-specific deaths (A) and mortality rates (B) due to unintentional carbon monoxide poisoning, by sex and GBD super-region, 2021 GBD=Global Burden of Diseases, Injuries, and Risk Factors Study.

**Figure 3:**
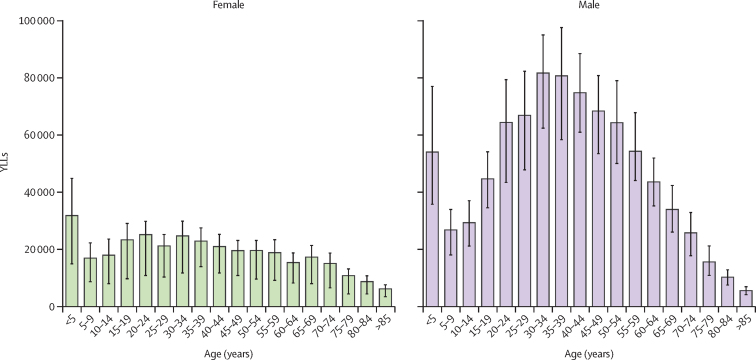
Global YLLs due to unintentional carbon monoxide poisoning, by age and sex, 2021 Error bars represent the 95% uncertainty interval. YLLs=years of life lost.

In 2021, the global mortality rate was 0·508 per 100 000 (95% UI 0·399–0·606) for males and 0·223 per 100 000 (0·109–0·259) for females (appendix p 40). There were 20 100 (15 800–24 000) male deaths and 8780 (4300–10 200) female deaths in 2021 (appendix p 40). The 50–54-year age group had the greatest number of deaths (2210 [1660–2590]; figure 2A; appendix p 40). The greatest number of male deaths by age occurred in the 50–54-year age group (1690 [1320–2080]), whereas the greatest number of female deaths occurred in the 70–74-year age group (759 [327–945]) in 2021 (figure 2A; appendix p 40). The highest mortality rate in 2021 was in those aged 85 years or older, with 1·96 deaths (1·38–2·32) per 100 000 (appendix p 40). A greater number of deaths occurred in every age group for males than for females, with the exception of those aged 85 years or older (figure 2A; appendix p 40). Similarly, males had greater mortality rates than females at every age; however, the largest difference was in the 40–44-year age group, in which the male mortality rate was 3·5-times greater than the female mortality rate (0·623 per 100 000 [0·507–0·737] *vs* 0·176 per 100 000 [0·0981–0·213]; figure 2B; appendix p 40). The 30–34-year age group had the largest number of YLLs (107 000 [77 100–120 000]) due to carbon monoxide poisoning in 2021 (figure 3; appendix p 41). The under-5 years age group contributed a similar number of YLLs (86 000 [54 200–115 000]) to the 45–49-year (88 000 [67 700–102 000]) and 50–54-year (84 000 [63 100–98 400]) age groups, despite having much lower mortality rates (figure 3; appendix p 41).

### Geographical patterns

Regionally, eastern Europe had the highest mortality rate due to carbon monoxide poisoning at 2·12 deaths per 100 000 (95% UI 1·98–2·30) and contributed 5610 deaths (5180–6080) in 2021 (table). This accounted for approximately 19% of all carbon monoxide poisoning deaths globally that year, despite only about 3% of the world's population residing in eastern Europe. The mortality rate in eastern Europe was more than two times greater than that of the region with the next-highest mortality rate, central Asia, which had a mortality rate of 0·988 deaths per 100 000 (0·865–1·12; table). However, eastern Europe saw one of the greatest reductions in carbon monoxide poisoning fatality, with a decrease of 68·9% (66·6–71·0) in the age-standardised mortality rate from 2000 (6·83 per 100 000 [6·75–6·91]) to 2021 (table). Central Asia and central Europe also had notable declines in mortality rates due to unintentional carbon monoxide poisoning from 2000 to 2021 (70·0% [66·0–73·0] in central Asia and 66·1% [63·6–68·1] in Europe; table). By contrast, high-income North America had a 30·4% (24·8–34·9) increase in mortality relative to 2000, with an age-standardised mortality rate of 0·314 per 100 000 (0·303–0·324) in 2021 (table). The regions with the lowest unintentional carbon monoxide poisoning mortality rates in 2021 were tropical Latin America (0·0287 deaths per 100 000 [0·0272–0·0302]) and western sub-Saharan Africa (0·0485 deaths per 100 000 [0·0288–0·120]; table).

**Figure 4:**
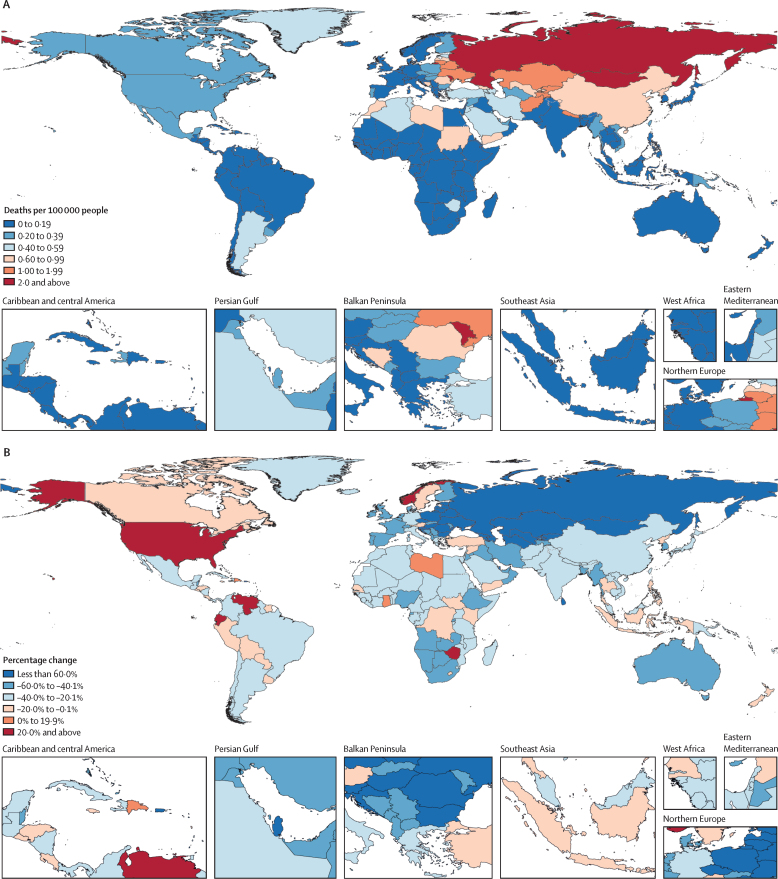
Country-specific, age-standardised mortality rate due to unintentional carbon monoxide poisoning in 2021 (A), and percentage change from 2000 to 2021 (B)

In 2021, the country with the highest unintentional carbon monoxide poisoning mortality rate was Moldova, with 2·74 deaths per 100 000 (95% UI 2·42–3·04), followed by Mongolia with 2·48 deaths per 100 000 (1·89–3·30) and Russia with 2·36 deaths per 100 000 (2·22–2·51; figure 4A; appendix pp 42–49). Eight of the countries (Moldova, Mongolia, Russia, Kazakhstan, Ukraine, Belarus, Lithuania, and Tajikistan) with the ten highest mortality rates in 2021 also showed substantial declines since 2000, with decreases of at least 50% (figure 4; appendix pp 42–49). Afghanistan and Nepal were the only countries in the top ten in terms of age-standardised mortality rates in 2021 that were not part of central Europe, eastern Europe, and central Asia. Mongolia had the greatest age-standardised YLL rate in 2021, at 113 per 100 000 (85·3–151), followed closely by Moldova (112 per 100 000 [98·1–126]) and Russia (107 per 100 000 [101–113]), which were the only countries with more than 100 YLLs per 100 000 due to unintentional carbon monoxide poisoning (appendix pp 50–57).

### Risk factors

The PAFs for occupational injuries as a risk factor for carbon monoxide poisoning mortality were highest globally in 2021 for both sexes in the 25–29-year age group, at 31·6% (95% UI 27·3–37·3) for males, and 21·5% (17·2–27·9) for females (appendix p 38). Male occupational injury PAFs were greater than female occupational injury PAFs, across all ages. The PAFs for high alcohol use as a risk factor for carbon monoxide poisoning mortality were highest in males aged 25–44 years, with PAFs between 6·1% and 6·2%, for all 5-year age groupings in that range (appendix p 38). PAFs for high alcohol use among males were greater than among females, across all ages (appendix p 38).

## Discussion

Drawing on thousands of data sources and a systematic modelling strategy to describe global patterns of unintentional carbon monoxide poisoning, we found that globally, unintentional carbon monoxide poisoning fatalities have decreased substantially over the past 21 years, with an age-standardised mortality rate in 2021 that is nearly half that in 2000. However, this decrease was driven by major improvements in a few select regions. Although 28 900 deaths from unintentional carbon monoxide poisoning constitute a relatively small proportion of all deaths in 2021, these are largely preventable.

Regionally, eastern Europe and central Asia contributed the most to fatality due to carbon monoxide poisoning, but also showed some of the greatest improvements over time. High mortality rates in these regions are likely to be partly attributable to temperature, as countries in these regions tend to have longer, colder winters relative to many other parts of the world.^30^, ^31^ Poorly ventilated indoor spaces combined with faulty heating systems can lead to accumulation of dangerous levels of carbon monoxide indoors.^5^, ^6^, ^30^ Additionally, use of alternative heating or cooking equipment (eg, charcoal grills and portable generators) indoors during power outages caused by winter storms is a common source of carbon monoxide.^32^, ^33^ We found that the most influential covariate on the cause of death model estimates for carbon monoxide poisoning was temperature, exhibiting a strong negative relationship with lower temperatures corresponding to greater mortality. This corroborates what has been reported in other studies; however, the exposure–outcome pair of low temperature and carbon monoxide poisoning was not formally assessed in the GBD risk factor framework to confirm an association.^15^, ^29^, ^34^, ^35^ High-income North America was one of only two regions that saw a substantial increase in the carbon monoxide poisoning mortality rate from 2000 to 2021, driven by country-level estimates from the USA. This result is supported by a report showing that unintentional non-fire-related carbon monoxide deaths associated with consumer products have increased significantly over the period of 2009–19 in the USA.^36^ However, other studies have shown that unintentional carbon monoxide poisonings have plateaued or decreased during this period in the USA.^8^, ^13^, ^37^ These variations are attributable to different case definitions and methods, such as single-coded versus dual-coded data. Stagnating trends have been identified in other parts of the world, where decreases in mortality have stalled in recent years despite the preventable nature of this cause of death.^5^, ^7^

At nearly every age, males died of carbon monoxide poisoning at a rate at least two times greater than females. This finding is in line with what has been reported in other studies.^5^, ^8^, ^12^, ^15^ Males might be more likely to inadvertently put themselves at risk of carbon monoxide poisoning when operating machinery, using fuel-burning appliances (eg, grilling), or being exposed in the workplace.^13^, ^38^, ^39^ This is reinforced by our risk factor analysis for occupational injuries. Although carbon monoxide poisoning deaths in the workplace have decreased due to more rigorous occupational regulations and carbon monoxide poisoning alarm requirements, some risk associated with occupational settings remains.^20^, ^38^, ^39^, ^40^ Additionally, alcohol use increases the risk of carbon monoxide poisoning due to reduced inhibitions and poor decision making. Individuals under the influence of alcohol might also be less likely to recognise early symptoms of carbon monoxide poisoning.^13^, ^41^ A few studies have also found that females have an ability to eliminate carbon monoxide more rapidly, leading to better prognosis after poisoning.^42^, ^43^ Higher death rates among the oldest age groups could be attributed to existing comorbidities that make this population more susceptible to the effects of carbon monoxide poisoning.^10^, ^44^ Older people might also be at increased risk because they are more likely to live in older homes without updated appliances or carbon monoxide alarms.^5^, ^21^ Although there were a small number of deaths in the youngest age groups, a significant proportion of all YLLs came from these ages as they constituted very premature deaths.

Most carbon monoxide poisonings occur in the home, with some studies reporting that as many as 80% happen within residences.^5^, ^6^, ^12^ Out-of-date, ill-maintained, or incorrectly installed heating systems have been linked to many cases of residential unintentional carbon monoxide poisoning.^6^, ^31^ Homes that rely solely on biomass fuels for heating or cooking can put residents at high risk for carbon monoxide poisoning.^30^ Even in warmer climates or in warmer months, carbon monoxide poisoning is possible under such circumstances.^45^ A study from South Korea showed that a transition away from wood and charcoal as the most commonly used fuels led to a drastic decrease in carbon monoxide poisoning deaths.^18^ Even though data are scarce on acute carbon monoxide poisoning incidents and fatalities in parts of sub-Saharan Africa, a few studies have found elevated levels of carbon monoxide indoors, well above the WHO recommended thresholds.^46^, ^47^ These studies noted that, particularly in rural areas, switching to clean fuels might not be an immediately feasible solution, and recommended addressing housing characteristics in other ways (eg, improving home ventilation by increasing window area) in the short term.^47^ Increased public health education about the sources and dangers of carbon monoxide, and the risks of using alternative heating and cooking equipment indoors, could help to reduce the occurrence of severe carbon monoxide poisoning events under these conditions.^2^, ^32^, ^48^, ^49^ Education of health professionals to recognise the signs of carbon monoxide poisoning can also help to reduce the risk of future, more severe poisoning events.^49^, ^50^ Additionally, when used properly, carbon monoxide alarms are cost-effective tools for preventing severe outcomes of carbon monoxide poisoning, and increased education and legislation around their use could further reduce mortality.^5^, ^41^, ^51^, ^52^ Findings from studies looking at the effectiveness of carbon monoxide alarms have been mixed and do not always show significant decreases in poisoning events, but these studies often cite scarce data and inconsistencies in usage as challenges to conclusive evidence.^8^, ^52^ Laws around carbon monoxide alarm use are highly variable across countries; if such laws exist, they often require only newly constructed residential homes and apartment buildings to contain carbon monoxide alarms. Even with laws mandating carbon monoxide alarm installation in most of the USA, only an estimated 50% of homes have carbon monoxide alarms in working order.^48^, ^53^ Further research is necessary to pinpoint the specific catalysts and inform future priority setting and resource allocation for preventive interventions.

Data challenges constitute many of the limitations of this analysis. First, without data in many countries, our ability to estimate carbon monoxide poisoning was severely affected. Where data were scarce, our cause of death models relied on covariates and regional data to estimate mortality.^24^, ^25^ Although the ability to make informed predictions in the absence of data is an advantage of the GBD methods, it can also introduce biases. Second, although the GBD addresses miscoding in vital registration data through garbage code redistribution, incorrect assignment in cause of death data could affect the estimation of deaths due to carbon monoxide poisoning. For example, deaths due to intentional carbon monoxide poisoning could have inadvertently been coded as unintentional, or a comorbid condition could have been listed as the underlying cause of death.^13^ Third, this study only looked at trends after 2000 to reduce the influence of ICD-9-coded data on the analysis, given some of the inconsistencies between poisoning-related codes in ICD-9 versus ICD-10.^3^, ^5^ However, not all countries had switched to ICD-10 by 2000, and ICD-9-coded data could still have influenced the estimates. Fourth, accurately identifying deaths by carbon monoxide poisoning is challenging without a thorough autopsy or strong circumstantial evidence. Many studies have reported that the true number of deaths due to carbon monoxide poisoning might be greater than that captured in the data, particularly in countries without high-quality vital registration systems; however, other evidence suggests that deaths due to carbon monoxide poisoning could be over-counted.^3^, ^5^, ^7^, ^18^ Best practice for ICD-10 cause of death coding is to use the cause of injury ICD code, X47, in conjunction with the nature of injury code T58, as supplemental information; however, most data sources available to the GBD do not contain this level of detail.^3^ Additionally, not all countries provide the four-digit detail for the cause of injury code X47 itself (ie, specifying one of X47.0–X47.9), affecting our ability to draw further information about the circumstances surrounding these deaths. Fifth, it was not possible to capture within-country variation, and between-country variation could have been masked by regional estimates. Risk factors for carbon monoxide poisoning can vary greatly, both between and within countries, and by season, climate, and demographic, which might have been masked in this study. Sixth, not all underlying data sources for both risk factors in the risk factor analysis provided detailed age, sex, or cause of injury information, requiring some degree of extrapolation to produce carbon-monoxide-specific results.^29^ Both risk factors were estimated for people aged 15 years or older, and as such cannot help to explain patterns seen among children. Currently, only two risk factors are associated with mortality due to carbon monoxide poisoning in the GBD risk factor framework, which means that important associations, such as low temperature, are not captured; we hope to address this in the future. Seventh, this study was unable to examine the effect of the COVID-19 pandemic. Unintentional carbon monoxide poisonings might have increased due to increased time at home, and initial studies suggested that those who had contracted COVID-19 might be at increased risk for poisoning, but more data will be necessary to reach conclusions.^54^ Finally, because this study only examined unintentional non-fire-related carbon monoxide poisoning mortality, it does not capture the burden of carbon monoxide poisoning more broadly. Other studies have noted that the distribution of types of carbon monoxide poisoning can vary greatly between locations, and approaches to prevention might differ depending on the cause.^5^, ^8^, ^12^

In conclusion, deaths due to unintentional carbon monoxide poisoning are largely preventable. Despite this, approximately 30 000 people die annually worldwide from carbon monoxide poisoning. Although unintentional carbon monoxide poisoning mortality has decreased over the past 21 years, the decline has not been consistent over time or across regions. The need for increased public health surveillance to fully understand the scope of the problem is great. Improved accessibility of safe heating and cooking equipment, increased use of carbon monoxide alarms, and public education to understand the causes of, and risks associated with, carbon monoxide exposure are needed to spur further reductions in mortality.

## Data sharing

Our study follows the Guidelines for Accurate and Transparent Health Estimates Reporting. The findings of this study are supported by data available in public online repositories, data publicly available upon request of the data provider, and data not publicly available due to restrictions by the data provider. Non-publicly available data were used under license for the current study but might be available from the authors upon reasonable request and with permission of the data provider. Data sources used in this analysis are listed in the appendix (pp 58–120) and on the Global Health Data Exchange website.

## Declaration of interests

S Afzal reports payment or honoraria from educational events and webinars with King Edward Medical University, Lahore, Pakistan, and collaborative partners including: Johns Hopkins University, Baltimore, MD, USA; University of California, CA, USA; University of Massachusetts, Boston, MA, USA; and University of Lahore, Lahore, Pakistan; participation on a data safety monitoring board or advisory board with the National Bioethics Committee Pakistan, King Edward Medical University Institutional Ethical Review Board, Fatima Jinnah Medical University, Lahore, Pakistan; and Sir Ganga Ram Hospital, Delhi, India; leadership or fiduciary roles in board, society, committee, or advocacy groups, paid or unpaid, with the Pakistan Association of Medical Editors, Fellow of Faculty of Public Health Royal Colleges UK, Society of Prevention, Advocacy and Research, King Edward Medical University, and as a member of the Pakistan Society of Infectious Diseases; outside the submitted work. M Carvalho reports grants or contracts from Fundação para a Ciência e a Tecnologia in the scope of the project UIDP/04378/2020 and UIDB/04378/2020 of the Research Unit on Applied Molecular Biosciences, and project LA/P/0140/2020 of i4HB; outside the submitted work. R C Franklin reports grants or contracts from Heatwaves in Queensland, Queensland Government; Arc Flash, Human Factors, Queensland Government; and Mobile Plant Safety, AgriFutures; honoraria from the World Safety Conference 2022- Conference Convener; support for attending the ACTM Tropical Medicine and Travel Medicine Conference 2022 and ISTM Travel Medicine Conference 2023 in Basel; leadership or fiduciary roles in board, society, committee, or advocacy groups, paid or unpaid, with Kidsafe as a Director, Auschem as a Director, ISASH on the Governance Committee, Farmsafe as a Director, and PHAA Injury Prevention SIG as a Convenor; outside the submitted work. K Krishan reports other, non-financial support from the UGC Centre of Advanced Study, CAS II, Department of Anthropology, Panjab University, Chandigarh, India; outside the submitted work. A-F A Mentis reports funding from MilkSafe: a novel pipeline to enrich formula milk using omics technologies, a research co-financed by the European Regional Development Fund of the European Union and Greek national funds through the Operational Program Competitiveness, Entrepreneurship and Innovation, under the call Research–Create–Innovate (project code: T2EDK-02222), and from ELIDEK (Hellenic Foundation for Research and Innovation, MIMS-860); payment or expert testimony as an external peer reviewer for Fondazione Cariplo, Italy; leadership or fiduciary roles in board, society, committee, or advocacy groups, paid or unpaid, with *Systematic Reviews* journal as an editorial board member, *Annals of Epidemiology* and *Translational Psychiatry* journal as Associate Editor; and other financial or non-financial support as a scientific officer at the BGI Group; outside the submitted work. I Myers reports research grants from CO Research Trust; payment or honoraria from CO Research Trust; support for attending meetings from CO Research Trust; leadership roles in board, society, committee, or advocacy groups, unpaid, with UK All Party Parliamentary CO Group Medical Sub-Group as a Chair, UK Indoor Environments Group as a Committee Member, and OFGEM DRS-GDN as a panel member; outside the submitted work. A E Peden reports support for this paper from The Australian National Health and Medical Research Council Emerging Leadership Fellowship (APPID: APP2009306).
