# Supplementary material for: Global, regional, and national mortality due to unintentional carbon monoxide poisoning, 2000–2021: results from the Global Burden of Disease Study 2021
Source: Lancet Public Health. 2023 Oct 6;8(11):e839–49. doi: 10.1016/S2468-2667(23)00185-8 (PMC10602911; doi:10.1016/S2468-2667(23)00185-8)
Supplement: Supplementary appendix [file mmc1.pdf]

THE LANCET
Public Health

Supplementary appendix

This appendix formed part of the original submission and has been peer reviewed. We post it as supplied by the authors.

Supplement to: GBD 2021 Carbon Monoxide Poisoning Collaborators. Global, regional, and national mortality due to unintentional carbon monoxide poisoning, 2000–2021: results from the Global Burden of Disease Study 2021. *Lancet Public Health* 2023; published online Oct 6. https://doi.org/10.1016/S2468-2667(23)00185-8.

Appendix to "Global, regional, and national burden of fatal carbon monoxide poisoning 2000–2021: results from the Global Burden of Disease Study 2021"

GBD 2021 Carbon Monoxide Poisoning Collaborators

Portions of this appendix have been reproduced or adapted from Vos et al.[1], Wang et al[2], and Murray et al[3]. References are provided for these sections. We have worked with our causes of death and risk factor teams to revise the text for any updates for this latest iteration of GBD.

Table of Contents

Section 1 Guidelines for Accurate and Transparent Health Estimates Reporting (GATHER) compliance 4

Table S1 GATHER checklist 4

Section 2 Supplemental methods 6

Section 2.1 Global Burden of Disease cause hierarchy 6

Section 2.2 Global Burden of Disease location hierarchy 6

Section 2.3 Cause of death data identification[1] 7

Section 2.4 Garbage code identification[1] 8

Section 2.5 Redistribution methodology[1] 9

Section 2.5.1 Redistribution using multiple cause of death data[1] 10

Section 2.5.2 Redistribution for external cause of injury garbage codes[1] 11

Section 2.5.3 Redistribution examples for unintentional carbon monoxide poisoning 11

Section 2.6 Noise reduction[1] 16

Section 2.7 Cause of death data star rating system[1] 18

Section 2.8 Cause of death ensemble model (CODEm) overview[1] 19

Section 2.9 Covariates provided to CODEm[1] 20

Section 2.9.1 Lag-distributed income[2] 21

Section 2.9.2 Socio-demographic index[2] 21

Section 2.9.3 Healthcare Access and Quality Index 22

Section 2.10 CODEm model pool development, testing, and ensemble creation[1] 22

Section 2.11 CODEm model fit statistics[1] 22

Section 2.12 GBD risk factor estimation overview[3] 23

Section 2.13 Occupational injuries risk factor[3] 24

Section 2.13.1 Occupational injuries risk factor definition and input data[3] 24

Section 2.13.2 Occupational injuries modelling strategy[3] 25

Section 2.14 High alcohol use risk factor[3] 26

Section 2.14.1 High alcohol use risk factor definition and input data[3] 26

Section 2.14.2 High alcohol use modelling strategy[3] 28

Section 3 References 33

Section 4 Supplementary figures and tables 35

Figure S1 Input data availability for carbon monoxide poisoning mortality 35

Table S2 Super-regional, regional, and national breakdown of locations with data sources for carbon monoxide poisoning 35

Table S3 National and subnational sources in countries with subnational estimation for carbon monoxide poisoning....................36
Figure S2 Covariates selected in the carbon monoxide poisoning CODEm models and their relative influence on estimates....................37
Figure S3 Population attributable fractions (PAFs) of risk factors for carbon monoxide poisoning mortality by age and sex, 2021, with 95% uncertainty intervals (UIs) demonstrated by the error bars38
Table S4 Global and regional counts and age-standardised rates of years of life lost (YLLs) due to carbon monoxide poisoning in 2000 and 2021 for both sexes combined, and percentage change for each metric, 2000 to 2021, with 95% UIs....................39
Table S5 Global age-specific counts and rates of fatal unintentional carbon monoxide poisoning deaths, 2021, with 95% UIs....................40
Table S6 Global age-specific counts and rates of years of life lost (YLLs) due to carbon monoxide poisoning in 2021, with 95% UIs....................41
Table S7 Global and country-level counts and age-standardised rates of fatal unintentional carbon monoxide poisoning in 2000 and 2021, for both sexes, and percentage change, 2000 to 2021, for each metric, with 95% UIs....................42
Table S8 Global and country-level years of life lost (YLLs) due to unintentional carbon monoxide poisoning and age-standardised rates of YLLs due to unintentional carbon monoxide poisoning in 2000 and 2021, for both sexes, and percentage change, 2000 to 2021, for each metric, with 95% UIs........50
Table S9 List of citations for input data sources on carbon monoxide poisoning deaths............58
Section 5 Collaborating Authors and Authors' Contributions....................121
Section 5.1 GBD 2021 Carbon Monoxide Poisoning Collaborators....................121
Section 5.2 Affiliations....................122
Section 5.3 Authors' Contributions....................128

Section 1 Guidelines for Accurate and Transparent Health Estimates Reporting (GATHER) compliance

This study complies with the Guidelines for Accurate and Transparent Health Estimates Reporting (GATHER) recommendations (Table S1, appendix pp 4-5).[4]

Table S1 GATHER checklist

Item #	Checklist item	Reporting location
Objectives and funding		
1	Define the indicator(s), populations (including age, sex, and geographic entities), and time period(s) for which estimates were made.	Summary; Main text: Introduction, Methods
2	List the funding sources for the work.	Summary
Data Inputs		
For all data inputs from multiple sources that are synthesized as part of the study:		
3	Describe how the data were identified and how the data were accessed.	Main text: Methods; Appendix: Supplemental methods
4	Specify the inclusion and exclusion criteria. Identify all ad-hoc exclusions.	Main text: Methods, Results; Appendix: Supplemental methods
5	Provide information on all included data sources and their main characteristics. For each data source used, report reference information or contact name/institution, population represented, data collection method, year(s) of data collection, sex and age range, diagnostic criteria or measurement method, and sample size, as relevant.	Citations are listed on the Global Health Data Exchange (GHDx) and provided in the appendix in table S9.
6	Identify and describe any categories of input data that have potentially important biases (e.g., based on characteristics listed in item 5).	Main text: Methods, Discussion
For data inputs that contribute to the analysis but were not synthesized as part of the study:		
7	Describe and give sources for any other data inputs.	Appendix: Supplemental methods.<br><br>Citations are listed on the Global Health Data Exchange (GHDx) and provided in the appendix in table S9.
For all data inputs:		
8	Provide all data inputs in a file format from which data can be efficiently extracted (e.g., a spreadsheet rather than a PDF), including all relevant meta-data listed in item 5. For any data inputs that cannot be shared because of ethical or legal reasons, such as third-party ownership, provide a contact name or the name of the institution that retains the right to the data.	Citations are listed on the Global Health Data Exchange (GHDx) and provided in the appendix in table S9.

Data analysis		
9	Provide a conceptual overview of the data analysis method. A diagram may be helpful.	Main text: Methods
10	Provide a detailed description of all steps of the analysis, including mathematical formulae. This description should cover, as relevant, data cleaning, data pre-processing, data adjustments and weighting of data sources, and mathematical or statistical model(s).	Main text: Methods; Appendix: Supplementary methods
11	Describe how candidate models were evaluated and how the final model(s) were selected.	Main text: Methods; Appendix: Supplemental methods
12	Provide the results of an evaluation of model performance, if done, as well as the results of any relevant sensitivity analysis.	Main text: Methods; Appendix: Supplemental methods
13	Describe methods for calculating uncertainty of the estimates. State which sources of uncertainty were, and were not, accounted for in the uncertainty analysis.	Main text: Methods
14	State how analytic or statistical source code used to generate estimates can be accessed.	Code will be shared via remote repository, linked on the Global Health Data Exchange website.
Results and Discussion		
15	Provide published estimates in a file format from which data can be efficiently extracted.	Summary; Main text: Results, table 1; Appendix: tables S4-S8.<br>Published estimates not available in main text, appendix, or Global Health Data Exchange website will be made available upon request.
16	Report a quantitative measure of the uncertainty of the estimates (e.g. uncertainty intervals).	95% uncertainty intervals (UIs) are given for all mean estimates in the main text: Summary, Results, Table 1, Figure 1, Figure 3; where 95% UIs are not present in Figure 2 and Figure 4 they are supplemented by tables with the mean estimates and uncertainty intervals (Table 1, table S7).
17	Interpret results in light of existing evidence. If updating a previous set of estimates, describe the reasons for changes in estimates.	Main text: Methods, Discussion; Appendix: Supplementary methods
18	Discuss limitations of the estimates. Include a discussion of any modelling assumptions or data limitations that affect interpretation of the estimates.	Main text: Discussion

Section 2 Supplemental methods

Section 2.1 Global Burden of Disease cause hierarchy

The GBD cause hierarchy is designed to be mutually exclusive and collectively exhaustive, and injuries make up one of its three main Level 1 categories – non-communicable diseases; communicable, maternal, neonatal, and nutritional diseases; and injuries. At Level 2, there is a category for unintentional injuries, which includes causes of injury at Level 3 such as poisonings, falls, and drowning. Poisoning by carbon monoxide and poisoning by other means are Level 4 causes.

Section 2.2 Global Burden of Disease location hierarchy

The below table displays the GBD location hierarchy, and is organised by the seven super-regions (headers), their corresponding regions (left column), and each region's corresponding countries (right column). Countries where subnational estimates were analysed are noted. The location hierarchy was constructed by grouping together countries with geographical proximity and shared epidemiological characteristics (e.g., under-5 mortality, income).[5]

**Central Europe, eastern Europe, and central Asia**	
Central Asia	Armenia, Azerbaijan, Georgia, Kazakhstan, Kyrgyzstan, Mongolia, Tajikistan, Turkmenistan, Uzbekistan
Central Europe	Albania, Bosnia and Herzegovina, Bulgaria, Croatia, Czech Republic, Hungary, Montenegro, North Macedonia, Poland (subnational), Romania, Serbia, Slovakia, Slovenia
Eastern Europe	Belarus, Estonia, Latvia, Lithuania, Moldova, Russia (subnational), Ukraine
**High-income**	
Australasia	Australia, New Zealand (subnational Maori + non-Maori)
High-income Asia Pacific	Brunei, Japan (subnational), Singapore, South Korea
High-income North America	Canada, Greenland, USA (subnational)
Southern Latin America	Argentina, Chile, Uruguay
Western Europe	Andorra, Austria, Belgium, Cyprus, Denmark, Finland, France, Germany, Greece, Iceland, Ireland, Israel, Italy (subnational), Luxembourg, Malta, Monaco, Netherlands, Norway (subnational), Portugal, San Marino, Spain, Sweden (subnational), Switzerland, UK (subnational)
**Latin America and Caribbean**	
Andean Latin America	Bolivia, Ecuador, Peru
Caribbean	Antigua and Barbuda, Bahamas, Barbados, Belize, Bermuda, Cuba, Dominica, Dominican Republic, Grenada, Guyana, Haiti, Jamaica, Puerto Rico, Saint Kitts and Nevis, Saint Lucia, Saint Vincent and the Grenadines, Suriname, Trinidad and Tobago, Virgin Islands
Central Latin America	Colombia, Costa Rica, El Salvador, Guatemala, Honduras, Mexico (subnational), Nicaragua, Panama, Venezuela
Tropical Latin America	Brazil (subnational), Paraguay
**North Africa and Middle East**	

North Africa and Middle East	Afghanistan, Algeria, Bahrain, Egypt, Iran (subnational), Iraq, Jordan, Kuwait, Lebanon, Libya, Morocco, Oman, Palestine, Qatar, Saudi Arabia, Sudan, Syria, Tunisia, Türkiye, United Arab Emirates, Yemen
**South Asia**	
South Asia	Bangladesh, Bhutan, India (subnational), Nepal, Pakistan (subnational)
**Southeast Asia, east Asia, Oceania**	
East Asia	China, North Korea, Taiwan (province of China)
Oceania	American Samoa, Cook Islands, Federated States of Micronesia, Fiji, Guam, Kiribati, Marshall Islands, Nauru, Niue, Northern Mariana Islands, Palau, Papua New Guinea, Samoa, Solomon Islands, Tokelau, Tonga, Tuvalu, Vanuatu
Southeast Asia	Cambodia, Indonesia (subnational), Laos, Malaysia, Maldives, Mauritius, Myanmar, Philippines (subnational), Seychelles, Sri Lanka, Thailand, Timor-Leste, Viet Nam
**Sub-Saharan Africa**	
Central sub-Saharan Africa	Angola, Central African Republic, Congo (Brazzaville), Democratic Republic of the Congo, Equatorial Guinea, Gabon
Eastern sub-Saharan Africa	Burundi, Comoros, Djibouti, Eritrea, Ethiopia (subnational), Kenya (subnational), Madagascar, Malawi, Mozambique, Rwanda, Somalia, South Sudan, Tanzania, Uganda, Zambia
Southern sub-Saharan Africa	Botswana, Eswatini, Lesotho, Namibia, South Africa (subnational), Zimbabwe
Western sub-Saharan Africa	Benin, Burkina Faso, Cabo Verde, Cameroon, Chad, Côte d'Ivoire, the Gambia, Ghana, Guinea, Guinea-Bissau, Liberia, Mali, Mauritania, Niger, Nigeria (subnational), São Tomé and Príncipe, Senegal, Sierra Leone, Togo

Section 2.3 Cause of death data identification[1]

All available data on causes of death (CoD) data are standardised and pooled into a single database used to generate cause-specific mortality estimates by age, sex, year, and geography. The CoD database contains seven types of data sources: vital registration (VR), verbal autopsy (VA), cancer registry, police records, sibling history, surveillance, survey/census, and minimally invasive tissue sample (MITS) diagnoses. In countries with VR systems with high completeness and low garbage coding, vital registration is often the primary source of data for causes of death. Less than half the world's population has deaths captured in a VR system; therefore, for countries with incomplete VR systems, vital statistics for causes of death may be supplemented with other data types.

A majority of the CoD data is VR data obtained from the World Health Organization (WHO) Mortality Database, a compilation of data submitted to WHO by individual countries. VR is also obtained from country-specific mortality databases operated by official offices. Each cause is coded directly to the 3- or 4-digit ICD-coded Cause of Death when possible, whereas cause codes in data tabulated by International Classification of Disease (ICD-) are coded to aggregated cause groups. Detailed causes are coded to one of the following ICD-detail coding systems: ICD-8, ICD-9, or ICD-10 (table S5). Each coding system has a similar cause hierarchy and cause list that has continually developed over time.

ICD-10 is the current standard and the most exhaustive cause list.

In countries without VR systems, VA studies are a viable data source to inform CoD. Data are obtained by trained interviewers who use a standardised questionnaire to ask relatives about the signs, symptoms, and demographic characteristics of family members deceased within a year. CoD is assigned based on the answers to the questionnaires using a variety of methods. VA data are highly heterogeneous: studies use different instruments, different cause lists (from single causes to full ICD cause lists), different methods for assigning CoD, different recall periods, and different age groups. Cultural differences may also affect the interpretation of specific questions. VAs are likely accurate in assigning CoD to road injury or homicide but less accurate for causes requiring medical certification, such as cardiovascular causes. Studies may also occur as stand-alone assessments or as part of an extended network. Each GBD round, a systematic review of verbal autopsy literature data is conducted to ascertain and include new VA studies.

Section 2.4 Garbage code identification[1]

A crucial aspect of enhancing the comparability of data for causes of death is to deal with uninformative, so-called garbage codes. Garbage codes are codes to which deaths were assigned that cannot or should not be considered as the underlying cause of death, for example, heart failure, ill-defined cancer site, senility, ill-defined external causes of injuries, and sepsis. Additionally, any code not specific enough to assign to a detailed cause of death is considered a garbage code, such as unspecified infectious disease or unspecified injury. In GBD 2019, we developed additional maps to translate ICD codes found in the input data that are non-underlying causes to appropriate target codes based on the levels of the GBD cause list. These garbage codes were mapped to Class 1–4 of the GBD cause list according to the following criteria:

1. **Class 1** includes all garbage codes for which a Level 1 GBD cause (communicable, maternal, neonatal, and nutritional diseases; injuries; and non-communicable diseases) cannot be directly assigned. For example, the underlying causes of “sepsis” or “peritonitis”, if not specified in the data, could be an injury, a non-communicable disease, or a type of communicable disease. In these cases, deaths will be redistributed across all three of these Level 1 causes. In addition, deaths coded to impossible or ill-defined causes of death (including “senility” and “unspecified causes”) fall into this category, as they will be redistributed onto all causes.

2. **Class 2** includes all garbage codes that can be assigned to one Level 1 cause in the GBD cause list. This would include deaths coded to “unspecified injuries” (X59), which are redistributed onto all injuries.

3. **Class 3** includes all garbage codes for which we know the Level 2 cause of death and can redistribute onto one Level 3 cause. This includes deaths coded to causes such as “unspecified cardiovascular disease”, which falls within the Level 2 cause “cardiovascular diseases”, as well as those coded to “unspecified cancer site”, which falls within the Level 2 cause “neoplasms”.

4. **Class 4** includes all garbage codes for underlying causes of death that can be redistributed within a Level 4 cause. This includes garbage codes such as “unspecified stroke” or “unspecified road injuries”.

The methods for redistributing these garbage-coded deaths are outlined in detail in Johnson et al.[6], and the primary algorithm for redistributing deaths assigned to these codes has not changed since GBD 2013. However, key pieces of redistribution methodology are summarized below, including examples for carbon monoxide poisoning.

Section 2.5 Redistribution methodology[1]

For each redistribution package, we defined the “universe” of data as all deaths coded to either the package’s garbage codes or the package’s redistribution targets for each country, year, age, and sex. We then ran a regression based on the following equation separately for each target group and sex:

$$TG_{crt} = \alpha + \beta_1 Gar_{crt} + \beta_2 Age_{crt} Gar_{crt} + \theta_r Gar_{crt} + \gamma_r + \varepsilon_{ct}$$

Where:

TG_{crt} = percentage of deaths within the given garbage code’s universe that were coded to a given target group, by country

Gar_{crt} = percentage of deaths within the given garbage code’s universe that were coded to a given set of garbage codes

Age_{crt} = age interaction term for the fixed effect on the interaction of garbage and age

α = constant

β_1 = slope coefficient describing the association between Gar_{crt} and TG_{crt}

β_2 = slope coefficient describing the association between the interaction $Age_{crt}Gar_{crt}$ and G_{crt}

γ_r = region-specific random intercept (or super-region if the random effect on region is not significant)

θ_r = region-specific random slope (or super-region if the random effect on region is not significant)

ε_{ct} = standard error, normally distributed and calculated by bootstrapping

This regression was adjusted from GBD 2013 to include fixed effects on the interaction of garbage and age to ensure smooth age patterns. We made this decision after investigating diagnostic visualisations

that showed unlikely gaps between proportions assigned to different age groups.

Once proportions were produced for each country, sex, age, and target group, certain adjustments were made to conform our packages to the best medical evidence available. In some cases, we implemented restrictions on the proportions that the regressions could yield. For example, we did not allow any redistribution onto “Chagas disease” outside of Latin America and the Caribbean or “suicide” under the age of 15 years. In other cases, we capped the proportion for some targets to the level that would be produced from proportional redistribution; for example, “haemoglobinopathy” and “haemolytic anaemia” were restricted to the level of proportional redistribution in the redistribution of “left heart failure”. Occasionally, further adjustments were made on a case-by-case basis per country, age, sex, and target group to suppress the impact of outliers based on existing epidemiological evidence and expert judgment.

Section 2.5.1 Redistribution using multiple cause of death data[1]

Multiple CoD data are a form of individual record causes of death data that include an underlying CoD along with other causes in the death chain, including intermediate and immediate causes. By analysing this type of data, we can sometimes find the true underlying CoD in other CoD data where the underlying cause is a garbage code or a mis-assigned CoD.

As of GBD 2019, this method has been expanded and used in redistribution of the following intermediate causes: sepsis, embolism (pulmonary and arterial), heart failure (left, right, and unspecified), acute kidney injury, hepatic failure, acute respiratory failure, pneumonitis, and unspecified central nervous system disorders. Using multiple CoD records for the USA, Mexico, Brazil, Taiwan (province of China), Italy, Canada, New Zealand, Austria, South Africa, and Colombia we identified the fraction of deaths where the underlying cause of death and the intermediate cause was in the causal chain. Using a mixed effect linear regression, we estimated the fraction of intermediate-cause related deaths by underlying GBD cause. These fractions were multiplied by the GBD CoDCorrect result to calculate the number of deaths intermediate cause-related deaths for each GBD cause. Lastly, we calculated the “intermediate cause fraction”, with total intermediate-cause related deaths as the denominator, by age, sex, location, year GBD cause. These fractions were used to redistribute the intermediate-cause-related deaths to a GBD cause. An example is given below for sepsis where a, s, l, y, c denotes a given age group, sex, location, year, and underlying cause of death:

1. $sepsis\ fraction = \beta_{HAQ\ Index} + \beta_{age\ group} + \beta_{sex} + Y_{cause} + \varepsilon$

2. $sepsis\ deaths_{a,s,l,y,c} = sepsis\ fraction_{a,s,l,y,c} * GBD\ deaths_{a,s,l,y,c}$

3. $total\ sepsis\ deaths_{a,s,l,y} = \sum_c sepsis\ deaths_{a,s,l,y,c}$

4. $fraction\ of\ sepsis\ to\ redistribute_{a,s,l,y} = \frac{sepsis\ deaths_{a,s,l,y,c}}{total\ sepsis\ deaths_{a,s,l,y}}$

Section 2.5.2 Redistribution for external cause of injury garbage codes[1]

To redistribute X59 and Y34 (unspecified injuries) deaths, we used a multi-step approach that utilised the pattern of nature of injury codes in the causal chain in the multiple CoD data. First, we looked at deaths where X59, Y34, and GBD injuries causes were the underlying cause of death and got the pattern of nature of injury codes in the chain. We then derived a cause-specific redistribution proportion based on the probability of a given pattern being coded to X59/Y34 or a GBD injuries cause and summing up these proportions for all patterns. An example below is given for X59:

1. $$P_{(pattern_j|UCoD\ X59)} = \frac{\#\ of\ pattern_j\ deaths\ |\ UCoD\ X59}{\sum_{j=0}^{m}(\#\ of\ pattern_j\ deaths\ |UCoD\ X59)}$$

2. $$P_{(GBD\ injuries\ cause_i|pattern_j)} = \frac{\#\ of\ UCoD\ GBD\ injuries\ cause_i\ deaths\ |\ pattern_j}{\sum_{i=0}^{n}(\#\ of\ UCoD\ GBD\ injuries\ cause_i\ deaths\ |pattern_j)}$$

3. $$redistribution\ proportion_{GBD\ injuries\ cause_i} = \sum_{j=0}^{m}\left(P\left(pattern_j\middle|UCoDX59\right) * P(GBD\ injuries\ cause_i|pattern_j)\right)$$

Where:

$pattern_j$ = a given nature of injury code pattern in the chain of the multiple CoD data

UCoD X59 = a death with X59 coded as the underlying cause of death (UCoD)

UCoD GBD injuries $cause_i$ = a death with a GBD injuries causes coded as the UCoD

We applied these cause-specific redistribution proportions on the data where X59/Y34 were the underlying cause of death to get the number of X59/Y34 deaths "attributable" to each GBD injuries cause. Then, for each GBD injuries cause in the multiple CoD data, we calculated the fraction of redistributed X59/Y34 deaths over the fraction of total injuries death for that cause and modelled this intermediate cause fraction using a mixed effects linear regression. These fractions were then multiplied by GBD CoDCorrect results, and the cause fractions for X59 and Y34 were calculated by age, sex, location, year, and GBD injuries cause, and then used to redistribute X59 and Y34 deaths to GBD injuries causes.

Section 2.5.3 Redistribution examples for unintentional carbon monoxide poisoning

The following diagrams provide examples for select countries demonstrating the redistribution process, where garbage codes are reassigned to unintentional carbon monoxide poisoning. "Original" indicates the deaths that were originally coded to unintentional carbon monoxide poisoning as the underlying cause of death using the specified ICD codes, while all other segments of the figure represent the different types of garbage codes that were remapped to unintentional carbon monoxide poisoning through the redistribution process.

Example 1: Lithuania, both sexes and all ages, 2019

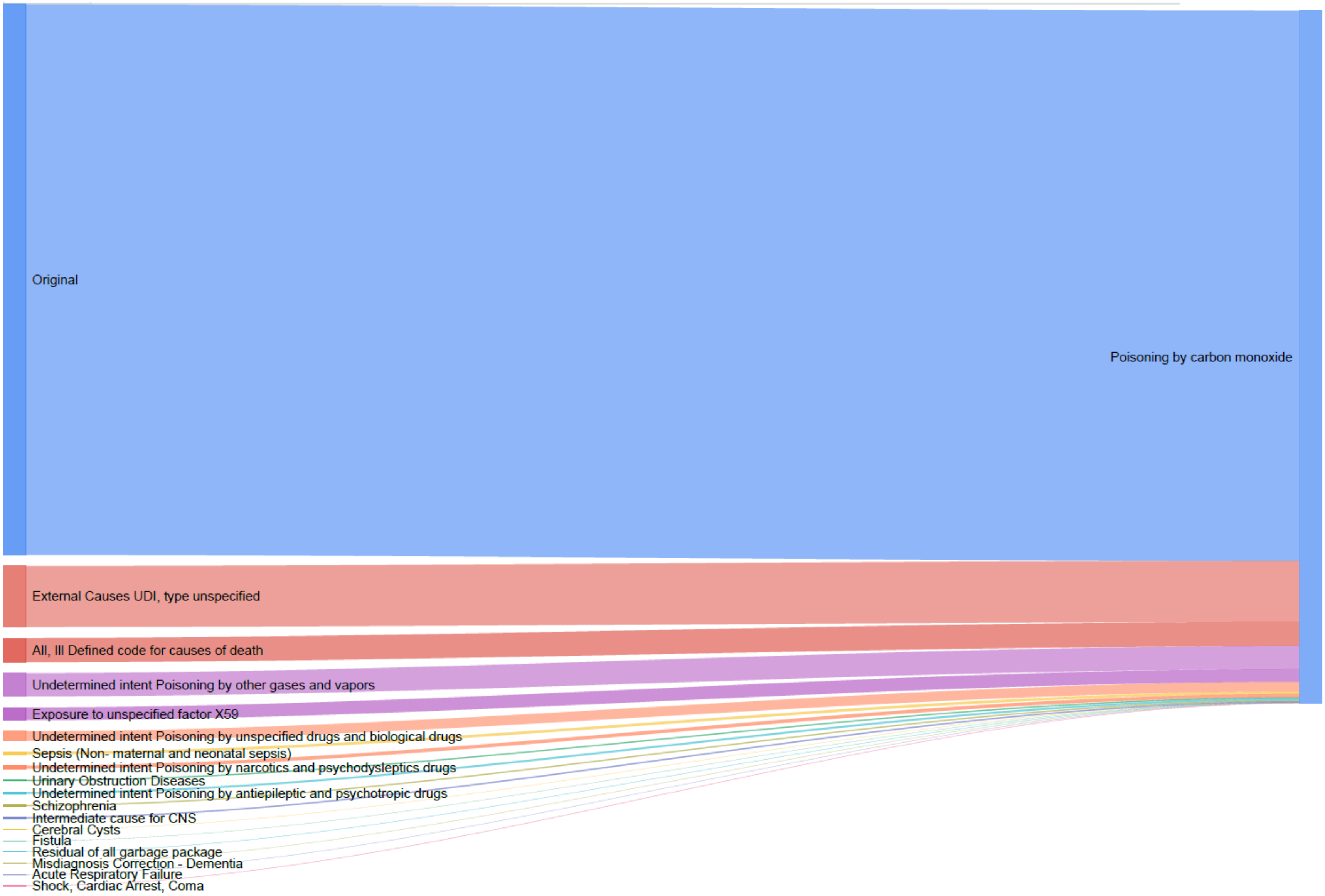

Example 2: South Korea, both sexes and all ages, 2017

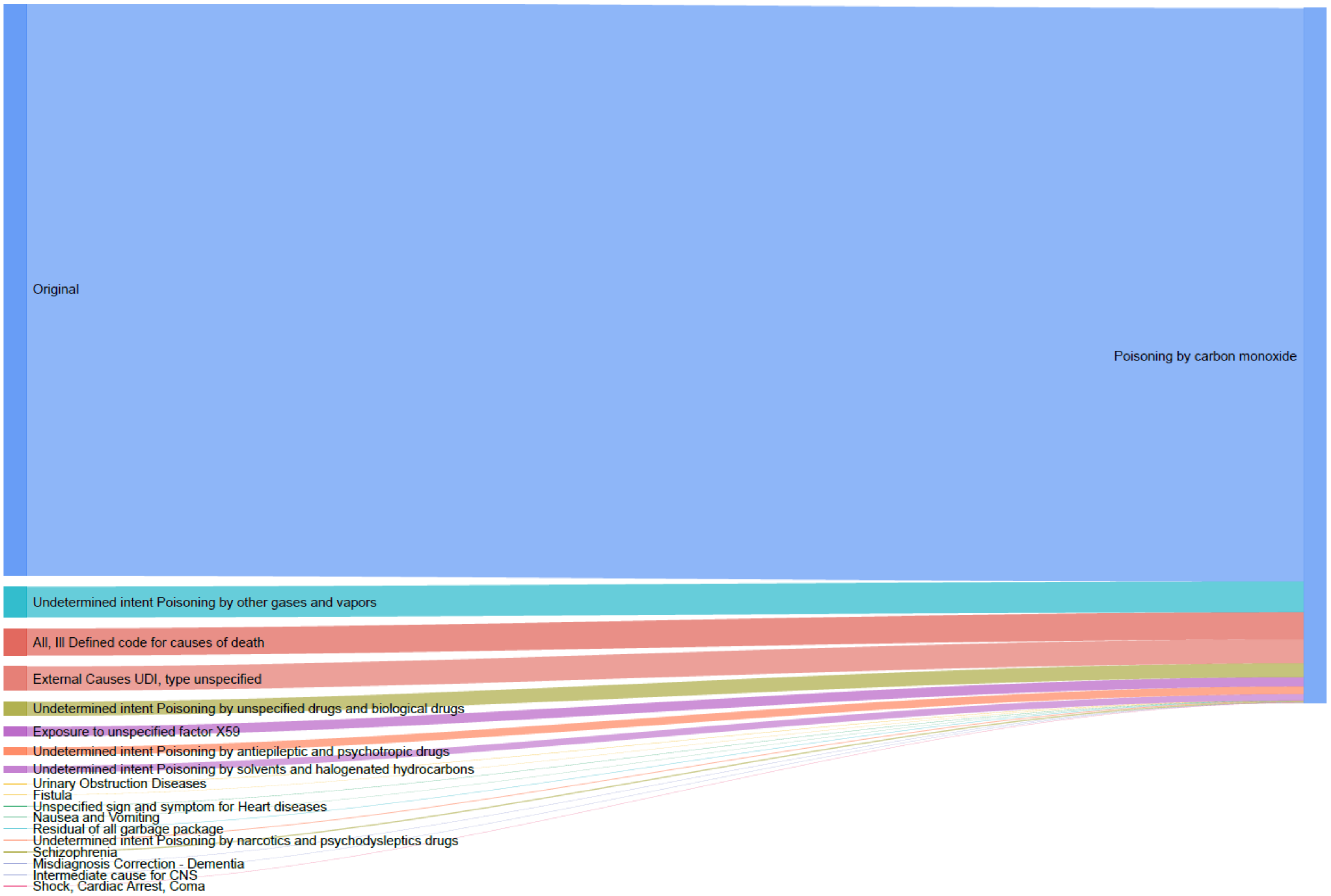

Example 3: United States of America, both sexes and all ages, 2019

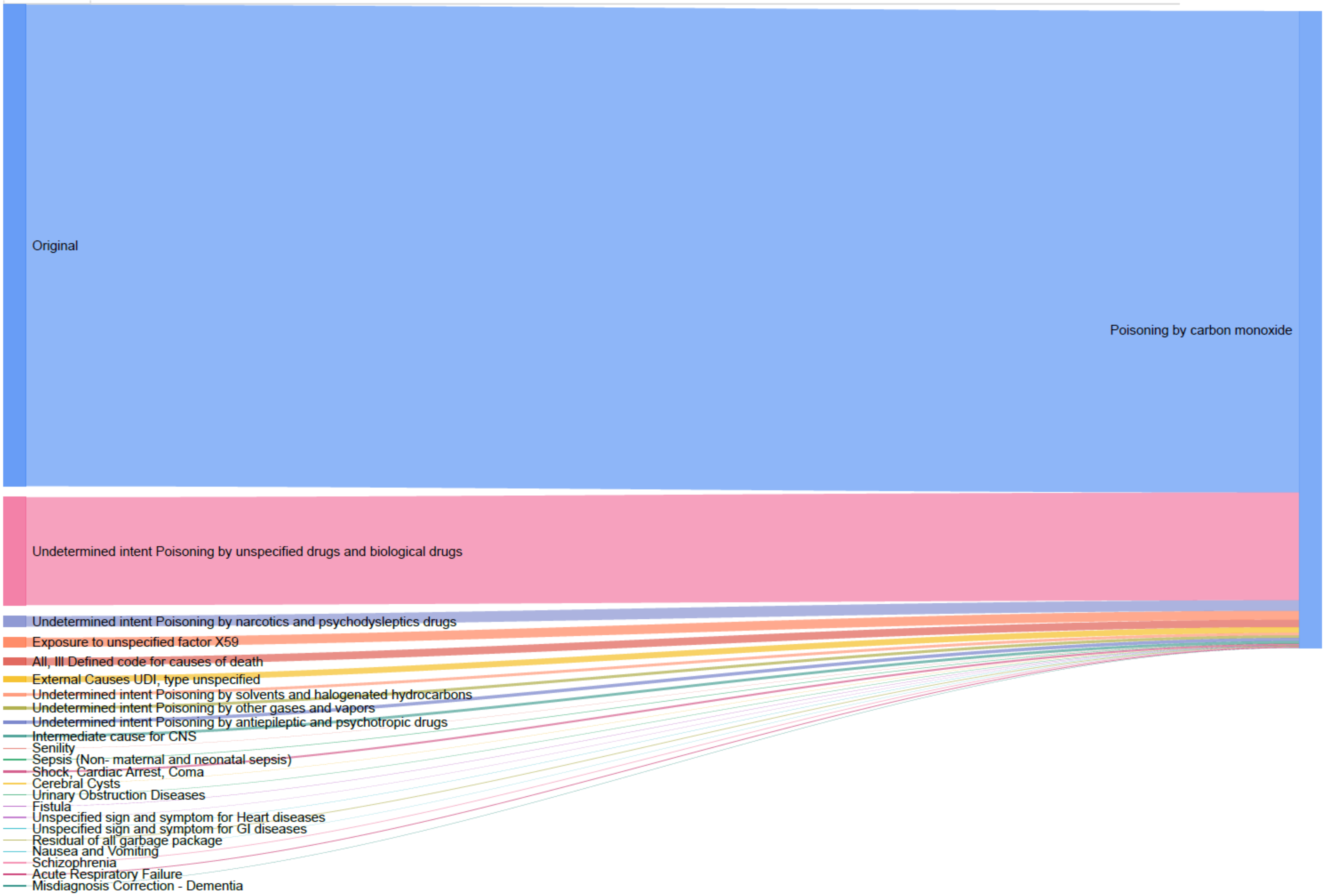

Example 4: Russia, both sexes and all ages, 2019

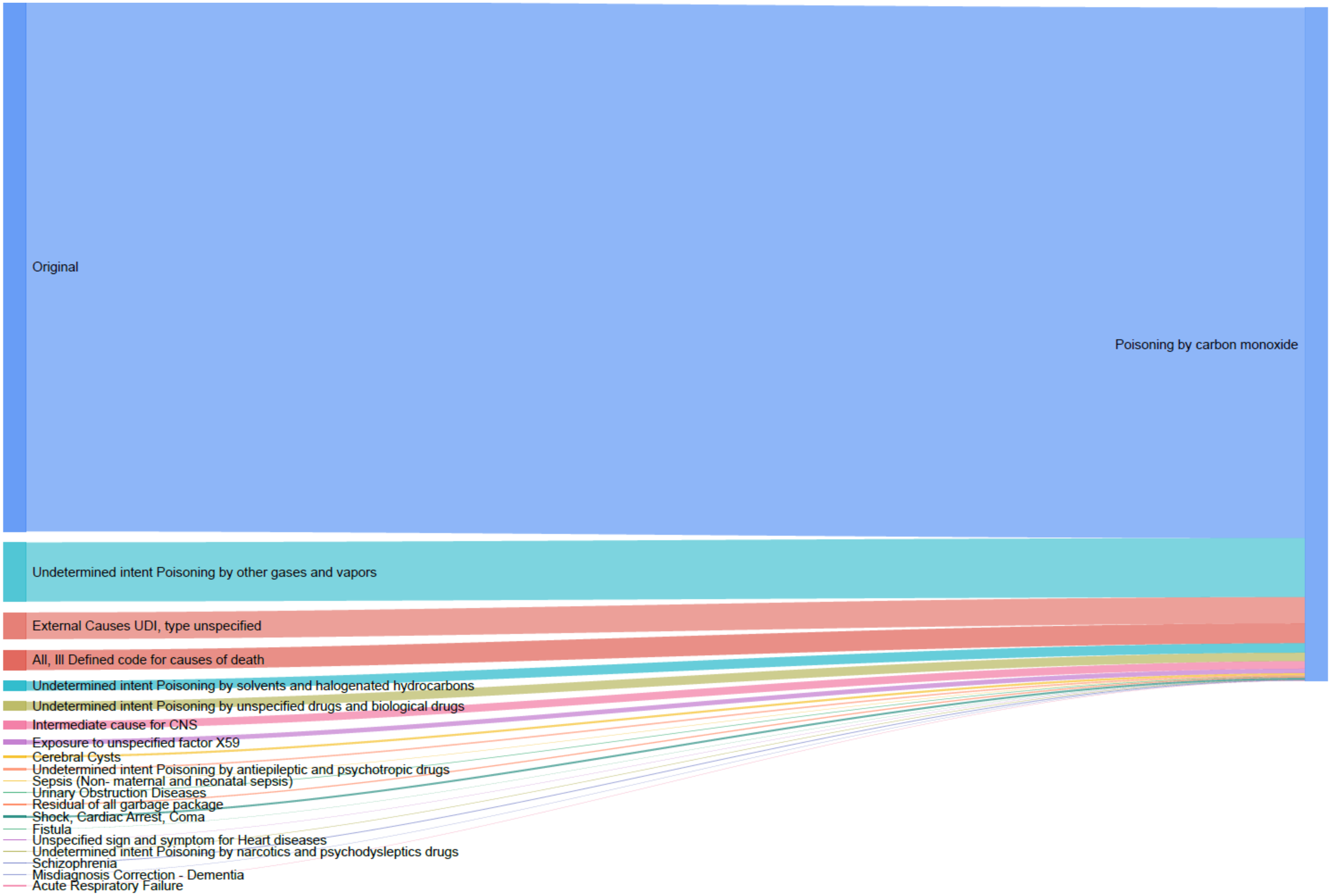

Section 2.6 Noise reduction[1]

To deal with problems of zero counts and stochastic variation in cause of death data, we use a Bayesian noise-reduction algorithm. We estimate a prior for a given series of data by running a Poisson regression to estimate the number of deaths due to each respective cause and sex with dummy variables for country, age, and year. With several notable exceptions, these regressions are sex-, cause-, and GBD region-specific, so borrowing strength over age, year, and country is only within a given data type, GBD region, cause, and sex.

This approach to noise reduction adjusts zero counts to non-zero values, mitigating the problem that zero counts in a log rate model or a logit cause fraction cause of death ensemble model (CODEm) must be dropped from the regression, leading to upward bias in the estimates. This is particularly important in three settings: small high-income countries with low death counts overall; rare causes with small numbers of cause-specific deaths; and the analysis of sibling history, verbal autopsy, and survey data with small samples.

We also employ several strategies to combat data sparsity and failure to converge in noise reduction models. First, for models run on GBD regions of VR data, if a given year has only one country of data in that region, we bin this year with the preceding year in the Poisson regression. Second, for VA data, if a given cause/super-region/sex group has six or fewer observations or zero non-zero observations, we include all VA globally available for that cause/sex in a regression with fixed effects on age group and study-location, and use the predictions from this global model as the prior for that cause/super-region/sex group. Third, for VA and CHAMPS data, if a model that includes random effects fails to converge, we rerun the model with the corresponding fixed effects. Fourth, after this, for all noise reduction models, if the model fails to converge, we use a weighted average of the observed cause fractions within age groups in the model input data as the prior in noise reduction. This is essentially pooling information across study, location, and year within a grouping of data with similar data type and geography.

During noise reduction, all datapoints with cause fractions of zero are raised to a non-zero value through the Bayesian average with a non-zero prior. However, some of these values can be extremely small in magnitude (eg, on the order of 10^{-50} or smaller), to the point that they would be unobservable in the raw data. Cause fractions of this magnitude would become extremely large in absolute value when log- or logit-transformed during the Cause of Death Ensemble modelling (CODEm) process, leading to poor model fit in other ranges of the input data. However, these datapoints must remain non-zero in order to include them in a log- or logit-transformed model at all and avoid upwardly biasing estimates. To address this problem, we enforce a set of non-zero cause-, age-, and sex-specific minimums, known as the “non-zero floor,” on all cause fractions in the cause of death database. These floor values are chosen to be high enough to be relatively close to the bulk of the remaining data in log- or logit-space, but low enough to avoid significantly upwardly biasing the distribution of the data.

Floor values are determined based on VR data for cause/age/sex groups where we have enough data

to do so accurately. We first consider VR data from all countries with greater than 50 million person-years represented in the database, after cause mapping and age-sex splitting but before any further processing. We calculate all-year cause fractions for each country, cause, age, and sex in this dataset. If the minimum cause fraction across country for a given cause/age/sex is non-zero, then we consider this cause/age/sex group to have sufficient data to determine a data-driven floor value. Otherwise, we consider this cause/age/sex to be too rare to have sufficient data to determine a minimum observable non-zero rate.

For cause/age/sex groups that have sufficient data, we set floor values based on all national-level noise-reduced VR datapoints in the CoD database that are non-zero before noise reduction. For all other cause/age/sex groups, which have insufficient data to inform the above calculation, we set a floor value such that (a) all floor values for a given cause add up to one death globally per year and (b) the age/sex distribution of the floor values in each cause follows the age/sex distribution of the global cause-specific mortality rates calculated for the purposes of age/sex splitting. This ensures that we add at most one death in the world per cause per year for causes where we have insufficient data to determine a plausible minimum observable cause fraction from the data.

We have assessed our data for overdispersion during the continued development of this algorithm. The Poisson distribution describes the likelihood of observing a certain number of events within a fixed interval. In noise reduction, this is the number of deaths due to a given cause (the "event") within a set of deaths due to all causes (aka sample size, the "interval"). The Poisson distribution is most useful for modeling rare events that have low likelihoods. In noise reduction on causes of death, the rarest causes and demographics often have the most stochastic trends, and therefore rely on the prior estimate from the Poisson regression most heavily. The Poisson distribution assumes that the distribution of deaths looks like this:

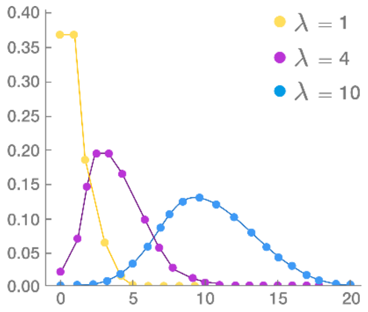

Which you can see in our example below of Iceland, it does (showing distribution of deaths, pre-noise reduction):

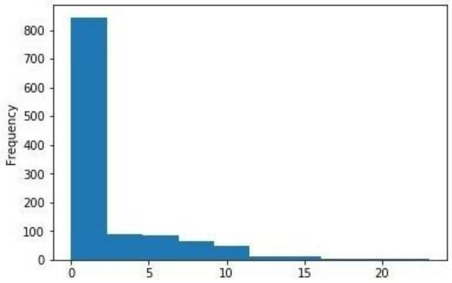

Second, each noise reduction model is run on a group, which is a set of related data that we believe share important epidemiological trends and data collection biases. This ensures that incompatible datasets can't propagate their patterns to each other. Vital registration (VR) data are generally grouped by region. This ensures that highly stochastic data from small countries and rare causes can borrow strength from larger countries with smoother patterns. For subnationally estimated countries, the country is treated like the "region", and all subnationals for a given country are noise-reduced together.

Lastly, in addition to running separate regressions for every model group, we also run separate regressions for every cause and sex. In general, region-level model groups use age group, country, and year as their covariates. For country-level VR model groups for subnationals, we include subnational location as a covariate as well. We also calculated dispersion scalars for each individual NR model to identify most over-dispersed models and tested using quasipoisson, which ultimately we did not use due to adverse effects on the majority of the data, but we believe our model groupings adequately address over-dispersion.

Including a variable as a covariate allows the model prediction to vary across values of that variable; for example, if we didn't include age group as a covariate, then the predicted cause fraction would be the same for every age group. However, by including a variable as a covariate, we also assume that the pattern across that variable is the same for all of the data in the regression. This is why we must run separate regressions for every cause and sex.

Section 2.7 Cause of death data star rating system[1]

GBD estimates are most accurate when computed with a full time series of complete VR with a low percentage of garbage codes. For GBD 2016, we developed a simple star-rating system from 0 to 5 to give a picture of the quality of data available in a given country over the full time series used in GBD estimates. Countries improve in the star rating as they increase availability, completeness, and detail of their mortality data and reduce the percentage of deaths coded to ill-defined garbage codes or highly aggregated causes.

We assign star ratings to rate the quality of data for any given location year. Two dimensions determine this star rating: (I) the percentage of total deaths determined to be major garbage (such as ill-defined). Causes such as "injuries" or "cancer" will also be included in major garbage percentage because this

percentage includes use of highly aggregated causes; and (II) the level of completeness of death registration (percentage of total deaths captured by the death registration). These two values were used to create a “percent well-certified” value between 0 and 1, determined as:

$$pct_{wellcertified} = Completeness \times (1 - pct_{majgarbage})$$

The mapping of percent well certified to star rating is as followed:

0 star: $0\% = pct_{wellcertified}$

1 star: $0\% < pct_{wellcertified} < 10\%$

2 star: $10\% <= pct_{wellcertified} < 35\%$

3 star: $35\% <= pct_{wellcertified} < 65\%$

4 star: $65\% <= pct_{wellcertified} < 85\%$

5 star: $pct_{wellcertified} \geq 85\%$

Star ratings are calculated for individual years of data as well as the full time series by location, and among other purposes, are used to help determine which countries fall into the “data-rich” set of locations for CODEm. Locations deemed as “data-rich” are those with 4- or 5-star ratings for the full time series. Included below are the national locations considered to be data-rich.

Data-rich countries: Antigua and Barbuda, Argentina, Armenia, Australia, Austria, Barbados, Belarus, Belgium, Belize, Bermuda, Brazil, Bulgaria, Canada, Chile, Colombia, Costa Rica, Croatia, Cuba, Czechia, Denmark, Ecuador, Estonia, Finland, France, Georgia, Germany, Greece, Grenada, Guatemala, Guyana, Hungary, Iceland, Ireland, Israel, Italy, Jamaica, Japan, Kazakhstan, Kuwait, Kyrgyzstan, Latvia, Lithuania, Luxembourg, Malta, Mauritius, Mexico, Moldova, Netherlands, New Zealand, Norway, Panama, Poland, Portugal, Puerto Rico, Romania, Russia, Saint Kitts and Nevis, Saint Lucia, Saint Vincent and the Grenadines, Singapore, Slovenia, Spain, Sweden, Switzerland, Taiwan (province of China), The Bahamas, Trinidad and Tobago, Turkmenistan, UK, Ukraine, Uruguay, USA, Uzbekistan, Venezuela

Section 2.8 Cause of death ensemble model (CODEm) overview[1]

Cause of death ensemble modelling (CODEm) is the framework used to model most cause-specific death rates in the GBD. It relies on four key components:

First, all available data are identified and gathered to be used in the modelling process. Although the data may vary in quality, they all contain some signal of the true epidemiological process.

Second, a diverse set of plausible models are developed to capture well-documented associations in the estimates. Using a wide variety of individual models to create an ensemble predictive model has been shown to outperform techniques using only a single model both in CoD estimation and in more general prediction applications.

Third, the out-of-sample predictive validity is assessed for all individual models, which are then ranked for use in the ensemble modelling stage.

Finally, differently weighted combinations of individual models are evaluated to select the ensemble model with the highest out-of-sample predictive validity.

Separate models are developed for countries with extensive, complete, and representative VR for every cause to ensure that uncertainty can better reflect the more complete data in these locations.

Because many factors may co-vary with any given CoD, a range of plausible statistical models are developed for each cause. In the CODEm framework, four families of statistical models are used: linear mixed effects regression (LMER) models of the natural log of the cause-specific death rate, LMER models of the logit of the cause fraction, spatiotemporal Gaussian process regression (ST-GPR) models of the natural logarithm of the cause-specific death rate, and ST-GPR models of the logit of the cause fraction (see the 2x2 table in Foreman et al., page 6).[7] Both types of statistical models (LMER and ST-GPR) draw upon information from nearby age groups, locations, or years; LMER models include random effects on age and location, while ST-GPR borrows across "space" (location), "time" (year), and age to adjust model predictions. In brief, as described by Foreman et al. on page 6, the choice for these model types was made to fulfill the following desired characteristics: flexibility in terms of dependent variable (mortality rate versus cause fraction), and relationships across location, age, and year (simple hierarchical model versus additional spatiotemporal patterns).[7] More details about the choice of these model types as well as their underlying formulas can be found on pages 7-9 in Foreman et al.

The families of models that go through ST-GPR incorporate information about data variance. The main inputs for a Gaussian process regression (GPR) are a mean function, a covariance function, and data variance for each data point. These inputs are described in detail in Foreman et al.[7] For GBD 2019, we have updated this calculation to incorporate garbage code redistribution uncertainty.[1]

Three components of data variance are now used in CODEm: sampling variance, non-sampling variance, and garbage code redistribution variance. The computation of sampling variance and non-sampling variance has not changed since previous iterations of the GBD and is also described in Foreman et al.[7] Since variance is additive, we calculate total data variance as the sum of sampling variance, non-sampling variance, and redistribution variance. Increased data variance in GPR results in the GPR draws not following the data point as closely.

Section 2.9 Covariates provided to CODEm[1]

In the CODEm models for carbon monoxide poisoning, covariates were included to inform model estimates in locations with data sparsity. This set of covariates was chosen based on prior knowledge of associations with mortality for the given cause of death, and the level and direction of covariates were set a priori. Level 1 covariates are generally those with the strongest evidence of association between

the covariate and cause of death (e.g., those with a strong biological link to the outcome), level 2 covariates are those with a strong evidence of relationship, but no proximal biological link, and level 3 covariates are those with weak or unknown association of a relationship with the outcome. For instance, since there is strong evidence for a relationship between temperature and carbon monoxide poisoning, but it is not a biological mechanism, the mean temperature covariate was set to level 2. To set a direction means to enforce a positive or negative correlation on the relationship between each covariate and mortality, for instance, a negative direction means that mortality is expected to decrease with an increase in the covariate. The set of covariates with their level and direction are provided in the below table. Figure S2 (appendix p 37) shows the covariates that were selected in the final ensemble models and their relative influence.

Covariates set at 'level 1' are prioritized when testing for association between covariates and the outcome using linear mixed effects models, such that first models with level 1 covariates are tested individually and then in combinations, followed by models with combinations of covariates from levels 1 and 2 based on the results of the first set of testing for association, followed by models with combinations of covariates from levels 1, 2, and 3, based on the results of the second round of testing for association. This approach tests for both significance of association and also maintains epidemiologically valid relationships defined by the literature between the predictors (covariates) and outcome (cause of death). During the covariate selection process, if there is a mismatch in the direction of a coefficient on a covariate between what was provided to the model and what was evaluated during model testing, the covariate is not selected.

**Covariate**	**Level**	**Direction**
Alcohol consumption (litres per capita)[8]	1	+
Summary exposure value (SEV), poisoning by carbon monoxide, log-transformed	1	+
Temperature (mean, population-weighted)[9–13]	2	-
Education (years per capita)[8]	3	-
Healthcare Access and Quality (HAQ) Index[8,14,15]	3	-
Lag-distributed income (LDI) per capita, log-transformed[11,16–18]	3	-
Socio-demographic index (SDI)[18–20]	3	-

Section 2.9.1 Lag-distributed income[2]

Lag-distributed income (LDI) per capita is a moving average transformation of gross domestic product (GDP) per capita.[21] Information from four data sources on GDP per capita was compiled to create a unified GDP per capita series for GBD. LDI was then calculated as a ten-year lagged average of GDP.

Section 2.9.2 Socio-demographic index[2]

The Socio-demographic Index (SDI) is a composite indicator created to get at the background social and economic conditions that shape health outcomes. SDI includes three components: an economic indicator, lag-distributed income (LDI) per capita, and two demographic indicators, total fertility rate under the age of 25 mean educational attainment for those aged 15 and older. To create the index, each component was rescaled to obtain a value between 0.005 and 1. SDI was calculated by taking the geometric mean of those rescaled values, for every location-year estimated in GBD.

Section 2.9.3 Healthcare Access and Quality Index

The Healthcare Access and Quality Index (HAQ Index) is a summary measure designed to represent the accessibility and quality of health care available to persons in a given location. HAQ Index values range from 0 to 100 and were estimated based on risk-standardised mortality rates for amenable causes of death – those that should not result in death when high-quality health care is available.[22]

Section 2.10 CODEm model pool development, testing, and ensemble creation[1]

The performance of all models (individual and ensemble) is evaluated by means of out-of-sample predictive validity tests. Thirty percent of the data are randomly excluded from the initial model fits. These individual model fits are evaluated and ranked by using half of the excluded data (15% of the total), then used to construct the ensembles on the basis of their performance. Data are held out from the analysis on the basis of the cause-specific missingness patterns for ages and years across locations. Out-of-sample predictive validity testing is repeated 20 times for each model, which has been shown to produce stable results. These performance tests include the root mean square error (RMSE) for the log of the cause-specific death rate, the direction of the predicted versus actual trend in the data, and the coverage of the predicted 95% UI.

The component models are weighted on the basis of their predictive validity rank to determine their contribution to the ensemble estimate. The relative weights are determined both by the model ranks and by a parameter ψ, whose value determines how quickly the weights taper off as rank decreases. The distribution of ψ is described in more detail in Foreman et al.[7] A set of ensemble models is then created by using the weights constructed from the combinations of ranks and ψ values. These ensembles are tested by using the predictive validity metrics on the remaining 15% of the data, and the ensemble with the best performance in out-of-sample trend and RMSE is chosen as the final model.

Once a weighting scheme has been chosen, 1000 draws are created for the final ensemble, and the number of draws contributed by each model is proportional to its weight. The mean of the draws is used as the final estimate for the CODEm process, and a 95% UI is created from the 0.025 and 0.975 quantiles of the draws. The validity of the UI can be checked via its coverage of the out-of-sample data; ideally, the 95% UI would capture 95% of these data. Higher coverage suggests that the UIs are too large, and lower coverage suggests overfitting.

The uncertainty intervals associated with final cause of death estimates take into account any uncertainty in the underlying data, including variance due to sampling error and non-sampling error, as well as any uncertainty associated with the redistribution and noise reduction processes.[1,7]

Section 2.11 CODEm model fit statistics[1]

The performance of all models (individual and ensemble) is evaluated by means of out-of-sample predictive validity tests. Data are held out from the analysis on the basis of the cause-specific

missingness patterns for ages and years across locations. These performance tests include the root mean square error (RMSE) for the log of the cause-specific death rate, the direction of the predicted versus actual trend in the data, and the coverage of the predicted 95% UI. The ensemble with the best performance in out-of-sample trend and RMSE is chosen as the final model (shown below).

Separate "data-rich" models are developed for countries with extensive, complete, and representative VR for every cause to ensure that uncertainty can better reflect the more complete data in these locations; "global" models include all data for all locations (see appendix p 19 for list of locations).

		RMSE in-sample	RMSE out-of-sample	Trend in-sample	Trend out-of-sample	Coverage in-sample	Coverage out-of-sample
**Global**	**Female**	0.413346	0.719882	0.265882	0.263329	0.998709	0.992113
**Data-rich**	**Female**	0.257792	0.385033	0.21149	0.271939	0.998487	0.996339
**Global**	**Male**	0.377433	0.63944	0.223457	0.226754	0.998347	0.992009
**Data-rich**	**Male**	0.213626	0.301313	0.174932	0.206034	0.999342	0.99834

Section 2.12 GBD risk factor estimation overview[3]

At a high level, the comparative risk assessment framework used in the GBD uses the following steps. First, risk-outcome pairs are identified and assessed for inclusion. Importantly, from page 1225 of Murray et al., inclusion requires "convincing evidence" based on the following criteria: "more than one study type, at least two cohorts, no substantial unexplained heterogeneity across studies, good-quality studies to exclude the risk of confounding and selection bias, and biologically-plausible dose-response gradients."[3] Second, for each risk-outcome pair, the relative risk is estimated as a function of exposure. Third, the distribution of each exposure is estimated, for every age-sex-location-year included in the GBD. Fourth, the theoretical minimum risk exposure level (TMREL) is identified for each risk factor. Fifth, population attributable fractions (PAFs) are estimated for each risk factor, and for combinations of risk factors, where applicable. Sixth, summary exposure values (SEVs) are estimated to provide a comprehensive summary estimate of risk exposure at the population level. Finally, risk-deleted death rates are calculated, estimating the death rate that would result from all risk factors being set to their TMRELs. A more detailed overview of the GBD risk factor framework is available in Murray et al. (main text pages 1225-1228), including a technical walkthrough of all underlying methods and associated model formulas in the accompanying appendix 1, pages 16-49.[3]

While all risk factors included in the GBD broadly follow these steps, each risk factor has its own specific methodology. Detailed descriptions of the methods used for the two risk factors in this study, occupational injuries and high alcohol use, are included below.

Section 2.13 Occupational injuries risk factor[3]

Section 2.13.1 Occupational injuries risk factor definition and input data[3]

The occupational injuries risk factor exposure is defined as the proportion of injuries in the working-age population attributable to occupation, based on fatal injury rates in 17 economic activities.

Economic activities and occupations were coded according to the following categories:

**Economic activities**
Agriculture, hunting, forestry
Fishing
Mining and quarrying
Manufacturing
Electricity, gas, and water
Construction
Wholesale and retail trade/repair
Hospitality
Transport, storage, and communication
Financial intermediation
Real estate/renting
Public administration/defense; compulsory social security
Education
Health and social work
Other community/social/personal service activities
Private households
Extra-territorial organisations/bodies

**Occupational categories**
Legislators, senior officials, and managers
Professionals
Technicians and associate professionals
Clerks
Service workers and shop/market sales workers
Skilled agricultural and fishery workers
Plant and machine operators and assemblers
Craft and related workers
Elementary occupations

Primary inputs were obtained from the International Labour Organization (ILO). These inputs included raw data on economic activity proportions, occupation proportions, fatal injury rates, and employment to population ratio estimates. No data on informal employment was included due to data sparseness. In 2017, a systematic review was conducted to collect the underlying microdata from the ILO's estimates to aid in re-extraction at greater levels of granularity. Where freely available, survey datasets were

downloaded from the survey organisations in question. Other datasets were obtained through submission of requests to agencies and through the GBD collaborator network. Microdata were tabulated in order to create survey-weighted estimates of economic activities and occupations for the GBD geographies and years. Various classification systems were adjusted to match the ISIC Rev.3 classification (for economic activities) and ISCO 1988 classification (for occupations). For the current GBD cycle, we updated our ILO data by downloading the most recent data files from their website.

Section 2.13.2 Occupational injuries modelling strategy[3]

Population-level occupational injury counts were estimated for each age (from years 15–84), sex, location, year by multiplying fatal injury rates for each of the 17 activities by the proportion of the population engaged in each activity, as well as an aggregate count, using spatiotemporal Gaussian process regression (ST-GPR).

Occupational injury counts were estimated using the following equation:

$$Occupational\ fatal\ injuries_{c,y,a,s} = \sum_{EA} Injury\ rate_{EA,c,y,s} * Population_{c,y,a,s} * EAP_{c,y,s,a} * Proportion_{EA,c,y}$$

where:

EAP = economically active population	c = country	y = year
EA = economic activity	a = age	s = sex

In the latest GBD study, we used meta-regression—Bayesian, regularised, trimmed (MR-BRT)[3,23] to crosswalk our data. Occupational injuries exposure data come from a number of different sources: insurance records, labour inspectorate records, establishment surveys, establishment or business registers, labour force surveys, economic or establishment censuses, official estimates, records of employers' organizations, and other administrative records and related sources. We expect insurance records to be the gold-standard source, because people should have more incentive to report injuries when they stand to benefit from their insurance plans. As such, we wanted to correct the data reported from other sources for under-reporting, and so we crosswalked all of the data with insurance records data as our reference. To do so, we ran a mixed-effects log-linear regression using MR-BRT, with fixed effects on type of data source and random effects on super-region and region.

<u>Theoretical minimum-risk exposure level (TMREL)</u>

For all occupational risks, the theoretical minimum-risk exposure level was assumed to be no exposure to that risk.

<u>Population attributable fraction (PAF)</u>

The PAFs for occupational injuries were calculated using the following formula:

$$PAF_{c,y,a,s} = \frac{Occupational\ fatal\ injuries_{c,y,a,s} - TMREL}{Fatal\ injuries_{c,y,a,s}}$$

where:

c = country	a = age

y = year s = sex

Since the TMREL is zero, the occupational injuries PAF is simply the ratio of occupational fatal injuries to total fatal injuries. Fatal injury totals were obtained from the latest GBD causes of death estimates.

Section 2.14 High alcohol use risk factor[3]

Section 2.14.1 High alcohol use risk factor definition and input data[3]

High alcohol use is defined as alcohol consumption in excess of the theoretical minimum risk exposure level (TMREL), the level of alcohol consumption at which all-cause risk is minimised. In previous iteration of GBD, this risk factor was simply "Alcohol use" and quantified the burden of alcohol consumption over the entire exposure range.

We defined exposure as the grams per day of pure alcohol consumed among current drinkers. We constructed this exposure using the indicators outlined below:

1. Current drinkers, defined as the proportion of individuals who have consumed at least one alcoholic beverage (or some approximation) in a 12-month period.
2. Alcohol consumption (in grams per day), defined as grams of alcohol consumed by current drinkers, per day, over a 12-month period.
3. Alcohol litres per capita (LPC) stock, defined in LBC of pure alcohol, over a 12-month period.

We also used three additional indicators to adjust alcohol exposure estimates to account for different types of bias:

1. Number of tourists within a location, defined as the total amount of visitors to a location within a 12-month period.
2. Tourists' duration of stay, defined as the number of days resided in a hosting country.
3. Unrecorded alcohol stock, defined as a percentage of the total alcohol stock produced outside established markets.

A systematic review of the literature was performed to extract data on our primary indicators. The Global Health Exchange (GHDx), IHME's online database of health-related data, was searched for population survey data containing participant-level information from which we could formulate the required alcohol use indicators on current drinkers and alcohol consumption. Data sources were included if they captured a sample representative of the geographical location under study.

For relative risks, in GBD 2016 we performed a systematic literature review of all cohort and case-control studies reporting a relative risk, hazard ratio, or odds ratio for any risk-outcome pairs studied in GBD 2016. Studies were included if they reported a categorical or continuous dose for alcohol consumption, as well as uncertainty measures for their outcomes, and the population under study was

representative.[24] Importantly, the risk-outcome pair that was assessed pertaining to carbon monoxide poisoning was alcohol use-unintentional injuries. Because data was unavailable at a more granular level (i.e., more specific causes of unintentional injury), the relative risk curves were estimated for alcohol use-unintentional injuries, and then applied to any sub-causes (e.g., carbon monoxide poisoning).

Estimates of current drinking prevalence were split by age and sex where necessary. First, studies that reported prevalence for both sexes were split using a region-specific sex ratio estimated using meta-regression—Bayesian, regularised, trimmed (MR-BRT).[3,23] Second, where studies reported estimates across non-GBD age groups, these were split into standard five-year age groups using the global age pattern estimated by ST-GPR.

To allow for the inclusion of data that did not meet our reference definition for current drinking, two crosswalks were performed using MR-BRT. The first crosswalk converted estimates of one-month drinking prevalence to what they would be if data represented estimates of 12-month drinking prevalence. This crosswalk incorporated two binary covariates: male and age ≥50. The second crosswalk converted estimates of one-week drinking prevalence to 12-month drinking prevalence. This crosswalk incorporated age <20 and male as covariates. The covariates utilised in both crosswalks were included as both x and z covariates. A uniform prior of 0 was set as the upper bound for the beta coefficients to enforce the logical constraint that one-month and one-week prevalence could not be greater than 12-month prevalence.

The raw data used in the supply-side model are domestic supply (WHO GISAH; FAO) and retail supply (Euromonitor) of litres of pure ethanol consumed. Domestic supply is calculated as the sum of production and imports, subtracting exports. The WHO and FAO sources were combined, so that FAO data were only used if there were no data available for that location-year from WHO. This was done because the WHO source takes into consideration FAO values when available. Since the WHO data are given in more granular alcohol types, the following adjustments were made:

$$LPC\ Pure\ Ethanol = 0.13 * \left(\frac{Wine}{0.973}\right)$$

$$LPC\ Pure\ Ethanol = 0.05 * \left(\frac{Beer}{0.989}\right)$$

$$LPC\ Pure\ Ethanol = 0.4 * \left(\frac{Spirits}{0.91}\right)$$

Three outliering strategies are used to omit implausible datapoints and data that created implausible model fluctuations. First, estimates from the current drinking model are used to calculate the grams of alcohol consumed per drinker per day. A point is outliered if the grams of pure ethanol per drinker per day for a given source-location-year is greater than 100 (approximately 10 drinks). These thresholds were chosen by using expert knowledge about reasonable consumption levels. In the second round of outliering, the mean LPC value over a 10-year window is calculated. If a point is over 70% of that mean value away from the mean value, it is outliered. The 70% limit was chosen using histograms of these distances. Additionally, some manual outliering is performed to account for edge cases. Finally, data smoothing is performed by taking a three-year rolling mean over each location-year.

Next, an imputation to fill in missing years is performed for all series to remove compositional bias from our final estimates. Since the data from our main sources cover different time periods, by imputing a complete time series for each data series, we reduce the probability that compositional bias of the sources is leading to biased final estimates. To impute the missing years for each series, we model the log ratio of each pair of sources as a function of an intercept and nested random effects on super-region, region, and location. The appropriate predicted ratio is multiplied by the source that we do have, which generates an estimated value for the missing source. For some locations where there was limited overlap between series, the predicted ratio did not make sense, and a regional ratio was used.

Finally, variance was calculated both across series (within a location-year) as well as across years (within a location-source). Additionally, if a location-year had one imputed point, the variance was multiplied by 2. If a location-year had two imputed points, the variance was multiplied by 4. The average estimates in each location-year were the input to an ST-GPR model. This uses a mixed-effects model modelled in log space with nested location random effects.

We obtained data on the number of tourists and their duration of stay from the UN World Tourism Organization. We applied a crosswalk across different tourist categories, similar to the one used for the LPC data, to arrive at a consistent definition (ie, visitors to a country).

We obtained estimates on unrecorded alcohol stock from data available in WHO GISAH database, consisting of 189 locations. For locations with no data available, the national or regional average was used.

Section 2.14.2 High alcohol use modelling strategy[3]

Exposure

While population-based surveys provide accurate estimates of the prevalence of current drinkers, they typically underestimate real alcohol consumption levels. As a result, we considered the LPC input to be a better estimate of overall volume of consumption. Per capita consumption, however, does not provide age- and sex-specific consumption estimates needed to compute alcohol-attributable burden of disease. Therefore, we use the age-sex pattern of consumption among drinkers modelled from the population survey data and the overall volume of consumption from FAO, GISAH, and Euromonitor to determine the total amount of alcohol consumed within a location. In the paragraphs that follow, we outline how we estimated each primary input in the alcohol exposure model, as well as how we combined these inputs to arrive at our final estimate of grams per day of pure alcohol. We estimated all models below using 1000 draws.

For data obtained through surveys, we used spatiotemporal Gaussian process regression (ST-GPR) to construct estimates for each location/year/age/sex. We chose to use ST-GPR due to its ability to leverage information across the nearby locations or time periods. We also modelled the alcohol LPC data, as well as the total number of tourists, using ST-GPR. To improve the LPC model fit in years beyond those in which data was available, we forecasted ST-GPR estimates using a damped holt function.

Given the heterogeneous nature of the estimates on unrecorded consumption, as well as the wide variation across countries and time periods, we took 1000 draws from the uniform distribution of the lowest and highest estimates available for a given country. We did this to incorporate the diffuse uncertainty within the unrecorded estimates reported. We used these 1000 draws in the equation below.

We adjusted the alcohol LPC for unrecorded consumption using the following equation:

$$Alcohol\ LPC = \frac{Alcohol\ LPC}{(1 - \%\ Unrecorded)}$$

We then adjusted the estimates for alcohol LPC for tourist consumption by adding in the per capita rate of consumption abroad and subtracting the per capita rate of tourist consumption domestically.

$$Alcohol\ LPC_d = Unadjusted\ Alcohol\ LPC_d + Alcohol\ LPC_{Domestic\ consumption\ abroad} - Alcohol\ LPC_{Tourist\ consumption\ domestically}$$

$$Alcohol\ LPC_i = \frac{\sum_l Tourist\ Population_l * Proportion\ of\ tourists_{i,l} * Unadjusted\ Alcohol\ LPC_l * \frac{Average\ length\ of\ stay_{i,l}}{365} *}{Population_d}$$

where:

l *is the set of all locations*, i *is either* Domestic consumption abroad *or* Tourist consumption domestically, *and* d *is a domestic location.*

After adjusting alcohol LPC by tourist consumption and unrecorded consumption for all location/years reported, sex-specific and age-specific estimates were generated by incorporating estimates modelled in ST-GPR for percentage of current drinkers within a location/year/sex/age, as well as consumption trends modelled in the ST-GPR grams per day model. We do this by first calculating the proportion of total consumption for a given location/year by age and sex, using the estimates of alcohol consumed per day, the population size, and the percentage of current drinkers. We then multiply this proportion of total stock for a given location/year/sex/age by the total stock for a given location/year to calculate the consumption in terms of LPC for a given location/year/sex/age. We then convert these estimates to be in terms of grams/per day. The following equations describe these calculations:

$$Proportion\ of\ total\ consumption_{l,y,s,a} = \frac{Alcohol\ \ g/day_{\ l,y,s,a} \ * \ Population_{\,l,y,s,a} \ * \ \%\ Current\ drinkers_{\,l,y,s,a}}{\sum_{s,a} Alcohol\ \ g/day_{\,l,y,s,a} \ * \ Population_{\,l,y,s,a} \ * \ \%\ Current\ drinkers_{\,l,y,s,a}}$$

$$Alcohol\ LPC_{\,l,y,s,a} = \frac{Alcohol\ LPC_{\,l,y} \ * \ Population_{\,l,y} \ * \ Proportion\ of\ total\ consumption_{\,l,y,s,a}}{\%\ Current\ drinkers_{\,l,y,s,a} * Population_{\,l,y,s,a}}$$

$$Alcohol\ g/day_{\,l,y,s,a} = Alcohol\ LPC_{\,l,y,s,a} * \frac{789\ g/L}{365}$$

where:

$$l\ is\ a\ location, y\ is\ a\ year, s\ is\ a\ sex, and\ a\ is\ an\ age\ group.$$

We then used the gamma distribution to estimate individual-level variation within location, year, sex, age drinking populations, following the recommendations of other published alcohol studies. We chose parameters of the gamma distribution based on the mean and standard deviation of the 1000 draws of alcohol g/day exposure for a given population. Standard deviation was calculated using the following formula. We tested several alternative models using our data and found this model performed best.

$$standard\ deviation = mean * (0.087 * female + \ 1.171\ )$$

Theoretical minimum risk exposure level (TMREL)

The methods for calculating the TMREL were updated for this iteration of GBD. Previously, one global estimate of the TMREL was calculated. However, the contributions of each cause to overall health loss vary over geography, age, time, and sex, suggesting that the amount of alcohol that minimises health loss similarly varies over these domains. For this reason, in this GBD study we estimated an individual TMREL for each region, age, sex, and year.

For each region, age, sex, and year, we calculated TMREL by first calculating the overall risk attributable to alcohol. We did this by weighting each relative risk curve by the share of overall DALYs for a given cause. We then took the minimum of this overall-risk curve as the TMREL of alcohol use. More formally,

$$TMREL = argmin\ average\ overall\ risk_{\omega}(g/day)$$

$$Average\ overall\ risk_{\omega,l,y,a,s}(g/day) = \sum_{i}^{\omega} \log\left(RR_i(g/day)\right) * \frac{DALY_{i,l,y,a,s}}{\sum_i^{\omega} DALY_{i,l,y,a,s}}$$

Where:

$\omega\ is\ the\ set\ of\ causes\ associated\ with\ alcohol, i\ is\ a\ given\ cause\ from\ that\ set,$

$l\ is\ a\ location,$ y $is\ a\ year, s\ is\ a\ sex, a\ is\ an\ age\ group, DALY\ is\ the\ DALY\ rate,$

$and\ RR\ is\ the\ dose\ response\ curve\ for\ a\ given\ cause\ and\ exposure\ level\ in\ grams\ per\ day.$

In other words, we chose TMREL as being the exposure that minimises the risk of suffering burden from any given cause related to alcohol. We weight the risk for a particular cause in our aggregation by the proportion of DALYs due to that cause (eg, since more observed people die from ischaemic heart disease, we weight the risk for ischaemic heart disease more in the above calculation of average risk compared to, say, diabetes, even if both have the same relative risk for a given level of consumption).

Relative risk

In this GBD study, for the injuries-related outcomes, we used the studies identified through a systematic review to calculate a dose–response, modelled using DisMod ODE.[3,24] We chose DisMod ODE rather than a conventional mixed-effects meta-regression because of its ability to estimate non-parametric splines over doses (ie, for most alcohol causes, there is a non-linear relationship with different doses) and incorporate heterogeneous doses through dose-integration (ie, most studies report doses categorically in wide ranges. DisMod ODE estimates specific doses when categories overlap across studies, through an integration step.). We used the results of the meta-regression to estimate a non-parametric curve for all doses between zero and 100 g/day and their corresponding relative risks. For all causes, we assumed the relative risk was the same for all ages and sexes.

Regarding injuries outcomes, we constructed relative risks based on chronic exposure to alcohol rather than acute exposure immediately preceding injury, which has a weaker relationship to the outcome, though still significant. We decided to use chronic exposure given the lack of available data on acute exposure, as well as the lack of cohort studies using acute exposure as a metric. Further, using chronic exposure allowed us to construct relative risks curves for unintentional injuries, interpersonal violence, motor vehicle accidents, and self-harm using the same method as reported above.

Population attributable fraction (PAF)

We calculated population attributable fractions (PAFs) by setting the relative risk of alcohol consumption among abstainers and drinkers consuming alcohol below the TMREL to be 1. We then

calculated PAFs for drinkers consuming alcohol in excess of the TMREL as we have previously. For each location, age, sex, year, and cause, we defined PAF as:

$$PAF(x) = \frac{P_A + \int_0^{TMREL} P(x)\ dx + \int_{TMREL}^{100} P(x) * RR_C(x)\ dx - RR_C(TMREL)}{P_A + \int_0^{TMREL} P(x)\ dx + \int_{TMREL}^{100} P(x) * RR_C(x)\ dx} \qquad P(x) = P_C * \Gamma(p)$$

where:

P_C *is the prevalence of current drinkers,* P_A *is the prevalence of abstainers, and*

p *are parameters determined by the mean and sd of exposure for that location, age, sex, and year;*

$RR_c(x)$ *is the global relative risk function for current drinkers for a given cause, and*

TMREL is the theoretical minimum risk exposure level for that location's region, age, sex, and year

We performed the above equation for 1000 draws of the exposure and relative risk models. We then used the estimated PAF draws to calculate YLL, YLDs, and DALYs, as per the other risk factors.[3]

Section 3 References

1 Vos T, Lim SS, Abbafati C, *et al.* Global burden of 369 diseases and injuries in 204 countries and territories, 1990–2019: a systematic analysis for the Global Burden of Disease Study 2019. *The Lancet* 2020; **396**: 1204–22.

2 Wang H, Abbas KM, Abbasifard M, *et al.* Global age-sex-specific fertility, mortality, healthy life expectancy (HALE), and population estimates in 204 countries and territories, 1950–2019: a comprehensive demographic analysis for the Global Burden of Disease Study 2019. *The Lancet* 2020; **396**: 1160–203.

3 Murray CJL, Aravkin AY, Zheng P, *et al.* Global burden of 87 risk factors in 204 countries and territories, 1990–2019: a systematic analysis for the Global Burden of Disease Study 2019. *The Lancet* 2020; **396**: 1223–49.

4 Stevens GA, Alkema L, Black RE, *et al.* Guidelines for Accurate and Transparent Health Estimates Reporting: the GATHER statement. *The Lancet* 2016; **388**: e19–23.

5 Murray CJ, Ezzati M, Flaxman AD, *et al.* GBD 2010: design, definitions, and metrics. *The Lancet* 2012; **380**: 2063–6.

6 Johnson SC, Cunningham M, Dippenaar IN, *et al.* Public health utility of cause of death data: applying empirical algorithms to improve data quality. *BMC Medical Informatics and Decision Making* 2021; **21**: 175.

7 Foreman KJ, Lozano R, Lopez AD, Murray CJ. Modeling causes of death: an integrated approach using CODEm. *Population Health Metrics* 2012; **10**: 1.

8 Sircar K, Clower J, Shin M kyong, Bailey C, King M, Yip F. Carbon monoxide poisoning deaths in the United States, 1999 to 2012. *The American Journal of Emergency Medicine* 2015; **33**: 1140–5.

9 Hampson NB. U.S. Mortality Due to Carbon Monoxide Poisoning, 1999–2014. Accidental and Intentional Deaths. *Annals ATS* 2016; **13**: 1768–74.

10 Hosseininejad SM, Aminiahidashti H, Goli Khatir I, Ghasempouri SK, Jabbari A, Khandashpour M. Carbon monoxide poisoning in Iran during 1999–2016: A systematic review and meta-analysis. *Journal of Forensic and Legal Medicine* 2018; **53**: 87–96.

11 Braubach M, Algoet A, Beaton M, Lauriou S, Héroux M-E, Krzyzanowski M. Mortality associated with exposure to carbon monoxide in WHO European Member States. *Indoor Air* 2013; **23**: 115–25.

12 Lavigne E, Weichenthal S, Wong J, Smith-Doiron M, Dugandzic R, Kosatsky T. Mortality and hospital admission rates for unintentional nonfire-related carbon monoxide poisoning across Canada: a trend analysis. *Canadian Medical Association Open Access Journal* 2015; **3**: E223–30.

13 Can G, Sayılı U, Aksu Sayman Ö, *et al.* Mapping of carbon monoxide related death risk in Turkey: a ten-year analysis based on news agency records. *BMC Public Health* 2019; **19**: 9.

14 Raub JA, Mathieu-Nolf M, Hampson NB, Thom SR. Carbon monoxide poisoning — a public health perspective. *Toxicology* 2000; **145**: 1–14.

15 Chiew AL, Buckley NA. Carbon monoxide poisoning in the 21st century. *Critical Care* 2014; **18**: 221.

16 King ME, Damon SA. Attitudes about Carbon Monoxide Safety in the United States: Results from the 2005 and 2006 HealthStyles Survey. *Public Health Rep* 2011; **126**: 100–7.

17 Runyan CW, Johnson RM, Yang J, *et al.* Risk and protective factors for fires, burns, and carbon monoxide poisoning in U.S. households. *American Journal of Preventive Medicine* 2005; **28**: 102–8.

18 Cui P, Jin Y, Feng H, Li Z, Ding S, Li Y. Burden of carbon monoxide poisoning in China, 1990–2019: A systematic analysis of data from the global burden of disease study 2019. *Frontiers in Public Health* 2022; **10**. https://www.frontiersin.org/articles/10.3389/fpubh.2022.930784 (accessed Nov 16, 2022).

19 Long J, Sun Y, Zhao J, Liu J, Peng X. Temporal trends of carbon monoxide poisoning mortality at the global, regional and national levels: a cross-sectional study from the Global Burden of Disease study, 1990 and 2017. *BMJ Open* 2021; **11**: e053240.

20 Liu F, Jiang X, Zhang M. Global burden analysis and AutoGluon prediction of accidental carbon monoxide poisoning by Global Burden of Disease Study 2019. *Environ Sci Pollut Res* 2022; **29**: 6911–28.

21 James SL, Gubbins P, Murray CJ, Gakidou E. Developing a comprehensive time series of GDP per capita for 210 countries from 1950 to 2015. *Population Health Metrics* 2012; **10**: 12.

22 Barber RM, Fullman N, Sorensen RJD, *et al.* Healthcare Access and Quality Index based on mortality from causes amenable to personal health care in 195 countries and territories, 1990–2015: a novel analysis from the Global Burden of Disease Study 2015. *The Lancet* 2017; **390**: 231–66.

23 Zheng P, Barber R, Sorensen RJD, Murray CJL, Aravkin AY. Trimmed Constrained Mixed Effects Models: Formulations and Algorithms. *Journal of Computational and Graphical Statistics* 2021; **30**: 544–56.

24 Griswold MG, Fullman N, Hawley C, *et al.* Alcohol use and burden for 195 countries and territories, 1990–2016: a systematic analysis for the Global Burden of Disease Study 2016. *The Lancet* 2018; **392**: 1015–35.

Section 4 Supplementary figures and tables

Figure S1 Input data availability for carbon monoxide poisoning mortality

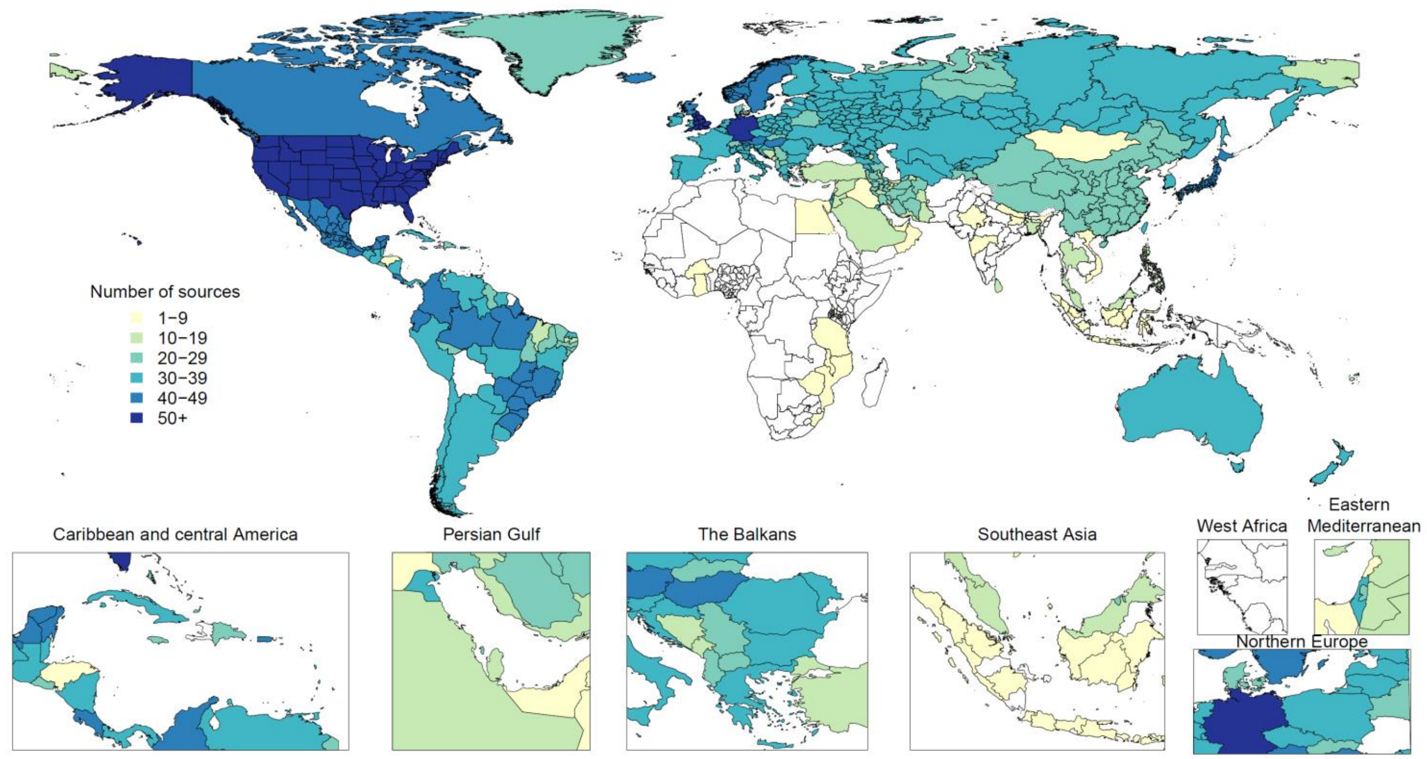

Note that this figure displays all available data in the GBD for carbon monoxide poisoning deaths, from 1980 onward. The list of citations for all input data sources is available in table S9 (appendix pp 58-120). In the absence of data, the cause of death ensemble model (CODEm) framework relies on predictive covariates and information inferred from nearby countries, age groups, or years with data to make estimates.

Table S2 Super-regional, regional, and national breakdown of locations with data sources for carbon monoxide poisoning

Super-region	Region	Countries
Central Europe, eastern Europe, and central Asia	Central Asia	Armenia, Azerbaijan, Georgia, Kazakhstan, Kyrgyzstan, Mongolia, Tajikistan, Turkmenistan, Uzbekistan
	Central Europe	Albania, Bosnia and Herzegovina, Bulgaria, Croatia, Czechia, Hungary, Montenegro, North Macedonia, Poland, Romania, Serbia, Slovakia, Slovenia
	Eastern Europe	Belarus, Estonia, Latvia, Lithuania, Moldova, Russia, Ukraine
High-income	Australasia	Australia, New Zealand
	High-income Asia Pacific	Brunei, Japan, South Korea, Singapore
	High-income North America	Canada, Greenland, USA
	Western Europe	Andorra, Austria, Belgium, Cyprus, Denmark, Finland, France, Germany, Greece, Iceland, Ireland, Israel, Italy, Luxembourg, Malta, Monaco, Netherlands, Norway, Portugal, San Marino, Spain, Sweden, Switzerland, UK
	Southern Latin America	Argentina, Chile, Uruguay
	Andean Latin America	Ecuador, Peru

Latin America and Caribbean	Caribbean	Antigua and Barbuda, The Bahamas, Barbados, Belize, Bermuda, Cuba, Dominica, Dominican Republic, Grenada, Guyana, Jamaica, Puerto Rico, Saint Kitts and Nevis, Saint Lucia, Saint Vincent and the Grenadines, Suriname, Trinidad and Tobago, Virgin Islands
	Central Latin America	Colombia, Costa Rica, El Salvador, Guatemala, Honduras, Mexico, Nicaragua, Panama, Venezuela
	Tropical Latin America	Brazil, Paraguay
North Africa and Middle East	North Africa and Middle East	Bahrain, Egypt, Iran, Iraq, Jordan, Kuwait, Lebanon, Oman, Palestine, Qatar, Saudi Arabia, Syria, Türkiye, United Arab Emirates
South Asia	South Asia	Bangladesh, India, Nepal
Southeast Asia, east Asia, and Oceania	East Asia	China, Taiwan (province of China)
	Oceania	America Samoa, Cook Islands, Fiji, Guam, Kiribati, Northern Mariana Islands, Palau
	Southeast Asia	Indonesia, Malaysia, Maldives, Mauritius, Philippines, Seychelles, Sri Lanka, Thailand, Viet Nam
Sub-Saharan Africa	Eastern sub-Saharan Africa	Ethiopia, Kenya, Malawi, Mozambique, Tanzania
	Southern sub-Saharan Africa	South Africa, Zimbabwe
	Western sub-Saharan Africa	Burkina Faso, Cabo Verde, Ghana, São Tomé and Príncipe

In the absence of data, the cause of death ensemble model (CODEm) framework relies on predictive covariates and information inferred from nearby countries, age groups, or years with data to make estimates.

Table S3 National and subnational sources in countries with subnational estimation for carbon monoxide poisoning

Country	Number of subnational and national sources
Brazil	National = 40, Subnational = 896
China	National = 65, Subnational = 838
Ethiopia	National = 5, Subnational = 5
India	National = 1, Subnational = 5
Indonesia	National = 8, Subnational = 39
Iran	National = 22, Subnational = 617
Italy	National = 39, Subnational = 819
Japan	National = 40, Subnational = 1880
Kenya	National = 1, Subnational = 1
Mexico	National = 40, Subnational = 1276
New Zealand	National = 37, Subnational = 74
Norway	National = 40, Subnational = 440
Philippines	National = 13, Subnational = 957
Poland	National = 38, Subnational = 608
Russia	National = 32, Subnational = 2589
South Africa	National = 1, Subnational = 1
Sweden	National = 47, Subnational = 80
UK	National = 149, Subnational = 5967
USA	National = 79, Subnational = 4029

Note this table only displays countries where we produce national and subnational estimates in the Global Burden of Disease study and does not display all countries with input data for the model, which are listed in Table S2.

Figure S2 Covariates selected in the carbon monoxide poisoning CODEm models and their relative influence on estimates.

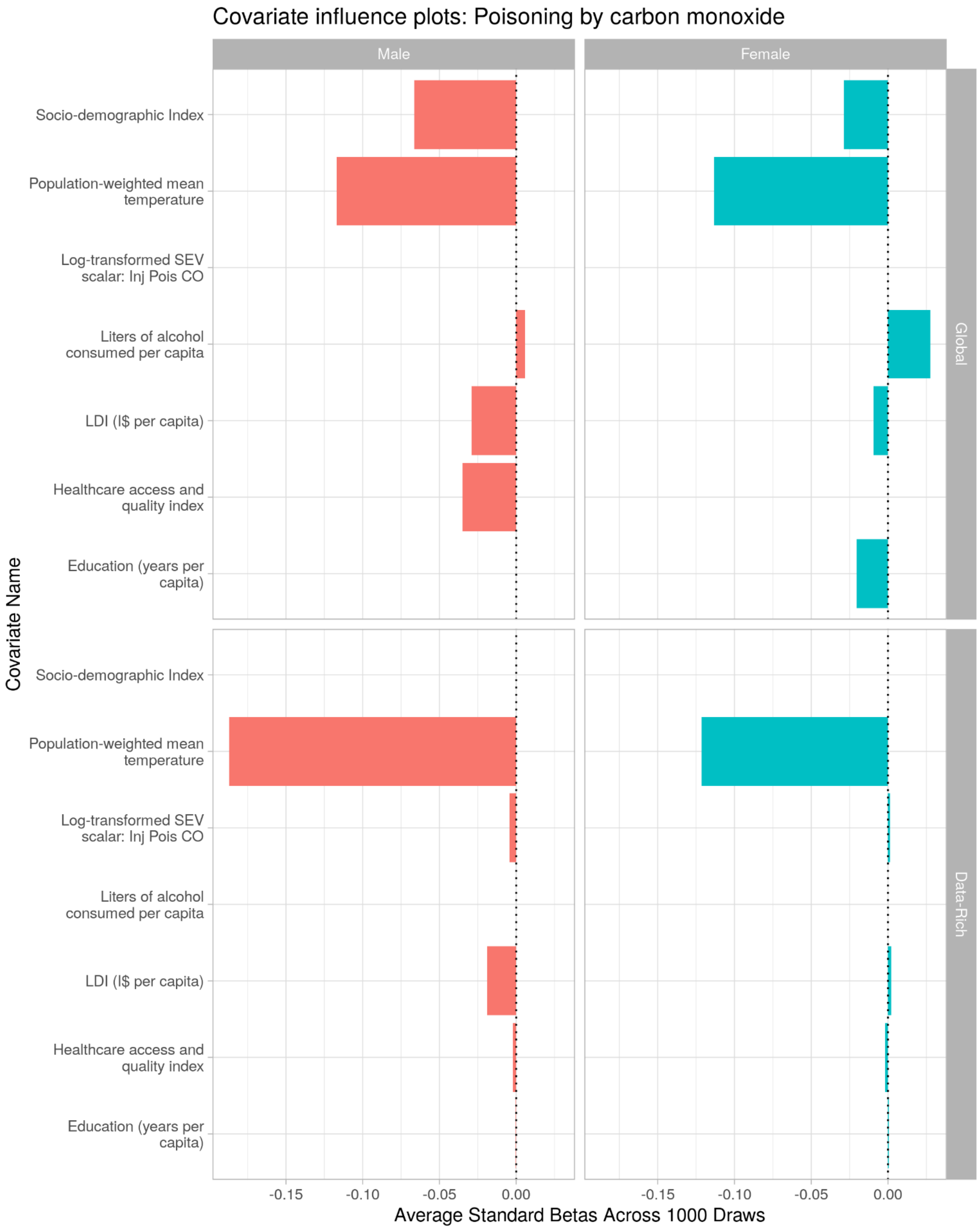

"Global" models are those that use all input data, while "data-rich" models are those that use the subset of location-years of input data from vital registration sources with the highest degree of completeness. See appendix pp 10-11 for more information on covariates.

Figure S3 Population attributable fractions (PAFs) of risk factors for carbon monoxide poisoning mortality by age and sex, 2021, with 95% uncertainty intervals (UIs) demonstrated by the error bars

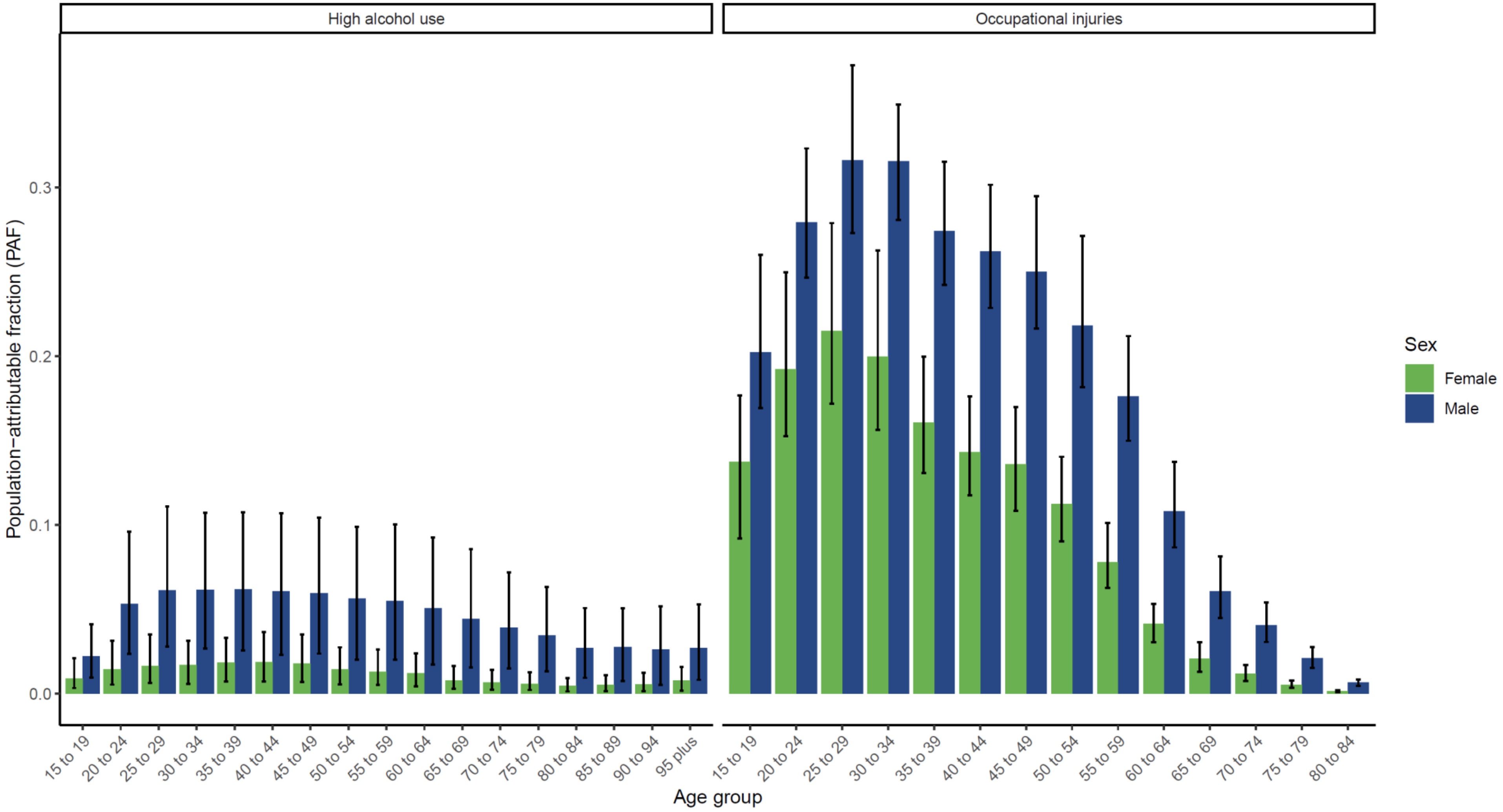

Table S4 Global and regional counts and age-standardised rates of years of life lost (YLLs) due to carbon monoxide poisoning in 2000 and 2021 for both sexes combined, and percentage change for each metric, 2000 to 2021, with 95% UIs

	Number, all-ages (95% UI)			Rate per 100,000, age-standardised (95% UI)		
**Location**	**2000**	**2021**	**Percentage change, 2000 to 2021**	**2000**	**2021**	**Percentage change, 2000 to 2021**
Global	2110000 (1760000 to 2250000)	1180000 (886000 to 1350000)	-43·9 (-55·1 to -36·7)	35·8 (29·9 to 38·1)	14·9 (11·1 to 17·0)	-58·1 (-66·4 to -52·8)
Andean Latin America	1620 (982 to 1950)	2340 (1550 to 2950)	44·5 (16·5 to 82·9)	3·29 (2·03 to 3·91)	3·48 (2·29 to 4·37)	5·2 (-15·1 to 33·4)
Australasia	2070 (1980 to 2170)	1290 (1240 to 1360)	-37·7 (-41·7 to -32·4)	9·42 (9·01 to 9·91)	4·35 (4·18 to 4·57)	-53·4 (-56·5 to -49·1)
Caribbean	2730 (1880 to 4020)	2410 (1460 to 3680)	-11·7 (-35·2 to 16·8)	7·81 (5·25 to 11·9)	6·36 (3·70 to 9·85)	-19·4 (-40·4 to 5·8)
Central Asia	110000 (101000 to 117000)	43600 (37800 to 49800)	-60·3 (-64·9 to -54·5)	161 (147 to 171)	45·7 (39·5 to 52·2)	-71·5 (-74·7 to -67·2)
Central Europe	59700 (58100 to 61500)	19000 (17800 to 20500)	-68·2 (-70·2 to -65·8)	51·1 (49·4 to 53·1)	15·0 (13·9 to 16·3)	-70·0 (-72·1 to -67·6)
Central Latin America	23600 (22700 to 24600)	19600 (17500 to 22100)	-17·1 (-25·0 to -7·5)	11·8 (11·3 to 12·2)	7·90 (7·03 to 8·97)	-32·7 (-39·2 to -24·4)
Central sub-Saharan Africa	3590 (979 to 13900)	3740 (752 to 15000)	4·4 (-64·6 to 91·8)	4·79 (1·61 to 18·0)	3·39 (0·657 to 13·9)	-30·4 (-74·5 to 5·2)
East Asia	761000 (554000 to 871000)	503000 (294000 to 605000)	-33·8 (-58·9 to -15·3)	70·2 (47·0 to 80·2)	35·6 (20·8 to 42·5)	-48·2 (-66·1 to -35·9)
Eastern Europe	750000 (741000 to 759000)	217000 (201000 to 235000)	-71·1 (-73·2 to -68·8)	327 (324 to 331)	95·5 (89·6 to 103)	-70·8 (-72·6 to -68·7)
Eastern sub-Saharan Africa	11500 (4930 to 42100)	10500 (2900 to 43600)	-8·3 (-59·7 to 46·5)	5·04 (2·02 to 16·6)	3·09 (0·819 to 13·1)	-39·0 (-72·5 to -12·9)
High-income Asia Pacific	18900 (17400 to 24100)	11000 (10100 to 14500)	-42·0 (-50·0 to -37·3)	10·2 (9·44 to 13·0)	5·66 (5·16 to 7·61)	-44·2 (-52·1 to -38·3)
High-income North America	37800 (36900 to 38400)	56500 (54600 to 58400)	49·7 (43·0 to 55·1)	12·1 (11·8 to 12·3)	15·6 (15·0 to 16·1)	29·0 (23·1 to 33·7)
North Africa and Middle East	158000 (86700 to 204000)	146000 (76400 to 198000)	-7·9 (-27·4 to 13·6)	36·8 (20·2 to 47·2)	23·7 (12·5 to 32·1)	-35·4 (-47·5 to -20·2)
Oceania	1120 (500 to 1980)	1490 (718 to 2550)	32·7 (-17·7 to 113·2)	12·5 (5·37 to 21·3)	10·2 (4·69 to 18·3)	-19·1 (-46·6 to 22·5)
South Asia	71300 (35000 to 104000)	63600 (35400 to 83200)	-10·8 (-32·5 to 21·2)	5·05 (2·63 to 7·17)	3·39 (1·89 to 4·48)	-33·3 (-48·7 to -10·1)
Southeast Asia	36100 (14000 to 48000)	35300 (14900 to 47000)	-2·3 (-25·1 to 34·7)	6·50 (2·56 to 8·65)	4·85 (2·10 to 6·44)	-25·5 (-42·2 to 2·5)
Southern Latin America	12500 (12100 to 12900)	10600 (10100 to 11200)	-14·8 (-19·5 to -10·2)	23·8 (23·0 to 24·6)	16·7 (15·6 to 17·7)	-29·6 (-33·8 to -25·3)
Southern sub-Saharan Africa	8680 (4880 to 12100)	9060 (5750 to 13600)	4·3 (-24·6 to 42·8)	12·8 (7·14 to 17·9)	11·3 (7·37 to 16·5)	-14·4 (-38·6 to 17·3)
Tropical Latin America	3350 (3200 to 3530)	3170 (3040 to 3340)	-5·3 (-10·1 to 0·1)	1·93 (1·84 to 2·04)	1·41 (1·34 to 1·48)	-25·7 (-29·6 to -21·6)
Western Europe	28700 (28100 to 29200)	17400 (16800 to 18000)	-39·2 (-40·6 to -37·7)	7·12 (6·98 to 7·25)	3·77 (3·66 to 3·88)	-46·9 (-48·0 to -45·5)
Western sub-Saharan Africa	4040 (2280 to 8400)	4940 (2840 to 13000)	22·3 (-25·2 to 81·3)	2·57 (1·42 to 5·21)	1·63 (0·947 to 4·18)	-36·7 (-61·2 to -2·8)

Estimates were computed at the draw level in order to propagate uncertainty; manual calculations (eg, for percentage change or grouped locations) may result in slight differences.

Table S5 Global age-specific counts and rates of fatal unintentional carbon monoxide poisoning deaths, 2021, with 95% UIs

	Both sexes		Female		Male	
**Age group (years)**	**Number (95% UI)**	**Rate (per 100,000) (95% UI)**	**Number (95% UI)**	**Rate (per 100,000) (95% UI)**	**Number (95% UI)**	**Rate (per 100,000) (95% UI)**
Under 5	977 (615 to 1310)	0·148 (0·0935 to 0·198)	361 (169 to 510)	0·114 (0·0530 to 0·160)	616 (406 to 877)	0·181 (0·119 to 0·258)
5 to 9	527 (336 to 684)	0·0766 (0·0489 to 0·0996)	203 (104 to 268)	0·0612 (0·0313 to 0·0805)	323 (217 to 410)	0·0912 (0·0612 to 0·116)
10 to 14	609 (405 to 719)	0·0914 (0·0608 to 0·108)	231 (103 to 304)	0·0714 (0·0318 to 0·0942)	379 (272 to 479)	0·110 (0·0792 to 0·139)
15 to 19	939 (667 to 1140)	0·150 (0·107 to 0·182)	321 (133 to 401)	0·106 (0·0438 to 0·132)	618 (477 to 750)	0·193 (0·149 to 0·234)
20 to 24	1330 (938 to 1530)	0·222 (0·157 to 0·257)	372 (158 to 441)	0·126 (0·0539 to 0·150)	956 (643 to 1180)	0·315 (0·212 to 0·388)
25 to 29	1410 (1000 to 1650)	0·239 (0·170 to 0·280)	338 (162 to 402)	0·116 (0·0558 to 0·138)	1070 (765 to 1320)	0·360 (0·257 to 0·444)
30 to 34	1850 (1340 to 2080)	0·306 (0·221 to 0·344)	428 (202 to 518)	0·143 (0·0674 to 0·173)	1420 (1090 to 1660)	0·465 (0·355 to 0·542)
35 to 39	1970 (1440 to 2270)	0·351 (0·257 to 0·404)	433 (263 to 522)	0·156 (0·0947 to 0·188)	1540 (1110 to 1860)	0·543 (0·392 to 0·657)
40 to 44	2010 (1570 to 2290)	0·401 (0·313 to 0·457)	438 (243 to 527)	0·176 (0·0981 to 0·213)	1570 (1280 to 1860)	0·623 (0·507 to 0·737)
45 to 49	2050 (1580 to 2380)	0·434 (0·334 to 0·503)	454 (250 to 538)	0·193 (0·106 to 0·228)	1600 (1250 to 1890)	0·673 (0·526 to 0·795)
50 to 54	2210 (1660 to 2590)	0·496 (0·373 to 0·582)	513 (250 to 607)	0·230 (0·112 to 0·272)	1690 (1320 to 2080)	0·763 (0·593 to 0·938)
55 to 59	2200 (1650 to 2610)	0·555 (0·417 to 0·659)	564 (273 to 701)	0·280 (0·136 to 0·349)	1630 (1330 to 2040)	0·839 (0·681 to 1·05)
60 to 64	2060 (1570 to 2360)	0·642 (0·490 to 0·737)	533 (284 to 652)	0·324 (0·173 to 0·397)	1520 (1230 to 1820)	0·979 (0·789 to 1·17)
65 to 69	2130 (1520 to 2500)	0·771 (0·550 to 0·907)	717 (329 to 887)	0·498 (0·229 to 0·616)	1410 (1080 to 1760)	1·07 (0·817 to 1·34)
70 to 74	2060 (1350 to 2480)	1·00 (0·654 to 1·21)	759 (327 to 945)	0·693 (0·298 to 0·864)	1300 (894 to 1660)	1·35 (0·928 to 1·73)
75 to 79	1670 (1110 to 2050)	1·26 (0·845 to 1·56)	681 (275 to 834)	0·945 (0·382 to 1·16)	986 (685 to 1340)	1·65 (1·15 to 2·25)
80 to 84	1550 (1040 to 1840)	1·77 (1·19 to 2·10)	715 (356 to 878)	1·40 (0·698 to 1·72)	832 (610 to 1050)	2·27 (1·66 to 2·85)
85 plus	1350 (953 to 1600)	1·96 (1·38 to 2·32)	724 (400 to 891)	1·63 (0·899 to 2·00)	630 (468 to 786)	2·56 (1·90 to 3·20)
All Ages	28900 (21700 to 32800)	0·366 (0·276 to 0·415)	8780 (4300 to 10200)	0·223 (0·109 to 0·259)	20100 (15800 to 24000)	0·508 (0·399 to 0·606)

Estimates were computed at the draw level in order to propagate uncertainty; manual calculations (eg, for percentage change or grouped locations) may result in slight differences.

Table S6 Global age-specific counts and rates of years of life lost (YLLs) due to carbon monoxide poisoning in 2021, with 95% UIs

	Both sexes		Female		Male	
Age group (years)	Number (95% UI)	Rate (per 100,000) (95% UI)	Number (95% UI)	Rate (per 100,000) (95% UI)	Number (95% UI)	Rate (per 100,000) (95% UI)
Under 5	86000 (54200 to 115000)	13·1 (8·24 to 17·5)	31800 (14800 to 44900)	9·99 (4·66 to 14·1)	54200 (35800 to 77300)	15·9 (10·5 to 22·7)
5 to 9	43600 (27800 to 56600)	6·34 (4·05 to 8·24)	16800 (8620 to 22200)	5·06 (2·59 to 6·67)	26700 (17900 to 33900)	7·54 (5·06 to 9·58)
10 to 14	47200 (31400 to 55700)	7·08 (4·70 to 8·35)	17900 (7960 to 23600)	5·53 (2·46 to 7·29)	29300 (21100 to 37000)	8·53 (6·13 to 10·8)
15 to 19	68000 (48300 to 82200)	10·9 (7·74 to 13·2)	23300 (9630 to 29100)	7·66 (3·17 to 9·57)	44700 (34500 to 54300)	14·0 (10·8 to 16·9)
20 to 24	89700 (63400 to 104000)	15·0 (10·6 to 17·4)	25100 (10700 to 29800)	8·55 (3·64 to 10·1)	64600 (43500 to 79600)	21·3 (14·3 to 26·2)
25 to 29	88200 (62700 to 103000)	15·0 (10·7 to 17·5)	21100 (10200 to 25100)	7·27 (3·49 to 8·64)	67100 (47900 to 82600)	22·6 (16·1 to 27·8)
30 to 34	107000 (77100 to 120000)	17·6 (12·8 to 19·8)	24600 (11600 to 29800)	8·24 (3·88 to 9·97)	81900 (62600 to 95400)	26·8 (20·5 to 31·2)
35 to 39	104000 (75900 to 119000)	18·5 (13·5 to 21·3)	22800 (13800 to 27500)	8·21 (4·98 to 9·88)	81000 (58500 to 98000)	28·6 (20·7 to 34·6)
40 to 44	95900 (74800 to 109000)	19·2 (15·0 to 21·8)	20900 (11600 to 25200)	8·42 (4·68 to 10·1)	75000 (61100 to 88800)	29·8 (24·2 to 35·2)
45 to 49	88000 (67700 to 102000)	18·6 (14·3 to 21·5)	19500 (10700 to 23000)	8·26 (4·55 to 9·77)	68600 (53600 to 81100)	28·8 (22·5 to 34·1)
50 to 54	84000 (63100 to 98400)	18·9 (14·2 to 22·1)	19500 (9500 to 23100)	8·75 (4·26 to 10·3)	64500 (50100 to 79300)	29·0 (22·6 to 35·7)
55 to 59	73200 (55000 to 86900)	18·5 (13·9 to 22·0)	18800 (9100 to 23300)	9·33 (4·53 to 11·6)	54400 (44200 to 68000)	28·0 (22·7 to 34·9)
60 to 64	58900 (44900 to 67600)	18·4 (14·0 to 21·1)	15300 (8150 to 18700)	9·27 (4·95 to 11·3)	43600 (35200 to 52000)	28·1 (22·6 to 33·5)
65 to 69	51200 (36500 to 60200)	18·6 (13·2 to 21·8)	17200 (7920 to 21300)	12·0 (5·50 to 14·8)	34000 (26000 to 42400)	25·8 (19·7 to 32·2)
70 to 74	40700 (26600 to 49000)	19·8 (12·9 to 23·8)	15000 (6450 to 18600)	13·7 (5·89 to 17·0)	25700 (17700 to 32900)	26·7 (18·4 to 34·1)
75 to 79	26100 (17500 to 32200)	19·8 (13·3 to 24·4)	10700 (4310 to 13000)	14·8 (5·98 to 18·1)	15500 (10800 to 21100)	25·9 (18·0 to 35·3)
80 to 84	18800 (12700 to 22300)	21·5 (14·5 to 25·5)	8660 (4310 to 10600)	17·0 (8·47 to 20·9)	10100 (7430 to 12700)	27·6 (20·3 to 34·7)
85 plus	11600 (8170 to 13800)	16·8 (11·8 to 20·0)	6130 (3350 to 7530)	13·8 (7·53 to 16·9)	5480 (4040 to 6850)	22·3 (16·4 to 27·9)
All Ages	1180000 (886000 to 1350000)	15·0 (11·2 to 17·1)	335000 (165000 to 396000)	8·52 (4·19 to 10·1)	846000 (654000 to 991000)	21·4 (16·5 to 25·0)

Estimates were computed at the draw level in order to propagate uncertainty; manual calculations (eg, for percentage change or grouped locations) may result in slight differences.

Table S7 Global and country-level counts and age-standardised rates of fatal unintentional carbon monoxide poisoning in 2000 and 2021, for both sexes, and percentage change, 2000 to 2021, for each metric, with 95% UIs

	Number, all-ages (95% UI)			Rate per 100,000, age-standardised (95% UI)		
**Location**	**2000**	**2021**	**Percentage change, 2000 to 2021**	**2000**	**2021**	**Percentage change, 2000 to 2021**
Global	42800 (37500 to 45700)	28900 (21700 to 32800)	-32·5 (-47·3 to -21·9)	0·761 (0·668 to 0·810)	0·353 (0·265 to 0·401)	-53·5 (-63·7 to -46·2)
Afghanistan	324 (111 to 559)	448 (151 to 687)	38·0 (-6·1 to 104·0)	2·51 (0·855 to 3·99)	1·85 (0·617 to 2·83)	-26·4 (-46·6 to 5·3)
Albania	8·58 (5·79 to 11·0)	4·30 (2·98 to 5·90)	-49·8 (-64·6 to -30·5)	0·302 (0·201 to 0·381)	0·146 (0·101 to 0·200)	-52·1 (-66·3 to -35·5)
Algeria	207 (81·8 to 315)	199 (74·9 to 306)	-4·0 (-27·8 to 30·1)	0·759 (0·304 to 1·15)	0·484 (0·190 to 0·736)	-36·1 (-49·8 to -15·9)
American Samoa	0·0646 (0·0382 to 0·0807)	0·0494 (0·0361 to 0·0657)	-23·5 (-49·3 to 14·6)	0·138 (0·0791 to 0·172)	0·100 (0·0728 to 0·129)	-26·9 (-50·5 to 10·2)
Andorra	0·0000614 (0·0000382 to 0·0000913)	0·0000831 (0·0000262 to 0·000139)	35·4 (-31·1 to 140·4)	0·0000797 (0·0000496 to 0·000117)	0·0000733 (0·0000259 to 0·000118)	-7·5 (-52·2 to 69·0)
Angola	13·2 (4·41 to 48·4)	13·7 (3·67 to 49·5)	4·0 (-63·9 to 83·9)	0·125 (0·0428 to 0·407)	0·0723 (0·0195 to 0·257)	-42·9 (-76·4 to -11·1)
Antigua and Barbuda	0·215 (0·198 to 0·234)	0·139 (0·128 to 0·149)	-35·3 (-41·5 to -30·0)	0·324 (0·297 to 0·361)	0·164 (0·149 to 0·178)	-48·8 (-53·7 to -44·6)
Argentina	192 (185 to 198)	195 (186 to 206)	1·3 (-4·4 to 7·1)	0·534 (0·513 to 0·550)	0·409 (0·388 to 0·431)	-23·1 (-27·5 to -18·9)
Armenia	47·1 (38·8 to 56·1)	15·0 (12·7 to 17·4)	-68·1 (-75·7 to -57·9)	1·42 (1·16 to 1·70)	0·406 (0·345 to 0·468)	-71·4 (-78·2 to -62·4)
Australia	36·1 (34·6 to 37·9)	22·8 (21·6 to 24·1)	-36·8 (-41·0 to -31·7)	0·184 (0·176 to 0·194)	0·0811 (0·0777 to 0·0855)	-55·6 (-58·7 to -51·6)
Austria	15·0 (14·1 to 15·8)	15·3 (14·5 to 16·2)	2·2 (-4·3 to 10·6)	0·156 (0·147 to 0·163)	0·134 (0·127 to 0·141)	-13·5 (-19·4 to -6·5)
Azerbaijan	100 (55·9 to 129)	58·2 (40·6 to 80·0)	-42·1 (-64·3 to -4·1)	1·37 (0·758 to 1·76)	0·518 (0·366 to 0·706)	-62·0 (-75·9 to -37·1)
Bahrain	1·57 (1·34 to 1·99)	2·37 (1·72 to 2·94)	50·8 (18·7 to 90·2)	0·304 (0·265 to 0·374)	0·175 (0·130 to 0·214)	-43·0 (-55·1 to -29·5)
Bangladesh	97·4 (16·7 to 206)	60·5 (14·5 to 113)	-37·8 (-67·4 to 28·4)	0·0655 (0·0157 to 0·118)	0·0371 (0·00913 to 0·0700)	-44·1 (-67·5 to 7·8)
Barbados	0·0629 (0·0561 to 0·0695)	0·0530 (0·0397 to 0·0707)	-15·7 (-35·6 to 16·5)	0·0235 (0·0208 to 0·0265)	0·0147 (0·0109 to 0·0204)	-38·0 (-52·3 to -12·7)
Belarus	722 (684 to 769)	177 (145 to 216)	-75·5 (-79·9 to -69·7)	6·26 (5·92 to 6·69)	1·41 (1·16 to 1·74)	-77·4 (-81·6 to -71·9)
Belgium	41·6 (38·8 to 43·7)	27·7 (25·8 to 29·3)	-33·4 (-37·3 to -29·4)	0·336 (0·315 to 0·354)	0·170 (0·160 to 0·178)	-49·3 (-52·4 to -45·7)
Belize	0·833 (0·771 to 0·893)	0·742 (0·657 to 0·843)	-10·8 (-23·1 to 4·5)	0·424 (0·393 to 0·453)	0·209 (0·184 to 0·238)	-49·3 (-56·3 to -41·2)
Benin	2·08 (0·888 to 4·16)	2·92 (1·22 to 7·52)	40·1 (-26·4 to 156·5)	0·0653 (0·0281 to 0·131)	0·0418 (0·0180 to 0·108)	-35·9 (-66·9 to 20·8)
Bermuda	0·148 (0·135 to 0·161)	0·0619 (0·0529 to 0·0722)	-58·3 (-64·1 to -50·4)	0·224 (0·204 to 0·244)	0·0756 (0·0629 to 0·0903)	-66·4 (-71·7 to -59·2)
Bhutan	0·326 (0·120 to 1·28)	0·253 (0·0982 to 1·16)	-22·3 (-52·5 to 17·1)	0·0572 (0·0216 to 0·231)	0·0358 (0·0145 to 0·160)	-37·6 (-60·0 to -10·6)
Bolivia	5·67 (1·21 to 8·73)	7·33 (1·12 to 11·7)	29·3 (-29·7 to 104·0)	0·0789 (0·0197 to 0·115)	0·0700 (0·0131 to 0·109)	-11·5 (-45·5 to 31·1)
Bosnia and Herzegovina	47·1 (28·6 to 67·1)	32·4 (23·6 to 45·7)	-31·2 (-51·3 to -3·2)	1·17 (0·704 to 1·65)	0·687 (0·507 to 1·03)	-40·7 (-57·7 to -13·6)

Botswana	2·76 (1·17 to 5·02)	1·72 (0·743 to 4·00)	-37·5 (-66·5 to 22·5)	0·165 (0·0675 to 0·313)	0·0718 (0·0329 to 0·168)	-57·2 (-77·7 to -13·5)
Brazil	57·7 (55·8 to 59·9)	64·3 (61·1 to 66·8)	11·4 (4·8 to 17·5)	0·0363 (0·0347 to 0·0377)	0·0279 (0·0265 to 0·0290)	-22·2 (-26·3 to -18·2)
Brunei	0·548 (0·454 to 0·679)	0·577 (0·474 to 0·735)	5·4 (-13·1 to 29·5)	0·198 (0·152 to 0·242)	0·131 (0·109 to 0·165)	-33·9 (-44·1 to -19·1)
Bulgaria	76·7 (70·3 to 84·0)	29·7 (26·0 to 33·8)	-61·2 (-66·9 to -54·2)	0·844 (0·772 to 0·923)	0·304 (0·264 to 0·347)	-63·4 (-69·0 to -56·8)
Burkina Faso	4·34 (1·71 to 10·7)	5·36 (1·99 to 17·4)	23·5 (-40·3 to 98·1)	0·0707 (0·0284 to 0·174)	0·0478 (0·0190 to 0·147)	-32·4 (-66·2 to 13·7)
Burundi	4·90 (1·99 to 14·7)	6·39 (1·54 to 25·5)	30·3 (-54·9 to 110·5)	0·128 (0·0460 to 0·433)	0·0916 (0·0203 to 0·390)	-28·7 (-73·3 to 20·0)
Cabo Verde	0·0449 (0·00825 to 0·0696)	0·115 (0·0153 to 0·182)	156·6 (40·5 to 359·4)	0·0185 (0·00340 to 0·0312)	0·0221 (0·00301 to 0·0349)	19·3 (-38·0 to 118·2)
Cambodia	10·0 (4·57 to 17·7)	12·0 (6·73 to 23·0)	19·2 (-23·1 to 90·4)	0·0997 (0·0486 to 0·169)	0·0784 (0·0447 to 0·146)	-21·4 (-44·5 to 17·0)
Cameroon	6·61 (3·67 to 14·3)	10·1 (4·57 to 25·6)	52·5 (-24·6 to 163·3)	0·0852 (0·0470 to 0·188)	0·0582 (0·0257 to 0·145)	-31·7 (-67·0 to 19·2)
Canada	82·3 (78·2 to 86·3)	94·0 (87·7 to 100)	14·2 (6·7 to 23·8)	0·240 (0·229 to 0·253)	0·212 (0·198 to 0·226)	-11·8 (-18·3 to -4·5)
Central African Republic	3·35 (0·925 to 14·4)	4·05 (0·578 to 19·3)	20·7 (-58·4 to 100·2)	0·137 (0·0400 to 0·554)	0·109 (0·0145 to 0·515)	-21·3 (-71·7 to 27·0)
Chad	3·16 (1·37 to 7·26)	4·67 (1·97 to 12·7)	47·9 (-21·4 to 137·6)	0·0785 (0·0341 to 0·174)	0·0595 (0·0247 to 0·163)	-24·0 (-61·0 to 25·3)
Chile	37·6 (35·4 to 39·5)	36·5 (34·5 to 38·5)	-2·8 (-9·2 to 3·3)	0·259 (0·244 to 0·273)	0·175 (0·166 to 0·185)	-32·8 (-37·3 to -28·5)
China	14200 (10800 to 16600)	13200 (7250 to 16300)	-6·9 (-46·8 to 18·9)	1·33 (0·972 to 1·52)	0·812 (0·455 to 0·981)	-38·1 (-62·9 to -22·3)
Colombia	36·5 (34·4 to 38·6)	35·2 (29·1 to 41·4)	-3·6 (-21·2 to 15·2)	0·0994 (0·0939 to 0·105)	0·0682 (0·0563 to 0·0809)	-31·2 (-43·5 to -18·0)
Comoros	0·320 (0·105 to 1·01)	0·404 (0·0903 to 1·82)	26·0 (-53·7 to 116·1)	0·0874 (0·0275 to 0·286)	0·0703 (0·0158 to 0·313)	-19·8 (-67·4 to 33·4)
Congo (Brazzaville)	2·49 (0·749 to 8·69)	3·00 (0·641 to 11·3)	20·8 (-53·8 to 111·9)	0·114 (0·0368 to 0·392)	0·0817 (0·0188 to 0·323)	-29·0 (-72·2 to 11·6)
Cook Islands	0·0118 (0·00635 to 0·0153)	0·00524 (0·00343 to 0·00779)	-55·4 (-71·0 to -18·1)	0·0666 (0·0360 to 0·0859)	0·0243 (0·0155 to 0·0366)	-63·6 (-76·2 to -33·5)
Costa Rica	1·63 (1·48 to 1·77)	2·23 (1·95 to 2·52)	36·5 (16·9 to 60·8)	0·0491 (0·0451 to 0·0532)	0·0436 (0·0382 to 0·0491)	-10·8 (-23·0 to 4·9)
Croatia	24·2 (21·9 to 26·9)	10·5 (9·26 to 11·6)	-56·6 (-61·8 to -50·6)	0·448 (0·408 to 0·496)	0·153 (0·135 to 0·170)	-65·5 (-69·9 to -60·4)
Cuba	7·98 (7·47 to 8·49)	7·72 (6·71 to 8·70)	-3·2 (-16·1 to 11·6)	0·0728 (0·0683 to 0·0776)	0·0516 (0·0453 to 0·0575)	-27·2 (-36·1 to -16·2)
Cyprus	3·14 (2·56 to 3·84)	2·38 (1·88 to 2·88)	-24·1 (-39·4 to -6·0)	0·335 (0·270 to 0·415)	0·142 (0·112 to 0·172)	-57·6 (-66·4 to -48·2)
Czechia	113 (105 to 120)	43·7 (38·0 to 49·1)	-61·4 (-65·7 to -56·6)	0·940 (0·879 to 0·989)	0·275 (0·243 to 0·310)	-70·5 (-73·8 to -66·7)
Côte d'Ivoire	6·58 (2·84 to 12·6)	8·12 (3·61 to 17·7)	23·4 (-34·5 to 94·3)	0·0805 (0·0343 to 0·155)	0·0516 (0·0239 to 0·113)	-35·9 (-66·4 to 4·1)
DR Congo	40·2 (10·8 to 158)	53·2 (8·39 to 233)	32·3 (-55·3 to 124·5)	0·115 (0·0353 to 0·480)	0·0971 (0·0154 to 0·424)	-15·7 (-68·0 to 26·9)
Denmark	3·74 (3·48 to 3·93)	2·44 (2·25 to 2·59)	-34·7 (-40·8 to -28·6)	0·0572 (0·0536 to 0·0599)	0·0289 (0·0268 to 0·0305)	-49·5 (-53·9 to -45·1)
Djibouti	0·367 (0·0912 to 1·28)	0·581 (0·118 to 2·15)	58·4 (-39·7 to 173·5)	0·0961 (0·0221 to 0·326)	0·0693 (0·0146 to 0·262)	-28·1 (-69·2 to 18·7)
Dominica	0·0605 (0·0200 to 0·0996)	0·0513 (0·0184 to 0·0744)	-15·2 (-40·0 to 24·7)	0·107 (0·0343 to 0·178)	0·0908 (0·0327 to 0·134)	-17·0 (-44·9 to 23·0)

Dominican Republic	5·19 (2·94 to 7·25)	7·81 (3·38 to 11·1)	50·4 (1·0 to 118·6)	0·0686 (0·0369 to 0·0984)	0·0786 (0·0325 to 0·118)	16·7 (-24·9 to 71·4)
Ecuador	14·6 (13·2 to 15·9)	29·5 (23·7 to 36·2)	102·6 (59·9 to 150·8)	0·124 (0·112 to 0·134)	0·165 (0·133 to 0·203)	32·9 (4·9 to 64·6)
Egypt	87·8 (47·6 to 130)	88·5 (55·9 to 114)	0·8 (-39·5 to 44·3)	0·145 (0·0877 to 0·202)	0·101 (0·0654 to 0·130)	-30·7 (-53·6 to -8·7)
El Salvador	4·08 (2·32 to 5·87)	4·12 (2·58 to 6·17)	1·0 (-30·2 to 41·2)	0·0778 (0·0443 to 0·113)	0·0643 (0·0404 to 0·0972)	-17·2 (-43·1 to 14·1)
Equatorial Guinea	0·413 (0·135 to 1·41)	0·461 (0·103 to 1·71)	11·6 (-50·3 to 161·1)	0·0922 (0·0370 to 0·328)	0·0513 (0·0129 to 0·203)	-44·6 (-74·0 to 3·9)
Eritrea	2·75 (1·07 to 9·32)	3·77 (1·09 to 13·8)	36·8 (-45·8 to 130·4)	0·112 (0·0468 to 0·357)	0·0860 (0·0257 to 0·326)	-23·0 (-67·0 to 21·5)
Estonia	35·4 (33·1 to 37·6)	10·2 (8·99 to 11·4)	-71·1 (-74·8 to -67·6)	2·14 (2·00 to 2·27)	0·540 (0·473 to 0·603)	-74·6 (-77·8 to -71·7)
Eswatini	1·90 (1·07 to 3·24)	1·40 (0·611 to 3·30)	-26·0 (-66·4 to 29·8)	0·193 (0·110 to 0·311)	0·123 (0·0560 to 0·288)	-36·4 (-68·8 to 10·0)
Ethiopia	66·9 (16·1 to 232)	51·9 (11·6 to 253)	-22·5 (-67·6 to 35·4)	0·181 (0·0399 to 0·727)	0·0878 (0·0190 to 0·455)	-51·8 (-77·4 to -22·5)
Federated States of Micronesia	0·249 (0·128 to 0·410)	0·194 (0·103 to 0·319)	-22·1 (-52·8 to 13·0)	0·261 (0·138 to 0·422)	0·194 (0·106 to 0·318)	-25·5 (-52·1 to 3·1)
Fiji	0·799 (0·576 to 1·07)	0·664 (0·477 to 0·936)	-16·9 (-45·6 to 12·2)	0·111 (0·0818 to 0·147)	0·0733 (0·0531 to 0·104)	-34·2 (-55·8 to -9·2)
Finland	36·9 (35·3 to 38·3)	23·3 (21·7 to 24·5)	-36·9 (-41·1 to -32·9)	0·606 (0·581 to 0·632)	0·302 (0·285 to 0·320)	-50·2 (-53·5 to -47·1)
France	103 (97·9 to 107)	72·5 (67·6 to 76·9)	-29·8 (-34·8 to -25·8)	0·141 (0·134 to 0·146)	0·0764 (0·0711 to 0·0802)	-45·8 (-48·9 to -43·0)
Gabon	0·826 (0·248 to 2·48)	0·874 (0·215 to 3·26)	5·8 (-54·6 to 92·9)	0·0955 (0·0291 to 0·284)	0·0666 (0·0176 to 0·253)	-30·3 (-69·6 to 29·1)
Georgia	119 (106 to 136)	28·4 (24·7 to 32·0)	-76·2 (-79·4 to -71·1)	2·27 (2·01 to 2·57)	0·630 (0·549 to 0·712)	-72·3 (-76·0 to -66·6)
Germany	133 (127 to 138)	120 (111 to 127)	-9·6 (-15·8 to -4·8)	0·135 (0·130 to 0·140)	0·0964 (0·0905 to 0·101)	-28·4 (-32·8 to -24·2)
Ghana	5·25 (2·66 to 7·81)	10·8 (4·69 to 18·8)	104·8 (15·5 to 247·9)	0·0480 (0·0247 to 0·0721)	0·0494 (0·0227 to 0·0845)	3·2 (-40·0 to 66·4)
Greece	13·7 (12·6 to 14·6)	11·5 (10·6 to 12·4)	-16·2 (-24·1 to -6·1)	0·102 (0·0934 to 0·109)	0·0768 (0·0718 to 0·0818)	-24·7 (-31·4 to -16·1)
Greenland	0·479 (0·411 to 0·571)	0·345 (0·278 to 0·427)	-28·1 (-44·7 to -10·9)	0·838 (0·724 to 0·993)	0·568 (0·469 to 0·700)	-32·5 (-46·5 to -18·1)
Grenada	0·113 (0·100 to 0·125)	0·0298 (0·0260 to 0·0336)	-73·6 (-77·2 to -69·2)	0·130 (0·115 to 0·145)	0·0340 (0·0293 to 0·0386)	-73·2 (-77·0 to -68·6)
Guam	0·137 (0·0961 to 0·165)	0·102 (0·0798 to 0·151)	-25·7 (-46·4 to 19·0)	0·0928 (0·0645 to 0·111)	0·0597 (0·0467 to 0·0869)	-35·7 (-53·7 to 3·1)
Guatemala	10·4 (9·33 to 11·7)	10·7 (8·79 to 12·7)	3·3 (-19·5 to 29·7)	0·114 (0·102 to 0·128)	0·0738 (0·0609 to 0·0866)	-34·8 (-49·0 to -18·7)
Guinea	3·24 (1·36 to 6·90)	3·47 (1·33 to 8·92)	7·0 (-45·0 to 83·9)	0·0682 (0·0278 to 0·148)	0·0496 (0·0180 to 0·127)	-27·1 (-63·3 to 30·1)
Guinea-Bissau	0·568 (0·255 to 1·24)	0·701 (0·328 to 1·87)	23·5 (-42·1 to 118·8)	0·0946 (0·0428 to 0·201)	0·0671 (0·0312 to 0·174)	-29·1 (-66·3 to 28·1)
Guyana	1·46 (1·23 to 1·66)	1·05 (0·816 to 1·33)	-28·1 (-47·9 to -1·8)	0·209 (0·182 to 0·233)	0·146 (0·114 to 0·184)	-29·9 (-48·1 to -7·2)
Haiti	18·5 (8·26 to 34·2)	22·3 (10·5 to 40·7)	20·5 (-24·9 to 93·8)	0·270 (0·111 to 0·456)	0·208 (0·0981 to 0·370)	-22·7 (-48·9 to 16·1)
Honduras	11·1 (4·85 to 17·6)	14·3 (6·43 to 22·2)	29·0 (-18·4 to 109·6)	0·210 (0·102 to 0·299)	0·172 (0·0812 to 0·255)	-18·5 (-45·7 to 25·5)
Hungary	70·4 (67·0 to 73·7)	29·9 (26·4 to 33·0)	-57·5 (-62·9 to -51·2)	0·582 (0·555 to 0·606)	0·218 (0·192 to 0·242)	-61·9 (-66·9 to -56·4)

Iceland	0·707<br>(0·664 to 0·750)	0·690<br>(0·628 to 0·758)	-2·4<br>(-11·9 to 8·2)	0·236<br>(0·221 to 0·251)	0·163<br>(0·148 to 0·179)	-30·5<br>(-37·1 to -23·0)
India	710<br>(165 to 1040)	720<br>(197 to 998)	1·5<br>(-18·0 to 40·0)	0·0722<br>(0·0187 to 0·103)	0·0513<br>(0·0147 to 0·0713)	-29·1<br>(-41·8 to -3·3)
Indonesia	184<br>(67·3 to 294)	235<br>(86·2 to 384)	27·7<br>(-7·2 to 85·0)	0·0873<br>(0·0335 to 0·139)	0·0807<br>(0·0306 to 0·132)	-7·5<br>(-32·0 to 35·3)
Iran	658<br>(385 to 809)	479<br>(323 to 561)	-27·3<br>(-44·6 to -5·6)	1·14<br>(0·667 to 1·39)	0·546<br>(0·387 to 0·634)	-51·1<br>(-62·7 to -36·8)
Iraq	62·7<br>(37·5 to 85·3)	57·9<br>(38·1 to 80·6)	-7·6<br>(-38·5 to 36·0)	0·291<br>(0·179 to 0·393)	0·163<br>(0·110 to 0·222)	-44·0<br>(-61·9 to -17·7)
Ireland	5·85<br>(5·55 to 6·20)	4·15<br>(3·70 to 4·53)	-29·0<br>(-36·9 to -21·6)	0·140<br>(0·132 to 0·148)	0·0684<br>(0·0616 to 0·0744)	-50·9<br>(-56·2 to -45·9)
Israel	2·68<br>(2·46 to 2·88)	2·73<br>(2·48 to 2·95)	1·9<br>(-9·6 to 13·6)	0·0419<br>(0·0386 to 0·0450)	0·0260<br>(0·0237 to 0·0280)	-37·8<br>(-44·8 to -30·6)
Italy	95·4<br>(90·7 to 98·7)	78·1<br>(71·4 to 82·5)	-18·1<br>(-23·2 to -14·6)	0·128<br>(0·124 to 0·132)	0·0778<br>(0·0726 to 0·0813)	-39·3<br>(-42·2 to -37·1)
Jamaica	0·748<br>(0·674 to 0·825)	0·803<br>(0·643 to 1·03)	7·3<br>(-14·8 to 39·1)	0·0325<br>(0·0291 to 0·0360)	0·0284<br>(0·0225 to 0·0360)	-9·9<br>(-28·5 to 18·3)
Japan	230<br>(225 to 236)	182<br>(171 to 189)	-20·9<br>(-24·9 to -17·7)	0·145<br>(0·142 to 0·148)	0·112<br>(0·108 to 0·115)	-22·8<br>(-24·8 to -20·1)
Jordan	41·0<br>(28·5 to 50·7)	43·6<br>(35·7 to 54·4)	6·4<br>(-20·5 to 38·2)	1·05<br>(0·721 to 1·28)	0·422<br>(0·343 to 0·534)	-59·8<br>(-69·2 to -48·4)
Kazakhstan	779<br>(705 to 863)	330<br>(297 to 369)	-57·6<br>(-62·2 to -50·2)	5·63<br>(5·09 to 6·24)	1·76<br>(1·59 to 1·96)	-68·2<br>(-71·6 to -62·8)
Kenya	25·7<br>(9·44 to 89·0)	36·1<br>(7·11 to 149)	40·6<br>(-35·5 to 107·9)	0·137<br>(0·0466 to 0·519)	0·120<br>(0·0230 to 0·524)	-12·4<br>(-54·5 to 36·7)
Kiribati	0·263<br>(0·116 to 0·350)	0·434<br>(0·143 to 0·656)	64·9<br>(-0·2 to 167·9)	0·343<br>(0·155 to 0·448)	0·381<br>(0·135 to 0·557)	11·0<br>(-29·7 to 64·9)
Kuwait	10·5<br>(9·98 to 11·0)	14·6<br>(12·3 to 17·3)	38·9<br>(17·9 to 60·5)	0·619<br>(0·590 to 0·653)	0·349<br>(0·297 to 0·412)	-43·8<br>(-51·3 to -35·4)
Kyrgyzstan	237<br>(215 to 257)	64·2<br>(56·5 to 74·3)	-72·9<br>(-77·0 to -67·7)	5·77<br>(5·22 to 6·25)	1·05<br>(0·926 to 1·21)	-81·9<br>(-84·7 to -78·4)
Laos	4·70<br>(2·54 to 8·89)	4·88<br>(2·72 to 10·1)	3·9<br>(-40·1 to 70·3)	0·109<br>(0·0610 to 0·193)	0·0726<br>(0·0421 to 0·149)	-33·7<br>(-59·2 to 2·5)
Latvia	72·6<br>(69·6 to 76·4)	19·7<br>(17·8 to 21·6)	-72·8<br>(-76·1 to -69·8)	2·58<br>(2·47 to 2·70)	0·716<br>(0·641 to 0·781)	-72·1<br>(-75·5 to -69·0)
Lebanon	3·22<br>(1·89 to 4·18)	3·96<br>(2·57 to 4·97)	23·0<br>(-4·9 to 81·3)	0·0988<br>(0·0573 to 0·128)	0·0675<br>(0·0440 to 0·0849)	-31·8<br>(-47·0 to 0·6)
Lesotho	3·10<br>(1·87 to 5·79)	2·78<br>(1·40 to 6·51)	-10·3<br>(-52·1 to 59·2)	0·182<br>(0·103 to 0·343)	0·151<br>(0·0773 to 0·350)	-17·6<br>(-55·8 to 51·6)
Liberia	1·03<br>(0·567 to 2·60)	1·59<br>(0·648 to 4·32)	54·8<br>(-23·2 to 146·7)	0·0701<br>(0·0366 to 0·180)	0·0531<br>(0·0209 to 0·147)	-24·3<br>(-61·8 to 27·2)
Libya	28·9<br>(12·9 to 37·8)	54·3<br>(19·8 to 84·5)	87·6<br>(31·7 to 162·5)	0·664<br>(0·286 to 0·858)	0·781<br>(0·289 to 1·21)	17·6<br>(-15·9 to 70·9)
Lithuania	154<br>(149 to 161)	53·2<br>(47·5 to 58·6)	-65·6<br>(-69·7 to -62·1)	3·88<br>(3·76 to 4·05)	1·34<br>(1·19 to 1·46)	-65·3<br>(-69·4 to -62·1)
Luxembourg	0·668<br>(0·628 to 0·708)	0·542<br>(0·489 to 0·599)	-18·8<br>(-25·4 to -9·1)	0·132<br>(0·125 to 0·139)	0·0623<br>(0·0568 to 0·0688)	-52·7<br>(-56·6 to -47·3)
Madagascar	10·1<br>(4·05 to 34·7)	11·8<br>(3·07 to 55·3)	16·4<br>(-53·3 to 99·4)	0·0990<br>(0·0366 to 0·369)	0·0687<br>(0·0167 to 0·304)	-30·7<br>(-66·1 to 12·9)
Malawi	8·89<br>(3·25 to 34·5)	8·80<br>(1·87 to 36·9)	-1·0<br>(-66·2 to 75·1)	0·116<br>(0·0420 to 0·428)	0·0778<br>(0·0169 to 0·339)	-33·4<br>(-73·6 to 3·8)
Malaysia	32·4<br>(22·8 to 43·5)	36·8<br>(28·1 to 56·1)	13·6<br>(-20·8 to 52·6)	0·156<br>(0·108 to 0·207)	0·115<br>(0·0897 to 0·176)	-26·2<br>(-47·4 to 0·9)
Maldives	0·0408<br>(0·0235 to 0·0708)	0·0709<br>(0·0390 to 0·205)	73·6<br>(-9·9 to 255·1)	0·0179<br>(0·0102 to 0·0299)	0·0120<br>(0·00716 to 0·0313)	-33·0<br>(-61·9 to 32·3)

Mali	3·93 (1·36 to 8·67)	5·50 (1·79 to 14·9)	39·9 (-27·0 to 152·0)	0·0680 (0·0238 to 0·150)	0·0482 (0·0161 to 0·131)	-28·6 (-62·9 to 32·5)
Malta	0·440 (0·414 to 0·469)	0·336 (0·310 to 0·366)	-23·8 (-32·2 to -15·6)	0·0989 (0·0928 to 0·105)	0·0581 (0·0527 to 0·0638)	-41·1 (-47·7 to -33·6)
Marshall Islands	0·108 (0·0474 to 0·189)	0·0982 (0·0438 to 0·174)	-8·8 (-39·1 to 47·4)	0·245 (0·111 to 0·431)	0·190 (0·0863 to 0·338)	-22·7 (-46·4 to 9·9)
Mauritania	0·730 (0·374 to 1·49)	0·976 (0·423 to 2·54)	33·6 (-31·2 to 131·1)	0·0515 (0·0268 to 0·106)	0·0385 (0·0170 to 0·101)	-25·3 (-63·0 to 29·9)
Mauritius	3·34 (3·17 to 3·53)	2·30 (2·12 to 2·43)	-31·1 (-37·1 to -25·7)	0·285 (0·270 to 0·302)	0·160 (0·147 to 0·170)	-43·4 (-48·3 to -39·0)
Mexico	315 (305 to 327)	307 (276 to 335)	-2·6 (-12·6 to 4·9)	0·359 (0·346 to 0·370)	0·243 (0·219 to 0·265)	-32·3 (-38·9 to -27·2)
Moldova	246 (237 to 255)	135 (120 to 149)	-45·0 (-50·7 to -39·0)	5·59 (5·38 to 5·79)	2·74 (2·42 to 3·04)	-50·8 (-56·1 to -45·2)
Monaco	0·00784 (0·00403 to 0·0123)	0·00640 (0·00393 to 0·00893)	-18·4 (-48·6 to 27·4)	0·0185 (0·0102 to 0·0291)	0·0133 (0·00751 to 0·0191)	-28·0 (-58·7 to 13·6)
Mongolia	137 (90·1 to 191)	77·1 (58·3 to 105)	-43·6 (-61·3 to -7·3)	7·10 (4·76 to 9·97)	2·48 (1·89 to 3·30)	-65·5 (-76·1 to -44·5)
Montenegro	3·54 (2·71 to 4·11)	2·17 (1·66 to 2·83)	-38·6 (-51·5 to -20·6)	0·539 (0·414 to 0·624)	0·287 (0·220 to 0·386)	-46·6 (-57·1 to -30·0)
Morocco	243 (78·9 to 401)	230 (87·5 to 382)	-5·5 (-28·8 to 32·1)	0·898 (0·301 to 1·46)	0·638 (0·245 to 1·06)	-28·7 (-44·9 to -4·7)
Mozambique	12·5 (4·09 to 55·4)	13·3 (3·74 to 57·0)	6·9 (-52·1 to 84·3)	0·102 (0·0325 to 0·397)	0·0752 (0·0201 to 0·323)	-26·6 (-66·5 to 14·9)
Myanmar	150 (36·7 to 257)	113 (37·6 to 157)	-24·9 (-54·1 to 51·3)	0·387 (0·0868 to 0·624)	0·222 (0·0701 to 0·310)	-42·7 (-63·9 to 9·2)
Namibia	2·60 (1·51 to 4·64)	2·09 (0·977 to 4·65)	-19·3 (-56·0 to 52·6)	0·149 (0·0851 to 0·255)	0·0888 (0·0426 to 0·197)	-41·2 (-65·6 to 9·7)
Nauru	0·0273 (0·0127 to 0·0420)	0·0196 (0·0114 to 0·0306)	-28·3 (-47·3 to 3·2)	0·303 (0·153 to 0·461)	0·199 (0·120 to 0·303)	-34·4 (-51·4 to -9·2)
Nepal	307 (168 to 549)	334 (180 to 556)	8·7 (-23·4 to 60·4)	1·64 (0·900 to 2·60)	1·28 (0·702 to 2·02)	-22·3 (-42·4 to 9·9)
Netherlands	10·6 (10·1 to 11·1)	7·55 (6·99 to 8·01)	-28·8 (-33·4 to -23·7)	0·0562 (0·0538 to 0·0590)	0·0307 (0·0288 to 0·0324)	-45·2 (-48·5 to -41·9)
New Zealand	4·11 (3·90 to 4·33)	5·27 (5·05 to 5·54)	28·2 (18·5 to 37·6)	0·106 (0·101 to 0·113)	0·0943 (0·0902 to 0·0997)	-11·2 (-18·3 to -4·9)
Nicaragua	2·80 (1·67 to 4·17)	2·55 (1·85 to 4·56)	-8·9 (-32·2 to 27·8)	0·0663 (0·0393 to 0·0993)	0·0429 (0·0309 to 0·0751)	-35·3 (-50·4 to -11·9)
Niger	3·69 (1·46 to 7·94)	4·76 (1·79 to 13·6)	29·0 (-20·7 to 107·5)	0·0683 (0·0260 to 0·152)	0·0436 (0·0156 to 0·124)	-36·1 (-62·8 to 2·6)
Nigeria	53·2 (25·1 to 115)	53·6 (26·9 to 153)	0·8 (-49·3 to 89·2)	0·0844 (0·0418 to 0·178)	0·0463 (0·0244 to 0·122)	-45·2 (-71·9 to 0·7)
Niue	0·00412 (0·00249 to 0·00636)	0·00261 (0·00166 to 0·00406)	-36·6 (-55·6 to -9·6)	0·205 (0·123 to 0·317)	0·143 (0·0882 to 0·225)	-30·3 (-51·1 to 0·2)
North Korea	180 (117 to 334)	216 (143 to 378)	20·2 (-11·7 to 59·0)	0·832 (0·534 to 1·53)	0·748 (0·484 to 1·32)	-10·0 (-33·4 to 20·8)
North Macedonia	7·07 (5·22 to 8·65)	4·88 (3·41 to 6·44)	-31·1 (-45·6 to -15·5)	0·363 (0·265 to 0·441)	0·191 (0·136 to 0·252)	-46·5 (-57·6 to -34·1)
Northern Mariana Islands	0·144 (0·0769 to 0·198)	0·162 (0·0810 to 0·223)	12·0 (-20·4 to 66·7)	0·266 (0·150 to 0·346)	0·306 (0·157 to 0·409)	15·2 (-15·1 to 61·1)
Norway	1·78 (1·73 to 1·84)	4·09 (3·90 to 4·27)	129·7 (118·3 to 139·8)	0·0400 (0·0389 to 0·0415)	0·0656 (0·0624 to 0·0682)	63·8 (55·4 to 70·3)
Oman	1·23 (0·823 to 1·58)	1·44 (1·00 to 1·92)	16·9 (-13·7 to 60·4)	0·0707 (0·0463 to 0·0877)	0·0373 (0·0256 to 0·0493)	-47·5 (-59·2 to -30·0)
Pakistan	56·2 (24·3 to 171)	72·3 (36·0 to 246)	28·8 (-11·7 to 80·0)	0·0485 (0·0211 to 0·152)	0·0356 (0·0175 to 0·123)	-26·9 (-48·1 to 2·0)

Palau	0·0598 (0·0322 to 0·0872)	0·0690 (0·0458 to 0·0924)	15·3 (-19·8 to 67·8)	0·347 (0·195 to 0·503)	0·316 (0·205 to 0·424)	-9·2 (-35·5 to 34·0)
Palestine	4·07 (2·94 to 5·28)	4·80 (3·34 to 6·31)	17·9 (-8·9 to 66·9)	0·179 (0·129 to 0·232)	0·118 (0·0807 to 0·149)	-34·7 (-47·4 to -11·6)
Panama	1·94 (1·75 to 2·08)	2·40 (1·95 to 2·92)	23·9 (1·4 to 54·7)	0·0738 (0·0665 to 0·0798)	0·0571 (0·0464 to 0·0692)	-22·2 (-36·9 to -2·7)
Papua New Guinea	12·5 (4·88 to 23·3)	18·9 (8·38 to 36·9)	51·8 (-11·8 to 154·8)	0·257 (0·0954 to 0·484)	0·204 (0·0805 to 0·436)	-20·9 (-49·2 to 23·1)
Paraguay	2·93 (2·26 to 5·08)	3·82 (2·70 to 6·43)	30·5 (-4·1 to 81·6)	0·0651 (0·0502 to 0·111)	0·0569 (0·0410 to 0·0958)	-13·7 (-36·6 to 17·8)
Peru	6·86 (1·59 to 9·74)	9·57 (1·81 to 15·0)	39·4 (-1·6 to 102·0)	0·0293 (0·00798 to 0·0413)	0·0269 (0·00550 to 0·0422)	-8·6 (-37·8 to 35·5)
Philippines	22·1 (8·88 to 27·7)	28·7 (12·1 to 38·0)	30·0 (2·6 to 60·2)	0·0316 (0·0125 to 0·0401)	0·0274 (0·0113 to 0·0360)	-13·2 (-30·4 to 5·3)
Poland	366 (355 to 372)	148 (137 to 159)	-59·5 (-62·7 to -56·5)	0·855 (0·831 to 0·870)	0·287 (0·266 to 0·307)	-66·3 (-68·8 to -63·8)
Portugal	45·9 (42·7 to 48·2)	33·7 (30·6 to 35·6)	-26·6 (-30·7 to -21·7)	0·383 (0·358 to 0·401)	0·202 (0·186 to 0·213)	-46·9 (-50·3 to -43·4)
Puerto Rico	8·69 (8·19 to 9·14)	0·895 (0·749 to 1·03)	-89·7 (-91·3 to -88·2)	0·223 (0·209 to 0·236)	0·0214 (0·0181 to 0·0247)	-90·3 (-91·7 to -88·9)
Qatar	4·47 (3·46 to 5·82)	9·56 (6·72 to 13·3)	113·8 (50·5 to 204·2)	1·03 (0·824 to 1·31)	0·352 (0·257 to 0·479)	-65·8 (-73·9 to -54·5)
Romania	603 (578 to 627)	222 (199 to 248)	-63·2 (-67·0 to -59·2)	2·59 (2·48 to 2·72)	0·823 (0·731 to 0·922)	-67·5 (-70·9 to -64·1)
Russia	12100 (12000 to 12200)	4300 (4030 to 4570)	-64·6 (-66·9 to -62·1)	7·26 (7·20 to 7·32)	2·36 (2·22 to 2·51)	-67·3 (-69·3 to -65·3)
Rwanda	6·19 (2·45 to 20·4)	5·47 (1·20 to 24·0)	-11·7 (-70·0 to 82·5)	0·122 (0·0484 to 0·425)	0·0642 (0·0135 to 0·301)	-47·5 (-80·1 to -8·4)
Saint Kitts and Nevis	0·0176 (0·0152 to 0·0198)	0·0118 (0·00931 to 0·0144)	-33·1 (-47·3 to -13·5)	0·0420 (0·0361 to 0·0480)	0·0215 (0·0166 to 0·0265)	-49·6 (-61·1 to -34·8)
Saint Lucia	0·260 (0·243 to 0·278)	0·229 (0·185 to 0·269)	-11·9 (-28·4 to 6·0)	0·198 (0·184 to 0·212)	0·124 (0·0986 to 0·148)	-35·9 (-48·1 to -23·2)
Saint Vincent and the Grenadines	0·0471 (0·0425 to 0·0513)	0·0413 (0·0356 to 0·0473)	-12·5 (-25·8 to 1·6)	0·0524 (0·0467 to 0·0575)	0·0382 (0·0324 to 0·0442)	-25·8 (-37·1 to -13·1)
Samoa	0·298 (0·136 to 0·485)	0·274 (0·148 to 0·438)	-8·2 (-32·2 to 29·7)	0·193 (0·0915 to 0·318)	0·143 (0·0798 to 0·229)	-25·6 (-43·5 to 0·1)
San Marino	0·0106 (0·00410 to 0·0148)	0·00919 (0·00369 to 0·0155)	-13·0 (-48·1 to 29·6)	0·0294 (0·0126 to 0·0401)	0·0173 (0·00895 to 0·0269)	-40·9 (-62·2 to -14·8)
Saudi Arabia	92·1 (70·6 to 123)	166 (119 to 226)	80·0 (8·6 to 163·4)	0·559 (0·447 to 0·737)	0·428 (0·317 to 0·564)	-23·0 (-49·3 to 7·6)
Senegal	3·07 (1·29 to 5·76)	4·61 (1·34 to 12·7)	50·2 (-23·4 to 146·0)	0·0585 (0·0250 to 0·111)	0·0484 (0·0144 to 0·134)	-17·4 (-57·1 to 37·9)
Serbia	48·3 (33·0 to 57·7)	24·1 (19·7 to 29·2)	-50·1 (-62·1 to -32·5)	0·465 (0·317 to 0·553)	0·187 (0·150 to 0·227)	-59·5 (-69·1 to -43·6)
Seychelles	0·198 (0·127 to 0·241)	0·137 (0·103 to 0·185)	-30·9 (-47·2 to -1·1)	0·240 (0·154 to 0·296)	0·121 (0·0884 to 0·158)	-49·9 (-61·7 to -28·7)
Sierra Leone	1·49 (0·756 to 3·56)	2·20 (0·976 to 5·81)	47·3 (-21·3 to 170·9)	0·0587 (0·0296 to 0·141)	0·0450 (0·0197 to 0·118)	-23·4 (-58·6 to 45·2)
Singapore	1·18 (1·08 to 1·29)	0·861 (0·773 to 0·929)	-27·2 (-35·2 to -19·8)	0·0271 (0·0250 to 0·0293)	0·0131 (0·0119 to 0·0140)	-51·7 (-57·0 to -47·0)
Slovakia	30·1 (23·4 to 35·5)	18·7 (15·1 to 23·3)	-38·0 (-50·1 to -22·1)	0·513 (0·400 to 0·601)	0·264 (0·212 to 0·334)	-48·6 (-58·8 to -34·5)
Slovenia	8·64 (8·03 to 9·15)	2·96 (2·53 to 3·34)	-65·7 (-70·6 to -61·0)	0·361 (0·335 to 0·385)	0·0876 (0·0752 to 0·0983)	-75·6 (-79·0 to -72·2)
Solomon Islands	1·10 (0·419 to 1·93)	1·34 (0·539 to 2·68)	22·6 (-23·7 to 85·8)	0·294 (0·110 to 0·484)	0·226 (0·0949 to 0·453)	-23·0 (-47·7 to 15·3)

Somalia	9·82 (2·18 to 51·2)	14·6 (2·10 to 80·6)	48·7 (-42·6 to 148·0)	0·160 (0·0353 to 0·788)	0·126 (0·0156 to 0·762)	-21·6 (-69·2 to 11·9)
South Africa	80·4 (50·0 to 118)	54·0 (32·7 to 111)	-32·8 (-55·3 to 10·7)	0·178 (0·112 to 0·262)	0·0962 (0·0588 to 0·196)	-47·3 (-64·6 to -13·1)
South Korea	198 (167 to 305)	127 (103 to 213)	-36·1 (-51·8 to -23·3)	0·423 (0·362 to 0·636)	0·172 (0·139 to 0·307)	-59·2 (-69·9 to -49·9)
South Sudan	4·56 (1·25 to 16·4)	5·51 (1·12 to 26·8)	20·8 (-54·2 to 148·4)	0·111 (0·0296 to 0·430)	0·0926 (0·0192 to 0·430)	-16·0 (-63·7 to 57·7)
Spain	77·6 (73·8 to 81·5)	56·6 (51·2 to 59·9)	-27·0 (-30·9 to -21·8)	0·156 (0·151 to 0·164)	0·0839 (0·0783 to 0·0882)	-46·3 (-48·8 to -42·9)
Sri Lanka	22·1 (11·6 to 28·3)	10·6 (7·00 to 18·9)	-52·1 (-70·2 to 2·2)	0·120 (0·0637 to 0·152)	0·0473 (0·0318 to 0·0835)	-60·7 (-75·3 to -19·0)
Sudan	274 (94·4 to 454)	296 (106 to 482)	8·1 (-33·5 to 83·9)	1·14 (0·422 to 1·86)	0·802 (0·294 to 1·31)	-29·8 (-51·4 to 9·7)
Suriname	0·0707 (0·0286 to 0·103)	0·0968 (0·0364 to 0·154)	36·8 (-10·5 to 91·0)	0·0197 (0·00728 to 0·0303)	0·0196 (0·00684 to 0·0319)	-1·1 (-34·9 to 40·4)
Sweden	11·9 (11·4 to 12·4)	13·3 (12·0 to 14·8)	11·1 (0·2 to 24·8)	0·115 (0·109 to 0·119)	0·105 (0·0962 to 0·117)	-8·2 (-17·9 to 3·6)
Switzerland	3·18 (2·99 to 3·35)	2·05 (1·88 to 2·20)	-35·5 (-38·7 to -31·4)	0·0357 (0·0341 to 0·0376)	0·0160 (0·0151 to 0·0170)	-55·1 (-57·7 to -52·0)
Syria	47·1 (39·0 to 56·0)	38·7 (27·2 to 53·2)	-17·9 (-44·6 to 13·0)	0·351 (0·292 to 0·418)	0·307 (0·215 to 0·422)	-14·2 (-41·5 to 19·9)
São Tomé and Príncipe	0·317 (0·213 to 0·487)	0·434 (0·265 to 0·683)	36·9 (-17·6 to 144·5)	0·336 (0·222 to 0·511)	0·276 (0·164 to 0·441)	-18·1 (-47·8 to 46·5)
Taiwan (province of China)	86·9 (81·5 to 92·5)	85·6 (76·7 to 94·2)	-1·5 (-12·8 to 8·0)	0·378 (0·355 to 0·403)	0·264 (0·239 to 0·288)	-30·2 (-36·5 to -24·0)
Tajikistan	141 (80·9 to 184)	108 (65·7 to 196)	-23·5 (-49·3 to 21·6)	2·73 (1·55 to 3·54)	1·25 (0·795 to 2·14)	-56·2 (-69·7 to -35·7)
Tanzania	24·1 (9·10 to 90·5)	23·0 (5·67 to 103)	-4·8 (-68·7 to 62·5)	0·101 (0·0353 to 0·368)	0·0617 (0·0145 to 0·283)	-39·1 (-74·5 to -5·9)
Thailand	37·7 (19·6 to 54·1)	34·1 (18·2 to 47·8)	-9·4 (-41·0 to 41·3)	0·0568 (0·0313 to 0·0796)	0·0470 (0·0242 to 0·0655)	-17·8 (-43·8 to 20·7)
The Bahamas	0·493 (0·432 to 0·548)	0·382 (0·302 to 0·472)	-22·5 (-37·7 to -0·9)	0·174 (0·153 to 0·194)	0·103 (0·0800 to 0·128)	-40·8 (-52·4 to -24·0)
The Gambia	0·537 (0·217 to 1·31)	0·781 (0·338 to 2·38)	45·3 (-21·5 to 133·8)	0·0851 (0·0351 to 0·212)	0·0640 (0·0285 to 0·193)	-24·8 (-57·4 to 25·1)
Timor-Leste	0·543 (0·295 to 1·02)	0·774 (0·436 to 1·63)	42·6 (-16·7 to 123·4)	0·0738 (0·0375 to 0·142)	0·0658 (0·0371 to 0·138)	-11·2 (-46·2 to 36·6)
Togo	1·68 (0·944 to 3·64)	2·89 (1·25 to 8·18)	72·7 (-13·1 to 171·1)	0·0699 (0·0390 to 0·152)	0·0546 (0·0235 to 0·155)	-21·9 (-61·5 to 22·6)
Tokelau	0·00344 (0·00138 to 0·00670)	0·00205 (0·000844 to 0·00454)	-40·4 (-58·3 to -16·0)	0·232 (0·0933 to 0·454)	0·145 (0·0592 to 0·317)	-37·6 (-55·3 to -13·2)
Tonga	0·596 (0·377 to 0·803)	0·526 (0·341 to 0·769)	-11·8 (-35·3 to 20·9)	0·698 (0·431 to 0·899)	0·554 (0·359 to 0·803)	-20·7 (-40·9 to 9·4)
Trinidad and Tobago	0·809 (0·749 to 0·882)	0·762 (0·611 to 0·966)	-5·9 (-26·0 to 21·1)	0·0791 (0·0725 to 0·0880)	0·0530 (0·0415 to 0·0676)	-31·5 (-46·1 to -11·9)
Tunisia	60·1 (23·7 to 95·1)	54·3 (19·8 to 76·6)	-9·6 (-37·1 to 27·3)	0·671 (0·270 to 1·04)	0·440 (0·167 to 0·634)	-34·3 (-53·1 to -8·1)
Turkmenistan	81·3 (74·1 to 91·4)	17·8 (14·3 to 21·5)	-78·2 (-82·4 to -73·3)	2·38 (2·15 to 2·69)	0·360 (0·291 to 0·433)	-84·5 (-87·6 to -81·0)
Tuvalu	0·0240 (0·0125 to 0·0378)	0·0218 (0·0125 to 0·0364)	-9·5 (-41·3 to 30·3)	0·260 (0·135 to 0·415)	0·181 (0·104 to 0·300)	-30·6 (-53·1 to -3·4)
Türkiye	284 (217 to 494)	345 (245 to 439)	21·4 (-39·3 to 76·5)	0·468 (0·361 to 0·792)	0·413 (0·298 to 0·529)	-12·6 (-54·5 to 26·8)
UK	107 (104 to 109)	56·0 (53·9 to 57·5)	-47·8 (-49·5 to -46·5)	0·162 (0·158 to 0·164)	0·0689 (0·0669 to 0·0706)	-57·4 (-58·8 to -56·3)

USA	738 (715 to 753)	1210 (1170 to 1250)	64·5 (57·2 to 69·7)	0·241 (0·235 to 0·246)	0·325 (0·314 to 0·336)	35·3 (28·9 to 39·8)
Uganda	17·2 (5·13 to 63·3)	19·7 (5·49 to 74·1)	14·2 (-56·2 to 135·3)	0·126 (0·0375 to 0·466)	0·0789 (0·0210 to 0·320)	-37·0 (-72·7 to 1·2)
Ukraine	3670 (3530 to 3820)	918 (657 to 1230)	-75·0 (-82·3 to -67·0)	6·35 (6·10 to 6·60)	1·59 (1·16 to 2·11)	-75·0 (-82·2 to -67·2)
United Arab Emirates	13·9 (9·26 to 17·9)	18·4 (12·6 to 24·8)	32·3 (-6·3 to 98·6)	0·547 (0·368 to 0·690)	0·260 (0·181 to 0·342)	-52·5 (-64·4 to -33·9)
Uruguay	15·1 (14·2 to 15·8)	16·0 (15·1 to 17·0)	6·0 (-2·4 to 15·8)	0·431 (0·407 to 0·454)	0·390 (0·366 to 0·416)	-8·9 (-16·0 to -0·8)
Uzbekistan	476 (429 to 528)	220 (181 to 253)	-53·8 (-63·8 to -44·5)	2·35 (2·11 to 2·59)	0·661 (0·551 to 0·759)	-71·9 (-77·8 to -66·3)
Vanuatu	0·450 (0·181 to 0·689)	0·570 (0·290 to 0·972)	26·7 (-11·0 to 92·4)	0·280 (0·116 to 0·453)	0·208 (0·110 to 0·349)	-25·8 (-45·1 to 4·4)
Venezuela	21·5 (19·7 to 23·3)	32·9 (24·0 to 44·8)	53·3 (12·3 to 102·3)	0·104 (0·0967 to 0·113)	0·128 (0·0939 to 0·175)	23·7 (-9·5 to 63·5)
Viet Nam	208 (59·9 to 309)	232 (69·5 to 353)	11·4 (-30·3 to 75·1)	0·299 (0·0798 to 0·440)	0·220 (0·0685 to 0·337)	-26·5 (-53·8 to 15·7)
Virgin Islands	0·284 (0·185 to 0·342)	0·180 (0·128 to 0·253)	-36·4 (-51·9 to -16·4)	0·282 (0·184 to 0·347)	0·202 (0·140 to 0·303)	-26·5 (-47·6 to 3·9)
Yemen	160 (47·7 to 272)	227 (69·9 to 402)	42·0 (-7·4 to 127·7)	1·10 (0·360 to 1·92)	0·884 (0·285 to 1·54)	-19·3 (-42·8 to 32·6)
Zambia	10·3 (3·51 to 35·9)	10·3 (2·71 to 41·9)	-0·4 (-64·0 to 80·3)	0·161 (0·0694 to 0·550)	0·0965 (0·0250 to 0·408)	-40·4 (-74·1 to -2·4)
Zimbabwe	44·6 (17·4 to 61·3)	79·3 (30·3 to 130)	77·7 (9·9 to 170·5)	0·384 (0·155 to 0·571)	0·507 (0·208 to 0·848)	29·7 (-19·4 to 105·9)

Estimates were computed at the draw level in order to propagate uncertainty; manual calculations (eg, for percentage change or grouped locations) may result in slight differences.

Table S8 Global and country-level years of life lost (YLLs) due to unintentional carbon monoxide poisoning and age-standardised rates of YLLs due to unintentional carbon monoxide poisoning in 2000 and 2021, for both sexes, and percentage change, 2000 to 2021, for each metric, with 95% UIs

	Number, all-ages (95% UI)			Rate per 100,000, age-standardised (95% UI)		
Location	2000	2021	Percentage change, 2000 to 2021	2000	2021	Percentage change, 2000 to 2021
Global	2110000 (1760000 to 2250000)	1180000 (886000 to 1350000)	-43·9 (-55·1 to -36·7)	35·8 (29·9 to 38·1)	14·9 (11·1 to 17·0)	-58·1 (-66·4 to -52·8)
Afghanistan	20100 (6880 to 37300)	27600 (9160 to 42700)	36·9 (-11·7 to 108·1)	127 (42·7 to 217)	91·5 (30·8 to 141)	-28·0 (-50·1 to 7·8)
Albania	491 (309 to 625)	163 (113 to 224)	-66·9 (-78·5 to -51·9)	17·1 (10·9 to 22·2)	7·17 (4·76 to 11·6)	-59·2 (-74·4 to -39·7)
Algeria	12400 (4930 to 19200)	10200 (3800 to 15600)	-18·3 (-38·9 to 13·4)	39·7 (15·6 to 61·2)	23·2 (8·79 to 35·6)	-41·4 (-55·5 to -20·9)
American Samoa	3·70 (2·13 to 4·73)	2·51 (1·73 to 3·40)	-32·2 (-58·3 to -0·7)	6·55 (3·76 to 8·38)	4·93 (3·33 to 6·55)	-23·8 (-51·7 to 12·0)
Andorra	0·00251 (0·00153 to 0·00387)	0·00291 (0·00107 to 0·00476)	15·9 (-45·3 to 120·3)	0·00363 (0·00220 to 0·00563)	0·00323 (0·00129 to 0·00524)	-10·0 (-53·4 to 86·5)
Angola	829 (224 to 3240)	718 (177 to 2700)	-13·3 (-73·6 to 76·0)	5·11 (1·82 to 17·5)	2·70 (0·725 to 9·60)	-48·8 (-80·3 to -15·6)
Antigua and Barbuda	11·0 (9·88 to 12·3)	5·77 (5·16 to 6·32)	-47·5 (-54·2 to -42·1)	16·6 (14·7 to 19·0)	7·60 (6·63 to 8·64)	-53·5 (-59·5 to -48·0)
Argentina	9900 (9560 to 10200)	8490 (8050 to 8950)	-14·2 (-19·2 to -9·5)	28·3 (27·1 to 29·3)	19·9 (18·6 to 21·1)	-29·3 (-33·7 to -24·9)
Armenia	2050 (1690 to 2450)	557 (474 to 640)	-72·8 (-79·4 to -64·2)	62·3 (50·8 to 74·7)	16·6 (14·1 to 19·0)	-73·2 (-79·8 to -64·6)
Australia	1850 (1760 to 1950)	1050 (1000 to 1110)	-43·4 (-47·5 to -37·9)	10·1 (9·61 to 10·7)	4·22 (4·03 to 4·47)	-57·7 (-60·9 to -53·5)
Austria	586 (556 to 613)	582 (552 to 614)	-0·7 (-7·6 to 6·8)	7·29 (6·91 to 7·68)	6·41 (6·07 to 6·82)	-11·1 (-18·1 to -4·5)
Azerbaijan	5140 (2830 to 6670)	2680 (1870 to 3740)	-47·9 (-67·2 to -13·0)	66·2 (36·1 to 86·2)	24·4 (17·2 to 33·7)	-63·0 (-75·7 to -38·3)
Bahrain	85·8 (72·4 to 108)	125 (90·3 to 158)	46·1 (11·7 to 87·4)	13·3 (11·3 to 16·6)	8·13 (5·99 to 10·2)	-39·5 (-52·6 to -22·8)
Bangladesh	7450 (970 to 16700)	4660 (777 to 9180)	-37·5 (-68·1 to 35·3)	4·24 (0·748 to 8·83)	2·86 (0·457 to 5·70)	-34·1 (-65·0 to 20·8)
Barbados	2·84 (2·52 to 3·26)	1·67 (1·22 to 2·34)	-41·1 (-56·3 to -15·2)	1·21 (1·05 to 1·40)	0·639 (0·455 to 0·948)	-48·6 (-61·9 to -24·4)
Belarus	31000 (29300 to 33200)	6400 (5190 to 7940)	-79·4 (-83·3 to -74·0)	290 (273 to 312)	59·8 (48·6 to 74·0)	-79·3 (-83·3 to -74·0)
Belgium	1610 (1500 to 1690)	889 (844 to 931)	-44·9 (-48·3 to -40·4)	15·8 (14·7 to 16·6)	7·25 (6·89 to 7·65)	-53·7 (-56·7 to -49·5)
Belize	53·1 (48·1 to 57·4)	39·9 (34·7 to 45·7)	-24·8 (-35·4 to -11·8)	24·0 (21·8 to 26·0)	10·8 (9·27 to 12·5)	-53·5 (-60·2 to -45·7)
Benin	87·9 (38·4 to 175)	122 (50·0 to 320)	39·0 (-25·4 to 132·0)	2·29 (0·930 to 4·67)	1·49 (0·620 to 3·81)	-35·2 (-66·5 to 21·6)
Bermuda	6·63 (5·95 to 7·27)	2·06 (1·72 to 2·45)	-68·9 (-73·7 to -62·1)	11·2 (9·95 to 12·4)	3·70 (3·02 to 4·53)	-67·3 (-73·1 to -58·8)
Bhutan	19·9 (7·42 to 77·9)	12·9 (4·78 to 59·9)	-34·9 (-62·6 to 3·0)	2·89 (1·07 to 11·4)	1·71 (0·632 to 7·69)	-41·2 (-65·6 to -7·9)
Bolivia	346 (67·1 to 556)	374 (41·6 to 648)	8·1 (-47·7 to 95·2)	3·85 (0·767 to 5·97)	3·18 (0·390 to 5·49)	-17·6 (-57·6 to 45·7)

Bosnia and Herzegovina	1930 (1210 to 2790)	920 (662 to 1530)	-52·3 (-65·9 to -29·2)	53·5 (33·1 to 76·1)	26·9 (18·7 to 45·0)	-49·2 (-63·5 to -22·2)
Botswana	178 (81·4 to 316)	108 (46·2 to 253)	-39·4 (-68·6 to 22·2)	9·71 (4·17 to 17·7)	4·37 (1·98 to 10·6)	-55·8 (-77·9 to -5·8)
Brazil	3180 (3050 to 3320)	2980 (2830 to 3100)	-6·4 (-11·6 to -1·2)	1·89 (1·80 to 1·98)	1·36 (1·29 to 1·42)	-26·5 (-30·5 to -22·3)
Brunei	31·1 (25·6 to 38·6)	27·8 (22·6 to 35·3)	-10·7 (-29·2 to 12·4)	9·33 (7·69 to 11·5)	5·76 (4·70 to 7·14)	-38·4 (-50·4 to -24·1)
Bulgaria	3010 (2760 to 3310)	946 (818 to 1080)	-68·6 (-73·5 to -62·6)	40·2 (36·5 to 44·2)	12·8 (11·1 to 14·8)	-67·3 (-72·6 to -61·8)
Burkina Faso	174 (72·1 to 434)	211 (75·7 to 693)	21·6 (-44·4 to 97·6)	2·31 (0·866 to 5·64)	1·58 (0·569 to 5·12)	-31·8 (-67·4 to 13·7)
Burundi	260 (106 to 826)	309 (75·0 to 1200)	18·6 (-64·5 to 100·3)	4·67 (1·89 to 14·4)	3·12 (0·755 to 12·6)	-33·6 (-76·8 to 7·8)
Cabo Verde	1·87 (0·316 to 2·90)	4·45 (0·562 to 7·26)	137·7 (41·9 to 322·2)	0·708 (0·122 to 1·18)	0·822 (0·106 to 1·34)	16·1 (-35·9 to 106·1)
Cambodia	588 (256 to 1090)	642 (360 to 1240)	9·1 (-34·5 to 80·9)	4·90 (2·22 to 8·80)	3·72 (2·08 to 7·26)	-24·1 (-52·1 to 21·0)
Cameroon	275 (159 to 591)	419 (189 to 1100)	52·5 (-23·4 to 154·8)	3·02 (1·69 to 6·47)	2·08 (0·934 to 5·27)	-31·1 (-66·2 to 21·2)
Canada	3680 (3480 to 3870)	3810 (3540 to 4050)	3·5 (-4·3 to 12·3)	11·7 (11·1 to 12·4)	10·4 (9·57 to 11·2)	-11·3 (-18·9 to -3·8)
Central African Republic	193 (45·9 to 864)	215 (32·8 to 1070)	11·4 (-65·1 to 98·7)	5·65 (1·54 to 23·1)	4·42 (0·620 to 21·3)	-22·6 (-73·5 to 24·5)
Chad	130 (56·1 to 312)	194 (82·4 to 532)	49·8 (-25·1 to 146·5)	2·73 (1·16 to 6·18)	2·07 (0·865 to 5·52)	-24·3 (-60·3 to 22·3)
Chile	1910 (1810 to 2010)	1530 (1450 to 1630)	-19·7 (-24·9 to -14·1)	13·0 (12·2 to 13·6)	8·31 (7·84 to 8·84)	-36·4 (-40·8 to -31·7)
China	748000 (542000 to 852000)	491000 (279000 to 595000)	-34·3 (-60·5 to -15·5)	72·0 (47·9 to 82·7)	36·1 (20·6 to 43·0)	-48·9 (-67·2 to -36·6)
Colombia	2090 (1960 to 2240)	1670 (1370 to 1980)	-20·5 (-35·2 to -3·3)	5·19 (4·88 to 5·56)	3·38 (2·77 to 4·05)	-34·9 (-46·9 to -21·0)
Comoros	16·7 (5·87 to 56·1)	17·6 (4·05 to 80·0)	5·1 (-64·5 to 90·6)	3·24 (1·09 to 10·2)	2·60 (0·601 to 12·0)	-20·2 (-71·2 to 34·2)
Congo (Brazzaville)	138 (38·0 to 508)	136 (27·8 to 527)	-1·3 (-64·0 to 93·0)	4·72 (1·47 to 16·4)	2·98 (0·625 to 11·3)	-37·4 (-76·6 to 8·3)
Cook Islands	0·581 (0·294 to 0·795)	0·205 (0·127 to 0·317)	-64·7 (-78·5 to -30·4)	3·14 (1·60 to 4·27)	1·07 (0·640 to 1·70)	-66·1 (-79·4 to -36·0)
Costa Rica	81·0 (71·6 to 89·5)	95·4 (83·3 to 107)	17·8 (-0·2 to 39·1)	2·16 (1·94 to 2·36)	1·96 (1·72 to 2·20)	-8·6 (-22·1 to 8·0)
Croatia	907 (824 to 1000)	287 (253 to 321)	-68·4 (-72·6 to -63·2)	19·5 (17·9 to 21·4)	5·74 (5·04 to 6·47)	-70·3 (-74·2 to -65·5)
Cuba	329 (311 to 351)	227 (201 to 252)	-31·0 (-39·6 to -21·0)	3·47 (3·26 to 3·73)	2·16 (1·86 to 2·41)	-35·2 (-43·5 to -25·6)
Cyprus	134 (108 to 164)	82·0 (63·4 to 101)	-38·6 (-51·5 to -22·7)	14·3 (11·6 to 17·6)	5·48 (4·14 to 6·82)	-61·8 (-70·0 to -51·9)
Czechia	4450 (4160 to 4680)	1310 (1150 to 1500)	-70·4 (-74·0 to -66·3)	42·3 (39·8 to 44·5)	10·8 (9·55 to 12·2)	-74·2 (-77·2 to -71·0)
Côte d'Ivoire	277 (123 to 535)	332 (144 to 730)	19·9 (-34·4 to 88·3)	2·85 (1·21 to 5·36)	1·83 (0·813 to 3·95)	-35·7 (-66·4 to 1·5)
DR Congo	2360 (527 to 9140)	2610 (401 to 11200)	10·6 (-66·8 to 110·6)	4·67 (1·26 to 19·1)	3·62 (0·571 to 15·9)	-23·4 (-73·3 to 24·2)
Denmark	144 (135 to 152)	76·4 (71·3 to 80·6)	-47·1 (-51·8 to -42·3)	2·59 (2·43 to 2·74)	1·18 (1·10 to 1·26)	-54·3 (-58·1 to -50·4)
Djibouti	20·3 (4·81 to 71·5)	26·3 (5·40 to 101)	29·6 (-53·2 to 139·7)	3·71 (0·917 to 13·0)	2·44 (0·497 to 9·15)	-34·5 (-75·1 to 8·2)

Dominica	2·49 (0·952 to 3·52)	1·96 (0·827 to 2·93)	-21·3 (-48·0 to 20·4)	4·66 (1·67 to 7·38)	4·47 (1·69 to 7·98)	-6·5 (-41·4 to 49·1)
Dominican Republic	334 (183 to 453)	435 (178 to 659)	30·3 (-13·3 to 106·6)	4·13 (2·17 to 6·06)	4·50 (1·74 to 8·06)	11·5 (-29·5 to 82·0)
Ecuador	867 (782 to 956)	1510 (1240 to 1810)	73·7 (41·3 to 110·7)	6·52 (5·87 to 7·10)	8·20 (6·80 to 9·81)	25·1 (1·1 to 52·4)
Egypt	5470 (2580 to 8890)	4810 (2940 to 6350)	-12·2 (-51·2 to 42·3)	7·65 (3·98 to 11·8)	4·74 (2·92 to 6·23)	-39·0 (-64·3 to -10·3)
El Salvador	230 (127 to 329)	199 (117 to 309)	-13·8 (-39·6 to 22·3)	3·91 (2·18 to 5·60)	3·11 (1·86 to 4·93)	-20·4 (-44·8 to 12·7)
Equatorial Guinea	25·1 (6·87 to 81·5)	25·2 (5·17 to 88·1)	0·4 (-60·1 to 172·5)	3·81 (1·38 to 12·9)	2·02 (0·459 to 7·41)	-47·5 (-77·8 to 9·1)
Eritrea	155 (58·4 to 553)	193 (54·9 to 692)	24·4 (-54·5 to 130·0)	4·45 (1·85 to 14·5)	3·38 (0·990 to 12·3)	-24·5 (-69·6 to 25·1)
Estonia	1430 (1330 to 1520)	338 (295 to 377)	-76·3 (-79·4 to -73·7)	96·9 (90·6 to 103)	21·7 (19·0 to 24·3)	-77·2 (-80·1 to -74·8)
Eswatini	126 (71·8 to 215)	88·4 (38·4 to 210)	-29·7 (-68·9 to 27·7)	11·3 (6·40 to 18·7)	7·15 (3·11 to 17·1)	-36·9 (-71·7 to 10·6)
Ethiopia	3490 (887 to 12800)	2420 (550 to 11900)	-30·7 (-74·5 to 41·8)	6·20 (1·49 to 23·1)	2·93 (0·661 to 14·3)	-53·1 (-79·4 to -19·6)
Federated States of Micronesia	15·0 (7·58 to 24·9)	10·3 (5·06 to 17·4)	-31·8 (-60·6 to 10·7)	13·0 (6·63 to 21·2)	9·50 (4·83 to 15·7)	-27·3 (-56·5 to 7·2)
Fiji	45·4 (32·4 to 60·8)	34·8 (24·2 to 49·1)	-23·4 (-50·7 to 13·1)	5·46 (3·98 to 7·26)	3·71 (2·59 to 5·23)	-32·4 (-55·6 to -3·1)
Finland	1510 (1450 to 1570)	771 (726 to 820)	-48·9 (-52·6 to -45·5)	28·9 (27·7 to 30·3)	13·9 (12·9 to 14·8)	-52·0 (-55·7 to -48·3)
France	3900 (3740 to 4070)	2290 (2130 to 2400)	-41·3 (-44·9 to -37·8)	6·27 (5·99 to 6·57)	3·32 (3·14 to 3·49)	-47·1 (-50·3 to -43·9)
Gabon	40·3 (11·7 to 122)	37·9 (8·27 to 134)	-5·8 (-61·6 to 70·7)	3·71 (1·15 to 11·2)	2·44 (0·587 to 8·88)	-34·5 (-72·4 to 16·1)
Georgia	4960 (4420 to 5590)	1070 (935 to 1210)	-78·4 (-81·2 to -74·0)	101 (89·5 to 114)	27·3 (23·8 to 31·0)	-73·3 (-76·8 to -68·1)
Germany	5300 (5100 to 5490)	3840 (3620 to 4030)	-27·5 (-32·2 to -23·0)	6·57 (6·33 to 6·81)	4·22 (3·97 to 4·42)	-35·3 (-39·5 to -30·8)
Ghana	213 (106 to 308)	436 (180 to 774)	104·8 (18·9 to 235·7)	1·70 (0·859 to 2·60)	1·76 (0·763 to 3·11)	3·6 (-41·7 to 80·5)
Greece	544 (499 to 582)	368 (344 to 392)	-32·4 (-38·4 to -24·4)	4·64 (4·24 to 5·00)	3·45 (3·23 to 3·65)	-25·8 (-33·2 to -17·0)
Greenland	24·4 (21·1 to 29·2)	15·5 (12·5 to 19·6)	-36·5 (-51·4 to -22·5)	41·9 (36·2 to 50·7)	27·6 (22·0 to 34·2)	-34·9 (-49·5 to -22·4)
Grenada	5·85 (5·13 to 6·65)	1·26 (1·07 to 1·44)	-78·5 (-82·0 to -75·0)	6·68 (5·73 to 7·65)	1·52 (1·29 to 1·78)	-76·6 (-80·3 to -72·7)
Guam	7·76 (5·40 to 9·45)	4·91 (3·75 to 7·05)	-36·7 (-54·1 to 1·5)	4·90 (3·44 to 6·01)	3·14 (2·39 to 4·49)	-36·0 (-54·2 to 1·7)
Guatemala	658 (591 to 738)	591 (479 to 698)	-10·3 (-30·8 to 13·0)	6·05 (5·47 to 6·84)	3·75 (3·07 to 4·42)	-37·5 (-51·5 to -21·5)
Guinea	129 (53·5 to 270)	140 (54·6 to 364)	8·4 (-43·3 to 79·9)	2·40 (0·970 to 5·24)	1·73 (0·630 to 4·37)	-28·1 (-62·7 to 25·4)
Guinea-Bissau	24·1 (10·9 to 54·1)	29·4 (13·8 to 79·7)	22·4 (-42·5 to 106·1)	3·37 (1·51 to 7·18)	2·42 (1·13 to 6·38)	-28·2 (-66·4 to 29·7)
Guyana	86·7 (71·6 to 100)	55·6 (43·2 to 70·7)	-35·8 (-53·6 to -11·5)	10·6 (9·00 to 12·1)	7·30 (5·63 to 9·17)	-31·1 (-50·2 to -5·4)
Haiti	1250 (489 to 2540)	1420 (637 to 2650)	13·5 (-33·1 to 93·7)	15·6 (6·57 to 30·1)	11·9 (5·28 to 21·8)	-23·5 (-51·8 to 23·9)
Honduras	695 (280 to 1210)	741 (294 to 1330)	6·6 (-35·5 to 89·5)	10·8 (4·65 to 16·9)	7·66 (3·14 to 13·3)	-29·4 (-54·3 to 23·5)

Hungary	2700 (2580 to 2820)	1000 (878 to 1120)	-63·0 (-68·0 to -57·5)	26·1 (25·1 to 27·1)	9·14 (8·00 to 10·2)	-64·0 (-68·6 to -59·0)
Iceland	31·0 (29·0 to 33·2)	26·8 (24·3 to 29·4)	-13·5 (-22·3 to -3·8)	11·2 (10·4 to 12·0)	7·50 (6·82 to 8·24)	-32·1 (-38·7 to -24·1)
India	42800 (8970 to 63500)	38800 (9650 to 54900)	-9·3 (-30·7 to 26·9)	3·92 (0·874 to 5·76)	2·64 (0·671 to 3·76)	-32·8 (-47·7 to -6·5)
Indonesia	10600 (3730 to 17200)	12900 (4430 to 21500)	22·0 (-11·7 to 74·2)	4·68 (1·73 to 7·55)	4·38 (1·52 to 7·33)	-6·3 (-31·4 to 32·5)
Iran	40100 (22700 to 49500)	22600 (15800 to 26700)	-43·6 (-57·2 to -23·4)	62·6 (33·8 to 77·2)	25·3 (18·6 to 30·2)	-58·3 (-68·3 to -41·2)
Iraq	3820 (2240 to 5210)	3130 (2030 to 4450)	-18·1 (-45·4 to 20·5)	14·8 (8·77 to 20·4)	7·67 (5·03 to 10·7)	-48·4 (-65·0 to -24·8)
Ireland	264 (249 to 280)	152 (136 to 165)	-42·5 (-49·2 to -35·9)	6·65 (6·26 to 7·03)	2·94 (2·67 to 3·20)	-55·7 (-60·7 to -50·7)
Israel	122 (112 to 133)	105 (95·9 to 113)	-14·5 (-23·4 to -3·6)	1·95 (1·79 to 2·10)	1·11 (1·01 to 1·20)	-42·6 (-48·5 to -35·2)
Italy	3480 (3400 to 3570)	2170 (2030 to 2270)	-37·6 (-41·0 to -35·2)	5·80 (5·67 to 5·94)	3·18 (3·05 to 3·31)	-45·1 (-47·3 to -43·2)
Jamaica	39·0 (34·7 to 44·0)	32·4 (25·7 to 42·0)	-16·7 (-36·3 to 11·2)	1·74 (1·53 to 2·00)	1·34 (1·04 to 1·74)	-20·1 (-38·4 to 6·9)
Japan	9540 (9350 to 9730)	6630 (6430 to 6820)	-30·5 (-32·3 to -28·0)	6·89 (6·77 to 7·02)	5·46 (5·34 to 5·60)	-20·4 (-22·5 to -18·0)
Jordan	2530 (1750 to 3110)	2360 (1970 to 2920)	-6·7 (-28·5 to 22·0)	51·5 (35·6 to 62·8)	19·9 (16·5 to 25·0)	-61·3 (-70·4 to -51·0)
Kazakhstan	39500 (35900 to 43900)	14600 (13200 to 16300)	-63·0 (-66·6 to -57·2)	288 (260 to 321)	77·1 (69·8 to 85·6)	-72·5 (-75·0 to -68·2)
Kenya	1440 (583 to 5210)	1620 (327 to 6540)	12·4 (-49·4 to 59·1)	5·26 (1·89 to 18·3)	4·22 (0·835 to 17·3)	-19·8 (-61·9 to 19·9)
Kiribati	16·0 (7·01 to 21·5)	25·5 (7·33 to 40·2)	59·8 (-7·1 to 192·3)	17·7 (7·85 to 23·5)	20·3 (6·28 to 31·3)	14·4 (-32·5 to 92·7)
Kuwait	589 (560 to 620)	737 (627 to 874)	25·1 (5·9 to 44·7)	30·1 (28·5 to 31·8)	16·5 (14·1 to 19·6)	-45·4 (-52·8 to -36·8)
Kyrgyzstan	12100 (11100 to 13100)	3030 (2680 to 3490)	-75·0 (-78·7 to -70·6)	269 (245 to 291)	45·9 (40·7 to 52·9)	-83·0 (-85·5 to -79·8)
Laos	275 (144 to 561)	269 (146 to 541)	-2·0 (-46·5 to 69·3)	5·36 (2·95 to 10·0)	3·51 (1·92 to 7·16)	-34·6 (-62·0 to 6·0)
Latvia	2930 (2800 to 3060)	651 (581 to 712)	-77·8 (-80·4 to -75·2)	117 (112 to 122)	29·0 (25·9 to 31·7)	-75·1 (-78·0 to -72·4)
Lebanon	157 (91·1 to 207)	169 (110 to 213)	7·7 (-17·8 to 65·0)	4·47 (2·59 to 5·87)	3·01 (1·97 to 3·79)	-32·9 (-48·8 to 1·5)
Lesotho	198 (126 to 368)	174 (88·6 to 407)	-12·4 (-53·5 to 56·6)	10·7 (6·14 to 20·0)	8·80 (4·46 to 20·5)	-18·3 (-57·1 to 52·7)
Liberia	41·6 (22·8 to 103)	65·3 (27·0 to 178)	57·1 (-24·5 to 156·1)	2·42 (1·30 to 6·21)	1·86 (0·750 to 5·13)	-22·9 (-62·7 to 32·6)
Libya	1670 (752 to 2250)	2720 (1040 to 4220)	62·5 (14·2 to 129·9)	32·9 (14·3 to 43·3)	37·8 (14·9 to 58·8)	14·7 (-16·3 to 67·7)
Lithuania	6450 (6220 to 6740)	1800 (1600 to 1970)	-72·1 (-75·4 to -69·4)	178 (172 to 186)	54·4 (48·5 to 58·9)	-69·0 (-72·5 to -66·2)
Luxembourg	26·2 (24·7 to 27·7)	17·5 (16·1 to 19·3)	-33·2 (-38·9 to -25·0)	5·89 (5·58 to 6·24)	2·49 (2·26 to 2·76)	-57·8 (-61·8 to -52·7)
Madagascar	595 (198 to 2050)	624 (167 to 3030)	4·8 (-61·7 to 98·2)	3·84 (1·57 to 13·6)	2·56 (0·665 to 11·9)	-33·3 (-71·8 to 14·7)
Malawi	526 (170 to 2180)	449 (91·2 to 1890)	-14·7 (-73·3 to 74·4)	4·63 (1·72 to 17·3)	2·91 (0·622 to 12·4)	-37·5 (-77·9 to 6·4)
Malaysia	1730 (1220 to 2370)	1780 (1340 to 2770)	2·8 (-30·3 to 36·1)	7·30 (5·15 to 9·86)	5·27 (4·05 to 8·19)	-27·6 (-50·3 to -2·9)

Maldives	2·42 (1·42 to 4·10)	3·77 (2·00 to 10·9)	55·8 (-19·3 to 234·8)	0·892 (0·520 to 1·56)	0·606 (0·346 to 1·59)	-32·0 (-63·1 to 31·7)
Mali	162 (57·3 to 363)	223 (73·7 to 605)	37·7 (-30·2 to 129·4)	2·32 (0·804 to 5·03)	1·65 (0·535 to 4·50)	-28·9 (-63·2 to 28·1)
Malta	19·0 (17·7 to 20·4)	11·9 (10·9 to 13·0)	-37·6 (-45·0 to -29·0)	4·73 (4·37 to 5·07)	2·76 (2·45 to 3·09)	-41·2 (-48·9 to -31·1)
Marshall Islands	6·72 (2·89 to 12·0)	5·41 (2·40 to 9·92)	-19·5 (-49·1 to 32·0)	12·2 (5·45 to 21·1)	9·35 (4·18 to 16·8)	-23·4 (-48·4 to 18·5)
Mauritania	29·6 (14·6 to 60·3)	37·3 (15·5 to 99·9)	25·8 (-36·4 to 112·4)	1·79 (0·917 to 3·60)	1·30 (0·550 to 3·34)	-27·6 (-64·4 to 28·7)
Mauritius	167 (158 to 177)	101 (92·0 to 108)	-39·5 (-45·5 to -34·0)	13·7 (12·9 to 14·6)	7·78 (6·99 to 8·33)	-42·6 (-48·7 to -37·3)
Mexico	18400 (17600 to 19200)	14400 (13200 to 15700)	-21·4 (-28·8 to -14·5)	18·0 (17·2 to 18·8)	11·4 (10·4 to 12·5)	-36·4 (-42·2 to -30·8)
Moldova	10600 (10200 to 10900)	4780 (4230 to 5300)	-54·7 (-59·4 to -49·2)	258 (245 to 271)	112 (98·1 to 126)	-56·3 (-61·1 to -50·6)
Monaco	0·284 (0·146 to 0·445)	0·224 (0·126 to 0·318)	-21·2 (-54·1 to 20·4)	0·915 (0·546 to 1·44)	0·652 (0·366 to 0·958)	-28·5 (-61·1 to 19·9)
Mongolia	7440 (4950 to 10400)	3710 (2830 to 5050)	-50·1 (-66·8 to -17·4)	339 (226 to 480)	113 (85·3 to 151)	-67·4 (-77·8 to -46·7)
Montenegro	162 (127 to 189)	76·2 (58·6 to 104)	-53·1 (-63·2 to -34·0)	26·4 (20·8 to 31·2)	11·7 (8·97 to 16·8)	-55·8 (-65·4 to -35·2)
Morocco	14300 (4460 to 24000)	11000 (4190 to 18700)	-23·5 (-47·8 to 12·5)	47·3 (14·9 to 77·5)	29·9 (11·4 to 52·6)	-36·5 (-55·7 to -8·4)
Mozambique	740 (231 to 3390)	707 (174 to 3120)	-4·5 (-63·8 to 104·6)	3·99 (1·34 to 16·6)	2·80 (0·787 to 11·9)	-30·4 (-67·2 to 11·6)
Myanmar	7910 (2060 to 14000)	5370 (2040 to 7540)	-32·0 (-58·7 to 42·5)	17·1 (4·39 to 29·6)	9·45 (3·61 to 13·1)	-44·9 (-66·3 to 17·4)
Namibia	166 (97·1 to 300)	129 (58·5 to 291)	-22·3 (-57·5 to 54·4)	8·58 (4·91 to 15·1)	5·13 (2·32 to 11·8)	-41·1 (-67·7 to 13·7)
Nauru	1·62 (0·733 to 2·47)	1·15 (0·638 to 1·80)	-28·8 (-50·7 to 8·1)	14·9 (6·97 to 22·9)	10·1 (5·87 to 15·7)	-32·3 (-50·3 to -1·9)
Nepal	17700 (9270 to 32900)	15700 (8440 to 28800)	-11·2 (-41·3 to 40·1)	72·5 (39·7 to 133)	51·8 (28·1 to 90·0)	-28·8 (-51·0 to 10·2)
Netherlands	407 (390 to 428)	237 (224 to 250)	-41·9 (-45·6 to -38·3)	2·51 (2·39 to 2·64)	1·29 (1·21 to 1·35)	-48·4 (-52·1 to -44·7)
New Zealand	224 (212 to 239)	244 (233 to 258)	9·1 (0·1 to 17·8)	6·18 (5·84 to 6·64)	4·95 (4·71 to 5·28)	-19·8 (-26·6 to -13·6)
Nicaragua	174 (101 to 253)	129 (89·8 to 241)	-26·1 (-46·7 to 10·7)	3·46 (2·08 to 5·09)	1·99 (1·38 to 3·71)	-42·6 (-58·3 to -16·1)
Niger	160 (66·3 to 344)	198 (71·1 to 575)	23·4 (-26·5 to 99·1)	2·35 (0·886 to 5·13)	1·49 (0·553 to 4·22)	-36·5 (-61·7 to 2·5)
Nigeria	2040 (964 to 4380)	2090 (1030 to 6440)	2·4 (-48·9 to 91·5)	2·78 (1·30 to 5·84)	1·48 (0·737 to 4·23)	-46·9 (-73·7 to 0·9)
Niue	0·208 (0·121 to 0·333)	0·125 (0·0745 to 0·202)	-39·9 (-57·0 to -13·0)	10·6 (6·17 to 17·0)	7·45 (4·30 to 12·3)	-30·0 (-51·6 to 1·8)
North Korea	9010 (5810 to 16600)	9040 (5850 to 15400)	0·3 (-28·9 to 38·4)	39·6 (25·8 to 72·5)	33·7 (21·0 to 59·2)	-14·7 (-41·6 to 21·1)
North Macedonia	316 (245 to 392)	167 (123 to 232)	-47·1 (-58·2 to -33·8)	16·6 (12·7 to 20·2)	7·20 (5·35 to 10·2)	-55·0 (-64·8 to -44·5)
Northern Mariana Islands	7·78 (4·15 to 10·7)	7·28 (3·67 to 10·2)	-6·5 (-34·8 to 44·4)	11·7 (6·59 to 15·3)	14·1 (7·24 to 19·6)	19·9 (-16·2 to 75·3)
Norway	93·0 (90·5 to 96·6)	171 (163 to 178)	84·0 (74·8 to 91·2)	2·30 (2·24 to 2·40)	3·39 (3·21 to 3·53)	46·5 (38·4 to 52·0)
Oman	69·2 (46·3 to 90·1)	76·1 (52·1 to 101)	10·0 (-19·6 to 52·1)	3·17 (2·10 to 4·00)	1·62 (1·10 to 2·10)	-49·5 (-61·2 to -31·8)

Pakistan	3350 (1460 to 10000)	4390 (2190 to 14800)	31·0 (-11·1 to 90·2)	2·36 (1·02 to 7·14)	1·79 (0·885 to 6·14)	-24·7 (-48·4 to 8·7)
Palau	2·96 (1·59 to 4·32)	2·87 (1·80 to 3·93)	-2·9 (-30·4 to 47·0)	15·9 (8·84 to 23·3)	14·5 (9·00 to 20·5)	-9·0 (-38·8 to 34·1)
Palestine	247 (178 to 319)	269 (184 to 359)	8·8 (-18·1 to 56·4)	8·22 (5·89 to 10·9)	5·37 (3·71 to 7·10)	-35·5 (-50·0 to -8·5)
Panama	107 (96·4 to 117)	113 (91·6 to 138)	5·1 (-12·8 to 32·0)	3·81 (3·45 to 4·18)	2·79 (2·24 to 3·41)	-26·1 (-38·8 to -7·5)
Papua New Guinea	804 (333 to 1520)	1170 (568 to 2060)	46·0 (-19·9 to 166·5)	13·3 (5·15 to 24·7)	10·6 (4·60 to 20·1)	-20·7 (-53·9 to 31·4)
Paraguay	167 (130 to 295)	195 (134 to 339)	16·9 (-12·3 to 63·6)	3·31 (2·55 to 5·75)	2·80 (1·97 to 4·92)	-17·2 (-39·0 to 15·7)
Peru	403 (73·5 to 583)	455 (52·9 to 730)	12·9 (-28·9 to 63·3)	1·51 (0·293 to 2·20)	1·25 (0·158 to 1·98)	-17·9 (-47·2 to 18·9)
Philippines	1280 (523 to 1600)	1550 (651 to 2040)	21·2 (-4·6 to 51·9)	1·61 (0·670 to 2·03)	1·35 (0·589 to 1·78)	-16·2 (-33·9 to 3·8)
Poland	15500 (15200 to 15700)	5190 (4810 to 5560)	-66·5 (-69·1 to -64·0)	38·6 (37·9 to 39·1)	12·3 (11·5 to 13·2)	-67·8 (-70·0 to -65·6)
Portugal	1950 (1830 to 2060)	1000 (925 to 1060)	-48·5 (-52·2 to -44·9)	18·9 (17·8 to 19·8)	8·61 (7·97 to 9·09)	-54·1 (-57·3 to -50·8)
Puerto Rico	410 (387 to 436)	32·7 (27·6 to 37·8)	-92·0 (-93·1 to -90·9)	11·5 (10·8 to 12·3)	1·07 (0·914 to 1·24)	-90·5 (-91·8 to -89·1)
Qatar	248 (190 to 321)	511 (362 to 709)	106·3 (47·0 to 190·9)	44·0 (34·7 to 57·0)	15·8 (11·6 to 21·6)	-64·2 (-72·6 to -51·9)
Romania	25800 (24700 to 27000)	7160 (6340 to 8030)	-72·3 (-75·3 to -69·2)	129 (123 to 136)	34·5 (30·4 to 39·1)	-72·3 (-75·5 to -69·3)
Russia	540000 (537000 to 544000)	168000 (158000 to 178000)	-68·9 (-70·9 to -66·9)	352 (349 to 356)	107 (101 to 113)	-69·5 (-71·2 to -67·7)
Rwanda	361 (135 to 1230)	260 (57·4 to 1110)	-27·9 (-78·9 to 69·3)	4·77 (1·92 to 15·6)	2·34 (0·519 to 10·3)	-51·2 (-83·1 to -0·5)
Saint Kitts and Nevis	0·965 (0·808 to 1·10)	0·534 (0·406 to 0·672)	-44·6 (-59·8 to -24·5)	2·36 (1·96 to 2·77)	1·14 (0·837 to 1·45)	-53·1 (-66·2 to -37·0)
Saint Lucia	14·4 (13·3 to 15·6)	10·0 (8·00 to 11·9)	-30·4 (-44·7 to -14·3)	10·5 (9·53 to 11·4)	6·33 (4·94 to 7·82)	-37·9 (-51·2 to -22·4)
Saint Vincent and the Grenadines	2·46 (2·18 to 2·74)	1·76 (1·48 to 2·05)	-28·6 (-40·1 to -13·5)	2·59 (2·24 to 2·95)	1·81 (1·49 to 2·21)	-27·7 (-40·0 to -10·7)
Samoa	17·3 (7·70 to 28·4)	14·8 (7·63 to 24·1)	-14·4 (-40·3 to 29·2)	9·41 (4·23 to 15·3)	6·89 (3·63 to 11·0)	-26·8 (-46·7 to 5·0)
San Marino	0·368 (0·167 to 0·510)	0·251 (0·135 to 0·387)	-31·8 (-57·9 to -1·4)	1·34 (0·627 to 1·98)	0·746 (0·419 to 1·11)	-44·0 (-65·3 to -18·0)
Saudi Arabia	5190 (3920 to 7020)	8480 (6020 to 11800)	63·4 (-2·6 to 140·3)	26·0 (19·9 to 34·8)	19·2 (14·2 to 26·0)	-25·6 (-52·4 to 6·9)
Senegal	124 (49·4 to 228)	182 (52·7 to 507)	46·4 (-29·0 to 142·2)	2·01 (0·800 to 3·73)	1·69 (0·485 to 4·60)	-16·1 (-59·0 to 38·1)
Serbia	1940 (1340 to 2340)	736 (586 to 921)	-62·1 (-71·5 to -47·1)	20·7 (14·3 to 25·1)	7·21 (5·71 to 9·60)	-64·7 (-73·6 to -49·9)
Seychelles	9·95 (6·40 to 12·2)	6·18 (4·56 to 8·29)	-37·8 (-53·3 to -13·0)	11·5 (7·46 to 14·1)	5·50 (4·09 to 7·33)	-52·5 (-64·1 to -34·0)
Sierra Leone	60·6 (31·2 to 140)	90·0 (38·5 to 239)	48·4 (-24·2 to 167·8)	2·04 (1·00 to 4·70)	1·59 (0·694 to 4·20)	-22·1 (-59·1 to 40·9)
Singapore	63·1 (57·4 to 68·6)	35·5 (32·6 to 38·0)	-43·7 (-49·8 to -38·3)	1·42 (1·31 to 1·53)	0·635 (0·577 to 0·679)	-55·4 (-60·1 to -51·4)
Slovakia	1250 (984 to 1470)	651 (520 to 854)	-48·1 (-59·2 to -34·5)	23·4 (18·4 to 27·1)	11·4 (9·07 to 15·0)	-51·5 (-62·7 to -38·0)
Slovenia	333 (310 to 356)	82·7 (70·4 to 93·1)	-75·2 (-78·8 to -71·6)	15·7 (14·6 to 16·9)	3·32 (2·87 to 3·75)	-78·6 (-81·6 to -75·6)

Solomon Islands	67·2<br>(25·2 to 118)	78·0<br>(30·0 to 156)	15·9<br>(-32·7 to 75·8)	14·5<br>(5·46 to 25·5)	11·2<br>(4·47 to 22·4)	-23·0<br>(-51·8 to 15·8)
Somalia	575<br>(121 to 2920)	824<br>(123 to 4230)	43·2<br>(-46·6 to 167·6)	6·06<br>(1·39 to 30·8)	4·70<br>(0·636 to 27·1)	-23·3<br>(-69·3 to 22·9)
South Africa	5020<br>(3200 to 7320)	3210<br>(1960 to 6660)	-36·1<br>(-57·3 to 5·3)	10·6<br>(6·75 to 15·5)	5·77<br>(3·50 to 11·9)	-47·5<br>(-65·3 to -12·7)
South Korea	9310<br>(7870 to 14500)	4290<br>(3430 to 7770)	-53·9<br>(-66·3 to -44·5)	19·5<br>(16·7 to 29·5)	6·98<br>(5·45 to 13·5)	-63·9<br>(-74·2 to -52·5)
South Sudan	240<br>(61·8 to 973)	296<br>(58·6 to 1660)	23·1<br>(-63·0 to 167·2)	3·83<br>(1·03 to 13·5)	3·34<br>(0·698 to 15·2)	-12·0<br>(-64·0 to 82·7)
Spain	3180<br>(3060 to 3340)	1780<br>(1670 to 1880)	-44·0<br>(-46·7 to -40·4)	7·46<br>(7·17 to 7·83)	3·63<br>(3·43 to 3·84)	-51·3<br>(-54·1 to -48·7)
Sri Lanka	1220<br>(602 to 1560)	495<br>(334 to 881)	-59·2<br>(-75·4 to -9·0)	6·31<br>(3·11 to 7·99)	2·30<br>(1·57 to 4·06)	-63·5<br>(-77·9 to -20·3)
Sudan	17800<br>(5880 to 31900)	17700<br>(6300 to 28800)	-0·8<br>(-44·1 to 83·1)	61·7<br>(21·0 to 98·7)	40·8<br>(14·4 to 66·3)	-34·0<br>(-58·5 to 10·5)
Suriname	3·91<br>(1·59 to 5·91)	4·60<br>(1·79 to 7·31)	17·6<br>(-24·1 to 71·1)	1·07<br>(0·413 to 1·72)	1·05<br>(0·369 to 1·89)	-2·1<br>(-38·8 to 46·4)
Sweden	500<br>(473 to 520)	519<br>(473 to 574)	4·0<br>(-7·0 to 17·5)	5·57<br>(5·24 to 5·85)	5·06<br>(4·61 to 5·56)	-9·1<br>(-19·0 to 3·1)
Switzerland	123<br>(117 to 129)	65·3<br>(61·4 to 69·2)	-47·0<br>(-50·3 to -42·8)	1·68<br>(1·59 to 1·77)	0·697<br>(0·647 to 0·745)	-58·2<br>(-61·5 to -54·4)
Syria	2930<br>(2410 to 3490)	1860<br>(1350 to 2580)	-36·5<br>(-56·3 to -14·8)	17·1<br>(14·2 to 20·3)	14·4<br>(10·4 to 20·1)	-19·1<br>(-44·1 to 10·9)
São Tomé and Príncipe	16·3<br>(9·45 to 28·5)	20·0<br>(12·0 to 32·9)	22·7<br>(-33·9 to 114·2)	13·7<br>(9·36 to 20·4)	10·7<br>(6·66 to 16·9)	-22·1<br>(-51·9 to 42·8)
Taiwan (province of China)	4090<br>(3830 to 4390)	2800<br>(2530 to 3030)	-31·6<br>(-38·3 to -25·0)	17·6<br>(16·5 to 18·9)	10·9<br>(9·91 to 11·8)	-38·1<br>(-44·4 to -32·5)
Tajikistan	8490<br>(4860 to 11200)	5820<br>(3370 to 10900)	-31·4<br>(-56·5 to 13·7)	141<br>(80·5 to 182)	58·9<br>(35·4 to 106)	-58·7<br>(-73·3 to -34·4)
Tanzania	1420<br>(489 to 5590)	1180<br>(292 to 5030)	-17·2<br>(-76·0 to 60·0)	4·04<br>(1·54 to 14·9)	2·32<br>(0·581 to 10·6)	-42·9<br>(-79·0 to -4·7)
Thailand	1940<br>(965 to 2870)	1560<br>(795 to 2240)	-19·3<br>(-49·9 to 22·2)	2·73<br>(1·42 to 3·96)	2·44<br>(1·17 to 3·39)	-11·9<br>(-44·9 to 29·9)
The Bahamas	27·2<br>(23·6 to 30·6)	18·4<br>(14·5 to 23·1)	-32·3<br>(-46·4 to -12·7)	9·46<br>(8·14 to 10·7)	5·26<br>(3·99 to 6·70)	-44·5<br>(-56·5 to -27·6)
The Gambia	21·4<br>(8·91 to 54·4)	30·9<br>(12·4 to 100)	44·5<br>(-28·5 to 132·1)	2·88<br>(1·14 to 6·98)	2·19<br>(0·947 to 6·71)	-23·9<br>(-60·1 to 23·6)
Timor-Leste	32·9<br>(17·7 to 61·0)	43·9<br>(24·6 to 90·4)	33·5<br>(-27·5 to 113·8)	3·66<br>(1·91 to 6·96)	3·19<br>(1·81 to 6·74)	-13·4<br>(-49·4 to 36·3)
Togo	70·8<br>(40·1 to 153)	116<br>(50·2 to 337)	63·6<br>(-15·8 to 159·1)	2·47<br>(1·37 to 5·40)	1·95<br>(0·840 to 5·53)	-21·0<br>(-60·2 to 23·0)
Tokelau	0·182<br>(0·0680 to 0·366)	0·0988<br>(0·0372 to 0·224)	-45·7<br>(-65·3 to -17·9)	11·5<br>(4·26 to 23·0)	7·03<br>(2·63 to 15·7)	-38·9<br>(-59·5 to -7·1)
Tonga	31·0<br>(19·5 to 44·5)	25·9<br>(16·0 to 38·8)	-16·5<br>(-42·8 to 18·0)	31·2<br>(19·4 to 42·8)	25·0<br>(15·8 to 37·4)	-20·1<br>(-44·3 to 13·7)
Trinidad and Tobago	40·0<br>(36·5 to 44·3)	28·8<br>(22·2 to 36·9)	-28·1<br>(-44·9 to -6·2)	4·05<br>(3·59 to 4·62)	2·49<br>(1·84 to 3·22)	-36·8<br>(-51·3 to -16·6)
Tunisia	3320<br>(1290 to 5410)	2490<br>(918 to 3660)	-25·1<br>(-46·9 to 8·0)	34·3<br>(13·4 to 55·2)	21·0<br>(8·13 to 32·3)	-38·8<br>(-55·0 to -14·9)
Turkmenistan	4570<br>(4130 to 5330)	893<br>(729 to 1090)	-80·5<br>(-84·3 to -75·8)	122<br>(110 to 143)	17·8<br>(14·7 to 21·6)	-84·8<br>(-87·7 to -81·4)
Tuvalu	1·35<br>(0·693 to 2·12)	1·16<br>(0·674 to 1·96)	-14·6<br>(-47·2 to 26·3)	13·6<br>(6·91 to 21·5)	9·14<br>(5·27 to 15·4)	-32·9<br>(-57·4 to -0·6)
Türkiye	15900<br>(11700 to 29400)	15000<br>(11200 to 19000)	-5·7<br>(-53·3 to 39·4)	24·0<br>(17·8 to 44·0)	19·3<br>(14·7 to 24·7)	-21·7<br>(-60·4 to 14·7)

UK	4720<br>(4640 to 4800)	2250<br>(2190 to 2300)	-52·3<br>(-54·0 to -51·1)	8·19<br>(8·06 to 8·32)	3·33<br>(3·25 to 3·42)	-59·3<br>(-60·6 to -58·2)
USA	34000<br>(33300 to 34700)	52700<br>(50800 to 54500)	54·7<br>(47·2 to 60·0)	12·1<br>(11·8 to 12·3)	16·1<br>(15·6 to 16·7)	33·6<br>(26·9 to 38·1)
Uganda	987<br>(295 to 3730)	1080<br>(297 to 4060)	9·0<br>(-61·1 to 198·3)	4·69<br>(1·42 to 17·2)	2·98<br>(0·831 to 11·6)	-36·1<br>(-74·8 to 17·0)
Ukraine	158000<br>(151000 to 164000)	34900<br>(25000 to 47100)	-77·9<br>(-84·3 to -70·5)	293<br>(282 to 305)	70·3<br>(51·9 to 92·3)	-76·2<br>(-82·8 to -69·1)
United Arab Emirates	774<br>(516 to 997)	930<br>(639 to 1240)	20·1<br>(-14·6 to 79·0)	23·7<br>(15·9 to 30·0)	11·4<br>(7·94 to 15·1)	-52·1<br>(-64·7 to -30·8)
Uruguay	683<br>(645 to 724)	618<br>(576 to 664)	-9·6<br>(-16·7 to -1·1)	21·9<br>(20·6 to 23·3)	18·4<br>(17·1 to 19·9)	-14·9<br>(-21·8 to -6·4)
Uzbekistan	25700<br>(23100 to 28800)	11200<br>(9310 to 12900)	-56·2<br>(-65·3 to -46·8)	113<br>(102 to 125)	32·5<br>(27·1 to 37·3)	-71·3<br>(-77·2 to -65·5)
Vanuatu	27·3<br>(10·9 to 43·3)	32·4<br>(15·8 to 56·9)	18·5<br>(-21·0 to 88·1)	13·8<br>(5·46 to 21·2)	10·2<br>(5·13 to 17·6)	-25·8<br>(-47·5 to 10·4)
Venezuela	1220<br>(1100 to 1320)	1620<br>(1150 to 2220)	32·8<br>(-2·4 to 74·6)	5·29<br>(4·82 to 5·72)	6·69<br>(4·80 to 9·20)	26·8<br>(-6·3 to 67·2)
Viet Nam	10400<br>(3240 to 15600)	10500<br>(3450 to 16400)	1·5<br>(-38·5 to 67·7)	13·3<br>(3·97 to 20·0)	9·78<br>(3·37 to 15·0)	-26·5<br>(-55·5 to 22·4)
Virgin Islands	13·9<br>(9·27 to 17·2)	7·23<br>(4·95 to 10·7)	-48·0<br>(-63·3 to -27·0)	14·3<br>(9·58 to 18·1)	10·4<br>(6·78 to 15·9)	-23·8<br>(-49·5 to 9·8)
Yemen	10200<br>(3030 to 18000)	12900<br>(3910 to 23400)	26·1<br>(-24·1 to 110·0)	54·7<br>(16·4 to 92·2)	41·2<br>(12·6 to 73·4)	-24·4<br>(-49·5 to 23·5)
Zambia	628<br>(175 to 2120)	515<br>(133 to 2030)	-18·0<br>(-73·8 to 81·9)	6·29<br>(2·34 to 22·4)	3·46<br>(0·906 to 14·5)	-45·6<br>(-78·8 to -7·7)
Zimbabwe	2990<br>(1140 to 4230)	5350<br>(1910 to 8830)	78·7<br>(13·9 to 168·7)	22·6<br>(8·76 to 31·9)	31·4<br>(11·9 to 51·4)	35·5<br>(-17·2 to 109·6)

Estimates were computed at the draw level in order to propagate uncertainty; manual calculations (eg, for percentage change or grouped locations) may result in slight differences.

Table S9 List of citations for input data sources on carbon monoxide poisoning deaths

Addis Ababa University. Ethiopia - Addis Ababa Mortality Surveillance Program 2008.
Addis Ababa University. Ethiopia - Addis Ababa Mortality Surveillance Program 2009.
Addis Ababa University. Ethiopia - Addis Ababa Mortality Surveillance Program 2011.
Addis Ababa University. Ethiopia - Addis Ababa Mortality Surveillance Program 2012.
Addis Ababa University. Ethiopia - Addis Ababa Mortality Surveillance Program 2015.
Agency of Health Research and Development (Indonesia). Indonesia Basic Health Research 2007-2008.
Agency of Health Research and Development (Indonesia). Indonesia Mortality Registration System Strengthening Project.
Agency of Health Research and Development (Indonesia). Indonesia Sample Registration System - Deaths 2015.
Albania Vital Registration - Deaths 1987 ICD9.
Albania Vital Registration - Deaths 1988 ICD9.
Albania Vital Registration - Deaths 1989 ICD9.
Albania Vital Registration - Deaths 1992 ICD9.
Albania Vital Registration - Deaths 1993 ICD9.
Albania Vital Registration - Deaths 1994 ICD9.
Albania Vital Registration - Deaths 1995 ICD9.
Albania Vital Registration - Deaths 1996 ICD9.
Albania Vital Registration - Deaths 1997 ICD9.
Albania Vital Registration - Deaths 1998 ICD9.
Albania Vital Registration - Deaths 1999 ICD9.
Albania Vital Registration - Deaths 2000 ICD9.
Albania Vital Registration - Deaths 2001 ICD9.
Albania Vital Registration - Deaths 2002 ICD9.
Albania Vital Registration - Deaths 2003 ICD9.
Albania Vital Registration - Deaths 2004 ICD9.
Albania Vital Registration - Deaths 2005 ICD9.
Albania Vital Registration - Deaths 2006 ICD9.
Albania Vital Registration - Deaths 2007 ICD9.
Albania Vital Registration - Deaths 2008 ICD9.
Albania Vital Registration - Deaths 2009 ICD9.
Albania Vital Registration - Deaths 2010 ICD9.
Andorra Vital Registration - Deaths 2011 ICD10.
Andorra Vital Registration - Deaths 2012 ICD10.
Antigua and Barbuda Vital Registration - Deaths 1983 ICD9.
Antigua and Barbuda Vital Registration - Deaths 1985 ICD9.
Antigua and Barbuda Vital Registration - Deaths 1986 ICD9.
Antigua and Barbuda Vital Registration - Deaths 1987 ICD9.
Antigua and Barbuda Vital Registration - Deaths 1988 ICD9.
Antigua and Barbuda Vital Registration - Deaths 1989 ICD9.
Antigua and Barbuda Vital Registration - Deaths 1990 ICD9.
Antigua and Barbuda Vital Registration - Deaths 1991 ICD9.
Antigua and Barbuda Vital Registration - Deaths 1992 ICD9.
Antigua and Barbuda Vital Registration - Deaths 1993 ICD9.
Antigua and Barbuda Vital Registration - Deaths 1994 ICD9.
Antigua and Barbuda Vital Registration - Deaths 1995 ICD9.
Antigua and Barbuda Vital Registration - Deaths 2000 ICD10.
Antigua and Barbuda Vital Registration - Deaths 2001 ICD10.
Antigua and Barbuda Vital Registration - Deaths 2002 ICD10.
Antigua and Barbuda Vital Registration - Deaths 2003 ICD10.
Antigua and Barbuda Vital Registration - Deaths 2004 ICD10.
Antigua and Barbuda Vital Registration - Deaths 2005 ICD10.
Antigua and Barbuda Vital Registration - Deaths 2006 ICD10.
Antigua and Barbuda Vital Registration - Deaths 2007 ICD10.
Antigua and Barbuda Vital Registration - Deaths 2008 ICD10.
Antigua and Barbuda Vital Registration - Deaths 2009 ICD10.
Antigua and Barbuda Vital Registration - Deaths 2012 ICD10.
Antigua and Barbuda Vital Registration - Deaths 2013 ICD10.
Antigua and Barbuda Vital Registration - Deaths 2014 ICD10.
Antigua and Barbuda Vital Registration - Deaths 2015 ICD10.

Antigua and Barbuda Vital Registration - Deaths 2016 ICD10.
Antigua and Barbuda Vital Registration - Deaths 2017 ICD10.
Argentina Vital Registration - Deaths 1980 ICD9.
Argentina Vital Registration - Deaths 1981 ICD9.
Argentina Vital Registration - Deaths 1982 ICD9.
Argentina Vital Registration - Deaths 1983 ICD9.
Argentina Vital Registration - Deaths 1984 ICD9.
Argentina Vital Registration - Deaths 1985 ICD9.
Argentina Vital Registration - Deaths 1986 ICD9.
Argentina Vital Registration - Deaths 1987 ICD9.
Argentina Vital Registration - Deaths 1988 ICD9.
Argentina Vital Registration - Deaths 1989 ICD9.
Argentina Vital Registration - Deaths 1990 ICD9.
Argentina Vital Registration - Deaths 1991 ICD9.
Argentina Vital Registration - Deaths 1992 ICD9.
Argentina Vital Registration - Deaths 1993 ICD9.
Argentina Vital Registration - Deaths 1994 ICD9.
Argentina Vital Registration - Deaths 1995 ICD9.
Argentina Vital Registration - Deaths 1996 ICD9.
Argentina Vital Registration - Deaths 1997 ICD10.
Argentina Vital Registration - Deaths 1998 ICD10.
Argentina Vital Registration - Deaths 1999 ICD10.
Argentina Vital Registration - Deaths 2000 ICD10.
Argentina Vital Registration - Deaths 2001 ICD10.
Argentina Vital Registration - Deaths 2002 ICD10.
Argentina Vital Registration - Deaths 2003 ICD10.
Argentina Vital Registration - Deaths 2004 ICD10.
Argentina Vital Registration - Deaths 2005 ICD10.
Argentina Vital Registration - Deaths 2006 ICD10.
Argentina Vital Registration - Deaths 2007 ICD10.
Argentina Vital Registration - Deaths 2008 ICD10.
Argentina Vital Registration - Deaths 2009 ICD10.
Argentina Vital Registration - Deaths 2010 ICD10.
Argentina Vital Registration - Deaths 2011 ICD10.
Argentina Vital Registration - Deaths 2012 ICD10.
Argentina Vital Registration - Deaths 2013 ICD10.
Argentina Vital Registration - Deaths 2014 ICD10.
Argentina Vital Registration - Deaths 2015 ICD10.
Argentina Vital Registration - Deaths 2016 ICD10.
Argentina Vital Registration - Deaths 2017 ICD10.
Argentina Vital Registration - Deaths 2018 ICD10.
Armenia Vital Registration - Deaths 1981 ICD9.
Armenia Vital Registration - Deaths 1982 ICD9.
Armenia Vital Registration - Deaths 1985 ICD9.
Armenia Vital Registration - Deaths 1986 ICD9.
Armenia Vital Registration - Deaths 1987 ICD9.
Armenia Vital Registration - Deaths 1988 ICD9.
Armenia Vital Registration - Deaths 1989 ICD9.
Armenia Vital Registration - Deaths 1990 ICD9.
Armenia Vital Registration - Deaths 1991 ICD9.
Armenia Vital Registration - Deaths 1992 ICD9.
Armenia Vital Registration - Deaths 1993 ICD9.
Armenia Vital Registration - Deaths 1994 ICD9.
Armenia Vital Registration - Deaths 1995 ICD9.
Armenia Vital Registration - Deaths 1996 ICD9.
Armenia Vital Registration - Deaths 1997 ICD9.
Armenia Vital Registration - Deaths 1998 ICD9.
Armenia Vital Registration - Deaths 1999 ICD9.
Armenia Vital Registration - Deaths 2000 ICD9.
Armenia Vital Registration - Deaths 2001 ICD9.
Armenia Vital Registration - Deaths 2002 ICD9.

Armenia Vital Registration - Deaths 2003 ICD9.
Armenia Vital Registration - Deaths 2006 ICD10.
Armenia Vital Registration - Deaths 2007 ICD10.
Armenia Vital Registration - Deaths 2008 ICD10.
Armenia Vital Registration - Deaths 2009 ICD10.
Armenia Vital Registration - Deaths 2010 ICD10.
Armenia Vital Registration - Deaths 2011 ICD10.
Armenia Vital Registration - Deaths 2012 ICD10.
Armenia Vital Registration - Deaths 2013 ICD10.
Armenia Vital Registration - Deaths 2014 ICD10.
Armenia Vital Registration - Deaths 2015 ICD10.
Armenia Vital Registration - Deaths 2016 ICD10.
Armenia Vital Registration - Deaths 2017 ICD10.
Armenia Vital Registration - Deaths 2018 ICD10.
Associates for Community and Population Research (ACPR), International Centre for Diarrhoeal Disease Research, Bangladesh (ICDDR,B), Johns Hopkins University, Mitra and Associates, National Institute of Population Research and Training (NIPORT), ORC Macro. Bangladesh Special Demographic and Health Survey 2001. Fairfax, United States of America: ICF International.
Australia Vital Registration - Deaths 1980 ICD9.
Australia Vital Registration - Deaths 1981 ICD9.
Australia Vital Registration - Deaths 1982 ICD9.
Australia Vital Registration - Deaths 1983 ICD9.
Australia Vital Registration - Deaths 1984 ICD9.
Australia Vital Registration - Deaths 1985 ICD9.
Australia Vital Registration - Deaths 1986 ICD9.
Australia Vital Registration - Deaths 1987 ICD9.
Australia Vital Registration - Deaths 1988 ICD9.
Australia Vital Registration - Deaths 1989 ICD9.
Australia Vital Registration - Deaths 1990 ICD9.
Australia Vital Registration - Deaths 1991 ICD9.
Australia Vital Registration - Deaths 1992 ICD9.
Australia Vital Registration - Deaths 1993 ICD9.
Australia Vital Registration - Deaths 1994 ICD9.
Australia Vital Registration - Deaths 1995 ICD9.
Australia Vital Registration - Deaths 1996 ICD9.
Australia Vital Registration - Deaths 1997 ICD9.
Australia Vital Registration - Deaths 1998 ICD10.
Australia Vital Registration - Deaths 1999 ICD10.
Australia Vital Registration - Deaths 2000 ICD10.
Australia Vital Registration - Deaths 2001 ICD10.
Australia Vital Registration - Deaths 2002 ICD10.
Australia Vital Registration - Deaths 2003 ICD10.
Australia Vital Registration - Deaths 2004 ICD10.
Australia Vital Registration - Deaths 2006 ICD10.
Australia Vital Registration - Deaths 2007 ICD10.
Australia Vital Registration - Deaths 2008 ICD10.
Australia Vital Registration - Deaths 2009 ICD10.
Australia Vital Registration - Deaths 2010 ICD10.
Australia Vital Registration - Deaths 2011 ICD10.
Australia Vital Registration - Deaths 2012 ICD10.
Australia Vital Registration - Deaths 2013 ICD10.
Australia Vital Registration - Deaths 2014 ICD10.
Australia Vital Registration - Deaths 2015 ICD10.
Australia Vital Registration - Deaths 2016 ICD10.
Australia Vital Registration - Deaths 2017 ICD10.
Australia Vital Registration - Deaths 2018 ICD10.
Australian Bureau of Statistics. Australia Vital Registration - Deaths 2005.
Austria Vital Registration - Deaths 1980 ICD9.
Austria Vital Registration - Deaths 1981 ICD9.
Austria Vital Registration - Deaths 1982 ICD9.
Austria Vital Registration - Deaths 1983 ICD9.
Austria Vital Registration - Deaths 1984 ICD9.

Austria Vital Registration - Deaths 1985 ICD9.
Austria Vital Registration - Deaths 1986 ICD9.
Austria Vital Registration - Deaths 1987 ICD9.
Austria Vital Registration - Deaths 1988 ICD9.
Austria Vital Registration - Deaths 1989 ICD9.
Austria Vital Registration - Deaths 1990 ICD9.
Austria Vital Registration - Deaths 1991 ICD9.
Austria Vital Registration - Deaths 1992 ICD9.
Austria Vital Registration - Deaths 1993 ICD9.
Austria Vital Registration - Deaths 1994 ICD9.
Austria Vital Registration - Deaths 1995 ICD9.
Austria Vital Registration - Deaths 1996 ICD9.
Austria Vital Registration - Deaths 1997 ICD9.
Austria Vital Registration - Deaths 1998 ICD9.
Austria Vital Registration - Deaths 1999 ICD9.
Austria Vital Registration - Deaths 2000 ICD9.
Austria Vital Registration - Deaths 2001 ICD9.
Austria Vital Registration - Deaths 2002 ICD10.
Austria Vital Registration - Deaths 2003 ICD10.
Austria Vital Registration - Deaths 2004 ICD10.
Austria Vital Registration - Deaths 2005 ICD10.
Austria Vital Registration - Deaths 2006 ICD10.
Austria Vital Registration - Deaths 2007 ICD10.
Austria Vital Registration - Deaths 2008 ICD10.
Austria Vital Registration - Deaths 2009 ICD10.
Austria Vital Registration - Deaths 2010 ICD10.
Austria Vital Registration - Deaths 2011 ICD10.
Austria Vital Registration - Deaths 2012 ICD10.
Austria Vital Registration - Deaths 2013 ICD10.
Austria Vital Registration - Deaths 2014 ICD10.
Austria Vital Registration - Deaths 2015 ICD10.
Austria Vital Registration - Deaths 2016 ICD10.
Austria Vital Registration - Deaths 2017 ICD10.
Austria Vital Registration - Deaths 2018 ICD10.
Azerbaijan Vital Registration - Deaths 1981 ICD9.
Azerbaijan Vital Registration - Deaths 1982 ICD9.
Azerbaijan Vital Registration - Deaths 1985 ICD9.
Azerbaijan Vital Registration - Deaths 1986 ICD9.
Azerbaijan Vital Registration - Deaths 1987 ICD9.
Azerbaijan Vital Registration - Deaths 1988 ICD9.
Azerbaijan Vital Registration - Deaths 1989 ICD9.
Azerbaijan Vital Registration - Deaths 1990 ICD9.
Azerbaijan Vital Registration - Deaths 1991 ICD9.
Azerbaijan Vital Registration - Deaths 1992 ICD9.
Azerbaijan Vital Registration - Deaths 1993 ICD9.
Azerbaijan Vital Registration - Deaths 1994 ICD9.
Azerbaijan Vital Registration - Deaths 1995 ICD9.
Azerbaijan Vital Registration - Deaths 1996 ICD9.
Azerbaijan Vital Registration - Deaths 1997 ICD9.
Azerbaijan Vital Registration - Deaths 1998 ICD9.
Azerbaijan Vital Registration - Deaths 1999 ICD9.
Azerbaijan Vital Registration - Deaths 2000 ICD9.
Azerbaijan Vital Registration - Deaths 2001 ICD10.
Azerbaijan Vital Registration - Deaths 2002 ICD10.
Azerbaijan Vital Registration - Deaths 2003 ICD10.
Azerbaijan Vital Registration - Deaths 2004 ICD10.
Azerbaijan Vital Registration - Deaths 2007 ICD10.
Bahamas Vital Registration - Deaths 1980 ICD9.
Bahamas Vital Registration - Deaths 1981 ICD9.
Bahamas Vital Registration - Deaths 1984 ICD9.
Bahamas Vital Registration - Deaths 1985 ICD9.

Bahamas Vital Registration - Deaths 1987 ICD9.
Bahamas Vital Registration - Deaths 1993 ICD9.
Bahamas Vital Registration - Deaths 1994 ICD9.
Bahamas Vital Registration - Deaths 1995 ICD9.
Bahamas Vital Registration - Deaths 1996 ICD9.
Bahamas Vital Registration - Deaths 1997 ICD9.
Bahamas Vital Registration - Deaths 1998 ICD9.
Bahamas Vital Registration - Deaths 1999 ICD10.
Bahamas Vital Registration - Deaths 2000 ICD10.
Bahamas Vital Registration - Deaths 2001 ICD10.
Bahamas Vital Registration - Deaths 2002 ICD10.
Bahamas Vital Registration - Deaths 2003 ICD10.
Bahamas Vital Registration - Deaths 2004 ICD10.
Bahamas Vital Registration - Deaths 2005 ICD10.
Bahamas Vital Registration - Deaths 2006 ICD10.
Bahamas Vital Registration - Deaths 2007 ICD10.
Bahamas Vital Registration - Deaths 2008 ICD10.
Bahamas Vital Registration - Deaths 2009 ICD10.
Bahamas Vital Registration - Deaths 2010 ICD10.
Bahamas Vital Registration - Deaths 2011 ICD10.
Bahamas Vital Registration - Deaths 2012 ICD10.
Bahamas Vital Registration - Deaths 2013 ICD10.
Bahamas Vital Registration - Deaths 2014 ICD10.
Bahrain Vital Registration - Deaths 1985 ICD9.
Bahrain Vital Registration - Deaths 1987 ICD9.
Bahrain Vital Registration - Deaths 1988 ICD9.
Bahrain Vital Registration - Deaths 1997 ICD10.
Bahrain Vital Registration - Deaths 1998 ICD10.
Bahrain Vital Registration - Deaths 1999 ICD10.
Bahrain Vital Registration - Deaths 2000 ICD10.
Bahrain Vital Registration - Deaths 2001 ICD10.
Bahrain Vital Registration - Deaths 2002 ICD10.
Bahrain Vital Registration - Deaths 2003 ICD10.
Bahrain Vital Registration - Deaths 2004 ICD10.
Bahrain Vital Registration - Deaths 2005 ICD10.
Bahrain Vital Registration - Deaths 2006 ICD10.
Bahrain Vital Registration - Deaths 2007 ICD10.
Bahrain Vital Registration - Deaths 2008 ICD10.
Bahrain Vital Registration - Deaths 2009 ICD10.
Bahrain Vital Registration - Deaths 2010 ICD10.
Bahrain Vital Registration - Deaths 2011 ICD10.
Bahrain Vital Registration - Deaths 2012 ICD10.
Bahrain Vital Registration - Deaths 2013 ICD10.
Bahrain Vital Registration - Deaths 2014 ICD10.
Barbados Vital Registration - Deaths 1980 ICD9.
Barbados Vital Registration - Deaths 1981 ICD9.
Barbados Vital Registration - Deaths 1982 ICD9.
Barbados Vital Registration - Deaths 1983 ICD9.
Barbados Vital Registration - Deaths 1984 ICD9.
Barbados Vital Registration - Deaths 1985 ICD9.
Barbados Vital Registration - Deaths 1986 ICD9.
Barbados Vital Registration - Deaths 1987 ICD9.
Barbados Vital Registration - Deaths 1988 ICD9.
Barbados Vital Registration - Deaths 1989 ICD9.
Barbados Vital Registration - Deaths 1990 ICD9.
Barbados Vital Registration - Deaths 1991 ICD9.
Barbados Vital Registration - Deaths 1992 ICD9.
Barbados Vital Registration - Deaths 1993 ICD9.
Barbados Vital Registration - Deaths 1994 ICD9.
Barbados Vital Registration - Deaths 1995 ICD9.
Barbados Vital Registration - Deaths 2000 ICD10.

Barbados Vital Registration - Deaths 2001 ICD10.
Barbados Vital Registration - Deaths 2002 ICD10.
Barbados Vital Registration - Deaths 2003 ICD10.
Barbados Vital Registration - Deaths 2004 ICD10.
Barbados Vital Registration - Deaths 2005 ICD10.
Barbados Vital Registration - Deaths 2006 ICD10.
Barbados Vital Registration - Deaths 2007 ICD10.
Barbados Vital Registration - Deaths 2008 ICD10.
Barbados Vital Registration - Deaths 2009 ICD10.
Barbados Vital Registration - Deaths 2010 ICD10.
Barbados Vital Registration - Deaths 2011 ICD10.
Barbados Vital Registration - Deaths 2012 ICD10.
Barbados Vital Registration - Deaths 2013 ICD10.
Baskent University, Ministry of Health (Turkey), State Institute of Statistics (Turkey). Turkey Verbal Autopsy Survey 2003.
Belarus Vital Registration - Deaths 1981 ICD9.
Belarus Vital Registration - Deaths 1982 ICD9.
Belarus Vital Registration - Deaths 1985 ICD9.
Belarus Vital Registration - Deaths 1986 ICD9.
Belarus Vital Registration - Deaths 1987 ICD9.
Belarus Vital Registration - Deaths 1988 ICD9.
Belarus Vital Registration - Deaths 1989 ICD9.
Belarus Vital Registration - Deaths 1990 ICD9.
Belarus Vital Registration - Deaths 1991 ICD9.
Belarus Vital Registration - Deaths 1992 ICD9.
Belarus Vital Registration - Deaths 1993 ICD9.
Belarus Vital Registration - Deaths 1994 ICD9.
Belarus Vital Registration - Deaths 1995 ICD9.
Belarus Vital Registration - Deaths 1997 ICD9.
Belarus Vital Registration - Deaths 1998 ICD9.
Belarus Vital Registration - Deaths 1999 ICD9.
Belarus Vital Registration - Deaths 2000 ICD9.
Belarus Vital Registration - Deaths 2001 ICD9.
Belarus Vital Registration - Deaths 2002 ICD10.
Belarus Vital Registration - Deaths 2003 ICD10.
Belarus Vital Registration - Deaths 2007 ICD10.
Belarus Vital Registration - Deaths 2008 ICD10.
Belarus Vital Registration - Deaths 2009 ICD10.
Belarus Vital Registration - Deaths 2010 ICD10.
Belarus Vital Registration - Deaths 2011 ICD10.
Belarus Vital Registration - Deaths 2013 ICD10.
Belarus Vital Registration - Deaths 2014 ICD10.
Belarus Vital Registration - Deaths 2018 ICD10.
Belgium Vital Registration - Deaths 1980 ICD9.
Belgium Vital Registration - Deaths 1981 ICD9.
Belgium Vital Registration - Deaths 1982 ICD9.
Belgium Vital Registration - Deaths 1983 ICD9.
Belgium Vital Registration - Deaths 1984 ICD9.
Belgium Vital Registration - Deaths 1985 ICD9.
Belgium Vital Registration - Deaths 1986 ICD9.
Belgium Vital Registration - Deaths 1987 ICD9.
Belgium Vital Registration - Deaths 1988 ICD9.
Belgium Vital Registration - Deaths 1989 ICD9.
Belgium Vital Registration - Deaths 1990 ICD9.
Belgium Vital Registration - Deaths 1991 ICD9.
Belgium Vital Registration - Deaths 1992 ICD9.
Belgium Vital Registration - Deaths 1993 ICD9.
Belgium Vital Registration - Deaths 1994 ICD9.
Belgium Vital Registration - Deaths 1995 ICD9.
Belgium Vital Registration - Deaths 1996 ICD9.
Belgium Vital Registration - Deaths 1997 ICD9.
Belgium Vital Registration - Deaths 1998 ICD10.

Belgium Vital Registration - Deaths 1999 ICD10.
Belgium Vital Registration - Deaths 2000 ICD10.
Belgium Vital Registration - Deaths 2001 ICD10.
Belgium Vital Registration - Deaths 2002 ICD10.
Belgium Vital Registration - Deaths 2003 ICD10.
Belgium Vital Registration - Deaths 2004 ICD10.
Belgium Vital Registration - Deaths 2005 ICD10.
Belgium Vital Registration - Deaths 2006 ICD10.
Belgium Vital Registration - Deaths 2007 ICD10.
Belgium Vital Registration - Deaths 2008 ICD10.
Belgium Vital Registration - Deaths 2009 ICD10.
Belgium Vital Registration - Deaths 2010 ICD10.
Belgium Vital Registration - Deaths 2011 ICD10.
Belgium Vital Registration - Deaths 2012 ICD10.
Belgium Vital Registration - Deaths 2013 ICD10.
Belgium Vital Registration - Deaths 2014 ICD10.
Belgium Vital Registration - Deaths 2015 ICD10.
Belgium Vital Registration - Deaths 2016 ICD10.
Belize Vital Registration - Deaths 1980 ICD9.
Belize Vital Registration - Deaths 1981 ICD9.
Belize Vital Registration - Deaths 1982 ICD9.
Belize Vital Registration - Deaths 1983 ICD9.
Belize Vital Registration - Deaths 1984 ICD9.
Belize Vital Registration - Deaths 1986 ICD9.
Belize Vital Registration - Deaths 1987 ICD9.
Belize Vital Registration - Deaths 1989 ICD9.
Belize Vital Registration - Deaths 1990 ICD9.
Belize Vital Registration - Deaths 1991 ICD9.
Belize Vital Registration - Deaths 1993 ICD9.
Belize Vital Registration - Deaths 1994 ICD9.
Belize Vital Registration - Deaths 1995 ICD9.
Belize Vital Registration - Deaths 1996 ICD9.
Belize Vital Registration - Deaths 1997 ICD10.
Belize Vital Registration - Deaths 1998 ICD10.
Belize Vital Registration - Deaths 1999 ICD10.
Belize Vital Registration - Deaths 2000 ICD10.
Belize Vital Registration - Deaths 2001 ICD10.
Belize Vital Registration - Deaths 2002 ICD10.
Belize Vital Registration - Deaths 2003 ICD10.
Belize Vital Registration - Deaths 2004 ICD10.
Belize Vital Registration - Deaths 2005 ICD10.
Belize Vital Registration - Deaths 2006 ICD10.
Belize Vital Registration - Deaths 2007 ICD10.
Belize Vital Registration - Deaths 2008 ICD10.
Belize Vital Registration - Deaths 2009 ICD10.
Belize Vital Registration - Deaths 2010 ICD10.
Belize Vital Registration - Deaths 2011 ICD10.
Belize Vital Registration - Deaths 2012 ICD10.
Belize Vital Registration - Deaths 2013 ICD10.
Belize Vital Registration - Deaths 2014 ICD10.
Belize Vital Registration - Deaths 2015 ICD10.
Belize Vital Registration - Deaths 2016 ICD10.
Bermuda Vital Registration - Deaths 1980 ICD9.
Bermuda Vital Registration - Deaths 1983 ICD9.
Bermuda Vital Registration - Deaths 1984 ICD9.
Bermuda Vital Registration - Deaths 1985 ICD9.
Bermuda Vital Registration - Deaths 1986 ICD9.
Bermuda Vital Registration - Deaths 1987 ICD9.
Bermuda Vital Registration - Deaths 1988 ICD9.
Bermuda Vital Registration - Deaths 1989 ICD9.
Bermuda Vital Registration - Deaths 1990 ICD9.

Bermuda Vital Registration - Deaths 1991 ICD9.
Bermuda Vital Registration - Deaths 1992 ICD9.
Bermuda Vital Registration - Deaths 1993 ICD9.
Bermuda Vital Registration - Deaths 1994 ICD9.
Bermuda Vital Registration - Deaths 1995 ICD9.
Bermuda Vital Registration - Deaths 1996 ICD10.
Bermuda Vital Registration - Deaths 1997 ICD10.
Bermuda Vital Registration - Deaths 1998 ICD10.
Bermuda Vital Registration - Deaths 1999 ICD10.
Bermuda Vital Registration - Deaths 2000 ICD10.
Bermuda Vital Registration - Deaths 2001 ICD10.
Bermuda Vital Registration - Deaths 2002 ICD10.
Bermuda Vital Registration - Deaths 2003 ICD10.
Bermuda Vital Registration - Deaths 2004 ICD10.
Bermuda Vital Registration - Deaths 2005 ICD10.
Bermuda Vital Registration - Deaths 2006 ICD10.
Bermuda Vital Registration - Deaths 2007 ICD10.
Bermuda Vital Registration - Deaths 2008 ICD10.
Bermuda Vital Registration - Deaths 2009 ICD10.
Bermuda Vital Registration - Deaths 2010 ICD10.
Bermuda Vital Registration - Deaths 2011 ICD10.
Bermuda Vital Registration - Deaths 2012 ICD10.
Bermuda Vital Registration - Deaths 2013 ICD10.
Bermuda Vital Registration - Deaths 2014 ICD10.
Bermuda Vital Registration - Deaths 2015 ICD10.
Bermuda Vital Registration - Deaths 2016 ICD10.
Bermuda Vital Registration - Deaths 2017 ICD10.
Births and Deaths Registry (Ghana). Ghana - Accra Births and Deaths Registry - Deaths 2000-2007.
Bosnia and Herzegovina Vital Registration - Deaths 1991 ICD9.
Bosnia and Herzegovina Vital Registration - Deaths 2011 ICD10.
Bosnia and Herzegovina Vital Registration - Deaths 2014 ICD10.
Bosnia and Herzegovina Vital Registration - Deaths 2016 ICD10.
Brunei Darussalam Vital Registration - Deaths 2017 ICD10.
Brunei Darussalam Vital Registration - Deaths 2018 ICD10.
Brunei Vital Registration - Deaths 1996 ICD10.
Brunei Vital Registration - Deaths 1997 ICD10.
Brunei Vital Registration - Deaths 1998 ICD10.
Brunei Vital Registration - Deaths 1999 ICD10.
Brunei Vital Registration - Deaths 2000 ICD10.
Brunei Vital Registration - Deaths 2001 ICD10.
Brunei Vital Registration - Deaths 2002 ICD10.
Brunei Vital Registration - Deaths 2003 ICD10.
Brunei Vital Registration - Deaths 2004 ICD10.
Brunei Vital Registration - Deaths 2005 ICD10.
Brunei Vital Registration - Deaths 2006 ICD10.
Brunei Vital Registration - Deaths 2007 ICD10.
Brunei Vital Registration - Deaths 2008 ICD10.
Brunei Vital Registration - Deaths 2009 ICD10.
Brunei Vital Registration - Deaths 2010 ICD10.
Brunei Vital Registration - Deaths 2011 ICD10.
Brunei Vital Registration - Deaths 2012 ICD10.
Brunei Vital Registration - Deaths 2013 ICD10.
Brunei Vital Registration - Deaths 2014 ICD10.
Brunei Vital Registration - Deaths 2015 ICD10.
Brunei Vital Registration - Deaths 2016 ICD10.
Bulgaria Vital Registration - Deaths 1980 ICD9.
Bulgaria Vital Registration - Deaths 1981 ICD9.
Bulgaria Vital Registration - Deaths 1982 ICD9.
Bulgaria Vital Registration - Deaths 1983 ICD9.
Bulgaria Vital Registration - Deaths 1984 ICD9.
Bulgaria Vital Registration - Deaths 1985 ICD9.

Bulgaria Vital Registration - Deaths 1986 ICD9.
Bulgaria Vital Registration - Deaths 1987 ICD9.
Bulgaria Vital Registration - Deaths 1988 ICD9.
Bulgaria Vital Registration - Deaths 1989 ICD9.
Bulgaria Vital Registration - Deaths 1990 ICD9.
Bulgaria Vital Registration - Deaths 1991 ICD9.
Bulgaria Vital Registration - Deaths 1992 ICD9.
Bulgaria Vital Registration - Deaths 1993 ICD9.
Bulgaria Vital Registration - Deaths 1994 ICD9.
Bulgaria Vital Registration - Deaths 1995 ICD9.
Bulgaria Vital Registration - Deaths 1996 ICD9.
Bulgaria Vital Registration - Deaths 1997 ICD9.
Bulgaria Vital Registration - Deaths 1998 ICD9.
Bulgaria Vital Registration - Deaths 1999 ICD9.
Bulgaria Vital Registration - Deaths 2000 ICD9.
Bulgaria Vital Registration - Deaths 2001 ICD9.
Bulgaria Vital Registration - Deaths 2002 ICD9.
Bulgaria Vital Registration - Deaths 2003 ICD9.
Bulgaria Vital Registration - Deaths 2004 ICD9.
Bulgaria Vital Registration - Deaths 2005 ICD10.
Bulgaria Vital Registration - Deaths 2006 ICD10.
Bulgaria Vital Registration - Deaths 2007 ICD10.
Bulgaria Vital Registration - Deaths 2008 ICD10.
Bulgaria Vital Registration - Deaths 2009 ICD10.
Bulgaria Vital Registration - Deaths 2010 ICD10.
Bulgaria Vital Registration - Deaths 2011 ICD10.
Bulgaria Vital Registration - Deaths 2012 ICD10.
Bulgaria Vital Registration - Deaths 2013 ICD10.
Bulgaria Vital Registration - Deaths 2014 ICD10.
Bulgaria Vital Registration - Deaths 2015 ICD10.
Bulgaria Vital Registration - Deaths 2016 ICD10.
Bulgaria Vital Registration - Deaths 2017 ICD10.
Bulgaria Vital Registration - Deaths 2018 ICD10.
Canada Vital Registration - Deaths 1980 ICD9.
Canada Vital Registration - Deaths 1981 ICD9.
Canada Vital Registration - Deaths 1982 ICD9.
Canada Vital Registration - Deaths 1983 ICD9.
Canada Vital Registration - Deaths 1984 ICD9.
Canada Vital Registration - Deaths 1985 ICD9.
Canada Vital Registration - Deaths 1986 ICD9.
Canada Vital Registration - Deaths 1987 ICD9.
Canada Vital Registration - Deaths 1988 ICD9.
Canada Vital Registration - Deaths 1989 ICD9.
Canada Vital Registration - Deaths 1990 ICD9.
Canada Vital Registration - Deaths 1991 ICD9.
Canada Vital Registration - Deaths 1992 ICD9.
Canada Vital Registration - Deaths 1993 ICD9.
Canada Vital Registration - Deaths 1994 ICD9.
Canada Vital Registration - Deaths 1995 ICD9.
Canada Vital Registration - Deaths 1996 ICD9.
Canada Vital Registration - Deaths 1997 ICD9.
Canada Vital Registration - Deaths 1998 ICD9.
Canada Vital Registration - Deaths 1999 ICD9.
Canada Vital Registration - Deaths 2000 ICD10.
Canada Vital Registration - Deaths 2001 ICD10.
Canada Vital Registration - Deaths 2002 ICD10.
Canada Vital Registration - Deaths 2003 ICD10.
Canada Vital Registration - Deaths 2004 ICD10.
Canada Vital Registration - Deaths 2005 ICD10.
Canada Vital Registration - Deaths 2006 ICD10.
Canada Vital Registration - Deaths 2007 ICD10.

Canada Vital Registration - Deaths 2008 ICD10.
Canada Vital Registration - Deaths 2009 ICD10.
Canada Vital Registration - Deaths 2010 ICD10.
Canada Vital Registration - Deaths 2011 ICD10.
Canada Vital Registration - Deaths 2012 ICD10.
Canada Vital Registration - Deaths 2013 ICD10.
Canada Vital Registration - Deaths 2014 ICD10.
Canada Vital Registration - Deaths 2015 ICD10.
Canada Vital Registration - Deaths 2016 ICD10.
Canada Vital Registration - Deaths 2017 ICD10.
Cape Verde Vital Registration - Deaths 2011 ICD10.
Cape Verde Vital Registration - Deaths 2012 ICD10.
Center for Demographic Research, New Economic School (Russia). Russia Mortality by Region, Age, Sex, and Cause of Death 1989. Moscow, Russia: Center for Demographic Research, New Economic School (Russia). http://demogr.nes.ru/index.php/ru/demogr_indicat/data
Center for Demographic Research, New Economic School (Russia). Russia Mortality by Region, Age, Sex, and Cause of Death 1990. Moscow, Russia: Center for Demographic Research, New Economic School (Russia). http://demogr.nes.ru/index.php/ru/demogr_indicat/data
Center for Demographic Research, New Economic School (Russia). Russia Mortality by Region, Age, Sex, and Cause of Death 1991. Moscow, Russia: Center for Demographic Research, New Economic School (Russia). http://demogr.nes.ru/index.php/ru/demogr_indicat/data
Center for Demographic Research, New Economic School (Russia). Russia Mortality by Region, Age, Sex, and Cause of Death 1992. Moscow, Russia: Center for Demographic Research, New Economic School (Russia). http://demogr.nes.ru/index.php/ru/demogr_indicat/data
Center for Demographic Research, New Economic School (Russia). Russia Mortality by Region, Age, Sex, and Cause of Death 1993. Moscow, Russia: Center for Demographic Research, New Economic School (Russia). http://demogr.nes.ru/index.php/ru/demogr_indicat/data
Center for Demographic Research, New Economic School (Russia). Russia Mortality by Region, Age, Sex, and Cause of Death 1994. Moscow, Russia: Center for Demographic Research, New Economic School (Russia). http://demogr.nes.ru/index.php/ru/demogr_indicat/data
Center for Demographic Research, New Economic School (Russia). Russia Mortality by Region, Age, Sex, and Cause of Death 1995. Moscow, Russia: Center for Demographic Research, New Economic School (Russia). http://demogr.nes.ru/index.php/ru/demogr_indicat/data
Center for Demographic Research, New Economic School (Russia). Russia Mortality by Region, Age, Sex, and Cause of Death 1996. Moscow, Russia: Center for Demographic Research, New Economic School (Russia). http://demogr.nes.ru/index.php/ru/demogr_indicat/data
Center for Demographic Research, New Economic School (Russia). Russia Mortality by Region, Age, Sex, and Cause of Death 1997. Moscow, Russia: Center for Demographic Research, New Economic School (Russia). http://demogr.nes.ru/index.php/ru/demogr_indicat/data
Center for Demographic Research, New Economic School (Russia). Russia Mortality by Region, Age, Sex, and Cause of Death 1998. Moscow, Russia: Center for Demographic Research, New Economic School (Russia). http://demogr.nes.ru/index.php/ru/demogr_indicat/data
Center for Demographic Research, New Economic School (Russia). Russia Mortality by Region, Age, Sex, and Cause of Death 1999. Moscow, Russia: Center for Demographic Research, New Economic School (Russia). http://demogr.nes.ru/index.php/ru/demogr_indicat/data
Center for Demographic Research, New Economic School (Russia). Russia Mortality by Region, Age, Sex, and Cause of Death 2000. Moscow, Russia: Center for Demographic Research, New Economic School (Russia). http://demogr.nes.ru/index.php/ru/demogr_indicat/data
Center for Demographic Research, New Economic School (Russia). Russia Mortality by Region, Age, Sex, and Cause of Death 2001. Moscow, Russia: Center for Demographic Research, New Economic School (Russia). http://demogr.nes.ru/index.php/ru/demogr_indicat/data
Center for Demographic Research, New Economic School (Russia). Russia Mortality by Region, Age, Sex, and Cause of Death 2002. Moscow, Russia: Center for Demographic Research, New Economic School (Russia). http://demogr.nes.ru/index.php/ru/demogr_indicat/data
Center for Demographic Research, New Economic School (Russia). Russia Mortality by Region, Age, Sex, and Cause of Death 2003. Moscow, Russia: Center for Demographic Research, New Economic School (Russia). http://demogr.nes.ru/index.php/ru/demogr_indicat/data
Center for Demographic Research, New Economic School (Russia). Russia Mortality by Region, Age, Sex, and Cause of Death 2004. Moscow, Russia: Center for Demographic Research, New Economic School (Russia). http://demogr.nes.ru/index.php/ru/demogr_indicat/data
Center for Demographic Research, New Economic School (Russia). Russia Mortality by Region, Age, Sex, and Cause of Death 2005. Moscow, Russia: Center for Demographic Research, New Economic School (Russia). http://demogr.nes.ru/index.php/ru/demogr_indicat/data
Center for Demographic Research, New Economic School (Russia). Russia Mortality by Region, Age, Sex, and Cause of Death 2006. Moscow, Russia: Center for Demographic Research, New Economic School (Russia). http://demogr.nes.ru/index.php/ru/demogr_indicat/data
Center for Demographic Research, New Economic School (Russia). Russia Mortality by Region, Age, Sex, and Cause of Death 2007. Moscow, Russia: Center for Demographic Research, New Economic School (Russia). http://demogr.nes.ru/index.php/ru/demogr_indicat/data
Center for Demographic Research, New Economic School (Russia). Russia Mortality by Region, Age, Sex, and Cause of Death 2008. Moscow, Russia: Center for Demographic Research, New Economic School (Russia). http://demogr.nes.ru/index.php/ru/demogr_indicat/data
Center for Demographic Research, New Economic School (Russia). Russia Mortality by Region, Age, Sex, and Cause of Death 2009. Moscow, Russia: Center for Demographic Research, New Economic School (Russia). http://demogr.nes.ru/index.php/ru/demogr_indicat/data
Center for Demographic Research, New Economic School (Russia). Russia Mortality by Region, Age, Sex, and Cause of Death 2010. Moscow, Russia: Center for Demographic Research, New Economic School (Russia). http://demogr.nes.ru/index.php/ru/demogr_indicat/data
Center for Demographic Research, New Economic School (Russia). Russia Mortality by Region, Age, Sex, and Cause of Death 2011. Moscow, Russia: Center for Demographic Research, New Economic School (Russia). http://demogr.nes.ru/index.php/ru/demogr_indicat/data
Center for Demographic Research, New Economic School (Russia). Russia Mortality by Region, Age, Sex, and Cause of Death 2012. Moscow, Russia: Center for Demographic Research, New Economic School (Russia). http://demogr.nes.ru/index.php/ru/demogr_indicat/data
Center for Demographic Research, New Economic School (Russia). Russia Mortality by Region, Age, Sex, and Cause of Death 2013. Moscow, Russia: Center for Demographic Research, New Economic School (Russia). http://demogr.nes.ru/index.php/ru/demogr_indicat/data
Center for Demographic Research, New Economic School (Russia). Russia Mortality by Region, Age, Sex, and Cause of Death 2014. Moscow, Russia: Center for Demographic Research, New Economic School (Russia). http://demogr.nes.ru/index.php/ru/demogr_indicat/data
Center for Demographic Research, New Economic School (Russia). Russia Mortality by Region, Age, Sex, and Cause of Death 2015. Moscow, Russia: Center for Demographic Research, New Economic School (Russia). http://demogr.nes.ru/index.php/ru/demogr_indicat/data

Center for Demographic Research, New Economic School (Russia). Russia Mortality by Region, Age, Sex, and Cause of Death 2016. Moscow, Russia: Center for Demographic Research, New Economic School (Russia). http://demogr.nes.ru/index.php/ru/demogr_indicat/data
Center for Demographic Research, New Economic School (Russia). Ukraine - Republic of Crimea Mortality by Region, Age, Sex, and Cause of Death 2015. Moscow, Russian Federation: Center for Demographic Research, New Economic School (Russia).
Center for Demographic Research, New Economic School (Russia). Ukraine - Republic of Crimea Mortality by Region, Age, Sex, and Cause of Death 2016. Moscow, Russian Federation: Center for Demographic Research, New Economic School (Russia).
Center for Demographic Research, New Economic School (Russia). USSR - Russia Mortality by Region, Age, Sex, and Cause of Death 1980. Moscow, Russia: Center for Demographic Research, New Economic School (Russia). http://demogr.nes.ru/index.php/ru/demogr_indicat/data
Centers for Disease Control and Prevention (CDC), INDEPTH, International Vaccine Institute. Bangladesh - Abhoynagar, Mirsarai, and Kamalapur Health and Demographic Surveillance System.
Centers for Disease Control and Prevention (CDC), Kenya Medical Research Institute (KEMRI). Kenya KEMRI/CDC Health and Demographic Surveillance System.
Centers for Disease Control and Prevention (CDC), MEASURE Evaluation Project, Carolina Population Center, University of North Carolina, Ministry of Health (Mozambique), National Institute of Statistics (INE) (Mozambique), United States Census Bureau (USCB). Mozambique National Survey on the Causes of Death 2007-2008.
Central Statistical Office (Poland), National Institute of Public Health-National Institute of Hygiene (NIPH-NIH) (Poland). Poland Vital Registration - Causes of Death Data 1999.
Central Statistical Office (Poland), National Institute of Public Health-National Institute of Hygiene (NIPH-NIH) (Poland). Poland Vital Registration - Causes of Death Data 2000.
Central Statistical Office (Poland), National Institute of Public Health-National Institute of Hygiene (NIPH-NIH) (Poland). Poland Vital Registration - Causes of Death Data 2001.
Central Statistical Office (Poland), National Institute of Public Health-National Institute of Hygiene (NIPH-NIH) (Poland). Poland Vital Registration - Causes of Death Data 2002.
Central Statistical Office (Poland), National Institute of Public Health-National Institute of Hygiene (NIPH-NIH) (Poland). Poland Vital Registration - Causes of Death Data 2003.
Central Statistical Office (Poland), National Institute of Public Health-National Institute of Hygiene (NIPH-NIH) (Poland). Poland Vital Registration - Causes of Death Data 2004.
Central Statistical Office (Poland), National Institute of Public Health-National Institute of Hygiene (NIPH-NIH) (Poland). Poland Vital Registration - Causes of Death Data 2005.
Central Statistical Office (Poland), National Institute of Public Health-National Institute of Hygiene (NIPH-NIH) (Poland). Poland Vital Registration - Causes of Death Data 2006.
Central Statistical Office (Poland), National Institute of Public Health-National Institute of Hygiene (NIPH-NIH) (Poland). Poland Vital Registration - Causes of Death Data 2007.
Central Statistical Office (Poland), National Institute of Public Health-National Institute of Hygiene (NIPH-NIH) (Poland). Poland Vital Registration - Causes of Death Data 2008.
Central Statistical Office (Poland), National Institute of Public Health-National Institute of Hygiene (NIPH-NIH) (Poland). Poland Vital Registration - Causes of Death Data 2009.
Central Statistical Office (Poland), National Institute of Public Health-National Institute of Hygiene (NIPH-NIH) (Poland). Poland Vital Registration - Causes of Death Data 2010.
Central Statistical Office (Poland), National Institute of Public Health-National Institute of Hygiene (NIPH-NIH) (Poland). Poland Vital Registration - Causes of Death Data 2011.
Central Statistical Office (Poland), National Institute of Public Health-National Institute of Hygiene (NIPH-NIH) (Poland). Poland Vital Registration - Causes of Death Data 2012.
Central Statistical Office (Poland), National Institute of Public Health-National Institute of Hygiene (NIPH-NIH) (Poland). Poland Vital Registration - Causes of Death Data 2013.
Central Statistical Office (Poland), National Institute of Public Health-National Institute of Hygiene (NIPH-NIH) (Poland). Poland Vital Registration - Causes of Death Data 2014.
Central Statistical Office (Poland), National Institute of Public Health-National Institute of Hygiene (NIPH-NIH) (Poland). Poland Vital Registration - Causes of Death Data 2015.
Central Statistical Office (Poland), National Institute of Public Health-National Institute of Hygiene (NIPH-NIH) (Poland). Poland Vital Registration - Causes of Death Data 2016.
Central Statistical Office (Poland), National Institute of Public Health-National Institute of Hygiene (NIPH-NIH) (Poland). Poland Vital Registration - Causes of Death Data 2017.
Central Statistical Office (Poland), National Institute of Public Health-National Institute of Hygiene (NIPH-NIH) (Poland). Poland Vital Registration - Causes of Death Data 2018.
Central Statistical Office (Poland), National Institute of Public Health-National Institute of Hygiene (NIPH-NIH) (Poland). Poland Vital Registration - Causes of Death Data 2019.
Central Statistical Office (Poland), National Institute of Public Health-National Institute of Hygiene (NIPH-NIH) (Poland). Poland Vital Registration - Death Data 1980.
Central Statistical Office (Poland), National Institute of Public Health-National Institute of Hygiene (NIPH-NIH) (Poland). Poland Vital Registration - Death Data 1981.
Central Statistical Office (Poland), National Institute of Public Health-National Institute of Hygiene (NIPH-NIH) (Poland). Poland Vital Registration - Death Data 1982.
Central Statistical Office (Poland), National Institute of Public Health-National Institute of Hygiene (NIPH-NIH) (Poland). Poland Vital Registration - Death Data 1983.
Central Statistical Office (Poland), National Institute of Public Health-National Institute of Hygiene (NIPH-NIH) (Poland). Poland Vital Registration - Death Data 1984.

Central Statistical Office (Poland), National Institute of Public Health-National Institute of Hygiene (NIPH-NIH) (Poland). Poland Vital Registration - Death Data 1985.
Central Statistical Office (Poland), National Institute of Public Health-National Institute of Hygiene (NIPH-NIH) (Poland). Poland Vital Registration - Death Data 1986.
Central Statistical Office (Poland), National Institute of Public Health-National Institute of Hygiene (NIPH-NIH) (Poland). Poland Vital Registration - Death Data 1987.
Central Statistical Office (Poland), National Institute of Public Health-National Institute of Hygiene (NIPH-NIH) (Poland). Poland Vital Registration - Death Data 1988.
Central Statistical Office (Poland), National Institute of Public Health-National Institute of Hygiene (NIPH-NIH) (Poland). Poland Vital Registration - Death Data 1989.
Central Statistical Office (Poland), National Institute of Public Health-National Institute of Hygiene (NIPH-NIH) (Poland). Poland Vital Registration - Death Data 1990.
Central Statistical Office (Poland), National Institute of Public Health-National Institute of Hygiene (NIPH-NIH) (Poland). Poland Vital Registration - Death Data 1991.
Central Statistical Office (Poland), National Institute of Public Health-National Institute of Hygiene (NIPH-NIH) (Poland). Poland Vital Registration - Death Data 1992.
Central Statistical Office (Poland), National Institute of Public Health-National Institute of Hygiene (NIPH-NIH) (Poland). Poland Vital Registration - Death Data 1993.
Central Statistical Office (Poland), National Institute of Public Health-National Institute of Hygiene (NIPH-NIH) (Poland). Poland Vital Registration - Death Data 1994.
Central Statistical Office (Poland), National Institute of Public Health-National Institute of Hygiene (NIPH-NIH) (Poland). Poland Vital Registration - Death Data 1995.
Central Statistical Office (Poland), National Institute of Public Health-National Institute of Hygiene (NIPH-NIH) (Poland). Poland Vital Registration - Death Data 1996.
Chief Medical Office of Greenland. Greenland Vital Registration - Deaths 1995.
Chief Medical Office of Greenland. Greenland Vital Registration - Deaths 1996.
Chief Medical Office of Greenland. Greenland Vital Registration - Deaths 1997.
Chief Medical Office of Greenland. Greenland Vital Registration - Deaths 1998.
Chief Medical Office of Greenland. Greenland Vital Registration - Deaths 1999.
Chief Medical Office of Greenland. Greenland Vital Registration - Deaths 2000.
Chief Medical Office of Greenland. Greenland Vital Registration - Deaths 2001.
Chief Medical Office of Greenland. Greenland Vital Registration - Deaths 2002.
Chief Medical Office of Greenland. Greenland Vital Registration - Deaths 2003.
Chief Medical Office of Greenland. Greenland Vital Registration - Deaths 2004.
Chief Medical Office of Greenland. Greenland Vital Registration - Deaths 2005.
Chief Medical Office of Greenland. Greenland Vital Registration - Deaths 2006.
Chief Medical Office of Greenland. Greenland Vital Registration - Deaths 2007.
Chief Medical Office of Greenland. Greenland Vital Registration - Deaths 2008.
Chief Medical Office of Greenland. Greenland Vital Registration - Deaths 2009.
Chief Medical Office of Greenland. Greenland Vital Registration - Deaths 2010.
Chief Medical Office of Greenland. Greenland Vital Registration - Deaths 2011.
Chief Medical Office of Greenland. Greenland Vital Registration - Deaths 2012.
Chief Medical Office of Greenland. Greenland Vital Registration - Deaths 2013.
Chief Medical Office of Greenland. Greenland Vital Registration - Deaths 2014.
Chief Medical Office of Greenland. Greenland Vital Registration - Deaths 2015.
Chile Vital Registration - Deaths 1980 ICD9.
Chile Vital Registration - Deaths 1981 ICD9.
Chile Vital Registration - Deaths 1982 ICD9.
Chile Vital Registration - Deaths 1983 ICD9.
Chile Vital Registration - Deaths 1984 ICD9.
Chile Vital Registration - Deaths 1986 ICD9.
Chile Vital Registration - Deaths 1987 ICD9.
Chile Vital Registration - Deaths 1988 ICD9.
Chile Vital Registration - Deaths 1989 ICD9.
Chile Vital Registration - Deaths 1997 ICD10.
Chile Vital Registration - Deaths 1998 ICD10.
Chile Vital Registration - Deaths 1999 ICD10.
Chile Vital Registration - Deaths 2000 ICD10.
Chile Vital Registration - Deaths 2001 ICD10.
Chile Vital Registration - Deaths 2002 ICD10.
Chile Vital Registration - Deaths 2003 ICD10.
Chile Vital Registration - Deaths 2004 ICD10.
Chile Vital Registration - Deaths 2005 ICD10.
Chile Vital Registration - Deaths 2006 ICD10.

Chile Vital Registration - Deaths 2007 ICD10.
Chile Vital Registration - Deaths 2008 ICD10.
Chile Vital Registration - Deaths 2009 ICD10.
Chile Vital Registration - Deaths 2010 ICD10.
Chile Vital Registration - Deaths 2011 ICD10.
Chile Vital Registration - Deaths 2012 ICD10.
Chile Vital Registration - Deaths 2013 ICD10.
Chile Vital Registration - Deaths 2014 ICD10.
Chile Vital Registration - Deaths 2015 ICD10.
Chile Vital Registration - Deaths 2016 ICD10.
Chile Vital Registration - Deaths 2017 ICD10.
Chile Vital Registration - Deaths 2018 ICD10.
Chinese Center for Disease Control and Prevention (CCDC). China Disease Surveillance Points 1991 - China CDC.
Chinese Center for Disease Control and Prevention (CCDC). China Disease Surveillance Points 1992 - China CDC.
Chinese Center for Disease Control and Prevention (CCDC). China Disease Surveillance Points 1993 - China CDC.
Chinese Center for Disease Control and Prevention (CCDC). China Disease Surveillance Points 1994 - China CDC.
Chinese Center for Disease Control and Prevention (CCDC). China Disease Surveillance Points 1995 - China CDC.
Chinese Center for Disease Control and Prevention (CCDC). China Disease Surveillance Points 1996 - China CDC.
Chinese Center for Disease Control and Prevention (CCDC). China Disease Surveillance Points 1997 - China CDC.
Chinese Center for Disease Control and Prevention (CCDC). China Disease Surveillance Points 1998 - China CDC.
Chinese Center for Disease Control and Prevention (CCDC). China Disease Surveillance Points 1999 - China CDC.
Chinese Center for Disease Control and Prevention (CCDC). China Disease Surveillance Points 2000 - China CDC.
Chinese Center for Disease Control and Prevention (CCDC). China Disease Surveillance Points 2001 - China CDC.
Chinese Center for Disease Control and Prevention (CCDC). China Disease Surveillance Points 2002 - China CDC.
Chinese Center for Disease Control and Prevention (CCDC). China Disease Surveillance Points 2004 - China CDC.
Chinese Center for Disease Control and Prevention (CCDC). China Disease Surveillance Points 2005 - China CDC.
Chinese Center for Disease Control and Prevention (CCDC). China Disease Surveillance Points 2006 - China CDC.
Chinese Center for Disease Control and Prevention (CCDC). China Disease Surveillance Points 2007 - China CDC.
Chinese Center for Disease Control and Prevention (CCDC). China Disease Surveillance Points 2013 - China CDC.
Chinese Center for Disease Control and Prevention (CCDC). China Disease Surveillance Points 2014 - China CDC.
Chinese Center for Disease Control and Prevention (CCDC). China Disease Surveillance Points 2015 - China CDC.
Chinese Center for Disease Control and Prevention (CCDC). China Disease Surveillance Points 2016 - China CDC.
Chinese Center for Disease Control and Prevention (CCDC). China Disease Surveillance Points 2017 - China CDC.
Chinese Center for Disease Control and Prevention (CCDC). China Disease Surveillance Points and Death Registration 2008 - China CDC.
Chinese Center for Disease Control and Prevention (CCDC). China Disease Surveillance Points and Death Registration 2009 - China CDC.
Chinese Center for Disease Control and Prevention (CCDC). China Disease Surveillance Points and Death Registration 2010 - China CDC.
Chinese Center for Disease Control and Prevention (CCDC). China Disease Surveillance Points and Death Registration 2011 - China CDC.
Chinese Center for Disease Control and Prevention (CCDC). China Disease Surveillance Points and Death Registration 2012 - China CDC.
Colombia Vital Registration - Deaths 1997 ICD10.
Colombia Vital Registration - Deaths 1998 ICD10.
Colombia Vital Registration - Deaths 1999 ICD10.
Colombia Vital Registration - Deaths 2000 ICD10.
Colombia Vital Registration - Deaths 2001 ICD10.
Colombia Vital Registration - Deaths 2002 ICD10.
Colombia Vital Registration - Deaths 2003 ICD10.
Colombia Vital Registration - Deaths 2004 ICD10.
Colombia Vital Registration - Deaths 2005 ICD10.
Colombia Vital Registration - Deaths 2006 ICD10.
Colombia Vital Registration - Deaths 2007 ICD10.
Colombia Vital Registration - Deaths 2008 ICD10.
Colombia Vital Registration - Deaths 2009 ICD10.
Colombia Vital Registration - Deaths 2010 ICD10.
Colombia Vital Registration - Deaths 2011 ICD10.
Colombia Vital Registration - Deaths 2012 ICD10.
Colombia Vital Registration - Deaths 2013 ICD10.
Colombia Vital Registration - Deaths 2014 ICD10.
Colombia Vital Registration - Deaths 2015 ICD10.
Cook Islands Statistics Office, Ministry of Health (Cook Islands). Cook Islands Vital Registration - Causes of Death 2000.
Cook Islands Statistics Office, Ministry of Health (Cook Islands). Cook Islands Vital Registration - Causes of Death 2001.
Cook Islands Statistics Office, Ministry of Health (Cook Islands). Cook Islands Vital Registration - Causes of Death 2002.
Cook Islands Statistics Office, Ministry of Health (Cook Islands). Cook Islands Vital Registration - Causes of Death 2003.

Cook Islands Statistics Office, Ministry of Health (Cook Islands). Cook Islands Vital Registration - Causes of Death 2004.
Cook Islands Statistics Office, Ministry of Health (Cook Islands). Cook Islands Vital Registration - Causes of Death 2005.
Cook Islands Statistics Office, Ministry of Health (Cook Islands). Cook Islands Vital Registration - Causes of Death 2006.
Cook Islands Statistics Office, Ministry of Health (Cook Islands). Cook Islands Vital Registration - Causes of Death 2007.
Cook Islands Statistics Office, Ministry of Health (Cook Islands). Cook Islands Vital Registration - Causes of Death 2009.
Cook Islands Statistics Office, Ministry of Health (Cook Islands). Cook Islands Vital Registration - Causes of Death 2010.
Cook Islands Statistics Office, Ministry of Health (Cook Islands). Cook Islands Vital Registration - Causes of Death 2011.
Cook Islands Statistics Office, Ministry of Health (Cook Islands). Cook Islands Vital Registration - Causes of Death 2012.
Cook Islands Statistics Office, Ministry of Health (Cook Islands). Cook Islands Vital Registration - Causes of Death 2013.
Cook Islands Statistics Office, Ministry of Health (Cook Islands). Cook Islands Vital Registration - Causes of Death 2014.
Cook Islands Statistics Office, Ministry of Health (Cook Islands). Cook Islands Vital Registration - Causes of Death 2015.
Cook Islands Statistics Office, Ministry of Health (Cook Islands). Cook Islands Vital Registration - Causes of Death 2016.
Costa Rica Vital Registration - Deaths 1980 ICD9.
Costa Rica Vital Registration - Deaths 1981 ICD9.
Costa Rica Vital Registration - Deaths 1982 ICD9.
Costa Rica Vital Registration - Deaths 1983 ICD9.
Costa Rica Vital Registration - Deaths 1984 ICD9.
Costa Rica Vital Registration - Deaths 1985 ICD9.
Costa Rica Vital Registration - Deaths 1986 ICD9.
Costa Rica Vital Registration - Deaths 1987 ICD9.
Costa Rica Vital Registration - Deaths 1988 ICD9.
Costa Rica Vital Registration - Deaths 1989 ICD9.
Costa Rica Vital Registration - Deaths 1990 ICD9.
Costa Rica Vital Registration - Deaths 1991 ICD9.
Costa Rica Vital Registration - Deaths 1992 ICD9.
Costa Rica Vital Registration - Deaths 1993 ICD9.
Costa Rica Vital Registration - Deaths 1994 ICD9.
Costa Rica Vital Registration - Deaths 1995 ICD9.
Costa Rica Vital Registration - Deaths 1996 ICD9.
Costa Rica Vital Registration - Deaths 1997 ICD10.
Costa Rica Vital Registration - Deaths 1998 ICD10.
Costa Rica Vital Registration - Deaths 1999 ICD10.
Costa Rica Vital Registration - Deaths 2000 ICD10.
Costa Rica Vital Registration - Deaths 2001 ICD10.
Costa Rica Vital Registration - Deaths 2002 ICD10.
Costa Rica Vital Registration - Deaths 2003 ICD10.
Costa Rica Vital Registration - Deaths 2004 ICD10.
Costa Rica Vital Registration - Deaths 2005 ICD10.
Costa Rica Vital Registration - Deaths 2006 ICD10.
Costa Rica Vital Registration - Deaths 2007 ICD10.
Costa Rica Vital Registration - Deaths 2008 ICD10.
Costa Rica Vital Registration - Deaths 2009 ICD10.
Costa Rica Vital Registration - Deaths 2010 ICD10.
Costa Rica Vital Registration - Deaths 2011 ICD10.
Costa Rica Vital Registration - Deaths 2012 ICD10.
Costa Rica Vital Registration - Deaths 2013 ICD10.
Costa Rica Vital Registration - Deaths 2014 ICD10.
Croatia Vital Registration - Deaths 1991 ICD9.
Croatia Vital Registration - Deaths 1992 ICD9.
Croatia Vital Registration - Deaths 1993 ICD9.
Croatia Vital Registration - Deaths 1994 ICD9.
Croatia Vital Registration - Deaths 1995 ICD10.
Croatia Vital Registration - Deaths 1996 ICD10.
Croatia Vital Registration - Deaths 1997 ICD10.
Croatia Vital Registration - Deaths 1998 ICD10.
Croatia Vital Registration - Deaths 1999 ICD10.
Croatia Vital Registration - Deaths 2000 ICD10.
Croatia Vital Registration - Deaths 2001 ICD10.
Croatia Vital Registration - Deaths 2002 ICD10.
Croatia Vital Registration - Deaths 2003 ICD10.
Croatia Vital Registration - Deaths 2004 ICD10.

Croatia Vital Registration - Deaths 2005 ICD10.
Croatia Vital Registration - Deaths 2006 ICD10.
Croatia Vital Registration - Deaths 2007 ICD10.
Croatia Vital Registration - Deaths 2008 ICD10.
Croatia Vital Registration - Deaths 2009 ICD10.
Croatia Vital Registration - Deaths 2010 ICD10.
Croatia Vital Registration - Deaths 2011 ICD10.
Croatia Vital Registration - Deaths 2012 ICD10.
Croatia Vital Registration - Deaths 2013 ICD10.
Croatia Vital Registration - Deaths 2014 ICD10.
Croatia Vital Registration - Deaths 2015 ICD10.
Croatia Vital Registration - Deaths 2016 ICD10.
Croatia Vital Registration - Deaths 2017 ICD10.
Cuba Vital Registration - Deaths 1980 ICD9.
Cuba Vital Registration - Deaths 1981 ICD9.
Cuba Vital Registration - Deaths 1982 ICD9.
Cuba Vital Registration - Deaths 1983 ICD9.
Cuba Vital Registration - Deaths 1984 ICD9.
Cuba Vital Registration - Deaths 1985 ICD9.
Cuba Vital Registration - Deaths 1986 ICD9.
Cuba Vital Registration - Deaths 1987 ICD9.
Cuba Vital Registration - Deaths 1988 ICD9.
Cuba Vital Registration - Deaths 1989 ICD9.
Cuba Vital Registration - Deaths 1990 ICD9.
Cuba Vital Registration - Deaths 1991 ICD9.
Cuba Vital Registration - Deaths 1992 ICD9.
Cuba Vital Registration - Deaths 1993 ICD9.
Cuba Vital Registration - Deaths 1994 ICD9.
Cuba Vital Registration - Deaths 1995 ICD9.
Cuba Vital Registration - Deaths 1996 ICD9.
Cuba Vital Registration - Deaths 1997 ICD9.
Cuba Vital Registration - Deaths 1998 ICD9.
Cuba Vital Registration - Deaths 1999 ICD9.
Cuba Vital Registration - Deaths 2000 ICD9.
Cuba Vital Registration - Deaths 2001 ICD10.
Cuba Vital Registration - Deaths 2002 ICD10.
Cuba Vital Registration - Deaths 2003 ICD10.
Cuba Vital Registration - Deaths 2004 ICD10.
Cuba Vital Registration - Deaths 2005 ICD10.
Cuba Vital Registration - Deaths 2006 ICD10.
Cuba Vital Registration - Deaths 2007 ICD10.
Cuba Vital Registration - Deaths 2008 ICD10.
Cuba Vital Registration - Deaths 2009 ICD10.
Cuba Vital Registration - Deaths 2010 ICD10.
Cuba Vital Registration - Deaths 2011 ICD10.
Cuba Vital Registration - Deaths 2012 ICD10.
Cuba Vital Registration - Deaths 2013 ICD10.
Cuba Vital Registration - Deaths 2014 ICD10.
Cuba Vital Registration - Deaths 2015 ICD10.
Cuba Vital Registration - Deaths 2016 ICD10.
Cuba Vital Registration - Deaths 2017 ICD10.
Cyprus Vital Registration - Deaths 2004 ICD10.
Cyprus Vital Registration - Deaths 2005 ICD10.
Cyprus Vital Registration - Deaths 2006 ICD10.
Cyprus Vital Registration - Deaths 2007 ICD10.
Cyprus Vital Registration - Deaths 2008 ICD10.
Cyprus Vital Registration - Deaths 2009 ICD10.
Cyprus Vital Registration - Deaths 2010 ICD10.
Cyprus Vital Registration - Deaths 2011 ICD10.
Cyprus Vital Registration - Deaths 2012 ICD10.
Cyprus Vital Registration - Deaths 2013 ICD10.

Cyprus Vital Registration - Deaths 2014 ICD10.
Cyprus Vital Registration - Deaths 2015 ICD10.
Cyprus Vital Registration - Deaths 2016 ICD10.
Cyprus Vital Registration - Deaths 2017 ICD10.
Cyprus Vital Registration - Deaths 2018 ICD10.
Czech Republic Vital Registration - Deaths 1993 ICD9.
Czech Republic Vital Registration - Deaths 1994 ICD10.
Czech Republic Vital Registration - Deaths 1995 ICD10.
Czech Republic Vital Registration - Deaths 1996 ICD10.
Czech Republic Vital Registration - Deaths 1997 ICD10.
Czech Republic Vital Registration - Deaths 1998 ICD10.
Czech Republic Vital Registration - Deaths 1999 ICD10.
Czech Republic Vital Registration - Deaths 2000 ICD10.
Czech Republic Vital Registration - Deaths 2001 ICD10.
Czech Republic Vital Registration - Deaths 2002 ICD10.
Czech Republic Vital Registration - Deaths 2003 ICD10.
Czech Republic Vital Registration - Deaths 2004 ICD10.
Czech Republic Vital Registration - Deaths 2005 ICD10.
Czech Republic Vital Registration - Deaths 2006 ICD10.
Czech Republic Vital Registration - Deaths 2007 ICD10.
Czech Republic Vital Registration - Deaths 2008 ICD10.
Czech Republic Vital Registration - Deaths 2009 ICD10.
Czech Republic Vital Registration - Deaths 2010 ICD10.
Czech Republic Vital Registration - Deaths 2011 ICD10.
Czech Republic Vital Registration - Deaths 2012 ICD10.
Czech Republic Vital Registration - Deaths 2013 ICD10.
Czech Republic Vital Registration - Deaths 2014 ICD10.
Czech Republic Vital Registration - Deaths 2015 ICD10.
Czech Republic Vital Registration - Deaths 2016 ICD10.
Czech Republic Vital Registration - Deaths 2017 ICD10.
Czech Republic Vital Registration - Deaths 2018 ICD10.
Czechoslovakia - Czech Republic Vital Registration - Deaths 1986 ICD9.
Czechoslovakia - Czech Republic Vital Registration - Deaths 1987 ICD9.
Czechoslovakia - Czech Republic Vital Registration - Deaths 1988 ICD9.
Czechoslovakia - Czech Republic Vital Registration - Deaths 1989 ICD9.
Czechoslovakia - Czech Republic Vital Registration - Deaths 1990 ICD9.
Czechoslovakia - Czech Republic Vital Registration - Deaths 1991 ICD9.
Czechoslovakia - Czech Republic Vital Registration - Deaths 1992 ICD9.
Denmark Vital Registration - Deaths 1994 ICD10.
Denmark Vital Registration - Deaths 1995 ICD10.
Denmark Vital Registration - Deaths 1996 ICD10.
Denmark Vital Registration - Deaths 1997 ICD10.
Denmark Vital Registration - Deaths 1998 ICD10.
Denmark Vital Registration - Deaths 1999 ICD10.
Denmark Vital Registration - Deaths 2000 ICD10.
Denmark Vital Registration - Deaths 2001 ICD10.
Denmark Vital Registration - Deaths 2002 ICD10.
Denmark Vital Registration - Deaths 2003 ICD10.
Denmark Vital Registration - Deaths 2004 ICD10.
Denmark Vital Registration - Deaths 2005 ICD10.
Denmark Vital Registration - Deaths 2006 ICD10.
Denmark Vital Registration - Deaths 2007 ICD10.
Denmark Vital Registration - Deaths 2008 ICD10.
Denmark Vital Registration - Deaths 2009 ICD10.
Denmark Vital Registration - Deaths 2010 ICD10.
Denmark Vital Registration - Deaths 2011 ICD10.
Denmark Vital Registration - Deaths 2012 ICD10.
Denmark Vital Registration - Deaths 2013 ICD10.
Denmark Vital Registration - Deaths 2014 ICD10.
Denmark Vital Registration - Deaths 2015 ICD10.
Denmark Vital Registration - Deaths 2016 ICD10.

Denmark Vital Registration - Deaths 2017 ICD10.
Denmark Vital Registration - Deaths 2018 ICD10.
Department of Census and Statistics (Sri Lanka). Sri Lanka Vital Statistics - Births and Deaths 2010. Colombo, Sri Lanka: Department of Census and Statistics (Sri Lanka).
Department of Census and Statistics (Sri Lanka). Sri Lanka Vital Statistics - Deaths 2009-2010. Colombo, Sri Lanka: Department of Census and Statistics (Sri Lanka).
Department of Health (Taiwan). Taiwan Vital Registration - Deaths 1980.
Department of Health (Taiwan). Taiwan Vital Registration - Deaths 1981.
Department of Health (Taiwan). Taiwan Vital Registration - Deaths 1982.
Department of Health (Taiwan). Taiwan Vital Registration - Deaths 1983.
Department of Health (Taiwan). Taiwan Vital Registration - Deaths 1984.
Department of Health (Taiwan). Taiwan Vital Registration - Deaths 1985.
Department of Health (Taiwan). Taiwan Vital Registration - Deaths 1986.
Department of Health (Taiwan). Taiwan Vital Registration - Deaths 1987.
Department of Health (Taiwan). Taiwan Vital Registration - Deaths 1988.
Department of Health (Taiwan). Taiwan Vital Registration - Deaths 1989.
Department of Health (Taiwan). Taiwan Vital Registration - Deaths 1990.
Department of Health (Taiwan). Taiwan Vital Registration - Deaths 1991.
Department of Health (Taiwan). Taiwan Vital Registration - Deaths 1992. Taipei City, Taiwan (Province of China): Ministry of Health and Welfare (Taiwan).
Department of Health (Taiwan). Taiwan Vital Registration - Deaths 1993. Taipei City, Taiwan (Province of China): Ministry of Health and Welfare (Taiwan).
Department of Health (Taiwan). Taiwan Vital Registration - Deaths 1994. Taipei City, Taiwan (Province of China): Ministry of Health and Welfare (Taiwan).
Department of Health (Taiwan). Taiwan Vital Registration - Deaths 1995. Taipei City, Taiwan (Province of China): Ministry of Health and Welfare (Taiwan).
Department of Health (Taiwan). Taiwan Vital Registration - Deaths 1996. Taipei City, Taiwan (Province of China): Ministry of Health and Welfare (Taiwan).
Department of Health (Taiwan). Taiwan Vital Registration - Deaths 1997. Taipei City, Taiwan (Province of China): Ministry of Health and Welfare (Taiwan).
Department of Health (Taiwan). Taiwan Vital Registration - Deaths 1998. Taipei City, Taiwan (Province of China): Ministry of Health and Welfare (Taiwan).
Department of Health (Taiwan). Taiwan Vital Registration - Deaths 1999. Taipei City, Taiwan (Province of China): Ministry of Health and Welfare (Taiwan).
Department of Health (Taiwan). Taiwan Vital Registration - Deaths 2000. Taipei City, Taiwan (Province of China): Ministry of Health and Welfare (Taiwan).
Department of Health (Taiwan). Taiwan Vital Registration - Deaths 2001. Taipei City, Taiwan (Province of China): Ministry of Health and Welfare (Taiwan).
Department of Health (Taiwan). Taiwan Vital Registration - Deaths 2002. Taipei City, Taiwan (Province of China): Ministry of Health and Welfare (Taiwan).
Department of Health (Taiwan). Taiwan Vital Registration - Deaths 2003. Taipei City, Taiwan (Province of China): Ministry of Health and Welfare (Taiwan).
Department of Health (Taiwan). Taiwan Vital Registration - Deaths 2004. Taipei City, Taiwan (Province of China): Ministry of Health and Welfare (Taiwan).
Department of Health (Taiwan). Taiwan Vital Registration - Deaths 2005. Taipei City, Taiwan (Province of China): Ministry of Health and Welfare (Taiwan).
Department of Health (Taiwan). Taiwan Vital Registration - Deaths 2006. Taipei City, Taiwan (Province of China): Ministry of Health and Welfare (Taiwan).
Department of Health (Taiwan). Taiwan Vital Registration - Deaths 2007. Taipei City, Taiwan (Province of China): Ministry of Health and Welfare (Taiwan).
Department of Health (Taiwan). Taiwan Vital Registration - Deaths 2008. Taipei City, Taiwan (Province of China): Ministry of Health and Welfare (Taiwan).
Department of Health (Taiwan). Taiwan Vital Registration - Deaths 2009. Taipei City, Taiwan (Province of China): Ministry of Health and Welfare (Taiwan).
Department of Health (Taiwan). Taiwan Vital Registration - Deaths 2010. Taipei City, Taiwan (Province of China): Ministry of Health and Welfare (Taiwan).
Department of Health (Taiwan). Taiwan Vital Registration - Deaths 2011. Taipei City, Taiwan (Province of China): Ministry of Health and Welfare (Taiwan).
Department of Health (Taiwan). Taiwan Vital Registration - Deaths 2012. Taipei City, Taiwan (Province of China): Ministry of Health and Welfare (Taiwan).
Department of Public Health (Palau), Ministry of Health (Palau), Office of Planning and Statistics (Palau). Palau Vital Statistics - Causes of Death 2013.
Department of Statistics and Health Information, Ministry of Health (Chile), National Institute of Statistics (Chile). Chile Vital Statistics - Deaths 1990. Santiago, Chile: Department of Statistics and Health Information, Ministry of Health (Chile).
Department of Statistics and Health Information, Ministry of Health (Chile), National Institute of Statistics (Chile). Chile Vital Statistics - Deaths 1991. Santiago, Chile: Department of Statistics and Health Information, Ministry of Health (Chile).

Department of Statistics and Health Information, Ministry of Health (Chile), National Institute of Statistics (Chile). Chile Vital Statistics - Deaths 1992. Santiago, Chile: Department of Statistics and Health Information, Ministry of Health (Chile).
Department of Statistics and Health Information, Ministry of Health (Chile), National Institute of Statistics (Chile). Chile Vital Statistics - Deaths 1993. Santiago, Chile: Department of Statistics and Health Information, Ministry of Health (Chile).
Department of Statistics and Health Information, Ministry of Health (Chile), National Institute of Statistics (Chile). Chile Vital Statistics - Deaths 1994. Santiago, Chile: Department of Statistics and Health Information, Ministry of Health (Chile).
Department of Statistics and Health Information, Ministry of Health (Chile), National Institute of Statistics (Chile). Chile Vital Statistics - Deaths 1995. Santiago, Chile: Department of Statistics and Health Information, Ministry of Health (Chile).
Department of Statistics and Health Information, Ministry of Health (Chile), National Institute of Statistics (Chile). Chile Vital Statistics - Deaths 1996. Santiago, Chile: Department of Statistics and Health Information, Ministry of Health (Chile).
Directorate of Health (Iceland). Iceland Causes of Death Register 2017.
Directorate of Health (Iceland). Iceland Causes of Death Register 2018.
Directorate of Health (Iceland). Iceland Causes of Death Register 2019.
Directorate of Health (Iceland). Iceland Causes of Death Register 2020.
Dominica Vital Registration - Deaths 1980 ICD9.
Dominica Vital Registration - Deaths 1981 ICD9.
Dominica Vital Registration - Deaths 1982 ICD9.
Dominica Vital Registration - Deaths 1983 ICD9.
Dominica Vital Registration - Deaths 1984 ICD9.
Dominica Vital Registration - Deaths 1985 ICD9.
Dominica Vital Registration - Deaths 1986 ICD9.
Dominica Vital Registration - Deaths 1987 ICD9.
Dominica Vital Registration - Deaths 1988 ICD9.
Dominica Vital Registration - Deaths 1989 ICD9.
Dominica Vital Registration - Deaths 1990 ICD9.
Dominica Vital Registration - Deaths 1991 ICD9.
Dominica Vital Registration - Deaths 1992 ICD9.
Dominica Vital Registration - Deaths 1993 ICD9.
Dominica Vital Registration - Deaths 1994 ICD9.
Dominica Vital Registration - Deaths 1995 ICD9.
Dominica Vital Registration - Deaths 1996 ICD9.
Dominica Vital Registration - Deaths 1997 ICD9.
Dominica Vital Registration - Deaths 1998 ICD9.
Dominica Vital Registration - Deaths 1999 ICD9.
Dominica Vital Registration - Deaths 2000 ICD9.
Dominica Vital Registration - Deaths 2001 ICD10.
Dominica Vital Registration - Deaths 2002 ICD10.
Dominica Vital Registration - Deaths 2003 ICD10.
Dominica Vital Registration - Deaths 2004 ICD10.
Dominica Vital Registration - Deaths 2005 ICD10.
Dominica Vital Registration - Deaths 2006 ICD10.
Dominica Vital Registration - Deaths 2007 ICD10.
Dominica Vital Registration - Deaths 2008 ICD10.
Dominica Vital Registration - Deaths 2009 ICD10.
Dominica Vital Registration - Deaths 2010 ICD10.
Dominica Vital Registration - Deaths 2011 ICD10.
Dominica Vital Registration - Deaths 2012 ICD10.
Dominica Vital Registration - Deaths 2013 ICD10.
Dominica Vital Registration - Deaths 2014 ICD10.
Dominica Vital Registration - Deaths 2015 ICD10.
Dominican Republic Vital Registration - Deaths 1981 ICD9.
Dominican Republic Vital Registration - Deaths 1982 ICD9.
Dominican Republic Vital Registration - Deaths 1983 ICD9.
Dominican Republic Vital Registration - Deaths 1984 ICD9.
Dominican Republic Vital Registration - Deaths 1985 ICD9.
Dominican Republic Vital Registration - Deaths 1986 ICD9.
Dominican Republic Vital Registration - Deaths 1987 ICD9.
Dominican Republic Vital Registration - Deaths 1988 ICD9.
Dominican Republic Vital Registration - Deaths 1989 ICD9.
Dominican Republic Vital Registration - Deaths 1990 ICD9.
Dominican Republic Vital Registration - Deaths 1991 ICD9.
Dominican Republic Vital Registration - Deaths 1992 ICD9.

Dominican Republic Vital Registration - Deaths 1994 ICD9.
Dominican Republic Vital Registration - Deaths 1995 ICD9.
Dominican Republic Vital Registration - Deaths 1996 ICD10.
Dominican Republic Vital Registration - Deaths 1997 ICD10.
Dominican Republic Vital Registration - Deaths 1998 ICD10.
Dominican Republic Vital Registration - Deaths 1999 ICD10.
Dominican Republic Vital Registration - Deaths 2000 ICD10.
Dominican Republic Vital Registration - Deaths 2001 ICD10.
Dominican Republic Vital Registration - Deaths 2003 ICD10.
Dominican Republic Vital Registration - Deaths 2004 ICD10.
Dominican Republic Vital Registration - Deaths 2005 ICD10.
Dominican Republic Vital Registration - Deaths 2010 ICD10.
Dominican Republic Vital Registration - Deaths 2011 ICD10.
Dominican Republic Vital Registration - Deaths 2012 ICD10.
Dominican Republic Vital Registration - Deaths 2013 ICD10.
Ecuador Vital Registration - Deaths 1980 ICD9.
Ecuador Vital Registration - Deaths 1981 ICD9.
Ecuador Vital Registration - Deaths 1982 ICD9.
Ecuador Vital Registration - Deaths 1983 ICD9.
Ecuador Vital Registration - Deaths 1984 ICD9.
Ecuador Vital Registration - Deaths 1985 ICD9.
Ecuador Vital Registration - Deaths 1986 ICD9.
Ecuador Vital Registration - Deaths 1987 ICD9.
Ecuador Vital Registration - Deaths 1988 ICD9.
Ecuador Vital Registration - Deaths 1989 ICD9.
Ecuador Vital Registration - Deaths 1990 ICD9.
Ecuador Vital Registration - Deaths 1991 ICD9.
Ecuador Vital Registration - Deaths 1992 ICD9.
Ecuador Vital Registration - Deaths 1993 ICD9.
Ecuador Vital Registration - Deaths 1994 ICD9.
Ecuador Vital Registration - Deaths 1995 ICD9.
Ecuador Vital Registration - Deaths 1996 ICD9.
Ecuador Vital Registration - Deaths 1997 ICD10.
Ecuador Vital Registration - Deaths 1998 ICD10.
Ecuador Vital Registration - Deaths 1999 ICD10.
Ecuador Vital Registration - Deaths 2000 ICD10.
Ecuador Vital Registration - Deaths 2001 ICD10.
Ecuador Vital Registration - Deaths 2002 ICD10.
Ecuador Vital Registration - Deaths 2003 ICD10.
Ecuador Vital Registration - Deaths 2004 ICD10.
Ecuador Vital Registration - Deaths 2005 ICD10.
Ecuador Vital Registration - Deaths 2006 ICD10.
Ecuador Vital Registration - Deaths 2007 ICD10.
Ecuador Vital Registration - Deaths 2008 ICD10.
Ecuador Vital Registration - Deaths 2009 ICD10.
Ecuador Vital Registration - Deaths 2010 ICD10.
Ecuador Vital Registration - Deaths 2011 ICD10.
Ecuador Vital Registration - Deaths 2012 ICD10.
Ecuador Vital Registration - Deaths 2013 ICD10.
Ecuador Vital Registration - Deaths 2014 ICD10.
Ecuador Vital Registration - Deaths 2015 ICD10.
Ecuador Vital Registration - Deaths 2016 ICD10.
Egypt Vital Registration - Deaths 1991 ICD9.
Egypt Vital Registration - Deaths 1992 ICD9.
Egypt Vital Registration - Deaths 2016 ICD10.
Egypt Vital Registration - Deaths 2017 ICD10.
Egypt Vital Registration - Deaths 2018 ICD10.
El Salvador Vital Registration - Deaths 1981 ICD9.
El Salvador Vital Registration - Deaths 1982 ICD9.
El Salvador Vital Registration - Deaths 1983 ICD9.
El Salvador Vital Registration - Deaths 1984 ICD9.

El Salvador Vital Registration - Deaths 1990 ICD9.
El Salvador Vital Registration - Deaths 1991 ICD9.
El Salvador Vital Registration - Deaths 1992 ICD9.
El Salvador Vital Registration - Deaths 1993 ICD9.
El Salvador Vital Registration - Deaths 1995 ICD9.
El Salvador Vital Registration - Deaths 1996 ICD9.
El Salvador Vital Registration - Deaths 1997 ICD10.
El Salvador Vital Registration - Deaths 1998 ICD10.
El Salvador Vital Registration - Deaths 1999 ICD10.
El Salvador Vital Registration - Deaths 2000 ICD10.
El Salvador Vital Registration - Deaths 2001 ICD10.
El Salvador Vital Registration - Deaths 2002 ICD10.
El Salvador Vital Registration - Deaths 2003 ICD10.
El Salvador Vital Registration - Deaths 2004 ICD10.
El Salvador Vital Registration - Deaths 2005 ICD10.
El Salvador Vital Registration - Deaths 2006 ICD10.
El Salvador Vital Registration - Deaths 2007 ICD10.
El Salvador Vital Registration - Deaths 2008 ICD10.
El Salvador Vital Registration - Deaths 2009 ICD10.
El Salvador Vital Registration - Deaths 2010 ICD10.
El Salvador Vital Registration - Deaths 2011 ICD10.
El Salvador Vital Registration - Deaths 2012 ICD10.
El Salvador Vital Registration - Deaths 2013 ICD10.
El Salvador Vital Registration - Deaths 2014 ICD10.
El Salvador Vital Registration - Deaths 2015 ICD10.
Estonia Vital Registration - Deaths 1981 ICD9.
Estonia Vital Registration - Deaths 1982 ICD9.
Estonia Vital Registration - Deaths 1985 ICD9.
Estonia Vital Registration - Deaths 1986 ICD9.
Estonia Vital Registration - Deaths 1987 ICD9.
Estonia Vital Registration - Deaths 1988 ICD9.
Estonia Vital Registration - Deaths 1989 ICD9.
Estonia Vital Registration - Deaths 1990 ICD9.
Estonia Vital Registration - Deaths 1991 ICD9.
Estonia Vital Registration - Deaths 1992 ICD9.
Estonia Vital Registration - Deaths 1993 ICD9.
Estonia Vital Registration - Deaths 1994 ICD9.
Estonia Vital Registration - Deaths 1995 ICD9.
Estonia Vital Registration - Deaths 1996 ICD9.
Estonia Vital Registration - Deaths 1997 ICD10.
Estonia Vital Registration - Deaths 1998 ICD10.
Estonia Vital Registration - Deaths 1999 ICD10.
Estonia Vital Registration - Deaths 2000 ICD10.
Estonia Vital Registration - Deaths 2001 ICD10.
Estonia Vital Registration - Deaths 2002 ICD10.
Estonia Vital Registration - Deaths 2003 ICD10.
Estonia Vital Registration - Deaths 2004 ICD10.
Estonia Vital Registration - Deaths 2005 ICD10.
Estonia Vital Registration - Deaths 2006 ICD10.
Estonia Vital Registration - Deaths 2007 ICD10.
Estonia Vital Registration - Deaths 2008 ICD10.
Estonia Vital Registration - Deaths 2009 ICD10.
Estonia Vital Registration - Deaths 2010 ICD10.
Estonia Vital Registration - Deaths 2011 ICD10.
Estonia Vital Registration - Deaths 2012 ICD10.
Estonia Vital Registration - Deaths 2013 ICD10.
Estonia Vital Registration - Deaths 2014 ICD10.
Estonia Vital Registration - Deaths 2015 ICD10.
Estonia Vital Registration - Deaths 2016 ICD10.
Estonia Vital Registration - Deaths 2017 ICD10.
Estonia Vital Registration - Deaths 2018 ICD10.

Fiji Vital Registration - Deaths 1999 ICD9.
Fiji Vital Registration - Deaths 2002 ICD10.
Fiji Vital Registration - Deaths 2003 ICD10.
Fiji Vital Registration - Deaths 2004 ICD10.
Fiji Vital Registration - Deaths 2005 ICD10.
Fiji Vital Registration - Deaths 2006 ICD10.
Fiji Vital Registration - Deaths 2007 ICD10.
Fiji Vital Registration - Deaths 2008 ICD10.
Fiji Vital Registration - Deaths 2009 ICD10.
Fiji Vital Registration - Deaths 2011 ICD10.
Fiji Vital Registration - Deaths 2012 ICD10.
Finland Vital Registration - Deaths 1987 ICD9.
Finland Vital Registration - Deaths 1988 ICD9.
Finland Vital Registration - Deaths 1989 ICD9.
Finland Vital Registration - Deaths 1990 ICD9.
Finland Vital Registration - Deaths 1991 ICD9.
Finland Vital Registration - Deaths 1992 ICD9.
Finland Vital Registration - Deaths 1993 ICD9.
Finland Vital Registration - Deaths 1994 ICD9.
Finland Vital Registration - Deaths 1995 ICD9.
Finland Vital Registration - Deaths 1996 ICD10.
Finland Vital Registration - Deaths 1997 ICD10.
Finland Vital Registration - Deaths 1998 ICD10.
Finland Vital Registration - Deaths 1999 ICD10.
Finland Vital Registration - Deaths 2000 ICD10.
Finland Vital Registration - Deaths 2001 ICD10.
Finland Vital Registration - Deaths 2002 ICD10.
Finland Vital Registration - Deaths 2003 ICD10.
Finland Vital Registration - Deaths 2004 ICD10.
Finland Vital Registration - Deaths 2005 ICD10.
Finland Vital Registration - Deaths 2006 ICD10.
Finland Vital Registration - Deaths 2007 ICD10.
Finland Vital Registration - Deaths 2008 ICD10.
Finland Vital Registration - Deaths 2009 ICD10.
Finland Vital Registration - Deaths 2010 ICD10.
Finland Vital Registration - Deaths 2011 ICD10.
Finland Vital Registration - Deaths 2012 ICD10.
Finland Vital Registration - Deaths 2013 ICD10.
Finland Vital Registration - Deaths 2014 ICD10.
Finland Vital Registration - Deaths 2015 ICD10.
Finland Vital Registration - Deaths 2016 ICD10.
Finland Vital Registration - Deaths 2017 ICD10.
Finland Vital Registration - Deaths 2018 ICD10.
France Vital Registration - Deaths 1980 ICD9.
France Vital Registration - Deaths 1981 ICD9.
France Vital Registration - Deaths 1982 ICD9.
France Vital Registration - Deaths 1983 ICD9.
France Vital Registration - Deaths 1984 ICD9.
France Vital Registration - Deaths 1985 ICD9.
France Vital Registration - Deaths 1986 ICD9.
France Vital Registration - Deaths 1987 ICD9.
France Vital Registration - Deaths 1988 ICD9.
France Vital Registration - Deaths 1989 ICD9.
France Vital Registration - Deaths 1990 ICD9.
France Vital Registration - Deaths 1991 ICD9.
France Vital Registration - Deaths 1992 ICD9.
France Vital Registration - Deaths 1993 ICD9.
France Vital Registration - Deaths 1994 ICD9.
France Vital Registration - Deaths 1995 ICD9.
France Vital Registration - Deaths 1996 ICD9.
France Vital Registration - Deaths 1997 ICD9.

France Vital Registration - Deaths 1998 ICD9.
France Vital Registration - Deaths 1999 ICD9.
France Vital Registration - Deaths 2000 ICD10.
France Vital Registration - Deaths 2001 ICD10.
France Vital Registration - Deaths 2002 ICD10.
France Vital Registration - Deaths 2003 ICD10.
France Vital Registration - Deaths 2004 ICD10.
France Vital Registration - Deaths 2005 ICD10.
France Vital Registration - Deaths 2006 ICD10.
France Vital Registration - Deaths 2007 ICD10.
France Vital Registration - Deaths 2008 ICD10.
France Vital Registration - Deaths 2009 ICD10.
France Vital Registration - Deaths 2010 ICD10.
France Vital Registration - Deaths 2011 ICD10.
France Vital Registration - Deaths 2012 ICD10.
France Vital Registration - Deaths 2013 ICD10.
France Vital Registration - Deaths 2014 ICD10.
France Vital Registration - Deaths 2015 ICD10.
France Vital Registration - Deaths 2016 ICD10.
General Directorate of Strategic Health Information (DIGIES) (Paraguay), Ministry of Health (Paraguay). Paraguay Vital Registration - Deaths 2004.
General Directorate of Strategic Health Information (DIGIES) (Paraguay), Ministry of Health (Paraguay). Paraguay Vital Registration - Deaths 2005.
General Directorate of Strategic Health Information (DIGIES) (Paraguay), Ministry of Health (Paraguay). Paraguay Vital Registration - Deaths 2006.
General Directorate of Strategic Health Information (DIGIES) (Paraguay), Ministry of Health (Paraguay). Paraguay Vital Registration - Deaths 2007.
General Directorate of Strategic Health Information (DIGIES) (Paraguay), Ministry of Health (Paraguay). Paraguay Vital Registration - Deaths 2008.
General Directorate of Strategic Health Information (DIGIES) (Paraguay), Ministry of Health (Paraguay). Paraguay Vital Registration - Deaths 2009.
General Directorate of Strategic Health Information (DIGIES) (Paraguay), Ministry of Health (Paraguay). Paraguay Vital Registration - Deaths 2010.
General Directorate of Strategic Health Information (DIGIES) (Paraguay), Ministry of Health (Paraguay). Paraguay Vital Registration - Deaths 2011.
General Directorate of Strategic Health Information (DIGIES) (Paraguay), Ministry of Health (Paraguay). Paraguay Vital Registration - Deaths 2012.
General Directorate of Strategic Health Information (DIGIES) (Paraguay), Ministry of Health (Paraguay). Paraguay Vital Registration - Deaths 2013.
General Directorate of Strategic Health Information (DIGIES) (Paraguay), Ministry of Health (Paraguay). Paraguay Vital Registration - Deaths 2014.
General Directorate of Strategic Health Information (DIGIES) (Paraguay), Ministry of Health (Paraguay). Paraguay Vital Registration - Deaths 2015.
General Directorate of Strategic Health Information (DIGIES) (Paraguay), Ministry of Health (Paraguay). Paraguay Vital Registration - Deaths 2016.
General Directorate of Strategic Health Information (DIGIES) (Paraguay), Ministry of Health (Paraguay). Paraguay Vital Registration - Deaths 2017.
Georgia Vital Registration - Deaths 1981 ICD9.
Georgia Vital Registration - Deaths 1982 ICD9.
Georgia Vital Registration - Deaths 1985 ICD9.
Georgia Vital Registration - Deaths 1986 ICD9.
Georgia Vital Registration - Deaths 1987 ICD9.
Georgia Vital Registration - Deaths 1988 ICD9.
Georgia Vital Registration - Deaths 1989 ICD9.
Georgia Vital Registration - Deaths 1990 ICD9.
Georgia Vital Registration - Deaths 1991 ICD9.
Georgia Vital Registration - Deaths 1992 ICD9.
Georgia Vital Registration - Deaths 1994 ICD9.
Georgia Vital Registration - Deaths 1995 ICD9.
Georgia Vital Registration - Deaths 1996 ICD9.
Georgia Vital Registration - Deaths 1997 ICD9.
Georgia Vital Registration - Deaths 1998 ICD10.
Georgia Vital Registration - Deaths 1999 ICD10.
Georgia Vital Registration - Deaths 2000 ICD10.

Georgia Vital Registration - Deaths 2001 ICD10.
Georgia Vital Registration - Deaths 2004 ICD10.
Georgia Vital Registration - Deaths 2005 ICD10.
Georgia Vital Registration - Deaths 2006 ICD10.
Georgia Vital Registration - Deaths 2007 ICD10.
Georgia Vital Registration - Deaths 2009 ICD10.
Georgia Vital Registration - Deaths 2012 ICD10.
Georgia Vital Registration - Deaths 2013 ICD10.
Georgia Vital Registration - Deaths 2014 ICD10.
Georgia Vital Registration - Deaths 2015 ICD10.
Georgia Vital Registration - Deaths 2016 ICD10.
Georgia Vital Registration - Deaths 2017 ICD10.
Georgia Vital Registration - Deaths 2018 ICD10.
Germany Vital Registration - Deaths 1991 ICD9.
Germany Vital Registration - Deaths 1992 ICD9.
Germany Vital Registration - Deaths 1993 ICD9.
Germany Vital Registration - Deaths 1994 ICD9.
Germany Vital Registration - Deaths 1995 ICD9.
Germany Vital Registration - Deaths 1996 ICD9.
Germany Vital Registration - Deaths 1997 ICD9.
Germany Vital Registration - Deaths 1998 ICD10.
Germany Vital Registration - Deaths 1999 ICD10.
Germany Vital Registration - Deaths 2000 ICD10.
Germany Vital Registration - Deaths 2001 ICD10.
Germany Vital Registration - Deaths 2002 ICD10.
Germany Vital Registration - Deaths 2003 ICD10.
Germany Vital Registration - Deaths 2004 ICD10.
Germany Vital Registration - Deaths 2005 ICD10.
Germany Vital Registration - Deaths 2006 ICD10.
Germany Vital Registration - Deaths 2007 ICD10.
Germany Vital Registration - Deaths 2008 ICD10.
Germany Vital Registration - Deaths 2009 ICD10.
Germany Vital Registration - Deaths 2010 ICD10.
Germany Vital Registration - Deaths 2011 ICD10.
Germany Vital Registration - Deaths 2012 ICD10.
Germany Vital Registration - Deaths 2013 ICD10.
Germany Vital Registration - Deaths 2014 ICD10.
Germany Vital Registration - Deaths 2015 ICD10.
Germany Vital Registration - Deaths 2016 ICD10.
Germany Vital Registration - Deaths 2017 ICD10.
Germany Vital Registration - Deaths 2018 ICD10.
Germany, Former Democratic Republic Vital Registration - Deaths 1980 ICD9.
Germany, Former Democratic Republic Vital Registration - Deaths 1981 ICD9.
Germany, Former Democratic Republic Vital Registration - Deaths 1982 ICD9.
Germany, Former Democratic Republic Vital Registration - Deaths 1983 ICD9.
Germany, Former Democratic Republic Vital Registration - Deaths 1984 ICD9.
Germany, Former Democratic Republic Vital Registration - Deaths 1985 ICD9.
Germany, Former Democratic Republic Vital Registration - Deaths 1986 ICD9.
Germany, Former Democratic Republic Vital Registration - Deaths 1987 ICD9.
Germany, Former Democratic Republic Vital Registration - Deaths 1988 ICD9.
Germany, Former Democratic Republic Vital Registration - Deaths 1989 ICD9.
Germany, Former Democratic Republic Vital Registration - Deaths 1990 ICD9.
Germany, Former Federal Republic Vital Registration - Deaths 1980 ICD9.
Germany, Former Federal Republic Vital Registration - Deaths 1981 ICD9.
Germany, Former Federal Republic Vital Registration - Deaths 1982 ICD9.
Germany, Former Federal Republic Vital Registration - Deaths 1983 ICD9.
Germany, Former Federal Republic Vital Registration - Deaths 1984 ICD9.
Germany, Former Federal Republic Vital Registration - Deaths 1985 ICD9.
Germany, Former Federal Republic Vital Registration - Deaths 1986 ICD9.
Germany, Former Federal Republic Vital Registration - Deaths 1987 ICD9.
Germany, Former Federal Republic Vital Registration - Deaths 1988 ICD9.

Germany, Former Federal Republic Vital Registration - Deaths 1989 ICD9.
Germany, Former Federal Republic Vital Registration - Deaths 1990 ICD9.
Greece Vital Registration - Deaths 1980 ICD9.
Greece Vital Registration - Deaths 1981 ICD9.
Greece Vital Registration - Deaths 1982 ICD9.
Greece Vital Registration - Deaths 1983 ICD9.
Greece Vital Registration - Deaths 1984 ICD9.
Greece Vital Registration - Deaths 1985 ICD9.
Greece Vital Registration - Deaths 1986 ICD9.
Greece Vital Registration - Deaths 1987 ICD9.
Greece Vital Registration - Deaths 1988 ICD9.
Greece Vital Registration - Deaths 1989 ICD9.
Greece Vital Registration - Deaths 1990 ICD9.
Greece Vital Registration - Deaths 1991 ICD9.
Greece Vital Registration - Deaths 1992 ICD9.
Greece Vital Registration - Deaths 1993 ICD9.
Greece Vital Registration - Deaths 1994 ICD9.
Greece Vital Registration - Deaths 1995 ICD9.
Greece Vital Registration - Deaths 1996 ICD9.
Greece Vital Registration - Deaths 1997 ICD9.
Greece Vital Registration - Deaths 1998 ICD9.
Greece Vital Registration - Deaths 1999 ICD9.
Greece Vital Registration - Deaths 2000 ICD9.
Greece Vital Registration - Deaths 2001 ICD9.
Greece Vital Registration - Deaths 2002 ICD9.
Greece Vital Registration - Deaths 2003 ICD9.
Greece Vital Registration - Deaths 2004 ICD9.
Greece Vital Registration - Deaths 2005 ICD9.
Greece Vital Registration - Deaths 2006 ICD9.
Greece Vital Registration - Deaths 2007 ICD9.
Greece Vital Registration - Deaths 2008 ICD9.
Greece Vital Registration - Deaths 2009 ICD9.
Greece Vital Registration - Deaths 2010 ICD9.
Greece Vital Registration - Deaths 2011 ICD9.
Greece Vital Registration - Deaths 2012 ICD9.
Greece Vital Registration - Deaths 2013 ICD9.
Greece Vital Registration - Deaths 2014 ICD10.
Greece Vital Registration - Deaths 2015 ICD10.
Greece Vital Registration - Deaths 2016 ICD10.
Greece Vital Registration - Deaths 2017 ICD10.
Grenada Vital Registration - Deaths 1984 ICD9.
Grenada Vital Registration - Deaths 1985 ICD9.
Grenada Vital Registration - Deaths 1988 ICD9.
Grenada Vital Registration - Deaths 1989 ICD9.
Grenada Vital Registration - Deaths 1990 ICD9.
Grenada Vital Registration - Deaths 1991 ICD9.
Grenada Vital Registration - Deaths 1993 ICD9.
Grenada Vital Registration - Deaths 1994 ICD9.
Grenada Vital Registration - Deaths 1995 ICD9.
Grenada Vital Registration - Deaths 1996 ICD9.
Grenada Vital Registration - Deaths 2001 ICD10.
Grenada Vital Registration - Deaths 2002 ICD10.
Grenada Vital Registration - Deaths 2003 ICD10.
Grenada Vital Registration - Deaths 2004 ICD10.
Grenada Vital Registration - Deaths 2005 ICD10.
Grenada Vital Registration - Deaths 2006 ICD10.
Grenada Vital Registration - Deaths 2007 ICD10.
Grenada Vital Registration - Deaths 2008 ICD10.
Grenada Vital Registration - Deaths 2009 ICD10.
Grenada Vital Registration - Deaths 2010 ICD10.
Grenada Vital Registration - Deaths 2011 ICD10.

Grenada Vital Registration - Deaths 2012 ICD10.
Grenada Vital Registration - Deaths 2013 ICD10.
Grenada Vital Registration - Deaths 2014 ICD10.
Grenada Vital Registration - Deaths 2015 ICD10.
Grenada Vital Registration - Deaths 2016 ICD10.
Grenada Vital Registration - Deaths 2017 ICD10.
Grenada Vital Registration - Deaths 2018 ICD10.
Guatemala Vital Registration - Deaths 1980 ICD9.
Guatemala Vital Registration - Deaths 1981 ICD9.
Guatemala Vital Registration - Deaths 1984 ICD9.
Guatemala Vital Registration - Deaths 1986 ICD9.
Guatemala Vital Registration - Deaths 1987 ICD9.
Guatemala Vital Registration - Deaths 1988 ICD9.
Guatemala Vital Registration - Deaths 1989 ICD9.
Guatemala Vital Registration - Deaths 1990 ICD9.
Guatemala Vital Registration - Deaths 1991 ICD9.
Guatemala Vital Registration - Deaths 1992 ICD9.
Guatemala Vital Registration - Deaths 1993 ICD9.
Guatemala Vital Registration - Deaths 1994 ICD9.
Guatemala Vital Registration - Deaths 1995 ICD9.
Guatemala Vital Registration - Deaths 1996 ICD9.
Guatemala Vital Registration - Deaths 1997 ICD9.
Guatemala Vital Registration - Deaths 1998 ICD9.
Guatemala Vital Registration - Deaths 1999 ICD9.
Guatemala Vital Registration - Deaths 2000 ICD9.
Guatemala Vital Registration - Deaths 2001 ICD9.
Guatemala Vital Registration - Deaths 2002 ICD9.
Guatemala Vital Registration - Deaths 2003 ICD9.
Guatemala Vital Registration - Deaths 2004 ICD9.
Guatemala Vital Registration - Deaths 2005 ICD10.
Guatemala Vital Registration - Deaths 2006 ICD10.
Guatemala Vital Registration - Deaths 2007 ICD10.
Guatemala Vital Registration - Deaths 2008 ICD10.
Guatemala Vital Registration - Deaths 2009 ICD10.
Guyana Vital Registration - Deaths 1984 ICD9.
Guyana Vital Registration - Deaths 1988 ICD9.
Guyana Vital Registration - Deaths 1989 ICD9.
Guyana Vital Registration - Deaths 1990 ICD9.
Guyana Vital Registration - Deaths 1991 ICD9.
Guyana Vital Registration - Deaths 1992 ICD9.
Guyana Vital Registration - Deaths 1993 ICD9.
Guyana Vital Registration - Deaths 1994 ICD9.
Guyana Vital Registration - Deaths 1995 ICD9.
Guyana Vital Registration - Deaths 1996 ICD9.
Guyana Vital Registration - Deaths 1997 ICD9.
Guyana Vital Registration - Deaths 1998 ICD9.
Guyana Vital Registration - Deaths 1999 ICD9.
Guyana Vital Registration - Deaths 2000 ICD10 - PAHO.
Guyana Vital Registration - Deaths 2001 ICD10.
Guyana Vital Registration - Deaths 2002 ICD10.
Guyana Vital Registration - Deaths 2003 ICD10.
Guyana Vital Registration - Deaths 2004 ICD10.
Guyana Vital Registration - Deaths 2005 ICD10.
Guyana Vital Registration - Deaths 2006 ICD10.
Guyana Vital Registration - Deaths 2007 ICD10.
Guyana Vital Registration - Deaths 2008 ICD10.
Guyana Vital Registration - Deaths 2009 ICD10.
Guyana Vital Registration - Deaths 2010 ICD10.
Guyana Vital Registration - Deaths 2011 ICD10.
Guyana Vital Registration - Deaths 2012 ICD10.
Guyana Vital Registration - Deaths 2013 ICD10.

Guyana Vital Registration - Deaths 2014 ICD10.
Higher Population Council (Jordan), Ministry of Health (Jordan), Royal Medical Services (Jordan), United Nations Relief and Works Agency (UNRWA). Jordan National Maternal Mortality Study 2008.
Hoa NP, Rao C, Hoy DG, Hinh ND, Chuc NT, Ngo DA. Mortality measures from sample-based surveillance: evidence of the epidemiological transition in Viet Nam. Bull World Health Organ. 2012; 90(10): 764-72. [Unpublished data].
Honduras Vital Registration - Deaths 1987 ICD9.
Honduras Vital Registration - Deaths 1988 ICD9.
Honduras Vital Registration - Deaths 1990 ICD9.
Hong Kong Vital Registration - Deaths 1980 ICD9.
Hong Kong Vital Registration - Deaths 1981 ICD9.
Hong Kong Vital Registration - Deaths 1982 ICD9.
Hong Kong Vital Registration - Deaths 1983 ICD9.
Hong Kong Vital Registration - Deaths 1984 ICD9.
Hong Kong Vital Registration - Deaths 1985 ICD9.
Hong Kong Vital Registration - Deaths 1986 ICD9.
Hong Kong Vital Registration - Deaths 1987 ICD9.
Hong Kong Vital Registration - Deaths 1988 ICD9.
Hong Kong Vital Registration - Deaths 1989 ICD9.
Hong Kong Vital Registration - Deaths 1990 ICD9.
Hong Kong Vital Registration - Deaths 1991 ICD9.
Hong Kong Vital Registration - Deaths 1992 ICD9.
Hong Kong Vital Registration - Deaths 1993 ICD9.
Hong Kong Vital Registration - Deaths 1994 ICD9.
Hong Kong Vital Registration - Deaths 1995 ICD9.
Hong Kong Vital Registration - Deaths 1996 ICD9.
Hong Kong Vital Registration - Deaths 1997 ICD9.
Hong Kong Vital Registration - Deaths 1998 ICD9.
Hong Kong Vital Registration - Deaths 1999 ICD9.
Hong Kong Vital Registration - Deaths 2000 ICD9.
Hong Kong Vital Registration - Deaths 2001 ICD10.
Hong Kong Vital Registration - Deaths 2002 ICD10.
Hong Kong Vital Registration - Deaths 2003 ICD10.
Hong Kong Vital Registration - Deaths 2004 ICD10.
Hong Kong Vital Registration - Deaths 2005 ICD10.
Hong Kong Vital Registration - Deaths 2006 ICD10.
Hong Kong Vital Registration - Deaths 2007 ICD10.
Hong Kong Vital Registration - Deaths 2008 ICD10.
Hong Kong Vital Registration - Deaths 2009 ICD10.
Hong Kong Vital Registration - Deaths 2010 ICD10.
Hong Kong Vital Registration - Deaths 2011 ICD10.
Hong Kong Vital Registration - Deaths 2012 ICD10.
Hong Kong Vital Registration - Deaths 2013 ICD10.
Hong Kong Vital Registration - Deaths 2014 ICD10.
Hong Kong Vital Registration - Deaths 2015 ICD10.
Hong Kong Vital Registration - Deaths 2016 ICD10.
Hong Kong Vital Registration - Deaths 2017 ICD10.
Hungary Vital Registration - Deaths 1980 ICD9.
Hungary Vital Registration - Deaths 1981 ICD9.
Hungary Vital Registration - Deaths 1982 ICD9.
Hungary Vital Registration - Deaths 1983 ICD9.
Hungary Vital Registration - Deaths 1984 ICD9.
Hungary Vital Registration - Deaths 1985 ICD9.
Hungary Vital Registration - Deaths 1986 ICD9.
Hungary Vital Registration - Deaths 1987 ICD9.
Hungary Vital Registration - Deaths 1988 ICD9.
Hungary Vital Registration - Deaths 1989 ICD9.
Hungary Vital Registration - Deaths 1990 ICD9.
Hungary Vital Registration - Deaths 1991 ICD9.
Hungary Vital Registration - Deaths 1992 ICD9.
Hungary Vital Registration - Deaths 1993 ICD9.
Hungary Vital Registration - Deaths 1994 ICD9.

Hungary Vital Registration - Deaths 1995 ICD9.
Hungary Vital Registration - Deaths 1996 ICD10.
Hungary Vital Registration - Deaths 1997 ICD10.
Hungary Vital Registration - Deaths 1998 ICD10.
Hungary Vital Registration - Deaths 1999 ICD10.
Hungary Vital Registration - Deaths 2000 ICD10.
Hungary Vital Registration - Deaths 2001 ICD10.
Hungary Vital Registration - Deaths 2002 ICD10.
Hungary Vital Registration - Deaths 2003 ICD10.
Hungary Vital Registration - Deaths 2004 ICD10.
Hungary Vital Registration - Deaths 2005 ICD10.
Hungary Vital Registration - Deaths 2006 ICD10.
Hungary Vital Registration - Deaths 2007 ICD10.
Hungary Vital Registration - Deaths 2008 ICD10.
Hungary Vital Registration - Deaths 2009 ICD10.
Hungary Vital Registration - Deaths 2010 ICD10.
Hungary Vital Registration - Deaths 2011 ICD10.
Hungary Vital Registration - Deaths 2012 ICD10.
Hungary Vital Registration - Deaths 2013 ICD10.
Hungary Vital Registration - Deaths 2014 ICD10.
Hungary Vital Registration - Deaths 2015 ICD10.
Hungary Vital Registration - Deaths 2016 ICD10.
Hungary Vital Registration - Deaths 2017 ICD10.
Hungary Vital Registration - Deaths 2018 ICD10.
Iceland Vital Registration - Deaths 1981 ICD9.
Iceland Vital Registration - Deaths 1982 ICD9.
Iceland Vital Registration - Deaths 1983 ICD9.
Iceland Vital Registration - Deaths 1984 ICD9.
Iceland Vital Registration - Deaths 1985 ICD9.
Iceland Vital Registration - Deaths 1986 ICD9.
Iceland Vital Registration - Deaths 1987 ICD9.
Iceland Vital Registration - Deaths 1988 ICD9.
Iceland Vital Registration - Deaths 1989 ICD9.
Iceland Vital Registration - Deaths 1990 ICD9.
Iceland Vital Registration - Deaths 1991 ICD9.
Iceland Vital Registration - Deaths 1992 ICD9.
Iceland Vital Registration - Deaths 1993 ICD9.
Iceland Vital Registration - Deaths 1994 ICD9.
Iceland Vital Registration - Deaths 1995 ICD9.
Iceland Vital Registration - Deaths 1996 ICD10.
Iceland Vital Registration - Deaths 1997 ICD10.
Iceland Vital Registration - Deaths 1998 ICD10.
Iceland Vital Registration - Deaths 1999 ICD10.
Iceland Vital Registration - Deaths 2000 ICD10.
Iceland Vital Registration - Deaths 2001 ICD10.
Iceland Vital Registration - Deaths 2002 ICD10.
Iceland Vital Registration - Deaths 2003 ICD10.
Iceland Vital Registration - Deaths 2004 ICD10.
Iceland Vital Registration - Deaths 2005 ICD10.
Iceland Vital Registration - Deaths 2006 ICD10.
Iceland Vital Registration - Deaths 2007 ICD10.
Iceland Vital Registration - Deaths 2008 ICD10.
Iceland Vital Registration - Deaths 2009 ICD10.
Iceland Vital Registration - Deaths 2010 ICD10.
Iceland Vital Registration - Deaths 2011 ICD10.
Iceland Vital Registration - Deaths 2012 ICD10.
Iceland Vital Registration - Deaths 2013 ICD10.
Iceland Vital Registration - Deaths 2014 ICD10.
Iceland Vital Registration - Deaths 2015 ICD10.
Iceland Vital Registration - Deaths 2016 ICD10.

ICF International, Ministry of Health (Nepal), New ERA. Nepal Demographic and Health Survey 2016-2017. Fairfax, United States of America: ICF International, 2017.
INDEPTH, International Centre for Diarrhoeal Disease Research, Bangladesh (ICDDR,B). Bangladesh - Matlab Health and Demographic Surveillance System.
INDEPTH, Manhica Health Research Center (CISM). Mozambique - ManhiÃ§a Health and Demographic Surveillance System.
INDEPTH, Ministry of Health and Social Welfare (Tanzania), School of Public Health, Columbia University. Tanzania - Rufiji Health and Demographic Surveillance System.
INDEPTH, Nouna Health Research Center (CRSN) (Burkina Faso). Burkina Faso - Nouna Health and Demographic Surveillance System.
INDEPTH. Tanzania - Ifakara Health and Demographic Surveillance System.
Indian Council of Medical Research (ICMR). India Study on Causes of Death by Verbal Autopsy 2003.
International Centre for Diarrhoeal Disease Research, Bangladesh (ICDDR,B). Bangladesh - Chandpur and Comilla District Verbal Autopsy Study 2011-2014.
Iraq Vital Registration - Deaths 2008 ICD10.
Iraq Vital Registration - Deaths 2016 ICD10.
Ireland Vital Registration - Deaths 1980 ICD9.
Ireland Vital Registration - Deaths 1981 ICD9.
Ireland Vital Registration - Deaths 1982 ICD9.
Ireland Vital Registration - Deaths 1983 ICD9.
Ireland Vital Registration - Deaths 1984 ICD9.
Ireland Vital Registration - Deaths 1985 ICD9.
Ireland Vital Registration - Deaths 1986 ICD9.
Ireland Vital Registration - Deaths 1987 ICD9.
Ireland Vital Registration - Deaths 1988 ICD9.
Ireland Vital Registration - Deaths 1989 ICD9.
Ireland Vital Registration - Deaths 1990 ICD9.
Ireland Vital Registration - Deaths 1991 ICD9.
Ireland Vital Registration - Deaths 1992 ICD9.
Ireland Vital Registration - Deaths 1993 ICD9.
Ireland Vital Registration - Deaths 1994 ICD9.
Ireland Vital Registration - Deaths 1995 ICD9.
Ireland Vital Registration - Deaths 1996 ICD9.
Ireland Vital Registration - Deaths 1997 ICD9.
Ireland Vital Registration - Deaths 1998 ICD9.
Ireland Vital Registration - Deaths 1999 ICD9.
Ireland Vital Registration - Deaths 2000 ICD9.
Ireland Vital Registration - Deaths 2001 ICD9.
Ireland Vital Registration - Deaths 2002 ICD9.
Ireland Vital Registration - Deaths 2003 ICD9.
Ireland Vital Registration - Deaths 2004 ICD9.
Ireland Vital Registration - Deaths 2005 ICD9.
Ireland Vital Registration - Deaths 2006 ICD9.
Ireland Vital Registration - Deaths 2007 ICD10.
Ireland Vital Registration - Deaths 2008 ICD10.
Ireland Vital Registration - Deaths 2009 ICD10.
Ireland Vital Registration - Deaths 2010 ICD10.
Ireland Vital Registration - Deaths 2011 ICD10.
Ireland Vital Registration - Deaths 2012 ICD10.
Ireland Vital Registration - Deaths 2013 ICD10.
Ireland Vital Registration - Deaths 2014 ICD10.
Ireland Vital Registration - Deaths 2015 ICD10.
Israel Vital Registration - Deaths 1980 ICD9.
Israel Vital Registration - Deaths 1981 ICD9.
Israel Vital Registration - Deaths 1982 ICD9.
Israel Vital Registration - Deaths 1983 ICD9.
Israel Vital Registration - Deaths 1984 ICD9.
Israel Vital Registration - Deaths 1985 ICD9.
Israel Vital Registration - Deaths 1986 ICD9.
Israel Vital Registration - Deaths 1987 ICD9.
Israel Vital Registration - Deaths 1988 ICD9.
Israel Vital Registration - Deaths 1989 ICD9.
Israel Vital Registration - Deaths 1990 ICD9.
Israel Vital Registration - Deaths 1991 ICD9.

Israel Vital Registration - Deaths 1992 ICD9.
Israel Vital Registration - Deaths 1993 ICD9.
Israel Vital Registration - Deaths 1994 ICD9.
Israel Vital Registration - Deaths 1995 ICD9.
Israel Vital Registration - Deaths 1996 ICD9.
Israel Vital Registration - Deaths 1997 ICD9.
Israel Vital Registration - Deaths 1998 ICD10.
Israel Vital Registration - Deaths 1999 ICD10.
Israel Vital Registration - Deaths 2000 ICD10.
Israel Vital Registration - Deaths 2001 ICD10.
Israel Vital Registration - Deaths 2002 ICD10.
Israel Vital Registration - Deaths 2003 ICD10.
Israel Vital Registration - Deaths 2004 ICD10.
Israel Vital Registration - Deaths 2005 ICD10.
Israel Vital Registration - Deaths 2006 ICD10.
Israel Vital Registration - Deaths 2007 ICD10.
Israel Vital Registration - Deaths 2008 ICD10.
Israel Vital Registration - Deaths 2009 ICD10.
Israel Vital Registration - Deaths 2010 ICD10.
Israel Vital Registration - Deaths 2011 ICD10.
Israel Vital Registration - Deaths 2012 ICD10.
Israel Vital Registration - Deaths 2013 ICD10.
Israel Vital Registration - Deaths 2014 ICD10.
Israel Vital Registration - Deaths 2015 ICD10.
Israel Vital Registration - Deaths 2017 ICD10.
Israel Vital Registration - Deaths 2018 ICD10.
Italian National Institute of Statistics. Italy Vital Registration - Deaths 1980. Rome, Italy: Italian National Institute of Statistics.
Italian National Institute of Statistics. Italy Vital Registration - Deaths 1981. Rome, Italy: Italian National Institute of Statistics.
Italian National Institute of Statistics. Italy Vital Registration - Deaths 1982. Rome, Italy: Italian National Institute of Statistics.
Italian National Institute of Statistics. Italy Vital Registration - Deaths 1983. Rome, Italy: Italian National Institute of Statistics.
Italian National Institute of Statistics. Italy Vital Registration - Deaths 1984. Rome, Italy: Italian National Institute of Statistics.
Italian National Institute of Statistics. Italy Vital Registration - Deaths 1985. Rome, Italy: Italian National Institute of Statistics.
Italian National Institute of Statistics. Italy Vital Registration - Deaths 1986. Rome, Italy: Italian National Institute of Statistics.
Italian National Institute of Statistics. Italy Vital Registration - Deaths 1987. Rome, Italy: Italian National Institute of Statistics.
Italian National Institute of Statistics. Italy Vital Registration - Deaths 1988. Rome, Italy: Italian National Institute of Statistics.
Italian National Institute of Statistics. Italy Vital Registration - Deaths 1989. Rome, Italy: Italian National Institute of Statistics.
Italian National Institute of Statistics. Italy Vital Registration - Deaths 1990. Rome, Italy: Italian National Institute of Statistics.
Italian National Institute of Statistics. Italy Vital Registration - Deaths 1991. Rome, Italy: Italian National Institute of Statistics.
Italian National Institute of Statistics. Italy Vital Registration - Deaths 1992. Rome, Italy: Italian National Institute of Statistics.
Italian National Institute of Statistics. Italy Vital Registration - Deaths 1993. Rome, Italy: Italian National Institute of Statistics.
Italian National Institute of Statistics. Italy Vital Registration - Deaths 1994. Rome, Italy: Italian National Institute of Statistics.
Italian National Institute of Statistics. Italy Vital Registration - Deaths 1995. Rome, Italy: Italian National Institute of Statistics.
Italian National Institute of Statistics. Italy Vital Registration - Deaths 1996. Rome, Italy: Italian National Institute of Statistics.
Italian National Institute of Statistics. Italy Vital Registration - Deaths 1997. Rome, Italy: Italian National Institute of Statistics.
Italian National Institute of Statistics. Italy Vital Registration - Deaths 1998. Rome, Italy: Italian National Institute of Statistics.
Italian National Institute of Statistics. Italy Vital Registration - Deaths 1999. Rome, Italy: Italian National Institute of Statistics.
Italian National Institute of Statistics. Italy Vital Registration - Deaths 2000. Rome, Italy: Italian National Institute of Statistics.
Italian National Institute of Statistics. Italy Vital Registration - Deaths 2001. Rome, Italy: Italian National Institute of Statistics.
Italian National Institute of Statistics. Italy Vital Registration - Deaths 2002. Rome, Italy: Italian National Institute of Statistics.
Italian National Institute of Statistics. Italy Vital Registration - Deaths 2003. Rome, Italy: Italian National Institute of Statistics.
Italian National Institute of Statistics. Italy Vital Registration - Deaths 2004. Rome, Italy: Italian National Institute of Statistics.
Italian National Institute of Statistics. Italy Vital Registration - Deaths 2005. Rome, Italy: Italian National Institute of Statistics.
Italian National Institute of Statistics. Italy Vital Registration - Deaths 2006. Rome, Italy: Italian National Institute of Statistics.
Italian National Institute of Statistics. Italy Vital Registration - Deaths 2007. Rome, Italy: Italian National Institute of Statistics.
Italian National Institute of Statistics. Italy Vital Registration - Deaths 2008. Rome, Italy: Italian National Institute of Statistics.
Italian National Institute of Statistics. Italy Vital Registration - Deaths 2009. Rome, Italy: Italian National Institute of Statistics.
Italian National Institute of Statistics. Italy Vital Registration - Deaths 2010. Rome, Italy: Italian National Institute of Statistics.
Italian National Institute of Statistics. Italy Vital Registration - Deaths 2011. Rome, Italy: Italian National Institute of Statistics.
Italian National Institute of Statistics. Italy Vital Registration - Deaths 2012. Rome, Italy: Italian National Institute of Statistics.
Italian National Institute of Statistics. Italy Vital Registration - Deaths 2014. Rome, Italy: Italian National Institute of Statistics.
Italian National Institute of Statistics. Italy Vital Registration - Deaths 2015. Rome, Italy: Italian National Institute of Statistics.

Italian National Institute of Statistics. Italy Vital Registration - Deaths 2016. Rome, Italy: Italian National Institute of Statistics.
Italian National Institute of Statistics. Italy Vital Registration - Deaths 2017. Rome, Italy: Italian National Institute of Statistics.
Italian National Institute of Statistics. Italy Vital Registration - Deaths 2018. Rome, Italy: Italian National Institute of Statistics.
Italy Vital Registration - Deaths 2013. Rome, Italy: Italian National Institute of Statistics.
Jamaica Vital Registration - Deaths 1980 ICD9.
Jamaica Vital Registration - Deaths 1981 ICD9.
Jamaica Vital Registration - Deaths 1982 ICD9.
Jamaica Vital Registration - Deaths 1983 ICD9.
Jamaica Vital Registration - Deaths 1984 ICD9.
Jamaica Vital Registration - Deaths 1985 ICD9.
Jamaica Vital Registration - Deaths 1986 ICD9.
Jamaica Vital Registration - Deaths 1987 ICD9.
Jamaica Vital Registration - Deaths 1988 ICD9.
Jamaica Vital Registration - Deaths 1989 ICD9.
Jamaica Vital Registration - Deaths 1990 ICD9.
Jamaica Vital Registration - Deaths 1991 ICD9.
Jamaica Vital Registration - Deaths 2000 ICD10.
Jamaica Vital Registration - Deaths 2001 ICD10.
Jamaica Vital Registration - Deaths 2002 ICD10.
Jamaica Vital Registration - Deaths 2003 ICD10.
Jamaica Vital Registration - Deaths 2004 ICD10.
Jamaica Vital Registration - Deaths 2005 ICD10.
Jamaica Vital Registration - Deaths 2006 ICD10.
Jamaica Vital Registration - Deaths 2009 ICD10.
Jamaica Vital Registration - Deaths 2010 ICD10.
Jamaica Vital Registration - Deaths 2011 ICD10.
Jamaica Vital Registration - Deaths 2012 ICD10.
Jamaica Vital Registration - Deaths 2013 ICD10.
Jamaica Vital Registration - Deaths 2014 ICD10.
Jordan Vital Registration - Deaths 2008 ICD10.
Jordan Vital Registration - Deaths 2009 ICD10.
Jordan Vital Registration - Deaths 2010 ICD10.
Jordan Vital Registration - Deaths 2011 ICD10.
Jordan Vital Registration - Deaths 2012 ICD10.
Jordan Vital Registration - Deaths 2015 ICD10.
Kazakhstan Vital Registration - Deaths 1981 ICD9.
Kazakhstan Vital Registration - Deaths 1982 ICD9.
Kazakhstan Vital Registration - Deaths 1985 ICD9.
Kazakhstan Vital Registration - Deaths 1986 ICD9.
Kazakhstan Vital Registration - Deaths 1987 ICD9.
Kazakhstan Vital Registration - Deaths 1988 ICD9.
Kazakhstan Vital Registration - Deaths 1989 ICD9.
Kazakhstan Vital Registration - Deaths 1990 ICD9.
Kazakhstan Vital Registration - Deaths 1991 ICD9.
Kazakhstan Vital Registration - Deaths 1992 ICD9.
Kazakhstan Vital Registration - Deaths 1993 ICD9.
Kazakhstan Vital Registration - Deaths 1994 ICD9.
Kazakhstan Vital Registration - Deaths 1995 ICD9.
Kazakhstan Vital Registration - Deaths 1996 ICD9.
Kazakhstan Vital Registration - Deaths 1997 ICD9.
Kazakhstan Vital Registration - Deaths 1998 ICD9.
Kazakhstan Vital Registration - Deaths 1999 ICD9.
Kazakhstan Vital Registration - Deaths 2000 ICD9.
Kazakhstan Vital Registration - Deaths 2001 ICD9.
Kazakhstan Vital Registration - Deaths 2002 ICD9.
Kazakhstan Vital Registration - Deaths 2003 ICD9.
Kazakhstan Vital Registration - Deaths 2004 ICD10.
Kazakhstan Vital Registration - Deaths 2005 ICD10.
Kazakhstan Vital Registration - Deaths 2006 ICD10.
Kazakhstan Vital Registration - Deaths 2007 ICD10.
Kazakhstan Vital Registration - Deaths 2008 ICD10.

Kazakhstan Vital Registration - Deaths 2009 ICD10.
Kazakhstan Vital Registration - Deaths 2010 ICD10.
Kazakhstan Vital Registration - Deaths 2011 ICD10.
Kazakhstan Vital Registration - Deaths 2012 ICD10.
Kazakhstan Vital Registration - Deaths 2013 ICD10.
Kazakhstan Vital Registration - Deaths 2014 ICD10.
Kazakhstan Vital Registration - Deaths 2015 ICD10.
Kazakhstan Vital Registration - Deaths 2017 ICD10.
Kiribati Vital Registration - Deaths 1992 ICD9.
Kiribati Vital Registration - Deaths 1993 ICD9.
Kiribati Vital Registration - Deaths 1995 ICD9.
Kiribati Vital Registration - Deaths 1997 ICD9.
Kiribati Vital Registration - Deaths 1998 ICD9.
Kiribati Vital Registration - Deaths 1999 ICD10.
Kiribati Vital Registration - Deaths 2000 ICD10.
Kuwait Vital Registration - Deaths 1980 ICD9.
Kuwait Vital Registration - Deaths 1981 ICD9.
Kuwait Vital Registration - Deaths 1982 ICD9.
Kuwait Vital Registration - Deaths 1983 ICD9.
Kuwait Vital Registration - Deaths 1984 ICD9.
Kuwait Vital Registration - Deaths 1985 ICD9.
Kuwait Vital Registration - Deaths 1986 ICD9.
Kuwait Vital Registration - Deaths 1987 ICD9.
Kuwait Vital Registration - Deaths 1993 ICD9.
Kuwait Vital Registration - Deaths 1994 ICD9.
Kuwait Vital Registration - Deaths 1995 ICD10.
Kuwait Vital Registration - Deaths 1996 ICD10.
Kuwait Vital Registration - Deaths 1997 ICD10.
Kuwait Vital Registration - Deaths 1998 ICD10.
Kuwait Vital Registration - Deaths 1999 ICD10.
Kuwait Vital Registration - Deaths 2000 ICD10.
Kuwait Vital Registration - Deaths 2001 ICD10.
Kuwait Vital Registration - Deaths 2002 ICD10.
Kuwait Vital Registration - Deaths 2003 ICD10.
Kuwait Vital Registration - Deaths 2004 ICD10.
Kuwait Vital Registration - Deaths 2005 ICD10.
Kuwait Vital Registration - Deaths 2006 ICD10.
Kuwait Vital Registration - Deaths 2007 ICD10.
Kuwait Vital Registration - Deaths 2008 ICD10.
Kuwait Vital Registration - Deaths 2009 ICD10.
Kuwait Vital Registration - Deaths 2010 ICD10.
Kuwait Vital Registration - Deaths 2011 ICD10.
Kuwait Vital Registration - Deaths 2012 ICD10.
Kuwait Vital Registration - Deaths 2013 ICD10.
Kuwait Vital Registration - Deaths 2014 ICD10.
Kuwait Vital Registration - Deaths 2015 ICD10.
Kuwait Vital Registration - Deaths 2016 ICD10.
Kuwait Vital Registration - Deaths 2017 ICD10.
Kyrgyzstan Vital Registration - Deaths 1981 ICD9.
Kyrgyzstan Vital Registration - Deaths 1982 ICD9.
Kyrgyzstan Vital Registration - Deaths 1985 ICD9.
Kyrgyzstan Vital Registration - Deaths 1986 ICD9.
Kyrgyzstan Vital Registration - Deaths 1987 ICD9.
Kyrgyzstan Vital Registration - Deaths 1988 ICD9.
Kyrgyzstan Vital Registration - Deaths 1989 ICD9.
Kyrgyzstan Vital Registration - Deaths 1990 ICD9.
Kyrgyzstan Vital Registration - Deaths 1991 ICD9.
Kyrgyzstan Vital Registration - Deaths 1992 ICD9.
Kyrgyzstan Vital Registration - Deaths 1993 ICD9.
Kyrgyzstan Vital Registration - Deaths 1994 ICD9.
Kyrgyzstan Vital Registration - Deaths 1995 ICD9.

Kyrgyzstan Vital Registration - Deaths 1996 ICD9.
Kyrgyzstan Vital Registration - Deaths 1997 ICD9.
Kyrgyzstan Vital Registration - Deaths 1998 ICD9.
Kyrgyzstan Vital Registration - Deaths 1999 ICD9.
Kyrgyzstan Vital Registration - Deaths 2000 ICD10.
Kyrgyzstan Vital Registration - Deaths 2001 ICD10.
Kyrgyzstan Vital Registration - Deaths 2002 ICD10.
Kyrgyzstan Vital Registration - Deaths 2003 ICD10.
Kyrgyzstan Vital Registration - Deaths 2004 ICD10.
Kyrgyzstan Vital Registration - Deaths 2005 ICD10.
Kyrgyzstan Vital Registration - Deaths 2006 ICD10.
Kyrgyzstan Vital Registration - Deaths 2007 ICD10.
Kyrgyzstan Vital Registration - Deaths 2008 ICD10.
Kyrgyzstan Vital Registration - Deaths 2009 ICD10.
Kyrgyzstan Vital Registration - Deaths 2010 ICD10.
Kyrgyzstan Vital Registration - Deaths 2011 ICD10.
Kyrgyzstan Vital Registration - Deaths 2012 ICD10.
Kyrgyzstan Vital Registration - Deaths 2013 ICD10.
Kyrgyzstan Vital Registration - Deaths 2014 ICD10.
Kyrgyzstan Vital Registration - Deaths 2015 ICD10.
Kyrgyzstan Vital Registration - Deaths 2016 ICD10.
Latvia Vital Registration - Deaths 1980 ICD9.
Latvia Vital Registration - Deaths 1981 ICD9.
Latvia Vital Registration - Deaths 1982 ICD9.
Latvia Vital Registration - Deaths 1983 ICD9.
Latvia Vital Registration - Deaths 1984 ICD9.
Latvia Vital Registration - Deaths 1985 ICD9.
Latvia Vital Registration - Deaths 1986 ICD9.
Latvia Vital Registration - Deaths 1987 ICD9.
Latvia Vital Registration - Deaths 1988 ICD9.
Latvia Vital Registration - Deaths 1989 ICD9.
Latvia Vital Registration - Deaths 1990 ICD9.
Latvia Vital Registration - Deaths 1991 ICD9.
Latvia Vital Registration - Deaths 1992 ICD9.
Latvia Vital Registration - Deaths 1993 ICD9.
Latvia Vital Registration - Deaths 1994 ICD9.
Latvia Vital Registration - Deaths 1995 ICD9.
Latvia Vital Registration - Deaths 1996 ICD10.
Latvia Vital Registration - Deaths 1997 ICD10.
Latvia Vital Registration - Deaths 1998 ICD10.
Latvia Vital Registration - Deaths 1999 ICD10.
Latvia Vital Registration - Deaths 2000 ICD10.
Latvia Vital Registration - Deaths 2001 ICD10.
Latvia Vital Registration - Deaths 2002 ICD10.
Latvia Vital Registration - Deaths 2003 ICD10.
Latvia Vital Registration - Deaths 2004 ICD10.
Latvia Vital Registration - Deaths 2005 ICD10.
Latvia Vital Registration - Deaths 2006 ICD10.
Latvia Vital Registration - Deaths 2007 ICD10.
Latvia Vital Registration - Deaths 2008 ICD10.
Latvia Vital Registration - Deaths 2009 ICD10.
Latvia Vital Registration - Deaths 2010 ICD10.
Latvia Vital Registration - Deaths 2011 ICD10.
Latvia Vital Registration - Deaths 2012 ICD10.
Latvia Vital Registration - Deaths 2013 ICD10.
Latvia Vital Registration - Deaths 2014 ICD10.
Latvia Vital Registration - Deaths 2015 ICD10.
Latvia Vital Registration - Deaths 2016 ICD10.
Latvia Vital Registration - Deaths 2017 ICD10.
Latvia Vital Registration - Deaths 2018 ICD10.
Lebanon Vital Registration - Deaths 2017 ICD10.

Lebanon Vital Registration - Deaths 2018 ICD10.
Lithuania Vital Registration - Deaths 1981 ICD9.
Lithuania Vital Registration - Deaths 1982 ICD9.
Lithuania Vital Registration - Deaths 1985 ICD9.
Lithuania Vital Registration - Deaths 1986 ICD9.
Lithuania Vital Registration - Deaths 1987 ICD9.
Lithuania Vital Registration - Deaths 1988 ICD9.
Lithuania Vital Registration - Deaths 1989 ICD9.
Lithuania Vital Registration - Deaths 1990 ICD9.
Lithuania Vital Registration - Deaths 1991 ICD9.
Lithuania Vital Registration - Deaths 1992 ICD9.
Lithuania Vital Registration - Deaths 1993 ICD9.
Lithuania Vital Registration - Deaths 1994 ICD9.
Lithuania Vital Registration - Deaths 1995 ICD9.
Lithuania Vital Registration - Deaths 1996 ICD9.
Lithuania Vital Registration - Deaths 1997 ICD9.
Lithuania Vital Registration - Deaths 1998 ICD10.
Lithuania Vital Registration - Deaths 1999 ICD10.
Lithuania Vital Registration - Deaths 2000 ICD10.
Lithuania Vital Registration - Deaths 2001 ICD10.
Lithuania Vital Registration - Deaths 2002 ICD10.
Lithuania Vital Registration - Deaths 2003 ICD10.
Lithuania Vital Registration - Deaths 2004 ICD10.
Lithuania Vital Registration - Deaths 2005 ICD10.
Lithuania Vital Registration - Deaths 2006 ICD10.
Lithuania Vital Registration - Deaths 2007 ICD10.
Lithuania Vital Registration - Deaths 2008 ICD10.
Lithuania Vital Registration - Deaths 2009 ICD10.
Lithuania Vital Registration - Deaths 2010 ICD10.
Lithuania Vital Registration - Deaths 2011 ICD10.
Lithuania Vital Registration - Deaths 2012 ICD10.
Lithuania Vital Registration - Deaths 2013 ICD10.
Lithuania Vital Registration - Deaths 2014 ICD10.
Lithuania Vital Registration - Deaths 2015 ICD10.
Lithuania Vital Registration - Deaths 2016 ICD10.
Lithuania Vital Registration - Deaths 2017 ICD10.
Lithuania Vital Registration - Deaths 2018 ICD10.
Luxembourg Vital Registration - Deaths 1980 ICD9.
Luxembourg Vital Registration - Deaths 1981 ICD9.
Luxembourg Vital Registration - Deaths 1982 ICD9.
Luxembourg Vital Registration - Deaths 1983 ICD9.
Luxembourg Vital Registration - Deaths 1984 ICD9.
Luxembourg Vital Registration - Deaths 1985 ICD9.
Luxembourg Vital Registration - Deaths 1986 ICD9.
Luxembourg Vital Registration - Deaths 1987 ICD9.
Luxembourg Vital Registration - Deaths 1988 ICD9.
Luxembourg Vital Registration - Deaths 1989 ICD9.
Luxembourg Vital Registration - Deaths 1990 ICD9.
Luxembourg Vital Registration - Deaths 1991 ICD9.
Luxembourg Vital Registration - Deaths 1992 ICD9.
Luxembourg Vital Registration - Deaths 1993 ICD9.
Luxembourg Vital Registration - Deaths 1994 ICD9.
Luxembourg Vital Registration - Deaths 1995 ICD9.
Luxembourg Vital Registration - Deaths 1996 ICD9.
Luxembourg Vital Registration - Deaths 1997 ICD9.
Luxembourg Vital Registration - Deaths 1998 ICD10.
Luxembourg Vital Registration - Deaths 1999 ICD10.
Luxembourg Vital Registration - Deaths 2000 ICD10.
Luxembourg Vital Registration - Deaths 2001 ICD10.
Luxembourg Vital Registration - Deaths 2002 ICD10.
Luxembourg Vital Registration - Deaths 2003 ICD10.

Luxembourg Vital Registration - Deaths 2004 ICD10.
Luxembourg Vital Registration - Deaths 2005 ICD10.
Luxembourg Vital Registration - Deaths 2006 ICD10.
Luxembourg Vital Registration - Deaths 2007 ICD10.
Luxembourg Vital Registration - Deaths 2008 ICD10.
Luxembourg Vital Registration - Deaths 2009 ICD10.
Luxembourg Vital Registration - Deaths 2010 ICD10.
Luxembourg Vital Registration - Deaths 2011 ICD10.
Luxembourg Vital Registration - Deaths 2012 ICD10.
Luxembourg Vital Registration - Deaths 2013 ICD10.
Luxembourg Vital Registration - Deaths 2014 ICD10.
Luxembourg Vital Registration - Deaths 2015 ICD10.
Luxembourg Vital Registration - Deaths 2016 ICD10.
Luxembourg Vital Registration - Deaths 2017 ICD10.
Macau Vital Registration - Deaths 1994 ICD9.
Macedonia Vital Registration - Deaths 1991 ICD9.
Macedonia Vital Registration - Deaths 1992 ICD9.
Macedonia Vital Registration - Deaths 1993 ICD9.
Macedonia Vital Registration - Deaths 1994 ICD9.
Macedonia Vital Registration - Deaths 1995 ICD9.
Macedonia Vital Registration - Deaths 1996 ICD9.
Macedonia Vital Registration - Deaths 1997 ICD9.
Macedonia Vital Registration - Deaths 1998 ICD9.
Macedonia Vital Registration - Deaths 1999 ICD9.
Macedonia Vital Registration - Deaths 2000 ICD9.
Macedonia Vital Registration - Deaths 2001 ICD9.
Macedonia Vital Registration - Deaths 2002 ICD9.
Macedonia Vital Registration - Deaths 2003 ICD9.
Macedonia Vital Registration - Deaths 2004 ICD9.
Macedonia Vital Registration - Deaths 2005 ICD9.
Macedonia Vital Registration - Deaths 2006 ICD10.
Macedonia Vital Registration - Deaths 2007 ICD10.
Macedonia Vital Registration - Deaths 2008 ICD10.
Macedonia Vital Registration - Deaths 2009 ICD10.
Macedonia Vital Registration - Deaths 2010 ICD10.
Macedonia Vital Registration - Deaths 2011 ICD10.
Macedonia Vital Registration - Deaths 2012 ICD10.
Macedonia Vital Registration - Deaths 2013 ICD10.
Malaysia Vital Registration - Deaths 2000 ICD10.
Malaysia Vital Registration - Deaths 2001 ICD10.
Malaysia Vital Registration - Deaths 2002 ICD10.
Malaysia Vital Registration - Deaths 2003 ICD10.
Malaysia Vital Registration - Deaths 2004 ICD10.
Malaysia Vital Registration - Deaths 2005 ICD10.
Malaysia Vital Registration - Deaths 2006 ICD10.
Malaysia Vital Registration - Deaths 2007 ICD10.
Malaysia Vital Registration - Deaths 2008 ICD10.
Malaysia Vital Registration - Deaths 2009 ICD10.
Malaysia Vital Registration - Deaths 2010 ICD10.
Malaysia Vital Registration - Deaths 2011 ICD10.
Malaysia Vital Registration - Deaths 2012 ICD10.
Malaysia Vital Registration - Deaths 2013 ICD10.
Malaysia Vital Registration - Deaths 2014 ICD10.
Maldives Vital Registration - Deaths 2010 ICD10.
Maldives Vital Registration - Deaths 2011 ICD10.
Maldives Vital Registration - Deaths 2012 ICD10.
Maldives Vital Registration - Deaths 2013 ICD10.
Maldives Vital Registration - Deaths 2014 ICD10.
Maldives Vital Registration - Deaths 2015 ICD10.
Maldives Vital Registration - Deaths 2016 ICD10.
Maldives Vital Registration - Deaths 2017 ICD10.

Malta Vital Registration - Deaths 1980 ICD9.
Malta Vital Registration - Deaths 1981 ICD9.
Malta Vital Registration - Deaths 1982 ICD9.
Malta Vital Registration - Deaths 1983 ICD9.
Malta Vital Registration - Deaths 1984 ICD9.
Malta Vital Registration - Deaths 1985 ICD9.
Malta Vital Registration - Deaths 1986 ICD9.
Malta Vital Registration - Deaths 1987 ICD9.
Malta Vital Registration - Deaths 1988 ICD9.
Malta Vital Registration - Deaths 1989 ICD9.
Malta Vital Registration - Deaths 1990 ICD9.
Malta Vital Registration - Deaths 1991 ICD9.
Malta Vital Registration - Deaths 1992 ICD9.
Malta Vital Registration - Deaths 1993 ICD9.
Malta Vital Registration - Deaths 1994 ICD9.
Malta Vital Registration - Deaths 1995 ICD10.
Malta Vital Registration - Deaths 1996 ICD10.
Malta Vital Registration - Deaths 1997 ICD10.
Malta Vital Registration - Deaths 1998 ICD10.
Malta Vital Registration - Deaths 1999 ICD10.
Malta Vital Registration - Deaths 2000 ICD10.
Malta Vital Registration - Deaths 2001 ICD10.
Malta Vital Registration - Deaths 2002 ICD10.
Malta Vital Registration - Deaths 2003 ICD10.
Malta Vital Registration - Deaths 2004 ICD10.
Malta Vital Registration - Deaths 2005 ICD10.
Malta Vital Registration - Deaths 2006 ICD10.
Malta Vital Registration - Deaths 2007 ICD10.
Malta Vital Registration - Deaths 2008 ICD10.
Malta Vital Registration - Deaths 2009 ICD10.
Malta Vital Registration - Deaths 2010 ICD10.
Malta Vital Registration - Deaths 2011 ICD10.
Malta Vital Registration - Deaths 2012 ICD10.
Malta Vital Registration - Deaths 2013 ICD10.
Malta Vital Registration - Deaths 2014 ICD10.
Malta Vital Registration - Deaths 2015 ICD10.
Malta Vital Registration - Deaths 2016 ICD10.
Malta Vital Registration - Deaths 2017 ICD10.
Mauritius Vital Registration - Deaths 1981 ICD9.
Mauritius Vital Registration - Deaths 1982 ICD9.
Mauritius Vital Registration - Deaths 1983 ICD9.
Mauritius Vital Registration - Deaths 1984 ICD9.
Mauritius Vital Registration - Deaths 1985 ICD9.
Mauritius Vital Registration - Deaths 1986 ICD9.
Mauritius Vital Registration - Deaths 1987 ICD9.
Mauritius Vital Registration - Deaths 1988 ICD9.
Mauritius Vital Registration - Deaths 1989 ICD9.
Mauritius Vital Registration - Deaths 1990 ICD9.
Mauritius Vital Registration - Deaths 1991 ICD9.
Mauritius Vital Registration - Deaths 1992 ICD9.
Mauritius Vital Registration - Deaths 1993 ICD9.
Mauritius Vital Registration - Deaths 1994 ICD9.
Mauritius Vital Registration - Deaths 1995 ICD9.
Mauritius Vital Registration - Deaths 1996 ICD9.
Mauritius Vital Registration - Deaths 1997 ICD9.
Mauritius Vital Registration - Deaths 1998 ICD9.
Mauritius Vital Registration - Deaths 1999 ICD9.
Mauritius Vital Registration - Deaths 2000 ICD9.
Mauritius Vital Registration - Deaths 2001 ICD9.
Mauritius Vital Registration - Deaths 2002 ICD9.
Mauritius Vital Registration - Deaths 2003 ICD9.

Mauritius Vital Registration - Deaths 2004 ICD9.
Mauritius Vital Registration - Deaths 2005 ICD10.
Mauritius Vital Registration - Deaths 2006 ICD10.
Mauritius Vital Registration - Deaths 2007 ICD10.
Mauritius Vital Registration - Deaths 2008 ICD10.
Mauritius Vital Registration - Deaths 2009 ICD10.
Mauritius Vital Registration - Deaths 2010 ICD10.
Mauritius Vital Registration - Deaths 2011 ICD10.
Mauritius Vital Registration - Deaths 2012 ICD10.
Mauritius Vital Registration - Deaths 2013 ICD10.
Mauritius Vital Registration - Deaths 2014 ICD10.
Mauritius Vital Registration - Deaths 2015 ICD10.
Mauritius Vital Registration - Deaths 2016 ICD10.
Mauritius Vital Registration - Deaths 2017 ICD10.
Mauritius Vital Registration - Deaths 2018 ICD10.
Ministry of Health (Brazil). Brazil Mortality Information System - Deaths 1980. Rio de Janeiro, Brazil: Ministry of Health (Brazil).
Ministry of Health (Brazil). Brazil Mortality Information System - Deaths 1981. Rio de Janeiro, Brazil: Ministry of Health (Brazil).
Ministry of Health (Brazil). Brazil Mortality Information System - Deaths 1982. Rio de Janeiro, Brazil: Ministry of Health (Brazil).
Ministry of Health (Brazil). Brazil Mortality Information System - Deaths 1983. Rio de Janeiro, Brazil: Ministry of Health (Brazil).
Ministry of Health (Brazil). Brazil Mortality Information System - Deaths 1984. Rio de Janeiro, Brazil: Ministry of Health (Brazil).
Ministry of Health (Brazil). Brazil Mortality Information System - Deaths 1985. Rio de Janeiro, Brazil: Ministry of Health (Brazil).
Ministry of Health (Brazil). Brazil Mortality Information System - Deaths 1986. Rio de Janeiro, Brazil: Ministry of Health (Brazil).
Ministry of Health (Brazil). Brazil Mortality Information System - Deaths 1987. Rio de Janeiro, Brazil: Ministry of Health (Brazil).
Ministry of Health (Brazil). Brazil Mortality Information System - Deaths 1988. Rio de Janeiro, Brazil: Ministry of Health (Brazil).
Ministry of Health (Brazil). Brazil Mortality Information System - Deaths 1989. Rio de Janeiro, Brazil: Ministry of Health (Brazil).
Ministry of Health (Brazil). Brazil Mortality Information System - Deaths 1990. Rio de Janeiro, Brazil: Ministry of Health (Brazil).
Ministry of Health (Brazil). Brazil Mortality Information System - Deaths 1991. Rio de Janeiro, Brazil: Ministry of Health (Brazil).
Ministry of Health (Brazil). Brazil Mortality Information System - Deaths 1992. Rio de Janeiro, Brazil: Ministry of Health (Brazil).
Ministry of Health (Brazil). Brazil Mortality Information System - Deaths 1993. Rio de Janeiro, Brazil: Ministry of Health (Brazil).
Ministry of Health (Brazil). Brazil Mortality Information System - Deaths 1994. Rio de Janeiro, Brazil: Ministry of Health (Brazil).
Ministry of Health (Brazil). Brazil Mortality Information System - Deaths 1995. Rio de Janeiro, Brazil: Ministry of Health (Brazil).
Ministry of Health (Brazil). Brazil Mortality Information System - Deaths 1996. Rio de Janeiro, Brazil: Ministry of Health (Brazil).
Ministry of Health (Brazil). Brazil Mortality Information System - Deaths 1997. Rio de Janeiro, Brazil: Ministry of Health (Brazil).
Ministry of Health (Brazil). Brazil Mortality Information System - Deaths 1998. Rio de Janeiro, Brazil: Ministry of Health (Brazil).
Ministry of Health (Brazil). Brazil Mortality Information System - Deaths 1999. Rio de Janeiro, Brazil: Ministry of Health (Brazil).
Ministry of Health (Brazil). Brazil Mortality Information System - Deaths 2000. Rio de Janeiro, Brazil: Ministry of Health (Brazil).
Ministry of Health (Brazil). Brazil Mortality Information System - Deaths 2001. Rio de Janeiro, Brazil: Ministry of Health (Brazil).
Ministry of Health (Brazil). Brazil Mortality Information System - Deaths 2002. Rio de Janeiro, Brazil: Ministry of Health (Brazil).
Ministry of Health (Brazil). Brazil Mortality Information System - Deaths 2003. Rio de Janeiro, Brazil: Ministry of Health (Brazil).
Ministry of Health (Brazil). Brazil Mortality Information System - Deaths 2004. Rio de Janeiro, Brazil: Ministry of Health (Brazil).
Ministry of Health (Brazil). Brazil Mortality Information System - Deaths 2005. Rio de Janeiro, Brazil: Ministry of Health (Brazil).
Ministry of Health (Brazil). Brazil Mortality Information System - Deaths 2006. Rio de Janeiro, Brazil: Ministry of Health (Brazil).
Ministry of Health (Brazil). Brazil Mortality Information System - Deaths 2007. Rio de Janeiro, Brazil: Ministry of Health (Brazil).
Ministry of Health (Brazil). Brazil Mortality Information System - Deaths 2008. Rio de Janeiro, Brazil: Ministry of Health (Brazil).
Ministry of Health (Brazil). Brazil Mortality Information System - Deaths 2009. Rio de Janeiro, Brazil: Ministry of Health (Brazil).
Ministry of Health (Brazil). Brazil Mortality Information System - Deaths 2010. Rio de Janeiro, Brazil: Ministry of Health (Brazil).
Ministry of Health (Brazil). Brazil Mortality Information System - Deaths 2011. Rio de Janeiro, Brazil: Ministry of Health (Brazil).
Ministry of Health (Brazil). Brazil Mortality Information System - Deaths 2012. Rio de Janeiro, Brazil: Ministry of Health (Brazil).
Ministry of Health (Brazil). Brazil Mortality Information System - Deaths 2013. Rio de Janeiro, Brazil: Ministry of Health (Brazil).
Ministry of Health (Brazil). Brazil Mortality Information System - Deaths 2014. Rio de Janeiro, Brazil: Ministry of Health (Brazil).
Ministry of Health (Brazil). Brazil Mortality Information System - Deaths 2015. Rio de Janeiro, Brazil: Ministry of Health (Brazil).
Ministry of Health (Brazil). Brazil Mortality Information System - Deaths 2016. Rio de Janeiro, Brazil: Ministry of Health (Brazil).
Ministry of Health (Brazil). Brazil Mortality Information System - Deaths 2017. Rio de Janeiro, Brazil: Ministry of Health (Brazil).
Ministry of Health (Brazil). Brazil Mortality Information System - Deaths 2018. Rio de Janeiro, Brazil: Ministry of Health (Brazil).
Ministry of Health (Brazil). Brazil Mortality Information System - Deaths 2019. Rio de Janeiro, Brazil: Ministry of Health (Brazil).
Ministry of Health (Indonesia). Indonesia Cause of Death Survey 2010-2011.
Ministry of Health (Indonesia). Indonesia Sample Registration System - Deaths 2013.
Ministry of Health (Indonesia). Indonesia Sample Registration System - Deaths 2014.
Ministry of Health (Jordan). Jordan Causes of Death and Population 2013.
Ministry of Health (Jordan). Jordan Vital Registration - Deaths 2004-2006.
Ministry of Health (Kyrgyzstan). Kyrgyzstan Vital Registration - Deaths 2017.

Ministry of Health (Mongolia). Mongolia Vital Registration - Deaths 2018.
Ministry of Health (Mongolia). Mongolia Vital Registration - Deaths 2019.
Ministry of Health (Mongolia). Mongolia Vital Registration - Deaths 2020.
Ministry of Health (New Zealand). New Zealand Mortality Collection 1988. Wellington, New Zealand: Ministry of Health (New Zealand).
Ministry of Health (New Zealand). New Zealand Mortality Collection 1989. Wellington, New Zealand: Ministry of Health (New Zealand).
Ministry of Health (New Zealand). New Zealand Mortality Collection 1990. Wellington, New Zealand: Ministry of Health (New Zealand).
Ministry of Health (New Zealand). New Zealand Mortality Collection 1991. Wellington, New Zealand: Ministry of Health (New Zealand).
Ministry of Health (New Zealand). New Zealand Mortality Collection 1992. Wellington, New Zealand: Ministry of Health (New Zealand).
Ministry of Health (New Zealand). New Zealand Mortality Collection 1993. Wellington, New Zealand: Ministry of Health (New Zealand).
Ministry of Health (New Zealand). New Zealand Mortality Collection 1994. Wellington, New Zealand: Ministry of Health (New Zealand).
Ministry of Health (New Zealand). New Zealand Mortality Collection 1996. Wellington, New Zealand: Ministry of Health (New Zealand).
Ministry of Health (New Zealand). New Zealand Mortality Collection 1997. Wellington, New Zealand: Ministry of Health (New Zealand).
Ministry of Health (New Zealand). New Zealand Mortality Collection 1998. Wellington, New Zealand: Ministry of Health (New Zealand).
Ministry of Health (New Zealand). New Zealand Mortality Collection 1999. Wellington, New Zealand: Ministry of Health (New Zealand).
Ministry of Health (New Zealand). New Zealand Mortality Collection 2000. Wellington, New Zealand: Ministry of Health (New Zealand).
Ministry of Health (New Zealand). New Zealand Mortality Collection 2001. Wellington, New Zealand: Ministry of Health (New Zealand).
Ministry of Health (New Zealand). New Zealand Mortality Collection 2002. Wellington, New Zealand: Ministry of Health (New Zealand).
Ministry of Health (New Zealand). New Zealand Mortality Collection 2003. Wellington, New Zealand: Ministry of Health (New Zealand).
Ministry of Health (New Zealand). New Zealand Mortality Collection 2004. Wellington, New Zealand: Ministry of Health (New Zealand).
Ministry of Health (New Zealand). New Zealand Mortality Collection 2005. Wellington, New Zealand: Ministry of Health (New Zealand).
Ministry of Health (New Zealand). New Zealand Mortality Collection 2006. Wellington, New Zealand: Ministry of Health (New Zealand).
Ministry of Health (New Zealand). New Zealand Mortality Collection 2007. Wellington, New Zealand: Ministry of Health (New Zealand).
Ministry of Health (New Zealand). New Zealand Mortality Collection 2008. Wellington, New Zealand: Ministry of Health (New Zealand).
Ministry of Health (New Zealand). New Zealand Mortality Collection 2009. Wellington, New Zealand: Ministry of Health (New Zealand).
Ministry of Health (New Zealand). New Zealand Mortality Collection 2010. Wellington, New Zealand: Ministry of Health (New Zealand).
Ministry of Health (New Zealand). New Zealand Mortality Collection 2011. Wellington, New Zealand: Ministry of Health (New Zealand).
Ministry of Health (New Zealand). New Zealand Mortality Collection 2012. Wellington, New Zealand: Ministry of Health (New Zealand).
Ministry of Health (New Zealand). New Zealand Mortality Collection 2013. Wellington, New Zealand: Ministry of Health (New Zealand).
Ministry of Health (New Zealand). New Zealand Mortality Collection 2014. Wellington, New Zealand: Ministry of Health (New Zealand).
Ministry of Health (New Zealand). New Zealand Mortality Collection 2015. Wellington, New Zealand: Ministry of Health (New Zealand).
Ministry of Health (New Zealand). New Zealand Mortality Collection 2016. Wellington, New Zealand: Ministry of Health (New Zealand).
Ministry of Health (New Zealand). New Zealand Mortality Collection 2017. Wellington, New Zealand: Ministry of Health (New Zealand).
Ministry of Health (New Zealand). New Zealand Mortality Data 1980. Wellington, New Zealand: Ministry of Health (New Zealand).
Ministry of Health (New Zealand). New Zealand Mortality Data 1981. Wellington, New Zealand: Ministry of Health (New Zealand).
Ministry of Health (New Zealand). New Zealand Mortality Data 1982. Wellington, New Zealand: Ministry of Health (New Zealand).
Ministry of Health (New Zealand). New Zealand Mortality Data 1983. Wellington, New Zealand: Ministry of Health (New Zealand).
Ministry of Health (New Zealand). New Zealand Mortality Data 1984. Wellington, New Zealand: Ministry of Health (New Zealand).
Ministry of Health (New Zealand). New Zealand Mortality Data 1985. Wellington, New Zealand: Ministry of Health (New Zealand).
Ministry of Health (New Zealand). New Zealand Mortality Data 1986. Wellington, New Zealand: Ministry of Health (New Zealand).
Ministry of Health (New Zealand). New Zealand Mortality Data 1987. Wellington, New Zealand: Ministry of Health (New Zealand).
Ministry of Health (Palestine). Palestine - Gaza Strip Mortality by Sex, Age, and Cause 2012.
Ministry of Health (Palestine). Palestine - Gaza Strip Mortality by Sex, Age, and Cause 2013.
Ministry of Health (Palestine). Palestine - Gaza Strip Mortality by Sex, Age, and Cause 2014.
Ministry of Health (Palestine). Palestine - Gaza Strip Mortality by Sex, Age, and Cause 2015.
Ministry of Health (Palestine). Palestine - Gaza Strip Mortality by Sex, Age, and Cause 2016.
Ministry of Health (Palestine). Palestine - Gaza Strip Mortality by Sex, Age, and Cause 2017.
Ministry of Health (Palestine). Palestine - Gaza Strip Mortality by Sex, Age, and Cause 2018.
Ministry of Health (Palestine). Palestine - West Bank Mortality by Sex, Age, and Cause 2012.
Ministry of Health (Palestine). Palestine - West Bank Mortality by Sex, Age, and Cause 2013.
Ministry of Health (Palestine). Palestine - West Bank Mortality by Sex, Age, and Cause 2014.
Ministry of Health (Palestine). Palestine - West Bank Mortality by Sex, Age, and Cause 2015.
Ministry of Health (Palestine). Palestine - West Bank Mortality by Sex, Age, and Cause 2016.
Ministry of Health (Palestine). Palestine - West Bank Mortality by Sex, Age, and Cause 2017.
Ministry of Health (Palestine). Palestine - West Bank Mortality by Sex, Age, and Cause 2018.
Ministry of Health (Saudi Arabia). Saudi Arabia Vital Registration - Deaths 1996-2011.
Ministry of Health (Saudi Arabia). Saudi Arabia Vital Registration - Deaths 2009-2013.
Ministry of Health (Singapore). Singapore Causes of Death 1980.
Ministry of Health (Singapore). Singapore Causes of Death 1981.
Ministry of Health (Singapore). Singapore Causes of Death 1982.
Ministry of Health (Singapore). Singapore Causes of Death 1983.
Ministry of Health (Singapore). Singapore Causes of Death 1984.

Ministry of Health (Singapore). Singapore Causes of Death 1985.
Ministry of Health (Singapore). Singapore Causes of Death 1986.
Ministry of Health (Singapore). Singapore Causes of Death 1987.
Ministry of Health (Singapore). Singapore Causes of Death 1988.
Ministry of Health (Singapore). Singapore Causes of Death 1989.
Ministry of Health (Singapore). Singapore Causes of Death 1990.
Ministry of Health (Singapore). Singapore Causes of Death 1991.
Ministry of Health (Singapore). Singapore Causes of Death 1992.
Ministry of Health (Singapore). Singapore Causes of Death 1993.
Ministry of Health (Singapore). Singapore Causes of Death 1994.
Ministry of Health (Singapore). Singapore Causes of Death 1995.
Ministry of Health (Singapore). Singapore Causes of Death 1996.
Ministry of Health (Singapore). Singapore Causes of Death 1997.
Ministry of Health (Singapore). Singapore Causes of Death 1998.
Ministry of Health (Singapore). Singapore Causes of Death 1999.
Ministry of Health (Singapore). Singapore Causes of Death 2000.
Ministry of Health (Singapore). Singapore Causes of Death 2001.
Ministry of Health (Singapore). Singapore Causes of Death 2002.
Ministry of Health (Singapore). Singapore Causes of Death 2003.
Ministry of Health (Singapore). Singapore Causes of Death 2004.
Ministry of Health (Singapore). Singapore Causes of Death 2005.
Ministry of Health (Singapore). Singapore Causes of Death 2006.
Ministry of Health (Singapore). Singapore Causes of Death 2007.
Ministry of Health (Singapore). Singapore Causes of Death 2008.
Ministry of Health (Singapore). Singapore Causes of Death 2009.
Ministry of Health (Singapore). Singapore Causes of Death 2010.
Ministry of Health (Singapore). Singapore Causes of Death 2011.
Ministry of Health (Singapore). Singapore Causes of Death 2012.
Ministry of Health (Singapore). Singapore Causes of Death 2013.
Ministry of Health (Singapore). Singapore Causes of Death 2014.
Ministry of Health (Singapore). Singapore Causes of Death 2015.
Ministry of Health (Singapore). Singapore Causes of Death 2016.
Ministry of Health (Singapore). Singapore Causes of Death 2017.
Ministry of Health and Medical Education (Iran). Iran Death Registration System 2001.
Ministry of Health and Medical Education (Iran). Iran Death Registration System 2002.
Ministry of Health and Medical Education (Iran). Iran Death Registration System 2003.
Ministry of Health and Medical Education (Iran). Iran Death Registration System 2004.
Ministry of Health and Medical Education (Iran). Iran Death Registration System 2005.
Ministry of Health and Medical Education (Iran). Iran Death Registration System 2006.
Ministry of Health and Medical Education (Iran). Iran Death Registration System 2007.
Ministry of Health and Medical Education (Iran). Iran Death Registration System 2008.
Ministry of Health and Medical Education (Iran). Iran Death Registration System 2009.
Ministry of Health and Medical Education (Iran). Iran Death Registration System 2010.
Ministry of Health and Medical Education (Iran). Iran Death Registration System 2011.
Ministry of Health and Medical Education (Iran). Iran Death Registration System 2012.
Ministry of Health and Medical Education (Iran). Iran Death Registration System 2013.
Ministry of Health and Medical Education (Iran). Iran Death Registration System 2014.
Ministry of Health and Medical Education (Iran). Iran Death Registration System 2015.
Ministry of Health and Medical Education (Iran). Iran Death Registration System 2016.
Ministry of Health and Welfare (Taiwan). Taiwan Statistics of Causes of Death 2017. Taipei City, Taiwan (Province of China): Ministry of Health and Welfare (Taiwan), 2018.
Ministry of Health and Welfare (Taiwan). Taiwan Vital Registration - Deaths 2013. Taipei City, Taiwan (Province of China): Ministry of Health and Welfare (Taiwan).
Ministry of Health and Welfare (Taiwan). Taiwan Vital Registration - Deaths 2014. Taipei City, Taiwan (Province of China): Ministry of Health and Welfare (Taiwan).
Ministry of Health and Welfare (Taiwan). Taiwan Vital Registration - Deaths 2015. Taipei City, Taiwan (Province of China): Ministry of Health and Welfare (Taiwan).
Ministry of Health and Welfare (Taiwan). Taiwan Vital Registration - Deaths 2016. Taipei City, Taiwan (Province of China): Ministry of Health and Welfare (Taiwan).
Ministry of Health, Labour and Welfare (Japan). Japan Vital Registration - Deaths 1980.
Ministry of Health, Labour and Welfare (Japan). Japan Vital Registration - Deaths 1981.
Ministry of Health, Labour and Welfare (Japan). Japan Vital Registration - Deaths 1982.

Ministry of Health, Labour and Welfare (Japan). Japan Vital Registration - Deaths 1983.
Ministry of Health, Labour and Welfare (Japan). Japan Vital Registration - Deaths 1984.
Ministry of Health, Labour and Welfare (Japan). Japan Vital Registration - Deaths 1985.
Ministry of Health, Labour and Welfare (Japan). Japan Vital Registration - Deaths 1986.
Ministry of Health, Labour and Welfare (Japan). Japan Vital Registration - Deaths 1987.
Ministry of Health, Labour and Welfare (Japan). Japan Vital Registration - Deaths 1988.
Ministry of Health, Labour and Welfare (Japan). Japan Vital Registration - Deaths 1989.
Ministry of Health, Labour and Welfare (Japan). Japan Vital Registration - Deaths 1990.
Ministry of Health, Labour and Welfare (Japan). Japan Vital Registration - Deaths 1991.
Ministry of Health, Labour and Welfare (Japan). Japan Vital Registration - Deaths 1992.
Ministry of Health, Labour and Welfare (Japan). Japan Vital Registration - Deaths 1993.
Ministry of Health, Labour and Welfare (Japan). Japan Vital Registration - Deaths 1994.
Ministry of Health, Labour and Welfare (Japan). Japan Vital Registration - Deaths 1995.
Ministry of Health, Labour and Welfare (Japan). Japan Vital Registration - Deaths 1996.
Ministry of Health, Labour and Welfare (Japan). Japan Vital Registration - Deaths 1997.
Ministry of Health, Labour and Welfare (Japan). Japan Vital Registration - Deaths 1998.
Ministry of Health, Labour and Welfare (Japan). Japan Vital Registration - Deaths 1999.
Ministry of Health, Labour and Welfare (Japan). Japan Vital Registration - Deaths 2000.
Ministry of Health, Labour and Welfare (Japan). Japan Vital Registration - Deaths 2001.
Ministry of Health, Labour and Welfare (Japan). Japan Vital Registration - Deaths 2002.
Ministry of Health, Labour and Welfare (Japan). Japan Vital Registration - Deaths 2003.
Ministry of Health, Labour and Welfare (Japan). Japan Vital Registration - Deaths 2004.
Ministry of Health, Labour and Welfare (Japan). Japan Vital Registration - Deaths 2005.
Ministry of Health, Labour and Welfare (Japan). Japan Vital Registration - Deaths 2006.
Ministry of Health, Labour and Welfare (Japan). Japan Vital Registration - Deaths 2007.
Ministry of Health, Labour and Welfare (Japan). Japan Vital Registration - Deaths 2008.
Ministry of Health, Labour and Welfare (Japan). Japan Vital Registration - Deaths 2009.
Ministry of Health, Labour and Welfare (Japan). Japan Vital Registration - Deaths 2010.
Ministry of Health, Labour and Welfare (Japan). Japan Vital Registration - Deaths 2011.
Ministry of Health, Labour and Welfare (Japan). Japan Vital Registration - Deaths 2012.
Ministry of Health, Labour and Welfare (Japan). Japan Vital Registration - Deaths 2013.
Ministry of Health, Labour and Welfare (Japan). Japan Vital Registration - Deaths 2014.
Ministry of Health, Labour and Welfare (Japan). Japan Vital Registration - Deaths 2015.
Ministry of Health, Labour and Welfare (Japan). Japan Vital Registration - Deaths 2016.
Ministry of Health, Labour and Welfare (Japan). Japan Vital Registration - Deaths 2017.
Ministry of Health, Labour and Welfare (Japan). Japan Vital Registration - Deaths 2018.
Ministry of Health, Labour and Welfare (Japan). Japan Vital Registration - Deaths 2019.
Ministry of Public Health (Thailand). Thailand Burden of Disease and Injuries 1998-1999.
Moldova Vital Registration - Deaths 2010 ICD10.
Moldova Vital Registration - Deaths 2011 ICD10.
Moldova Vital Registration - Deaths 2012 ICD10.
Moldova Vital Registration - Deaths 2013 ICD10.
Moldova Vital Registration - Deaths 2014 ICD10.
Moldova Vital Registration - Deaths 2015 ICD10.
Moldova Vital Registration - Deaths 2016 ICD10.
Moldova Vital Registration - Deaths 2017 ICD10.
Monaco Vital Registration - Deaths 1986 ICD9.
Monaco Vital Registration - Deaths 1987 ICD9.
Mongolia Vital Registration - Deaths 1994 ICD9.
Mongolia Vital Registration - Deaths 2016 ICD10.
Montenegro Vital Registration - Deaths 2001 ICD10.
Montenegro Vital Registration - Deaths 2002 ICD10.
Montenegro Vital Registration - Deaths 2005 ICD10.
Montenegro Vital Registration - Deaths 2006 ICD10.
Montenegro Vital Registration - Deaths 2007 ICD10.
Montenegro Vital Registration - Deaths 2008 ICD10.
Montenegro Vital Registration - Deaths 2009 ICD10.
Muhwava W. Contributions of the Africa Centre Demographic Surveillance to the Community. Umbiko. 2011; 12. 3-4.
Munthali CV, Kangoma S, Nasasara K, Zaina LM, Lupafya C, Mziya J, Harries AD, Takarinda KC, Kwataine M, Dambula I. Can a village headman use an electronic village register and a simplified community-based verbal autopsy tool to record numbers and causes of death in rural Malawi? [Unpublished data]. Front Public Health. 2018; 6(246): 1-11.

National Administrative Department of Statistics (DANE) (Colombia). Colombia Vital Statistics - Deaths 2008. BogotÃ¡, Colombia: National Administrative Department of Statistics (DANE) (Colombia).
National Administrative Department of Statistics (DANE) (Colombia). Colombia Vital Statistics - Deaths 2016. BogotÃ¡, Colombia: National Administrative Department of Statistics (DANE) (Colombia).
National Administrative Department of Statistics (DANE) (Colombia). Colombia Vital Statistics - Deaths 2017. BogotÃ¡, Colombia: National Administrative Department of Statistics (DANE) (Colombia).
National Administrative Department of Statistics (DANE) (Colombia). Colombia Vital Statistics - Deaths 2018. BogotÃ¡, Colombia: National Administrative Department of Statistics (DANE) (Colombia).
National Administrative Department of Statistics (DANE) (Colombia). Colombia Vital Statistics - Deaths 2019. BogotÃ¡, Colombia: National Administrative Department of Statistics (DANE) (Colombia).
National Board of Health and Welfare (Sweden). Sweden - Stockholm County Vital Registration - Deaths 1980.
National Board of Health and Welfare (Sweden). Sweden - Stockholm County Vital Registration - Deaths 1981.
National Board of Health and Welfare (Sweden). Sweden - Stockholm County Vital Registration - Deaths 1982.
National Board of Health and Welfare (Sweden). Sweden - Stockholm County Vital Registration - Deaths 1983.
National Board of Health and Welfare (Sweden). Sweden - Stockholm County Vital Registration - Deaths 1984.
National Board of Health and Welfare (Sweden). Sweden - Stockholm County Vital Registration - Deaths 1985.
National Board of Health and Welfare (Sweden). Sweden - Stockholm County Vital Registration - Deaths 1986.
National Board of Health and Welfare (Sweden). Sweden Cause of Death Register 1987.
National Board of Health and Welfare (Sweden). Sweden Cause of Death Register 1988.
National Board of Health and Welfare (Sweden). Sweden Cause of Death Register 1989.
National Board of Health and Welfare (Sweden). Sweden Cause of Death Register 1990.
National Board of Health and Welfare (Sweden). Sweden Cause of Death Register 1991.
National Board of Health and Welfare (Sweden). Sweden Cause of Death Register 1992.
National Board of Health and Welfare (Sweden). Sweden Cause of Death Register 1993.
National Board of Health and Welfare (Sweden). Sweden Cause of Death Register 1994.
National Board of Health and Welfare (Sweden). Sweden Cause of Death Register 1995.
National Board of Health and Welfare (Sweden). Sweden Cause of Death Register 1996.
National Board of Health and Welfare (Sweden). Sweden Cause of Death Register 1997.
National Board of Health and Welfare (Sweden). Sweden Cause of Death Register 1998.
National Board of Health and Welfare (Sweden). Sweden Cause of Death Register 1999.
National Board of Health and Welfare (Sweden). Sweden Cause of Death Register 2000.
National Board of Health and Welfare (Sweden). Sweden Cause of Death Register 2001.
National Board of Health and Welfare (Sweden). Sweden Cause of Death Register 2002.
National Board of Health and Welfare (Sweden). Sweden Cause of Death Register 2003.
National Board of Health and Welfare (Sweden). Sweden Cause of Death Register 2004.
National Board of Health and Welfare (Sweden). Sweden Cause of Death Register 2005.
National Board of Health and Welfare (Sweden). Sweden Cause of Death Register 2006.
National Board of Health and Welfare (Sweden). Sweden Cause of Death Register 2007.
National Board of Health and Welfare (Sweden). Sweden Cause of Death Register 2008.
National Board of Health and Welfare (Sweden). Sweden Cause of Death Register 2009.
National Board of Health and Welfare (Sweden). Sweden Cause of Death Register 2010.
National Board of Health and Welfare (Sweden). Sweden Cause of Death Register 2011.
National Board of Health and Welfare (Sweden). Sweden Cause of Death Register 2012. Stockholm, Sweden: National Board of Health and Welfare (Sweden).
National Board of Health and Welfare (Sweden). Sweden Cause of Death Register 2013. Stockholm, Sweden: National Board of Health and Welfare (Sweden).
National Board of Health and Welfare (Sweden). Sweden Cause of Death Register 2014. Stockholm, Sweden: National Board of Health and Welfare (Sweden).
National Board of Health and Welfare (Sweden). Sweden Cause of Death Register 2015. Stockholm, Sweden: National Board of Health and Welfare (Sweden).
National Board of Health and Welfare (Sweden). Sweden Cause of Death Register 2016. Stockholm, Sweden: National Board of Health and Welfare (Sweden).
National Board of Health and Welfare (Sweden). Sweden Cause of Death Register 2017. Stockholm, Sweden: National Board of Health and Welfare (Sweden).
National Board of Health and Welfare (Sweden). Sweden Cause of Death Register 2018. Stockholm, Sweden: National Board of Health and Welfare (Sweden).
National Board of Health and Welfare (Sweden). Sweden Cause of Death Register 2019. Stockholm, Sweden: National Board of Health and Welfare (Sweden).
National Board of Health and Welfare (Sweden). Sweden Vital Registration - Deaths 1980.
National Board of Health and Welfare (Sweden). Sweden Vital Registration - Deaths 1981.
National Board of Health and Welfare (Sweden). Sweden Vital Registration - Deaths 1982.
National Board of Health and Welfare (Sweden). Sweden Vital Registration - Deaths 1983.
National Board of Health and Welfare (Sweden). Sweden Vital Registration - Deaths 1984.
National Board of Health and Welfare (Sweden). Sweden Vital Registration - Deaths 1985.

National Board of Health and Welfare (Sweden). Sweden Vital Registration - Deaths 1986.

National Center for Health Statistics, Centers for Disease Control and Prevention. American Samoa NVSS Mortality Data 1997 - NBER. Hyattsville, United States: National Center for Health Statistics, Centers for Disease Control and Prevention.

National Center for Health Statistics, Centers for Disease Control and Prevention. American Samoa NVSS Mortality Data 1998 - NBER. Hyattsville, United States: National Center for Health Statistics, Centers for Disease Control and Prevention.

National Center for Health Statistics, Centers for Disease Control and Prevention. American Samoa NVSS Mortality Data 1999 - NBER. Hyattsville, United States: National Center for Health Statistics, Centers for Disease Control and Prevention.

National Center for Health Statistics, Centers for Disease Control and Prevention. American Samoa NVSS Mortality Data 2000 - NBER. Hyattsville, United States of America: National Center for Health Statistics, Centers for Disease Control and Prevention.

National Center for Health Statistics, Centers for Disease Control and Prevention. American Samoa NVSS Mortality Data 2001 - NBER. Hyattsville, United States of America: National Center for Health Statistics, Centers for Disease Control and Prevention.

National Center for Health Statistics, Centers for Disease Control and Prevention. American Samoa NVSS Mortality Data 2002 - NBER. Hyattsville, United States of America: National Center for Health Statistics, Centers for Disease Control and Prevention.

National Center for Health Statistics, Centers for Disease Control and Prevention. American Samoa NVSS Mortality Data 2003 - NBER. Hyattsville, United States of America: National Center for Health Statistics, Centers for Disease Control and Prevention.

National Center for Health Statistics, Centers for Disease Control and Prevention. American Samoa NVSS Mortality Data 2004 - NBER. Hyattsville, United States of America: National Center for Health Statistics, Centers for Disease Control and Prevention.

National Center for Health Statistics, Centers for Disease Control and Prevention. Guam NVSS Mortality Data 1994 - NBER. Hyattsville, United States: National Center for Health Statistics, Centers for Disease Control and Prevention.

National Center for Health Statistics, Centers for Disease Control and Prevention. Guam NVSS Mortality Data 1995 - NBER. Hyattsville, United States: National Center for Health Statistics, Centers for Disease Control and Prevention.

National Center for Health Statistics, Centers for Disease Control and Prevention. Guam NVSS Mortality Data 1996 - NBER. Hyattsville, United States: National Center for Health Statistics, Centers for Disease Control and Prevention.

National Center for Health Statistics, Centers for Disease Control and Prevention. Guam NVSS Mortality Data 1997 - NBER. Hyattsville, United States: National Center for Health Statistics, Centers for Disease Control and Prevention.

National Center for Health Statistics, Centers for Disease Control and Prevention. Guam NVSS Mortality Data 1998 - NBER. Hyattsville, United States: National Center for Health Statistics, Centers for Disease Control and Prevention.

National Center for Health Statistics, Centers for Disease Control and Prevention. Guam NVSS Mortality Data 1999 - NBER. Hyattsville, United States: National Center for Health Statistics, Centers for Disease Control and Prevention.

National Center for Health Statistics, Centers for Disease Control and Prevention. Guam NVSS Mortality Data 2000 - NBER. Hyattsville, United States of America: National Center for Health Statistics, Centers for Disease Control and Prevention.

National Center for Health Statistics, Centers for Disease Control and Prevention. Guam NVSS Mortality Data 2001 - NBER. Hyattsville, United States of America: National Center for Health Statistics, Centers for Disease Control and Prevention.

National Center for Health Statistics, Centers for Disease Control and Prevention. Guam NVSS Mortality Data 2002 - NBER. Hyattsville, United States of America: National Center for Health Statistics, Centers for Disease Control and Prevention.

National Center for Health Statistics, Centers for Disease Control and Prevention. Guam NVSS Mortality Data 2003 - NBER. Hyattsville, United States of America: National Center for Health Statistics, Centers for Disease Control and Prevention.

National Center for Health Statistics, Centers for Disease Control and Prevention. Guam NVSS Mortality Data 2004 - NBER. Hyattsville, United States of America: National Center for Health Statistics, Centers for Disease Control and Prevention.

National Center for Health Statistics, Centers for Disease Control and Prevention. Northern Mariana Islands NVSS Mortality Data 1998 - NBER. Hyattsville, United States: National Center for Health Statistics, Centers for Disease Control and Prevention.

National Center for Health Statistics, Centers for Disease Control and Prevention. Northern Mariana Islands NVSS Mortality Data 1999 - NBER. Hyattsville, United States: National Center for Health Statistics, Centers for Disease Control and Prevention.

National Center for Health Statistics, Centers for Disease Control and Prevention. Northern Mariana Islands NVSS Mortality Data 2000 - NBER. Hyattsville, United States of America: National Center for Health Statistics, Centers for Disease Control and Prevention.

National Center for Health Statistics, Centers for Disease Control and Prevention. Northern Mariana Islands NVSS Mortality Data 2001 - NBER. Hyattsville, United States of America: National Center for Health Statistics, Centers for Disease Control and Prevention.

National Center for Health Statistics, Centers for Disease Control and Prevention. Northern Mariana Islands NVSS Mortality Data 2002 - NBER. Hyattsville, United States of America: National Center for Health Statistics, Centers for Disease Control and Prevention.

National Center for Health Statistics, Centers for Disease Control and Prevention. Northern Mariana Islands NVSS Mortality Data 2003 - NBER. Hyattsville, United States of America: National Center for Health Statistics, Centers for Disease Control and Prevention.

National Center for Health Statistics, Centers for Disease Control and Prevention. Northern Mariana Islands NVSS Mortality Data 2004 - NBER. Hyattsville, United States of America: National Center for Health Statistics, Centers for Disease Control and Prevention.

National Center for Health Statistics, Centers for Disease Control and Prevention. Puerto Rico NVSS Mortality Data 1994 - NBER. Hyattsville, United States: National Center for Health Statistics, Centers for Disease Control and Prevention.

National Center for Health Statistics, Centers for Disease Control and Prevention. Puerto Rico NVSS Mortality Data 1995 - NBER. Hyattsville, United States: National Center for Health Statistics, Centers for Disease Control and Prevention.

National Center for Health Statistics, Centers for Disease Control and Prevention. Puerto Rico NVSS Mortality Data 1996 - NBER. Hyattsville, United States: National Center for Health Statistics, Centers for Disease Control and Prevention.

National Center for Health Statistics, Centers for Disease Control and Prevention. Puerto Rico NVSS Mortality Data 1997 - NBER. Hyattsville, United States: National Center for Health Statistics, Centers for Disease Control and Prevention.

National Center for Health Statistics, Centers for Disease Control and Prevention. Puerto Rico NVSS Mortality Data 1998 - NBER. Hyattsville, United States: National Center for Health Statistics, Centers for Disease Control and Prevention.

National Center for Health Statistics, Centers for Disease Control and Prevention. Puerto Rico NVSS Mortality Data 1999 - NBER. Hyattsville, United States: National Center for Health Statistics, Centers for Disease Control and Prevention.

National Center for Health Statistics, Centers for Disease Control and Prevention. Puerto Rico NVSS Mortality Data 2000 - NBER. Hyattsville, United States of America: National Center for Health Statistics, Centers for Disease Control and Prevention.

National Center for Health Statistics, Centers for Disease Control and Prevention. Puerto Rico NVSS Mortality Data 2001 - NBER. Hyattsville, United States of America: National Center for Health Statistics, Centers for Disease Control and Prevention.

National Center for Health Statistics, Centers for Disease Control and Prevention. Puerto Rico NVSS Mortality Data 2002 - NBER. Hyattsville, United States of America: National Center for Health Statistics, Centers for Disease Control and Prevention.

National Center for Health Statistics, Centers for Disease Control and Prevention. Puerto Rico NVSS Mortality Data 2003 - NBER. Hyattsville, United States of America: National Center for Health Statistics, Centers for Disease Control and Prevention.

National Center for Health Statistics, Centers for Disease Control and Prevention. Puerto Rico NVSS Mortality Data 2004 - NBER. Hyattsville, United States of America: National Center for Health Statistics, Centers for Disease Control and Prevention.

National Center for Health Statistics, Centers for Disease Control and Prevention. United States NVSS Mortality Data 1980 - NBER. Hyattsville, United States of America: National Center for Health Statistics, Centers for Disease Control and Prevention.

National Center for Health Statistics, Centers for Disease Control and Prevention. United States NVSS Mortality Data 1981 - NBER. Hyattsville, United States of America: National Center for Health Statistics, Centers for Disease Control and Prevention.

National Center for Health Statistics, Centers for Disease Control and Prevention. United States NVSS Mortality Data 1982 - NBER. Hyattsville, United States of America: National Center for Health Statistics, Centers for Disease Control and Prevention.

National Center for Health Statistics, Centers for Disease Control and Prevention. United States NVSS Mortality Data 1983 - NBER. Hyattsville, United States of America: National Center for Health Statistics, Centers for Disease Control and Prevention.

National Center for Health Statistics, Centers for Disease Control and Prevention. United States NVSS Mortality Data 1984 - NBER. Hyattsville, United States of America: National Center for Health Statistics, Centers for Disease Control and Prevention.

National Center for Health Statistics, Centers for Disease Control and Prevention. United States NVSS Mortality Data 1985 - NBER. Hyattsville, United States of America: National Center for Health Statistics, Centers for Disease Control and Prevention.

National Center for Health Statistics, Centers for Disease Control and Prevention. United States NVSS Mortality Data 1986 - NBER. Hyattsville, United States of America: National Center for Health Statistics, Centers for Disease Control and Prevention.

National Center for Health Statistics, Centers for Disease Control and Prevention. United States NVSS Mortality Data 1987 - NBER. Hyattsville, United States of America: National Center for Health Statistics, Centers for Disease Control and Prevention.

National Center for Health Statistics, Centers for Disease Control and Prevention. United States NVSS Mortality Data 1988 - NBER. Hyattsville, United States of America: National Center for Health Statistics, Centers for Disease Control and Prevention.

National Center for Health Statistics, Centers for Disease Control and Prevention. United States NVSS Mortality Data 1989 - NBER. Hyattsville, United States of America: National Center for Health Statistics, Centers for Disease Control and Prevention.

National Center for Health Statistics, Centers for Disease Control and Prevention. United States NVSS Mortality Data 1990 - NBER. Hyattsville, United States of America: National Center for Health Statistics, Centers for Disease Control and Prevention.

National Center for Health Statistics, Centers for Disease Control and Prevention. United States NVSS Mortality Data 1991 - NBER. Hyattsville, United States of America: National Center for Health Statistics, Centers for Disease Control and Prevention.

National Center for Health Statistics, Centers for Disease Control and Prevention. United States NVSS Mortality Data 1992 - NBER. Hyattsville, United States of America: National Center for Health Statistics, Centers for Disease Control and Prevention.

National Center for Health Statistics, Centers for Disease Control and Prevention. United States NVSS Mortality Data 1993 - NBER. Hyattsville, United States of America: National Center for Health Statistics, Centers for Disease Control and Prevention.

National Center for Health Statistics, Centers for Disease Control and Prevention. United States NVSS Mortality Data 1994 - NBER. Hyattsville, United States of America: National Center for Health Statistics, Centers for Disease Control and Prevention.

National Center for Health Statistics, Centers for Disease Control and Prevention. United States NVSS Mortality Data 1995 - NBER. Hyattsville, United States of America: National Center for Health Statistics, Centers for Disease Control and Prevention.

National Center for Health Statistics, Centers for Disease Control and Prevention. United States NVSS Mortality Data 1996 - NBER. Hyattsville, United States of America: National Center for Health Statistics, Centers for Disease Control and Prevention.

National Center for Health Statistics, Centers for Disease Control and Prevention. United States NVSS Mortality Data 1997 - NBER. Hyattsville, United States of America: National Center for Health Statistics, Centers for Disease Control and Prevention.

National Center for Health Statistics, Centers for Disease Control and Prevention. United States NVSS Mortality Data 1998 - NBER. Hyattsville, United States of America: National Center for Health Statistics, Centers for Disease Control and Prevention.

National Center for Health Statistics, Centers for Disease Control and Prevention. United States NVSS Mortality Data 1999 - NBER. Hyattsville, United States of America: National Center for Health Statistics, Centers for Disease Control and Prevention.

National Center for Health Statistics, Centers for Disease Control and Prevention. United States NVSS Mortality Data 2000 - NBER. Hyattsville, United States of America: National Center for Health Statistics, Centers for Disease Control and Prevention.

National Center for Health Statistics, Centers for Disease Control and Prevention. United States NVSS Mortality Data 2001 - NBER. Hyattsville, United States of America: National Center for Health Statistics, Centers for Disease Control and Prevention.

National Center for Health Statistics, Centers for Disease Control and Prevention. United States NVSS Mortality Data 2002 - NBER. Hyattsville, United States of America: National Center for Health Statistics, Centers for Disease Control and Prevention.

National Center for Health Statistics, Centers for Disease Control and Prevention. United States NVSS Mortality Data 2003 - NBER. Hyattsville, United States of America: National Center for Health Statistics, Centers for Disease Control and Prevention.

National Center for Health Statistics, Centers for Disease Control and Prevention. United States NVSS Mortality Data 2004 - NBER. Hyattsville, United States of America: National Center for Health Statistics, Centers for Disease Control and Prevention.

National Center for Health Statistics, Centers for Disease Control and Prevention. United States Virgin Islands NVSS Mortality Data 1994 - NBER. Hyattsville, United States: National Center for Health Statistics, Centers for Disease Control and Prevention.

National Center for Health Statistics, Centers for Disease Control and Prevention. United States Virgin Islands NVSS Mortality Data 1995 - NBER. Hyattsville, United States: National Center for Health Statistics, Centers for Disease Control and Prevention.

National Center for Health Statistics, Centers for Disease Control and Prevention. United States Virgin Islands NVSS Mortality Data 1996 - NBER. Hyattsville, United States: National Center for Health Statistics, Centers for Disease Control and Prevention.

National Center for Health Statistics, Centers for Disease Control and Prevention. United States Virgin Islands NVSS Mortality Data 1997 - NBER. Hyattsville, United States: National Center for Health Statistics, Centers for Disease Control and Prevention.

National Center for Health Statistics, Centers for Disease Control and Prevention. United States Virgin Islands NVSS Mortality Data 1998 - NBER. Hyattsville, United States: National Center for Health Statistics, Centers for Disease Control and Prevention.

National Center for Health Statistics, Centers for Disease Control and Prevention. United States Virgin Islands NVSS Mortality Data 1999 - NBER. Hyattsville, United States: National Center for Health Statistics, Centers for Disease Control and Prevention.

National Center for Health Statistics, Centers for Disease Control and Prevention. United States Virgin Islands NVSS Mortality Data 2000 - NBER. Hyattsville, United States of America: National Center for Health Statistics, Centers for Disease Control and Prevention.
National Center for Health Statistics, Centers for Disease Control and Prevention. United States Virgin Islands NVSS Mortality Data 2001 - NBER. Hyattsville, United States of America: National Center for Health Statistics, Centers for Disease Control and Prevention.
National Center for Health Statistics, Centers for Disease Control and Prevention. United States Virgin Islands NVSS Mortality Data 2002 - NBER. Hyattsville, United States of America: National Center for Health Statistics, Centers for Disease Control and Prevention.
National Center for Health Statistics, Centers for Disease Control and Prevention. United States Virgin Islands NVSS Mortality Data 2003 - NBER. Hyattsville, United States of America: National Center for Health Statistics, Centers for Disease Control and Prevention.
National Center for Health Statistics, Centers for Disease Control and Prevention. United States Virgin Islands NVSS Mortality Data 2004 - NBER. Hyattsville, United States of America: National Center for Health Statistics, Centers for Disease Control and Prevention.
National Institute of Statistics and Censuses (Costa Rica). Costa Rica Registered Deaths 2015. San JosÃ©, Costa Rica: National Institute of Statistics and Censuses (Costa Rica).
National Institute of Statistics and Censuses (Costa Rica). Costa Rica Registered Deaths 2016. San JosÃ©, Costa Rica: National Institute of Statistics and Censuses (Costa Rica).
National Institute of Statistics and Censuses (Costa Rica). Costa Rica Registered Deaths 2017. San JosÃ©, Costa Rica: National Institute of Statistics and Censuses (Costa Rica).
National Institute of Statistics and Censuses (Costa Rica). Costa Rica Registered Deaths 2018. San JosÃ©, Costa Rica: National Institute of Statistics and Censuses (Costa Rica).
National Institute of Statistics and Censuses (Costa Rica). Costa Rica Registered Deaths 2019. San JosÃ©, Costa Rica: National Institute of Statistics and Censuses (Costa Rica).
National Institute of Statistics and Censuses (Ecuador). Ecuador General Deaths 2017. Quito, Ecuador: National Institute of Statistics and Censuses (Ecuador).
National Institute of Statistics and Censuses (Ecuador). Ecuador General Deaths 2018. Quito, Ecuador: National Institute of Statistics and Censuses (Ecuador).
National Institute of Statistics and Censuses (Ecuador). Ecuador General Deaths 2019. Quito, Ecuador: National Institute of Statistics and Censuses (Ecuador).
National Institute of Statistics and Geography (INEGI) (Mexico), Secretariat of Health (Mexico). Mexico Vital Registration - Deaths 1980.
National Institute of Statistics and Geography (INEGI) (Mexico), Secretariat of Health (Mexico). Mexico Vital Registration - Deaths 1981.
National Institute of Statistics and Geography (INEGI) (Mexico), Secretariat of Health (Mexico). Mexico Vital Registration - Deaths 1982.
National Institute of Statistics and Geography (INEGI) (Mexico), Secretariat of Health (Mexico). Mexico Vital Registration - Deaths 1983.
National Institute of Statistics and Geography (INEGI) (Mexico), Secretariat of Health (Mexico). Mexico Vital Registration - Deaths 1984.
National Institute of Statistics and Geography (INEGI) (Mexico), Secretariat of Health (Mexico). Mexico Vital Registration - Deaths 1985.
National Institute of Statistics and Geography (INEGI) (Mexico), Secretariat of Health (Mexico). Mexico Vital Registration - Deaths 1986.
National Institute of Statistics and Geography (INEGI) (Mexico), Secretariat of Health (Mexico). Mexico Vital Registration - Deaths 1987.
National Institute of Statistics and Geography (INEGI) (Mexico), Secretariat of Health (Mexico). Mexico Vital Registration - Deaths 1988.
National Institute of Statistics and Geography (INEGI) (Mexico), Secretariat of Health (Mexico). Mexico Vital Registration - Deaths 1989.
National Institute of Statistics and Geography (INEGI) (Mexico), Secretariat of Health (Mexico). Mexico Vital Registration - Deaths 1990.
National Institute of Statistics and Geography (INEGI) (Mexico), Secretariat of Health (Mexico). Mexico Vital Registration - Deaths 1991.
National Institute of Statistics and Geography (INEGI) (Mexico), Secretariat of Health (Mexico). Mexico Vital Registration - Deaths 1992.
National Institute of Statistics and Geography (INEGI) (Mexico), Secretariat of Health (Mexico). Mexico Vital Registration - Deaths 1993.
National Institute of Statistics and Geography (INEGI) (Mexico), Secretariat of Health (Mexico). Mexico Vital Registration - Deaths 1994.
National Institute of Statistics and Geography (INEGI) (Mexico), Secretariat of Health (Mexico). Mexico Vital Registration - Deaths 1995.
National Institute of Statistics and Geography (INEGI) (Mexico), Secretariat of Health (Mexico). Mexico Vital Registration - Deaths 1996.
National Institute of Statistics and Geography (INEGI) (Mexico), Secretariat of Health (Mexico). Mexico Vital Registration - Deaths 1997.
National Institute of Statistics and Geography (INEGI) (Mexico), Secretariat of Health (Mexico). Mexico Vital Registration - Deaths 1998.
National Institute of Statistics and Geography (INEGI) (Mexico), Secretariat of Health (Mexico). Mexico Vital Registration - Deaths 1999.
National Institute of Statistics and Geography (INEGI) (Mexico), Secretariat of Health (Mexico). Mexico Vital Registration - Deaths 2000.
National Institute of Statistics and Geography (INEGI) (Mexico), Secretariat of Health (Mexico). Mexico Vital Registration - Deaths 2001.
National Institute of Statistics and Geography (INEGI) (Mexico), Secretariat of Health (Mexico). Mexico Vital Registration - Deaths 2002.
National Institute of Statistics and Geography (INEGI) (Mexico), Secretariat of Health (Mexico). Mexico Vital Registration - Deaths 2003.
National Institute of Statistics and Geography (INEGI) (Mexico), Secretariat of Health (Mexico). Mexico Vital Registration - Deaths 2004.
National Institute of Statistics and Geography (INEGI) (Mexico), Secretariat of Health (Mexico). Mexico Vital Registration - Deaths 2005.
National Institute of Statistics and Geography (INEGI) (Mexico), Secretariat of Health (Mexico). Mexico Vital Registration - Deaths 2006.
National Institute of Statistics and Geography (INEGI) (Mexico), Secretariat of Health (Mexico). Mexico Vital Registration - Deaths 2007.
National Institute of Statistics and Geography (INEGI) (Mexico), Secretariat of Health (Mexico). Mexico Vital Registration - Deaths 2008.
National Institute of Statistics and Geography (INEGI) (Mexico), Secretariat of Health (Mexico). Mexico Vital Registration - Deaths 2010.
National Institute of Statistics and Geography (INEGI) (Mexico), Secretariat of Health (Mexico). Mexico Vital Registration - Deaths 2011.
National Institute of Statistics and Geography (INEGI) (Mexico). Mexico Vital Registration - Deaths 2012.
National Institute of Statistics and Geography (INEGI) (Mexico). Mexico Vital Registration - Deaths 2013.
National Institute of Statistics and Geography (INEGI) (Mexico). Mexico Vital Registration - Deaths 2014. Mexico City, Mexico: National Institute of Statistics and Geography (INEGI) (Mexico).
National Institute of Statistics and Geography (INEGI) (Mexico). Mexico Vital Registration - Deaths 2015. Mexico City, Mexico: National Institute of Statistics and Geography (INEGI) (Mexico).

National Institute of Statistics and Geography (INEGI) (Mexico). Mexico Vital Registration - Deaths 2016. Mexico City, Mexico: National Institute of Statistics and Geography (INEGI) (Mexico).
National Institute of Statistics and Geography (INEGI) (Mexico). Mexico Vital Registration - Deaths 2017. Mexico City, Mexico: National Institute of Statistics and Geography (INEGI) (Mexico).
National Institute of Statistics and Geography (INEGI) (Mexico). Mexico Vital Registration - Deaths 2018. Mexico City, Mexico: National Institute of Statistics and Geography (INEGI) (Mexico).
National Institute of Statistics and Geography (INEGI) (Mexico). Mexico Vital Registration - Deaths 2019. Mexico City, Mexico: National Institute of Statistics and Geography (INEGI) (Mexico).
National Institute of Statistics and Geography (INEGI) (Mexico). Mexico Vital Statistics - Deaths 2009.
National Records of Scotland. United Kingdom - Scotland Vital Events Reference Tables 2015. Edinburgh, Scotland: National Records of Scotland, 2016.
National Records of Scotland. United Kingdom - Scotland Vital Events Reference Tables 2016. Edinburgh, Scotland: National Records of Scotland, 2017.
National Statistics Institute (Guatemala). Guatemala Vital Statistics 2010. Guatemala City, Guatemala: National Statistics Institute (Guatemala), 2011.
National Statistics Institute (Guatemala). Guatemala Vital Statistics 2011. Guatemala City, Guatemala: National Statistics Institute (Guatemala), 2012.
National Statistics Institute (Guatemala). Guatemala Vital Statistics 2012. Guatemala City, Guatemala: National Statistics Institute (Guatemala), 2013.
National Statistics Institute (Guatemala). Guatemala Vital Statistics 2013. Guatemala City, Guatemala: National Statistics Institute (Guatemala), 2014.
National Statistics Institute (Guatemala). Guatemala Vital Statistics 2014. Guatemala City, Guatemala: National Statistics Institute (Guatemala), 2015.
National Statistics Institute (Guatemala). Guatemala Vital Statistics 2015. Guatemala City, Guatemala: National Statistics Institute (Guatemala), 2016.
National Statistics Institute (Guatemala). Guatemala Vital Statistics 2016. Guatemala City, Guatemala: National Statistics Institute (Guatemala), 2017.
National Statistics Institute (Guatemala). Guatemala Vital Statistics 2017. Guatemala City, Guatemala: National Statistics Institute (Guatemala), 2018.
National Statistics Institute (Guatemala). Guatemala Vital Statistics 2018. Guatemala City, Guatemala: National Statistics Institute (Guatemala), 2019.
National Statistics Institute (Guatemala). Guatemala Vital Statistics 2019. Guatemala City, Guatemala: National Statistics Institute (Guatemala), 2020.
National Statistics Office (Philippines). Philippines Vital Registration - Deaths 2006.
National Statistics Office (Philippines). Philippines Vital Registration - Deaths 2007.
National Statistics Office (Philippines). Philippines Vital Registration - Deaths 2008.
National Statistics Office (Philippines). Philippines Vital Registration - Deaths 2009.
National Statistics Office (Philippines). Philippines Vital Registration - Deaths 2010.
National Statistics Office (Philippines). Philippines Vital Registration - Deaths 2011.
National Statistics Office (Philippines). Philippines Vital Registration - Deaths 2012.
Navrongo Health Research Centre. Ghana - Navrongo Health and Demographic Surveillance System.
Netherlands Vital Registration - Deaths 1980 ICD9.
Netherlands Vital Registration - Deaths 1981 ICD9.
Netherlands Vital Registration - Deaths 1982 ICD9.
Netherlands Vital Registration - Deaths 1983 ICD9.
Netherlands Vital Registration - Deaths 1984 ICD9.
Netherlands Vital Registration - Deaths 1985 ICD9.
Netherlands Vital Registration - Deaths 1986 ICD9.
Netherlands Vital Registration - Deaths 1987 ICD9.
Netherlands Vital Registration - Deaths 1988 ICD9.
Netherlands Vital Registration - Deaths 1989 ICD9.
Netherlands Vital Registration - Deaths 1990 ICD9.
Netherlands Vital Registration - Deaths 1991 ICD9.
Netherlands Vital Registration - Deaths 1992 ICD9.
Netherlands Vital Registration - Deaths 1993 ICD9.
Netherlands Vital Registration - Deaths 1994 ICD9.
Netherlands Vital Registration - Deaths 1995 ICD9.
Netherlands Vital Registration - Deaths 1996 ICD10.
Netherlands Vital Registration - Deaths 1997 ICD10.
Netherlands Vital Registration - Deaths 1998 ICD10.
Netherlands Vital Registration - Deaths 1999 ICD10.
Netherlands Vital Registration - Deaths 2000 ICD10.
Netherlands Vital Registration - Deaths 2001 ICD10.
Netherlands Vital Registration - Deaths 2002 ICD10.

Netherlands Vital Registration - Deaths 2003 ICD10.
Netherlands Vital Registration - Deaths 2004 ICD10.
Netherlands Vital Registration - Deaths 2005 ICD10.
Netherlands Vital Registration - Deaths 2006 ICD10.
Netherlands Vital Registration - Deaths 2007 ICD10.
Netherlands Vital Registration - Deaths 2008 ICD10.
Netherlands Vital Registration - Deaths 2009 ICD10.
Netherlands Vital Registration - Deaths 2010 ICD10.
Netherlands Vital Registration - Deaths 2011 ICD10.
Netherlands Vital Registration - Deaths 2012 ICD10.
Netherlands Vital Registration - Deaths 2013 ICD10.
Netherlands Vital Registration - Deaths 2014 ICD10.
Netherlands Vital Registration - Deaths 2015 ICD10.
Netherlands Vital Registration - Deaths 2016 ICD10.
Netherlands Vital Registration - Deaths 2017 ICD10.
Netherlands Vital Registration - Deaths 2018 ICD10.
Nicaragua Vital Registration - Deaths 1988 ICD9.
Nicaragua Vital Registration - Deaths 1989 ICD9.
Nicaragua Vital Registration - Deaths 1990 ICD9.
Nicaragua Vital Registration - Deaths 1991 ICD9.
Nicaragua Vital Registration - Deaths 1992 ICD9.
Nicaragua Vital Registration - Deaths 1993 ICD9.
Nicaragua Vital Registration - Deaths 1994 ICD9.
Nicaragua Vital Registration - Deaths 1996 ICD9.
Nicaragua Vital Registration - Deaths 1997 ICD10.
Nicaragua Vital Registration - Deaths 1998 ICD10.
Nicaragua Vital Registration - Deaths 1999 ICD10.
Nicaragua Vital Registration - Deaths 2000 ICD10.
Nicaragua Vital Registration - Deaths 2001 ICD10.
Nicaragua Vital Registration - Deaths 2002 ICD10.
Nicaragua Vital Registration - Deaths 2003 ICD10.
Nicaragua Vital Registration - Deaths 2004 ICD10.
Nicaragua Vital Registration - Deaths 2005 ICD10.
Nicaragua Vital Registration - Deaths 2006 ICD10.
Nicaragua Vital Registration - Deaths 2007 ICD10.
Nicaragua Vital Registration - Deaths 2008 ICD10.
Nicaragua Vital Registration - Deaths 2009 ICD10.
Nicaragua Vital Registration - Deaths 2010 ICD10.
Nicaragua Vital Registration - Deaths 2011 ICD10.
Nicaragua Vital Registration - Deaths 2012 ICD10.
Nicaragua Vital Registration - Deaths 2013 ICD10.
Nicaragua Vital Registration - Deaths 2014 ICD10.
Nicaragua Vital Registration - Deaths 2015 ICD10.
Nicaragua Vital Registration - Deaths 2016 ICD10.
Nicaragua Vital Registration - Deaths 2017 ICD10.
Nicaragua Vital Registration - Deaths 2018 ICD10.
Norwegian Institute of Public Health. Norway Cause of Death Registry 1980. Oslo, Norway: Norwegian Institute of Public Health.
Norwegian Institute of Public Health. Norway Cause of Death Registry 1981. Oslo, Norway: Norwegian Institute of Public Health.
Norwegian Institute of Public Health. Norway Cause of Death Registry 1982. Oslo, Norway: Norwegian Institute of Public Health.
Norwegian Institute of Public Health. Norway Cause of Death Registry 1983. Oslo, Norway: Norwegian Institute of Public Health.
Norwegian Institute of Public Health. Norway Cause of Death Registry 1984. Oslo, Norway: Norwegian Institute of Public Health.
Norwegian Institute of Public Health. Norway Cause of Death Registry 1985. Oslo, Norway: Norwegian Institute of Public Health.
Norwegian Institute of Public Health. Norway Cause of Death Registry 1986. Oslo, Norway: Norwegian Institute of Public Health.
Norwegian Institute of Public Health. Norway Cause of Death Registry 1987. Oslo, Norway: Norwegian Institute of Public Health.
Norwegian Institute of Public Health. Norway Cause of Death Registry 1988. Oslo, Norway: Norwegian Institute of Public Health.
Norwegian Institute of Public Health. Norway Cause of Death Registry 1989. Oslo, Norway: Norwegian Institute of Public Health.
Norwegian Institute of Public Health. Norway Cause of Death Registry 1990. Oslo, Norway: Norwegian Institute of Public Health.
Norwegian Institute of Public Health. Norway Cause of Death Registry 1991. Oslo, Norway: Norwegian Institute of Public Health.
Norwegian Institute of Public Health. Norway Cause of Death Registry 1992. Oslo, Norway: Norwegian Institute of Public Health.
Norwegian Institute of Public Health. Norway Cause of Death Registry 1993. Oslo, Norway: Norwegian Institute of Public Health.
Norwegian Institute of Public Health. Norway Cause of Death Registry 1994. Oslo, Norway: Norwegian Institute of Public Health.

Norwegian Institute of Public Health. Norway Cause of Death Registry 1995. Oslo, Norway: Norwegian Institute of Public Health.

Norwegian Institute of Public Health. Norway Cause of Death Registry 1996. Oslo, Norway: Norwegian Institute of Public Health.

Norwegian Institute of Public Health. Norway Cause of Death Registry 1997. Oslo, Norway: Norwegian Institute of Public Health.

Norwegian Institute of Public Health. Norway Cause of Death Registry 1998. Oslo, Norway: Norwegian Institute of Public Health.

Norwegian Institute of Public Health. Norway Cause of Death Registry 1999. Oslo, Norway: Norwegian Institute of Public Health.

Norwegian Institute of Public Health. Norway Cause of Death Registry 2000. Oslo, Norway: Norwegian Institute of Public Health.

Norwegian Institute of Public Health. Norway Cause of Death Registry 2001. Oslo, Norway: Norwegian Institute of Public Health.

Norwegian Institute of Public Health. Norway Cause of Death Registry 2002. Oslo, Norway: Norwegian Institute of Public Health.

Norwegian Institute of Public Health. Norway Cause of Death Registry 2003. Oslo, Norway: Norwegian Institute of Public Health.

Norwegian Institute of Public Health. Norway Cause of Death Registry 2004. Oslo, Norway: Norwegian Institute of Public Health.

Norwegian Institute of Public Health. Norway Cause of Death Registry 2005. Oslo, Norway: Norwegian Institute of Public Health.

Norwegian Institute of Public Health. Norway Cause of Death Registry 2006. Oslo, Norway: Norwegian Institute of Public Health.

Norwegian Institute of Public Health. Norway Cause of Death Registry 2007. Oslo, Norway: Norwegian Institute of Public Health.

Norwegian Institute of Public Health. Norway Cause of Death Registry 2008. Oslo, Norway: Norwegian Institute of Public Health.

Norwegian Institute of Public Health. Norway Cause of Death Registry 2009. Oslo, Norway: Norwegian Institute of Public Health.

Norwegian Institute of Public Health. Norway Cause of Death Registry 2010. Oslo, Norway: Norwegian Institute of Public Health.

Norwegian Institute of Public Health. Norway Cause of Death Registry 2011. Oslo, Norway: Norwegian Institute of Public Health.

Norwegian Institute of Public Health. Norway Cause of Death Registry 2012. Oslo, Norway: Norwegian Institute of Public Health.

Norwegian Institute of Public Health. Norway Cause of Death Registry 2013. Oslo, Norway: Norwegian Institute of Public Health.

Norwegian Institute of Public Health. Norway Cause of Death Registry 2014. Oslo, Norway: Norwegian Institute of Public Health.

Norwegian Institute of Public Health. Norway Cause of Death Registry 2015. Oslo, Norway: Norwegian Institute of Public Health.

Norwegian Institute of Public Health. Norway Cause of Death Registry 2016. Oslo, Norway: Norwegian Institute of Public Health.

Norwegian Institute of Public Health. Norway Cause of Death Registry 2017. Oslo, Norway: Norwegian Institute of Public Health.

Norwegian Institute of Public Health. Norway Cause of Death Registry 2018. Oslo, Norway: Norwegian Institute of Public Health.

Norwegian Institute of Public Health. Norway Cause of Death Registry 2019. Oslo, Norway: Norwegian Institute of Public Health.

Office for National Statistics (ONS) (United Kingdom). United Kingdom - England and Wales Mortality Data 1981-1994.

Office for National Statistics (ONS) (United Kingdom). United Kingdom - England and Wales Mortality Data 1995-2000.

Office for National Statistics (ONS) (United Kingdom). United Kingdom - England and Wales Mortality Data 2001-2012.

Office for National Statistics (ONS) (United Kingdom). United Kingdom - England and Wales Mortality Statistics 2013.

Office for National Statistics (ONS) (United Kingdom). United Kingdom - England and Wales Mortality Statistics 2014.

Office for National Statistics (ONS) (United Kingdom). United Kingdom - England and Wales Mortality Statistics 2015.

Office for National Statistics (ONS) (United Kingdom). United Kingdom - England and Wales Mortality Statistics 2016.

Office for National Statistics (ONS) (United Kingdom). United Kingdom - England and Wales Mortality Statistics 2017.

Office for National Statistics (ONS) (United Kingdom). United Kingdom - England and Wales Mortality Statistics 2018.

Office for National Statistics (ONS) (United Kingdom). United Kingdom - England and Wales Mortality Statistics 2019.

Office for National Statistics (ONS) (United Kingdom). United Kingdom - England Mortality Statistics 1981.

Office for National Statistics (ONS) (United Kingdom). United Kingdom - England Mortality Statistics 1982.

Office for National Statistics (ONS) (United Kingdom). United Kingdom - England Mortality Statistics 1983.

Office for National Statistics (ONS) (United Kingdom). United Kingdom - England Mortality Statistics 1984.

Office for National Statistics (ONS) (United Kingdom). United Kingdom - England Mortality Statistics 1985.

Office for National Statistics (ONS) (United Kingdom). United Kingdom - England Mortality Statistics 1986.

Office for National Statistics (ONS) (United Kingdom). United Kingdom - England Mortality Statistics 1987.

Office for National Statistics (ONS) (United Kingdom). United Kingdom - England Mortality Statistics 1988.

Office for National Statistics (ONS) (United Kingdom). United Kingdom - England Mortality Statistics 1989.

Office for National Statistics (ONS) (United Kingdom). United Kingdom - England Mortality Statistics 1990.

Office for National Statistics (ONS) (United Kingdom). United Kingdom - England Mortality Statistics 1991.

Office for National Statistics (ONS) (United Kingdom). United Kingdom - England Mortality Statistics 1992.

Office for National Statistics (ONS) (United Kingdom). United Kingdom - England Mortality Statistics 1993.

Office for National Statistics (ONS) (United Kingdom). United Kingdom - England Mortality Statistics 1994.

Office for National Statistics (ONS) (United Kingdom). United Kingdom - England Mortality Statistics 1995.

Office for National Statistics (ONS) (United Kingdom). United Kingdom - England Mortality Statistics 1996.

Office for National Statistics (ONS) (United Kingdom). United Kingdom - England Mortality Statistics 1997.

Office for National Statistics (ONS) (United Kingdom). United Kingdom - England Mortality Statistics 1998.

Office for National Statistics (ONS) (United Kingdom). United Kingdom - England Mortality Statistics 1999.

Office for National Statistics (ONS) (United Kingdom). United Kingdom - England Mortality Statistics 2000.

Office for National Statistics (ONS) (United Kingdom). United Kingdom - England Mortality Statistics 2001.

Office for National Statistics (ONS) (United Kingdom). United Kingdom - England Mortality Statistics 2002.

Office for National Statistics (ONS) (United Kingdom). United Kingdom - England Mortality Statistics 2003.

Office for National Statistics (ONS) (United Kingdom). United Kingdom - England Mortality Statistics 2004.

Office for National Statistics (ONS) (United Kingdom). United Kingdom - England Mortality Statistics 2005.

Office for National Statistics (ONS) (United Kingdom). United Kingdom - England Mortality Statistics 2006.

Office for National Statistics (ONS) (United Kingdom). United Kingdom - England Mortality Statistics 2007.
Office for National Statistics (ONS) (United Kingdom). United Kingdom - England Mortality Statistics 2008.
Office for National Statistics (ONS) (United Kingdom). United Kingdom - England Mortality Statistics 2009.
Office for National Statistics (ONS) (United Kingdom). United Kingdom - England Mortality Statistics 2010.
Office for National Statistics (ONS) (United Kingdom). United Kingdom - England Mortality Statistics 2011.
Office for National Statistics (ONS) (United Kingdom). United Kingdom - England Mortality Statistics 2012.
Office of Civil Service (Monaco), Government of Monaco. Monaco Vital Registration - Causes of Death 2011.
Office of Civil Service (Monaco), Government of Monaco. Monaco Vital Registration - Causes of Death 2012.
Office of Civil Service (Monaco), Government of Monaco. Monaco Vital Registration - Causes of Death 2013.
Oman Vital Registration - Deaths 2009 ICD10.
Oman Vital Registration - Deaths 2016 ICD10.
Palestine - West Bank and Gaza Strip Vital Registration - Deaths 2010 ICD10.
Palestine - West Bank and Gaza Strip Vital Registration - Deaths 2011 ICD10.
Palestine Vital Registration - Deaths 2008 ICD10.
Palestine Vital Registration - Deaths 2009 ICD10.
Palestinian Central Bureau of Statistics. Palestine - West Bank Vital Registration - Deaths 1997.
Palestinian Central Bureau of Statistics. Palestine - West Bank Vital Registration - Deaths 1998.
Palestinian Central Bureau of Statistics. Palestine - West Bank Vital Registration - Deaths 1999.
Palestinian Central Bureau of Statistics. Palestine - West Bank Vital Registration - Deaths 2000.
Palestinian Central Bureau of Statistics. Palestine - West Bank Vital Registration - Deaths 2001.
Palestinian Central Bureau of Statistics. Palestine - West Bank Vital Registration - Deaths 2002.
Palestinian Central Bureau of Statistics. Palestine - West Bank Vital Registration - Deaths 2003.
Palestinian Central Bureau of Statistics. Palestine - West Bank Vital Registration - Deaths 2004.
Palestinian Central Bureau of Statistics. Palestine - West Bank Vital Registration - Deaths 2005.
Palestinian Central Bureau of Statistics. Palestine - West Bank Vital Registration - Deaths 2007.
Panama Vital Registration - Deaths 1980 ICD9.
Panama Vital Registration - Deaths 1981 ICD9.
Panama Vital Registration - Deaths 1982 ICD9.
Panama Vital Registration - Deaths 1983 ICD9.
Panama Vital Registration - Deaths 1984 ICD9.
Panama Vital Registration - Deaths 1985 ICD9.
Panama Vital Registration - Deaths 1986 ICD9.
Panama Vital Registration - Deaths 1987 ICD9.
Panama Vital Registration - Deaths 1988 ICD9.
Panama Vital Registration - Deaths 1989 ICD9.
Panama Vital Registration - Deaths 1996 ICD9.
Panama Vital Registration - Deaths 1997 ICD9.
Panama Vital Registration - Deaths 1998 ICD10.
Panama Vital Registration - Deaths 1999 ICD10.
Panama Vital Registration - Deaths 2000 ICD10.
Panama Vital Registration - Deaths 2001 ICD10.
Panama Vital Registration - Deaths 2002 ICD10.
Panama Vital Registration - Deaths 2003 ICD10.
Panama Vital Registration - Deaths 2004 ICD10.
Panama Vital Registration - Deaths 2005 ICD10.
Panama Vital Registration - Deaths 2006 ICD10.
Panama Vital Registration - Deaths 2007 ICD10.
Panama Vital Registration - Deaths 2008 ICD10.
Panama Vital Registration - Deaths 2009 ICD10.
Panama Vital Registration - Deaths 2010 ICD10.
Panama Vital Registration - Deaths 2011 ICD10.
Panama Vital Registration - Deaths 2012 ICD10.
Panama Vital Registration - Deaths 2013 ICD10.
Panama Vital Registration - Deaths 2014 ICD10.
Panama Vital Registration - Deaths 2015 ICD10.
Panama Vital Registration - Deaths 2016 ICD10.
Panama Vital Registration - Deaths 2017 ICD10.
Paraguay Vital Registration - Deaths 1980 ICD9.
Paraguay Vital Registration - Deaths 1981 ICD9.
Paraguay Vital Registration - Deaths 1982 ICD9.
Paraguay Vital Registration - Deaths 1983 ICD9.

Paraguay Vital Registration - Deaths 1984 ICD9.
Paraguay Vital Registration - Deaths 1985 ICD9.
Paraguay Vital Registration - Deaths 1986 ICD9.
Paraguay Vital Registration - Deaths 1987 ICD9.
Paraguay Vital Registration - Deaths 1988 ICD9.
Paraguay Vital Registration - Deaths 1989 ICD9.
Paraguay Vital Registration - Deaths 1990 ICD9.
Paraguay Vital Registration - Deaths 1991 ICD9.
Paraguay Vital Registration - Deaths 1994 ICD9.
Paraguay Vital Registration - Deaths 1995 ICD9.
Paraguay Vital Registration - Deaths 1996 ICD10.
Paraguay Vital Registration - Deaths 1997 ICD10.
Paraguay Vital Registration - Deaths 1998 ICD10.
Paraguay Vital Registration - Deaths 1999 ICD10.
Paraguay Vital Registration - Deaths 2000 ICD10.
Paraguay Vital Registration - Deaths 2001 ICD10.
Paraguay Vital Registration - Deaths 2002 ICD10.
Paraguay Vital Registration - Deaths 2003 ICD10.
Peru Vital Registration - Deaths 1980 ICD9.
Peru Vital Registration - Deaths 1981 ICD9.
Peru Vital Registration - Deaths 1982 ICD9.
Peru Vital Registration - Deaths 1983 ICD9.
Peru Vital Registration - Deaths 1986 ICD9.
Peru Vital Registration - Deaths 1987 ICD9.
Peru Vital Registration - Deaths 1988 ICD9.
Peru Vital Registration - Deaths 1989 ICD9.
Peru Vital Registration - Deaths 1990 ICD9.
Peru Vital Registration - Deaths 1991 ICD9.
Peru Vital Registration - Deaths 1992 ICD9.
Peru Vital Registration - Deaths 1994 ICD9.
Peru Vital Registration - Deaths 1995 ICD9.
Peru Vital Registration - Deaths 1996 ICD9.
Peru Vital Registration - Deaths 1997 ICD9.
Peru Vital Registration - Deaths 1998 ICD9.
Peru Vital Registration - Deaths 1999 ICD10.
Peru Vital Registration - Deaths 2000 ICD10.
Peru Vital Registration - Deaths 2007 ICD10.
Peru Vital Registration - Deaths 2008 ICD10.
Peru Vital Registration - Deaths 2009 ICD10.
Peru Vital Registration - Deaths 2010 ICD10.
Peru Vital Registration - Deaths 2011 ICD10.
Peru Vital Registration - Deaths 2012 ICD10.
Peru Vital Registration - Deaths 2013 ICD10.
Peru Vital Registration - Deaths 2014 ICD10.
Peru Vital Registration - Deaths 2015 ICD10.
Peru Vital Registration - Deaths 2016 ICD10.
Peru Vital Registration - Deaths 2017 ICD10.
Philippines Statistics Authority. Philippines Vital Registration - Deaths 2013.
Philippines Statistics Authority. Philippines Vital Registration - Deaths 2014.
Philippines Statistics Authority. Philippines Vital Registration - Deaths 2015.
Philippines Statistics Authority. Philippines Vital Registration - Deaths 2016.
Philippines Statistics Authority. Philippines Vital Registration - Deaths 2017.
Philippines Statistics Authority. Philippines Vital Registration - Deaths 2018.
Porapakkham Y, Rao C, Pattaraarchachai J, Polprasert W, Vos T, Adair T, Lopez AD. Estimated causes of death in Thailand, 2005: implications for health policy. Popul Health Metr. 2010; 8:14.
Portugal Vital Registration - Deaths 1984 ICD9.
Portugal Vital Registration - Deaths 1985 ICD9.
Portugal Vital Registration - Deaths 1986 ICD9.
Portugal Vital Registration - Deaths 1987 ICD9.
Portugal Vital Registration - Deaths 1988 ICD9.
Portugal Vital Registration - Deaths 1989 ICD9.

Portugal Vital Registration - Deaths 1990 ICD9.
Portugal Vital Registration - Deaths 1991 ICD9.
Portugal Vital Registration - Deaths 1992 ICD9.
Portugal Vital Registration - Deaths 1993 ICD9.
Portugal Vital Registration - Deaths 1994 ICD9.
Portugal Vital Registration - Deaths 1995 ICD9.
Portugal Vital Registration - Deaths 1996 ICD9.
Portugal Vital Registration - Deaths 1997 ICD9.
Portugal Vital Registration - Deaths 1998 ICD9.
Portugal Vital Registration - Deaths 1999 ICD9.
Portugal Vital Registration - Deaths 2000 ICD9.
Portugal Vital Registration - Deaths 2001 ICD9.
Portugal Vital Registration - Deaths 2002 ICD10.
Portugal Vital Registration - Deaths 2003 ICD10.
Portugal Vital Registration - Deaths 2007 ICD10.
Portugal Vital Registration - Deaths 2008 ICD10.
Portugal Vital Registration - Deaths 2009 ICD10.
Portugal Vital Registration - Deaths 2010 ICD10.
Portugal Vital Registration - Deaths 2011 ICD10.
Portugal Vital Registration - Deaths 2012 ICD10.
Portugal Vital Registration - Deaths 2013 ICD10.
Portugal Vital Registration - Deaths 2014 ICD10.
Portugal Vital Registration - Deaths 2015 ICD10.
Portugal Vital Registration - Deaths 2016 ICD10.
Portugal Vital Registration - Deaths 2017 ICD10.
Portugal Vital Registration - Deaths 2018 ICD10.
Puerto Rico Vital Registration - Deaths 1980 ICD9.
Puerto Rico Vital Registration - Deaths 1981 ICD9.
Puerto Rico Vital Registration - Deaths 1982 ICD9.
Puerto Rico Vital Registration - Deaths 1983 ICD9.
Puerto Rico Vital Registration - Deaths 1984 ICD9.
Puerto Rico Vital Registration - Deaths 1985 ICD9.
Puerto Rico Vital Registration - Deaths 1986 ICD9.
Puerto Rico Vital Registration - Deaths 1987 ICD9.
Puerto Rico Vital Registration - Deaths 1988 ICD9.
Puerto Rico Vital Registration - Deaths 1989 ICD9.
Puerto Rico Vital Registration - Deaths 1990 ICD9.
Puerto Rico Vital Registration - Deaths 1991 ICD9.
Puerto Rico Vital Registration - Deaths 1992 ICD9.
Puerto Rico Vital Registration - Deaths 1993 ICD9.
Qatar Vital Registration - Deaths 1995 ICD10.
Qatar Vital Registration - Deaths 2001 ICD10.
Qatar Vital Registration - Deaths 2004 ICD10.
Qatar Vital Registration - Deaths 2005 ICD10.
Qatar Vital Registration - Deaths 2006 ICD10.
Qatar Vital Registration - Deaths 2007 ICD10.
Qatar Vital Registration - Deaths 2008 ICD10.
Qatar Vital Registration - Deaths 2009 ICD10.
Qatar Vital Registration - Deaths 2010 ICD10.
Qatar Vital Registration - Deaths 2011 ICD10.
Qatar Vital Registration - Deaths 2012 ICD10.
Qatar Vital Registration - Deaths 2013 ICD10.
Qatar Vital Registration - Deaths 2014 ICD10.
Qatar Vital Registration - Deaths 2015 ICD10.
Qatar Vital Registration - Deaths 2016 ICD10.
Qatar Vital Registration - Deaths 2017 ICD10.
Registrar General's Department (Zimbabwe), Zimbabwe National Statistics Agency. Zimbabwe Mortality Report 2007.
Republic of Moldova Vital Registration - Deaths 1981 ICD9.
Republic of Moldova Vital Registration - Deaths 1982 ICD9.
Republic of Moldova Vital Registration - Deaths 1985 ICD9.
Republic of Moldova Vital Registration - Deaths 1986 ICD9.

Republic of Moldova Vital Registration - Deaths 1987 ICD9.
Republic of Moldova Vital Registration - Deaths 1988 ICD9.
Republic of Moldova Vital Registration - Deaths 1989 ICD9.
Republic of Moldova Vital Registration - Deaths 1990 ICD9.
Republic of Moldova Vital Registration - Deaths 1991 ICD9.
Republic of Moldova Vital Registration - Deaths 1992 ICD9.
Republic of Moldova Vital Registration - Deaths 1993 ICD9.
Republic of Moldova Vital Registration - Deaths 1994 ICD9.
Republic of Moldova Vital Registration - Deaths 1995 ICD9.
Republic of Moldova Vital Registration - Deaths 1996 ICD10.
Republic of Moldova Vital Registration - Deaths 1997 ICD10.
Republic of Moldova Vital Registration - Deaths 1998 ICD10.
Republic of Moldova Vital Registration - Deaths 1999 ICD10.
Republic of Moldova Vital Registration - Deaths 2000 ICD10.
Republic of Moldova Vital Registration - Deaths 2001 ICD10.
Republic of Moldova Vital Registration - Deaths 2002 ICD10.
Republic of Moldova Vital Registration - Deaths 2003 ICD10.
Republic of Moldova Vital Registration - Deaths 2004 ICD10.
Republic of Moldova Vital Registration - Deaths 2005 ICD10.
Republic of Moldova Vital Registration - Deaths 2006 ICD10.
Republic of Moldova Vital Registration - Deaths 2007 ICD10.
Republic of Moldova Vital Registration - Deaths 2008 ICD10.
Republic of Moldova Vital Registration - Deaths 2009 ICD10.
Republic of Moldova Vital Registration - Deaths 2018 ICD10.
Romania Vital Registration - Deaths 1980 ICD9.
Romania Vital Registration - Deaths 1981 ICD9.
Romania Vital Registration - Deaths 1982 ICD9.
Romania Vital Registration - Deaths 1983 ICD9.
Romania Vital Registration - Deaths 1984 ICD9.
Romania Vital Registration - Deaths 1985 ICD9.
Romania Vital Registration - Deaths 1986 ICD9.
Romania Vital Registration - Deaths 1987 ICD9.
Romania Vital Registration - Deaths 1988 ICD9.
Romania Vital Registration - Deaths 1989 ICD9.
Romania Vital Registration - Deaths 1990 ICD9.
Romania Vital Registration - Deaths 1991 ICD9.
Romania Vital Registration - Deaths 1992 ICD9.
Romania Vital Registration - Deaths 1993 ICD9.
Romania Vital Registration - Deaths 1994 ICD9.
Romania Vital Registration - Deaths 1995 ICD9.
Romania Vital Registration - Deaths 1996 ICD9.
Romania Vital Registration - Deaths 1997 ICD9.
Romania Vital Registration - Deaths 1998 ICD9.
Romania Vital Registration - Deaths 1999 ICD10.
Romania Vital Registration - Deaths 2000 ICD10.
Romania Vital Registration - Deaths 2001 ICD10.
Romania Vital Registration - Deaths 2002 ICD10.
Romania Vital Registration - Deaths 2003 ICD10.
Romania Vital Registration - Deaths 2004 ICD10.
Romania Vital Registration - Deaths 2005 ICD10.
Romania Vital Registration - Deaths 2006 ICD10.
Romania Vital Registration - Deaths 2007 ICD10.
Romania Vital Registration - Deaths 2008 ICD10.
Romania Vital Registration - Deaths 2009 ICD10.
Romania Vital Registration - Deaths 2010 ICD10.
Romania Vital Registration - Deaths 2011 ICD10.
Romania Vital Registration - Deaths 2012 ICD10.
Romania Vital Registration - Deaths 2013 ICD10.
Romania Vital Registration - Deaths 2014 ICD10.
Romania Vital Registration - Deaths 2015 ICD10.
Romania Vital Registration - Deaths 2016 ICD10.

Romania Vital Registration - Deaths 2017 ICD10.
Romania Vital Registration - Deaths 2018 ICD10.
Saint Kitts and Nevis Vital Registration - Deaths 1980 ICD9.
Saint Kitts and Nevis Vital Registration - Deaths 1981 ICD9.
Saint Kitts and Nevis Vital Registration - Deaths 1982 ICD9.
Saint Kitts and Nevis Vital Registration - Deaths 1983 ICD9.
Saint Kitts and Nevis Vital Registration - Deaths 1984 ICD9.
Saint Kitts and Nevis Vital Registration - Deaths 1985 ICD9.
Saint Kitts and Nevis Vital Registration - Deaths 1986 ICD9.
Saint Kitts and Nevis Vital Registration - Deaths 1987 ICD9.
Saint Kitts and Nevis Vital Registration - Deaths 1988 ICD9.
Saint Kitts and Nevis Vital Registration - Deaths 1989 ICD9.
Saint Kitts and Nevis Vital Registration - Deaths 1990 ICD9.
Saint Kitts and Nevis Vital Registration - Deaths 1991 ICD9.
Saint Kitts and Nevis Vital Registration - Deaths 1992 ICD9.
Saint Kitts and Nevis Vital Registration - Deaths 1993 ICD9.
Saint Kitts and Nevis Vital Registration - Deaths 1994 ICD9.
Saint Kitts and Nevis Vital Registration - Deaths 1995 ICD9.
Saint Kitts and Nevis Vital Registration - Deaths 1998 ICD10.
Saint Kitts and Nevis Vital Registration - Deaths 1999 ICD10.
Saint Kitts and Nevis Vital Registration - Deaths 2000 ICD10.
Saint Kitts and Nevis Vital Registration - Deaths 2001 ICD10.
Saint Kitts and Nevis Vital Registration - Deaths 2002 ICD10.
Saint Kitts and Nevis Vital Registration - Deaths 2003 ICD10.
Saint Kitts and Nevis Vital Registration - Deaths 2004 ICD10.
Saint Kitts and Nevis Vital Registration - Deaths 2005 ICD10.
Saint Kitts and Nevis Vital Registration - Deaths 2006 ICD10.
Saint Kitts and Nevis Vital Registration - Deaths 2007 ICD10.
Saint Kitts and Nevis Vital Registration - Deaths 2008 ICD10.
Saint Kitts and Nevis Vital Registration - Deaths 2009 ICD10.
Saint Kitts and Nevis Vital Registration - Deaths 2010 ICD10.
Saint Kitts and Nevis Vital Registration - Deaths 2011 ICD10.
Saint Kitts and Nevis Vital Registration - Deaths 2012 ICD10.
Saint Kitts and Nevis Vital Registration - Deaths 2013 ICD10.
Saint Kitts and Nevis Vital Registration - Deaths 2014 ICD10.
Saint Kitts and Nevis Vital Registration - Deaths 2015 ICD10.
Saint Kitts and Nevis Vital Registration - Deaths 2016 ICD10.
Saint Lucia Vital Registration - Deaths 1980 ICD9.
Saint Lucia Vital Registration - Deaths 1981 ICD9.
Saint Lucia Vital Registration - Deaths 1983 ICD9.
Saint Lucia Vital Registration - Deaths 1986 ICD9.
Saint Lucia Vital Registration - Deaths 1987 ICD9.
Saint Lucia Vital Registration - Deaths 1988 ICD9.
Saint Lucia Vital Registration - Deaths 1989 ICD9.
Saint Lucia Vital Registration - Deaths 1990 ICD9.
Saint Lucia Vital Registration - Deaths 1991 ICD9.
Saint Lucia Vital Registration - Deaths 1992 ICD9.
Saint Lucia Vital Registration - Deaths 1993 ICD9.
Saint Lucia Vital Registration - Deaths 1994 ICD9.
Saint Lucia Vital Registration - Deaths 1995 ICD9.
Saint Lucia Vital Registration - Deaths 1996 ICD10.
Saint Lucia Vital Registration - Deaths 1997 ICD10.
Saint Lucia Vital Registration - Deaths 1998 ICD10.
Saint Lucia Vital Registration - Deaths 1999 ICD10.
Saint Lucia Vital Registration - Deaths 2000 ICD10.
Saint Lucia Vital Registration - Deaths 2001 ICD10.
Saint Lucia Vital Registration - Deaths 2002 ICD10.
Saint Lucia Vital Registration - Deaths 2003 ICD10.
Saint Lucia Vital Registration - Deaths 2004 ICD10.
Saint Lucia Vital Registration - Deaths 2005 ICD10.
Saint Lucia Vital Registration - Deaths 2006 ICD10.

Saint Lucia Vital Registration - Deaths 2008 ICD10.
Saint Lucia Vital Registration - Deaths 2009 ICD10.
Saint Lucia Vital Registration - Deaths 2010 ICD10.
Saint Lucia Vital Registration - Deaths 2011 ICD10.
Saint Lucia Vital Registration - Deaths 2012 ICD10.
Saint Lucia Vital Registration - Deaths 2013 ICD10.
Saint Lucia Vital Registration - Deaths 2014 ICD10.
Saint Lucia Vital Registration - Deaths 2015 ICD10.
Saint Vincent and the Grenadines Vital Registration - Deaths 1982 ICD9.
Saint Vincent and the Grenadines Vital Registration - Deaths 1983 ICD9.
Saint Vincent and the Grenadines Vital Registration - Deaths 1984 ICD9.
Saint Vincent and the Grenadines Vital Registration - Deaths 1985 ICD9.
Saint Vincent and the Grenadines Vital Registration - Deaths 1986 ICD9.
Saint Vincent and the Grenadines Vital Registration - Deaths 1987 ICD9.
Saint Vincent and the Grenadines Vital Registration - Deaths 1990 ICD9.
Saint Vincent and the Grenadines Vital Registration - Deaths 1995 ICD9.
Saint Vincent and the Grenadines Vital Registration - Deaths 1996 ICD9.
Saint Vincent and the Grenadines Vital Registration - Deaths 1997 ICD9.
Saint Vincent and the Grenadines Vital Registration - Deaths 1998 ICD9.
Saint Vincent and the Grenadines Vital Registration - Deaths 1999 ICD9.
Saint Vincent and the Grenadines Vital Registration - Deaths 2000 ICD10.
Saint Vincent and the Grenadines Vital Registration - Deaths 2001 ICD10.
Saint Vincent and the Grenadines Vital Registration - Deaths 2002 ICD10.
Saint Vincent and the Grenadines Vital Registration - Deaths 2003 ICD10.
Saint Vincent and the Grenadines Vital Registration - Deaths 2004 ICD10.
Saint Vincent and the Grenadines Vital Registration - Deaths 2005 ICD10.
Saint Vincent and the Grenadines Vital Registration - Deaths 2006 ICD10.
Saint Vincent and the Grenadines Vital Registration - Deaths 2007 ICD10.
Saint Vincent and the Grenadines Vital Registration - Deaths 2008 ICD10.
Saint Vincent and the Grenadines Vital Registration - Deaths 2009 ICD10.
Saint Vincent and the Grenadines Vital Registration - Deaths 2010 ICD10.
Saint Vincent and the Grenadines Vital Registration - Deaths 2011 ICD10.
Saint Vincent and the Grenadines Vital Registration - Deaths 2012 ICD10.
Saint Vincent and the Grenadines Vital Registration - Deaths 2013 ICD10.
Saint Vincent and the Grenadines Vital Registration - Deaths 2014 ICD10.
Saint Vincent and the Grenadines Vital Registration - Deaths 2015 ICD10.
Saint Vincent and the Grenadines Vital Registration - Deaths 2016 ICD10.
Saint Vincent and the Grenadines Vital Registration - Deaths 2017 ICD10.
San Marino Vital Registration - Deaths 1995 ICD9.
San Marino Vital Registration - Deaths 1996 ICD9.
San Marino Vital Registration - Deaths 1997 ICD9.
San Marino Vital Registration - Deaths 1998 ICD9.
San Marino Vital Registration - Deaths 1999 ICD9.
San Marino Vital Registration - Deaths 2000 ICD9.
San Marino Vital Registration - Deaths 2002 ICD9.
San Marino Vital Registration - Deaths 2005 ICD9.
San Marino Vital Registration - Deaths 2011 ICD10.
San Marino Vital Registration - Deaths 2012 ICD10.
San Marino Vital Registration - Deaths 2013 ICD10.
San Marino Vital Registration - Deaths 2014 ICD10.
San Marino Vital Registration - Deaths 2015 ICD10.
Sao Tome and Principe Vital Registration - Deaths 1985 ICD9.
Serbia and Montenegro - Montenegro Vital Registration - Deaths 2003 ICD10.
Serbia and Montenegro - Montenegro Vital Registration - Deaths 2004 ICD10.
Serbia and Montenegro - Serbia Vital Registration - Deaths 2003 ICD10.
Serbia and Montenegro - Serbia Vital Registration - Deaths 2004 ICD10.
Serbia and Montenegro - Serbia Vital Registration - Deaths 2005 ICD10.
Serbia and Montenegro - Serbia Vital Registration - Deaths 2006 ICD10.
Serbia Vital Registration - Deaths 2007 ICD10.
Serbia Vital Registration - Deaths 2008 ICD10.
Serbia Vital Registration - Deaths 2009 ICD10.

Serbia Vital Registration - Deaths 2010 ICD10.
Serbia Vital Registration - Deaths 2011 ICD10.
Serbia Vital Registration - Deaths 2012 ICD10.
Serbia Vital Registration - Deaths 2013 ICD10.
Serbia Vital Registration - Deaths 2014 ICD10.
Serbia Vital Registration - Deaths 2015 ICD10.
Serbia Vital Registration - Deaths 2016 ICD10.
Serbia Vital Registration - Deaths 2017 ICD10.
Seychelles Vital Registration - Deaths 1981 ICD9.
Seychelles Vital Registration - Deaths 1982 ICD9.
Seychelles Vital Registration - Deaths 1985 ICD9.
Seychelles Vital Registration - Deaths 1986 ICD9.
Seychelles Vital Registration - Deaths 1987 ICD9.
Seychelles Vital Registration - Deaths 2001 ICD10.
Seychelles Vital Registration - Deaths 2002 ICD10.
Seychelles Vital Registration - Deaths 2003 ICD10.
Seychelles Vital Registration - Deaths 2004 ICD10.
Seychelles Vital Registration - Deaths 2005 ICD10.
Seychelles Vital Registration - Deaths 2006 ICD10.
Seychelles Vital Registration - Deaths 2007 ICD10.
Seychelles Vital Registration - Deaths 2008 ICD10.
Seychelles Vital Registration - Deaths 2009 ICD10.
Seychelles Vital Registration - Deaths 2010 ICD10.
Seychelles Vital Registration - Deaths 2011 ICD10.
Seychelles Vital Registration - Deaths 2012 ICD10.
Seychelles Vital Registration - Deaths 2013 ICD10.
Seychelles Vital Registration - Deaths 2014 ICD10.
Seychelles Vital Registration - Deaths 2015 ICD10.
Slovakia Vital Registration - Deaths 1992 ICD9.
Slovakia Vital Registration - Deaths 1993 ICD9.
Slovakia Vital Registration - Deaths 1994 ICD10.
Slovakia Vital Registration - Deaths 1995 ICD10.
Slovakia Vital Registration - Deaths 1996 ICD10.
Slovakia Vital Registration - Deaths 1997 ICD10.
Slovakia Vital Registration - Deaths 1998 ICD10.
Slovakia Vital Registration - Deaths 1999 ICD10.
Slovakia Vital Registration - Deaths 2000 ICD10.
Slovakia Vital Registration - Deaths 2001 ICD10.
Slovakia Vital Registration - Deaths 2002 ICD10.
Slovakia Vital Registration - Deaths 2003 ICD10.
Slovakia Vital Registration - Deaths 2004 ICD10.
Slovakia Vital Registration - Deaths 2005 ICD10.
Slovakia Vital Registration - Deaths 2006 ICD10.
Slovakia Vital Registration - Deaths 2007 ICD10.
Slovakia Vital Registration - Deaths 2008 ICD10.
Slovakia Vital Registration - Deaths 2009 ICD10.
Slovakia Vital Registration - Deaths 2010 ICD10.
Slovakia Vital Registration - Deaths 2012 ICD10.
Slovakia Vital Registration - Deaths 2013 ICD10.
Slovakia Vital Registration - Deaths 2014 ICD10.
Slovenia Vital Registration - Deaths 1989 ICD9.
Slovenia Vital Registration - Deaths 1991 ICD9.
Slovenia Vital Registration - Deaths 1992 ICD9.
Slovenia Vital Registration - Deaths 1993 ICD9.
Slovenia Vital Registration - Deaths 1994 ICD9.
Slovenia Vital Registration - Deaths 1995 ICD9.
Slovenia Vital Registration - Deaths 1996 ICD9.
Slovenia Vital Registration - Deaths 1997 ICD10.
Slovenia Vital Registration - Deaths 1998 ICD10.
Slovenia Vital Registration - Deaths 1999 ICD10.
Slovenia Vital Registration - Deaths 2000 ICD10.

Slovenia Vital Registration - Deaths 2001 ICD10.
Slovenia Vital Registration - Deaths 2002 ICD10.
Slovenia Vital Registration - Deaths 2003 ICD10.
Slovenia Vital Registration - Deaths 2004 ICD10.
Slovenia Vital Registration - Deaths 2005 ICD10.
Slovenia Vital Registration - Deaths 2006 ICD10.
Slovenia Vital Registration - Deaths 2007 ICD10.
Slovenia Vital Registration - Deaths 2008 ICD10.
Slovenia Vital Registration - Deaths 2009 ICD10.
Slovenia Vital Registration - Deaths 2010 ICD10.
Slovenia Vital Registration - Deaths 2011 ICD10.
Slovenia Vital Registration - Deaths 2012 ICD10.
Slovenia Vital Registration - Deaths 2013 ICD10.
Slovenia Vital Registration - Deaths 2014 ICD10.
Slovenia Vital Registration - Deaths 2015 ICD10.
Slovenia Vital Registration - Deaths 2016 ICD10.
Slovenia Vital Registration - Deaths 2017 ICD10.
Slovenia Vital Registration - Deaths 2018 ICD10.
South Korea Vital Registration - Deaths 1985 ICD9.
South Korea Vital Registration - Deaths 1986 ICD9.
South Korea Vital Registration - Deaths 1987 ICD9.
South Korea Vital Registration - Deaths 1988 ICD9.
South Korea Vital Registration - Deaths 1989 ICD9.
South Korea Vital Registration - Deaths 1990 ICD9.
South Korea Vital Registration - Deaths 1991 ICD9.
South Korea Vital Registration - Deaths 1992 ICD9.
South Korea Vital Registration - Deaths 1993 ICD9.
South Korea Vital Registration - Deaths 1994 ICD9.
South Korea Vital Registration - Deaths 1995 ICD10.
South Korea Vital Registration - Deaths 1996 ICD10.
South Korea Vital Registration - Deaths 1997 ICD10.
South Korea Vital Registration - Deaths 1998 ICD10.
South Korea Vital Registration - Deaths 1999 ICD10.
South Korea Vital Registration - Deaths 2000 ICD10.
South Korea Vital Registration - Deaths 2001 ICD10.
South Korea Vital Registration - Deaths 2002 ICD10.
South Korea Vital Registration - Deaths 2003 ICD10.
South Korea Vital Registration - Deaths 2004 ICD10.
South Korea Vital Registration - Deaths 2005 ICD10.
South Korea Vital Registration - Deaths 2006 ICD10.
South Korea Vital Registration - Deaths 2007 ICD10.
South Korea Vital Registration - Deaths 2008 ICD10.
South Korea Vital Registration - Deaths 2009 ICD10.
South Korea Vital Registration - Deaths 2010 ICD10.
South Korea Vital Registration - Deaths 2011 ICD10.
South Korea Vital Registration - Deaths 2012 ICD10.
South Korea Vital Registration - Deaths 2013 ICD10.
South Korea Vital Registration - Deaths 2014 ICD10.
South Korea Vital Registration - Deaths 2015 ICD10.
South Korea Vital Registration - Deaths 2016 ICD10.
South Korea Vital Registration - Deaths 2017 ICD10.
South Korea Vital Registration - Deaths 2018 ICD10.
Spain Vital Registration - Deaths 1980 ICD9.
Spain Vital Registration - Deaths 1981 ICD9.
Spain Vital Registration - Deaths 1982 ICD9.
Spain Vital Registration - Deaths 1983 ICD9.
Spain Vital Registration - Deaths 1984 ICD9.
Spain Vital Registration - Deaths 1985 ICD9.
Spain Vital Registration - Deaths 1986 ICD9.
Spain Vital Registration - Deaths 1987 ICD9.
Spain Vital Registration - Deaths 1988 ICD9.

Spain Vital Registration - Deaths 1989 ICD9.
Spain Vital Registration - Deaths 1990 ICD9.
Spain Vital Registration - Deaths 1991 ICD9.
Spain Vital Registration - Deaths 1992 ICD9.
Spain Vital Registration - Deaths 1993 ICD9.
Spain Vital Registration - Deaths 1994 ICD9.
Spain Vital Registration - Deaths 1995 ICD9.
Spain Vital Registration - Deaths 1996 ICD9.
Spain Vital Registration - Deaths 1997 ICD9.
Spain Vital Registration - Deaths 1998 ICD9.
Spain Vital Registration - Deaths 1999 ICD10.
Spain Vital Registration - Deaths 2000 ICD10.
Spain Vital Registration - Deaths 2001 ICD10.
Spain Vital Registration - Deaths 2002 ICD10.
Spain Vital Registration - Deaths 2003 ICD10.
Spain Vital Registration - Deaths 2004 ICD10.
Spain Vital Registration - Deaths 2005 ICD10.
Spain Vital Registration - Deaths 2006 ICD10.
Spain Vital Registration - Deaths 2007 ICD10.
Spain Vital Registration - Deaths 2008 ICD10.
Spain Vital Registration - Deaths 2009 ICD10.
Spain Vital Registration - Deaths 2010 ICD10.
Spain Vital Registration - Deaths 2011 ICD10.
Spain Vital Registration - Deaths 2012 ICD10.
Spain Vital Registration - Deaths 2013 ICD10.
Spain Vital Registration - Deaths 2014 ICD10.
Spain Vital Registration - Deaths 2015 ICD10.
Spain Vital Registration - Deaths 2016 ICD10.
Spain Vital Registration - Deaths 2017 ICD10.
Sri Lanka Vital Registration - Deaths 1997 ICD10.
Sri Lanka Vital Registration - Deaths 1998 ICD10.
Sri Lanka Vital Registration - Deaths 1999 ICD10.
Sri Lanka Vital Registration - Deaths 2000 ICD10.
Sri Lanka Vital Registration - Deaths 2001 ICD10.
Sri Lanka Vital Registration - Deaths 2002 ICD10.
Sri Lanka Vital Registration - Deaths 2003 ICD10.
Sri Lanka Vital Registration - Deaths 2006 ICD10.
State Statistics Service (Ukraine). Ukraine Vital Registration - Deaths 2013.
State Statistics Service (Ukraine). Ukraine Vital Registration - Deaths 2015.
State Statistics Service (Ukraine). Ukraine Vital Registration - Deaths 2016.
Statistics Belgium. Belgium Vital Registration - Causes of Deaths 2017.
Statistics Belgium. Belgium Vital Registration - Causes of Deaths 2018.
Statistics Portugal. Portugal Vital Registration - Deaths 1980.
Statistics Portugal. Portugal Vital Registration - Deaths 1981.
Statistics Portugal. Portugal Vital Registration - Deaths 1982.
Statistics Portugal. Portugal Vital Registration - Deaths 1983.
Statistics Portugal. Portugal Vital Registration - Deaths 2004.
Suriname Vital Registration - Deaths 1980 ICD9.
Suriname Vital Registration - Deaths 1981 ICD9.
Suriname Vital Registration - Deaths 1982 ICD9.
Suriname Vital Registration - Deaths 1984 ICD9.
Suriname Vital Registration - Deaths 1985 ICD9.
Suriname Vital Registration - Deaths 1986 ICD9.
Suriname Vital Registration - Deaths 1987 ICD9.
Suriname Vital Registration - Deaths 1988 ICD9.
Suriname Vital Registration - Deaths 1989 ICD9.
Suriname Vital Registration - Deaths 1990 ICD9.
Suriname Vital Registration - Deaths 1991 ICD9.
Suriname Vital Registration - Deaths 1992 ICD9.
Suriname Vital Registration - Deaths 1995 ICD10.
Suriname Vital Registration - Deaths 1996 ICD10.

Suriname Vital Registration - Deaths 1997 ICD10.
Suriname Vital Registration - Deaths 1998 ICD10.
Suriname Vital Registration - Deaths 1999 ICD10.
Suriname Vital Registration - Deaths 2000 ICD10.
Suriname Vital Registration - Deaths 2001 ICD10.
Suriname Vital Registration - Deaths 2002 ICD10.
Suriname Vital Registration - Deaths 2003 ICD10.
Suriname Vital Registration - Deaths 2004 ICD10.
Suriname Vital Registration - Deaths 2005 ICD10.
Suriname Vital Registration - Deaths 2006 ICD10.
Suriname Vital Registration - Deaths 2008 ICD10.
Suriname Vital Registration - Deaths 2009 ICD10.
Suriname Vital Registration - Deaths 2010 ICD10.
Suriname Vital Registration - Deaths 2011 ICD10.
Suriname Vital Registration - Deaths 2012 ICD10.
Suriname Vital Registration - Deaths 2013 ICD10.
Suriname Vital Registration - Deaths 2014 ICD10.
Switzerland Vital Registration - Deaths 1995 ICD10.
Switzerland Vital Registration - Deaths 1996 ICD10.
Switzerland Vital Registration - Deaths 1997 ICD10.
Switzerland Vital Registration - Deaths 1998 ICD10.
Switzerland Vital Registration - Deaths 1999 ICD10.
Switzerland Vital Registration - Deaths 2000 ICD10.
Switzerland Vital Registration - Deaths 2001 ICD10.
Switzerland Vital Registration - Deaths 2002 ICD10.
Switzerland Vital Registration - Deaths 2003 ICD10.
Switzerland Vital Registration - Deaths 2004 ICD10.
Switzerland Vital Registration - Deaths 2005 ICD10.
Switzerland Vital Registration - Deaths 2006 ICD10.
Switzerland Vital Registration - Deaths 2007 ICD10.
Switzerland Vital Registration - Deaths 2008 ICD10.
Switzerland Vital Registration - Deaths 2009 ICD10.
Switzerland Vital Registration - Deaths 2010 ICD10.
Switzerland Vital Registration - Deaths 2011 ICD10.
Switzerland Vital Registration - Deaths 2012 ICD10.
Switzerland Vital Registration - Deaths 2013 ICD10.
Switzerland Vital Registration - Deaths 2014 ICD10.
Switzerland Vital Registration - Deaths 2015 ICD10.
Switzerland Vital Registration - Deaths 2016 ICD10.
Switzerland Vital Registration - Deaths 2017 ICD10.
Syria Vital Registration - Deaths 2010 ICD10.
Syrian Arab Republic Vital Registration - Deaths 1999 ICD10.
Syrian Arab Republic Vital Registration - Deaths 2000 ICD10.
Syrian Arab Republic Vital Registration - Deaths 2001 ICD10.
Syrian Arab Republic Vital Registration - Deaths 2002 ICD10.
Syrian Arab Republic Vital Registration - Deaths 2003 ICD10.
Syrian Arab Republic Vital Registration - Deaths 2004 ICD10.
Syrian Arab Republic Vital Registration - Deaths 2005 ICD10.
Syrian Arab Republic Vital Registration - Deaths 2006 ICD10.
Syrian Arab Republic Vital Registration - Deaths 2007 ICD10.
Syrian Arab Republic Vital Registration - Deaths 2008 ICD10.
Syrian Arab Republic Vital Registration - Deaths 2009 ICD10.
Tajikistan Vital Registration - Deaths 1981 ICD9.
Tajikistan Vital Registration - Deaths 1982 ICD9.
Tajikistan Vital Registration - Deaths 1985 ICD9.
Tajikistan Vital Registration - Deaths 1986 ICD9.
Tajikistan Vital Registration - Deaths 1987 ICD9.
Tajikistan Vital Registration - Deaths 1988 ICD9.
Tajikistan Vital Registration - Deaths 1989 ICD9.
Tajikistan Vital Registration - Deaths 1990 ICD9.
Tajikistan Vital Registration - Deaths 1991 ICD9.

Tajikistan Vital Registration - Deaths 1992 ICD9.
Tajikistan Vital Registration - Deaths 1993 ICD9.
Tajikistan Vital Registration - Deaths 1994 ICD9.
Tajikistan Vital Registration - Deaths 1995 ICD9.
Tajikistan Vital Registration - Deaths 1996 ICD9.
Tajikistan Vital Registration - Deaths 1997 ICD9.
Tajikistan Vital Registration - Deaths 1998 ICD9.
Tajikistan Vital Registration - Deaths 1999 ICD9.
Tajikistan Vital Registration - Deaths 2000 ICD9.
Tajikistan Vital Registration - Deaths 2001 ICD9.
Tajikistan Vital Registration - Deaths 2002 ICD9.
Tajikistan Vital Registration - Deaths 2003 ICD9.
Tajikistan Vital Registration - Deaths 2004 ICD9.
Tajikistan Vital Registration - Deaths 2005 ICD9.
Tajikistan Vital Registration - Deaths 2016 ICD10.
Thailand Vital Registration - Deaths 2011 ICD10.
Thailand Vital Registration - Deaths 2012 ICD10.
Thailand Vital Registration - Deaths 2013 ICD10.
Thailand Vital Registration - Deaths 2014 ICD10.
Thailand Vital Registration - Deaths 2015 ICD10.
Thailand Vital Registration - Deaths 2016 ICD10.
Thailand Vital Registration - Deaths 2017 ICD10.
Thailand Vital Registration - Deaths 2018 ICD10.
Trinidad and Tobago Vital Registration - Deaths 1980 ICD9.
Trinidad and Tobago Vital Registration - Deaths 1981 ICD9.
Trinidad and Tobago Vital Registration - Deaths 1982 ICD9.
Trinidad and Tobago Vital Registration - Deaths 1983 ICD9.
Trinidad and Tobago Vital Registration - Deaths 1984 ICD9.
Trinidad and Tobago Vital Registration - Deaths 1985 ICD9.
Trinidad and Tobago Vital Registration - Deaths 1986 ICD9.
Trinidad and Tobago Vital Registration - Deaths 1987 ICD9.
Trinidad and Tobago Vital Registration - Deaths 1988 ICD9.
Trinidad and Tobago Vital Registration - Deaths 1989 ICD9.
Trinidad and Tobago Vital Registration - Deaths 1990 ICD9.
Trinidad and Tobago Vital Registration - Deaths 1991 ICD9.
Trinidad and Tobago Vital Registration - Deaths 1992 ICD9.
Trinidad and Tobago Vital Registration - Deaths 1993 ICD9.
Trinidad and Tobago Vital Registration - Deaths 1994 ICD9.
Trinidad and Tobago Vital Registration - Deaths 1995 ICD9.
Trinidad and Tobago Vital Registration - Deaths 1996 ICD9.
Trinidad and Tobago Vital Registration - Deaths 1997 ICD9.
Trinidad and Tobago Vital Registration - Deaths 1998 ICD9.
Trinidad and Tobago Vital Registration - Deaths 1999 ICD10.
Trinidad and Tobago Vital Registration - Deaths 2000 ICD10.
Trinidad and Tobago Vital Registration - Deaths 2001 ICD10.
Trinidad and Tobago Vital Registration - Deaths 2002 ICD10.
Trinidad and Tobago Vital Registration - Deaths 2003 ICD10.
Trinidad and Tobago Vital Registration - Deaths 2004 ICD10.
Trinidad and Tobago Vital Registration - Deaths 2005 ICD10.
Trinidad and Tobago Vital Registration - Deaths 2006 ICD10.
Trinidad and Tobago Vital Registration - Deaths 2007 ICD10.
Trinidad and Tobago Vital Registration - Deaths 2008 ICD10.
Trinidad and Tobago Vital Registration - Deaths 2009 ICD10.
Trinidad and Tobago Vital Registration - Deaths 2010 ICD10.
Trinidad and Tobago Vital Registration - Deaths 2011 ICD10.
Trinidad and Tobago Vital Registration - Deaths 2012 ICD10.
Turkey Vital Registration - Deaths 2009 ICD10.
Turkey Vital Registration - Deaths 2010 ICD10.
Turkey Vital Registration - Deaths 2011 ICD10.
Turkey Vital Registration - Deaths 2012 ICD10.
Turkey Vital Registration - Deaths 2013 ICD10.

Turkey Vital Registration - Deaths 2014 ICD10.
Turkey Vital Registration - Deaths 2015 ICD10.
Turkey Vital Registration - Deaths 2016 ICD10.
Turkmenistan Vital Registration - Deaths 1981 ICD9.
Turkmenistan Vital Registration - Deaths 1982 ICD9.
Turkmenistan Vital Registration - Deaths 1985 ICD9.
Turkmenistan Vital Registration - Deaths 1986 ICD9.
Turkmenistan Vital Registration - Deaths 1987 ICD9.
Turkmenistan Vital Registration - Deaths 1988 ICD9.
Turkmenistan Vital Registration - Deaths 1989 ICD9.
Turkmenistan Vital Registration - Deaths 1990 ICD9.
Turkmenistan Vital Registration - Deaths 1991 ICD9.
Turkmenistan Vital Registration - Deaths 1992 ICD9.
Turkmenistan Vital Registration - Deaths 1993 ICD9.
Turkmenistan Vital Registration - Deaths 1994 ICD9.
Turkmenistan Vital Registration - Deaths 1995 ICD9.
Turkmenistan Vital Registration - Deaths 1996 ICD9.
Turkmenistan Vital Registration - Deaths 1997 ICD9.
Turkmenistan Vital Registration - Deaths 1998 ICD9.
Turkmenistan Vital Registration - Deaths 1999 ICD10.
Turkmenistan Vital Registration - Deaths 2000 ICD10.
Turkmenistan Vital Registration - Deaths 2001 ICD10.
Turkmenistan Vital Registration - Deaths 2002 ICD10.
Turkmenistan Vital Registration - Deaths 2003 ICD10.
Turkmenistan Vital Registration - Deaths 2004 ICD10.
Turkmenistan Vital Registration - Deaths 2005 ICD10.
Turkmenistan Vital Registration - Deaths 2006 ICD10.
Turkmenistan Vital Registration - Deaths 2007 ICD10.
Turkmenistan Vital Registration - Deaths 2008 ICD10.
Turkmenistan Vital Registration - Deaths 2009 ICD10.
Turkmenistan Vital Registration - Deaths 2010 ICD10.
Turkmenistan Vital Registration - Deaths 2011 ICD10.
Turkmenistan Vital Registration - Deaths 2012 ICD10.
Turkmenistan Vital Registration - Deaths 2013 ICD10.
Turkmenistan Vital Registration - Deaths 2014 ICD10.
Turkmenistan Vital Registration - Deaths 2015 ICD10.
U.S. Department of Defense. United States Military Deaths 1980-2014. Washington, D.C., United States of America: U.S. Department of Defense, 2016.
U.S. Department of Defense. United States Military Deaths 1980-2014. Washington, D.C., United States of America: U.S. Department of Defense, 2016.
U.S. Department of Defense. United States Military Deaths 1980-2014. Washington, D.C., United States of America: U.S. Department of Defense, 2016.
U.S. Department of Defense. United States Military Deaths 1980-2014. Washington, D.C., United States of America: U.S. Department of Defense, 2016.
U.S. Department of Defense. United States Military Deaths 1980-2014. Washington, D.C., United States of America: U.S. Department of Defense, 2016.
U.S. Department of Defense. United States Military Deaths 1980-2014. Washington, D.C., United States of America: U.S. Department of Defense, 2016.
U.S. Department of Defense. United States Military Deaths 1980-2014. Washington, D.C., United States of America: U.S. Department of Defense, 2016.
U.S. Department of Defense. United States Military Deaths 1980-2014. Washington, D.C., United States of America: U.S. Department of Defense, 2016.
U.S. Department of Defense. United States Military Deaths 1980-2014. Washington, D.C., United States of America: U.S. Department of Defense, 2016.
U.S. Department of Defense. United States Military Deaths 1980-2014. Washington, D.C., United States of America: U.S. Department of Defense, 2016.
U.S. Department of Defense. United States Military Deaths 1980-2014. Washington, D.C., United States of America: U.S. Department of Defense, 2016.
U.S. Department of Defense. United States Military Deaths 1980-2014. Washington, D.C., United States of America: U.S. Department of Defense, 2016.
U.S. Department of Defense. United States Military Deaths 1980-2014. Washington, D.C., United States of America: U.S. Department of Defense, 2016.
U.S. Department of Defense. United States Military Deaths 1980-2014. Washington, D.C., United States of America: U.S. Department of Defense, 2016.

U.S. Department of Defense. United States Military Deaths 1980-2014. Washington, D.C., United States of America: U.S. Department of Defense, 2016.
U.S. Department of Defense. United States Military Deaths 1980-2014. Washington, D.C., United States of America: U.S. Department of Defense, 2016.
U.S. Department of Defense. United States Military Deaths 1980-2014. Washington, D.C., United States of America: U.S. Department of Defense, 2016.
U.S. Department of Defense. United States Military Deaths 1980-2014. Washington, D.C., United States of America: U.S. Department of Defense, 2016.
U.S. Department of Defense. United States Military Deaths 1980-2014. Washington, D.C., United States of America: U.S. Department of Defense, 2016.
U.S. Department of Defense. United States Military Deaths 1980-2014. Washington, D.C., United States of America: U.S. Department of Defense, 2016.
U.S. Department of Defense. United States Military Deaths 1980-2014. Washington, D.C., United States of America: U.S. Department of Defense, 2016.
U.S. Department of Defense. United States Military Deaths 1980-2014. Washington, D.C., United States of America: U.S. Department of Defense, 2016.
U.S. Department of Defense. United States Military Deaths 1980-2014. Washington, D.C., United States of America: U.S. Department of Defense, 2016.
U.S. Department of Defense. United States Military Deaths 1980-2014. Washington, D.C., United States of America: U.S. Department of Defense, 2016.
U.S. Department of Defense. United States Military Deaths 1980-2014. Washington, D.C., United States of America: U.S. Department of Defense, 2016.
Ukraine Vital Registration - Deaths 1981 ICD9.
Ukraine Vital Registration - Deaths 1982 ICD9.
Ukraine Vital Registration - Deaths 1985 ICD9.
Ukraine Vital Registration - Deaths 1986 ICD9.
Ukraine Vital Registration - Deaths 1987 ICD9.
Ukraine Vital Registration - Deaths 1988 ICD9.
Ukraine Vital Registration - Deaths 1989 ICD9.
Ukraine Vital Registration - Deaths 1990 ICD9.
Ukraine Vital Registration - Deaths 1991 ICD9.
Ukraine Vital Registration - Deaths 1992 ICD9.
Ukraine Vital Registration - Deaths 1993 ICD9.
Ukraine Vital Registration - Deaths 1994 ICD9.
Ukraine Vital Registration - Deaths 1995 ICD9.
Ukraine Vital Registration - Deaths 1996 ICD9.
Ukraine Vital Registration - Deaths 1997 ICD9.
Ukraine Vital Registration - Deaths 1998 ICD9.
Ukraine Vital Registration - Deaths 1999 ICD9.
Ukraine Vital Registration - Deaths 2000 ICD9.
Ukraine Vital Registration - Deaths 2001 ICD9.
Ukraine Vital Registration - Deaths 2002 ICD9.
Ukraine Vital Registration - Deaths 2003 ICD9.
Ukraine Vital Registration - Deaths 2004 ICD9.
Ukraine Vital Registration - Deaths 2005 ICD10.
Ukraine Vital Registration - Deaths 2006 ICD10.
Ukraine Vital Registration - Deaths 2007 ICD10.
Ukraine Vital Registration - Deaths 2008 ICD10.
Ukraine Vital Registration - Deaths 2009 ICD10.
Ukraine Vital Registration - Deaths 2010 ICD10.
Ukraine Vital Registration - Deaths 2011 ICD10.
Ukraine Vital Registration - Deaths 2012 ICD10.
Ukraine Vital Registration - Deaths 2014 ICD10.
United Kingdom - Northern Ireland Vital Registration - Deaths 1980 ICD9.
United Kingdom - Northern Ireland Vital Registration - Deaths 1981 ICD9.
United Kingdom - Northern Ireland Vital Registration - Deaths 1982 ICD9.
United Kingdom - Northern Ireland Vital Registration - Deaths 1983 ICD9.
United Kingdom - Northern Ireland Vital Registration - Deaths 1984 ICD9.
United Kingdom - Northern Ireland Vital Registration - Deaths 1985 ICD9.
United Kingdom - Northern Ireland Vital Registration - Deaths 1986 ICD9.
United Kingdom - Northern Ireland Vital Registration - Deaths 1987 ICD9.
United Kingdom - Northern Ireland Vital Registration - Deaths 1988 ICD9.
United Kingdom - Northern Ireland Vital Registration - Deaths 1989 ICD9.

United Kingdom - Northern Ireland Vital Registration - Deaths 1990 ICD9.
United Kingdom - Northern Ireland Vital Registration - Deaths 1991 ICD9.
United Kingdom - Northern Ireland Vital Registration - Deaths 1992 ICD9.
United Kingdom - Northern Ireland Vital Registration - Deaths 1993 ICD9.
United Kingdom - Northern Ireland Vital Registration - Deaths 1994 ICD9.
United Kingdom - Northern Ireland Vital Registration - Deaths 1995 ICD9.
United Kingdom - Northern Ireland Vital Registration - Deaths 1996 ICD9.
United Kingdom - Northern Ireland Vital Registration - Deaths 1997 ICD9.
United Kingdom - Northern Ireland Vital Registration - Deaths 1998 ICD9.
United Kingdom - Northern Ireland Vital Registration - Deaths 1999 ICD9.
United Kingdom - Northern Ireland Vital Registration - Deaths 2000 ICD9.
United Kingdom - Northern Ireland Vital Registration - Deaths 2001 ICD10.
United Kingdom - Northern Ireland Vital Registration - Deaths 2002 ICD10.
United Kingdom - Northern Ireland Vital Registration - Deaths 2003 ICD10.
United Kingdom - Northern Ireland Vital Registration - Deaths 2004 ICD10.
United Kingdom - Northern Ireland Vital Registration - Deaths 2005 ICD10.
United Kingdom - Northern Ireland Vital Registration - Deaths 2006 ICD10.
United Kingdom - Northern Ireland Vital Registration - Deaths 2007 ICD10.
United Kingdom - Northern Ireland Vital Registration - Deaths 2008 ICD10.
United Kingdom - Northern Ireland Vital Registration - Deaths 2009 ICD10.
United Kingdom - Northern Ireland Vital Registration - Deaths 2010 ICD10.
United Kingdom - Northern Ireland Vital Registration - Deaths 2011 ICD10.
United Kingdom - Northern Ireland Vital Registration - Deaths 2012 ICD10.
United Kingdom - Northern Ireland Vital Registration - Deaths 2013 ICD10.
United Kingdom - Northern Ireland Vital Registration - Deaths 2014 ICD10.
United Kingdom - Northern Ireland Vital Registration - Deaths 2015 ICD10.
United Kingdom - Northern Ireland Vital Registration - Deaths 2016 ICD10.
United Kingdom - Scotland Vital Registration - Deaths 1980 ICD9.
United Kingdom - Scotland Vital Registration - Deaths 1981 ICD9.
United Kingdom - Scotland Vital Registration - Deaths 1982 ICD9.
United Kingdom - Scotland Vital Registration - Deaths 1983 ICD9.
United Kingdom - Scotland Vital Registration - Deaths 1984 ICD9.
United Kingdom - Scotland Vital Registration - Deaths 1985 ICD9.
United Kingdom - Scotland Vital Registration - Deaths 1986 ICD9.
United Kingdom - Scotland Vital Registration - Deaths 1987 ICD9.
United Kingdom - Scotland Vital Registration - Deaths 1988 ICD9.
United Kingdom - Scotland Vital Registration - Deaths 1989 ICD9.
United Kingdom - Scotland Vital Registration - Deaths 1990 ICD9.
United Kingdom - Scotland Vital Registration - Deaths 1991 ICD9.
United Kingdom - Scotland Vital Registration - Deaths 1992 ICD9.
United Kingdom - Scotland Vital Registration - Deaths 1993 ICD9.
United Kingdom - Scotland Vital Registration - Deaths 1994 ICD9.
United Kingdom - Scotland Vital Registration - Deaths 1995 ICD9.
United Kingdom - Scotland Vital Registration - Deaths 1996 ICD9.
United Kingdom - Scotland Vital Registration - Deaths 1997 ICD9.
United Kingdom - Scotland Vital Registration - Deaths 1998 ICD9.
United Kingdom - Scotland Vital Registration - Deaths 1999 ICD9.
United Kingdom - Scotland Vital Registration - Deaths 2000 ICD10.
United Kingdom - Scotland Vital Registration - Deaths 2001 ICD10.
United Kingdom - Scotland Vital Registration - Deaths 2002 ICD10.
United Kingdom - Scotland Vital Registration - Deaths 2003 ICD10.
United Kingdom - Scotland Vital Registration - Deaths 2004 ICD10.
United Kingdom - Scotland Vital Registration - Deaths 2005 ICD10.
United Kingdom - Scotland Vital Registration - Deaths 2006 ICD10.
United Kingdom - Scotland Vital Registration - Deaths 2007 ICD10.
United Kingdom - Scotland Vital Registration - Deaths 2008 ICD10.
United Kingdom - Scotland Vital Registration - Deaths 2009 ICD10.
United Kingdom - Scotland Vital Registration - Deaths 2010 ICD10.
United Kingdom - Scotland Vital Registration - Deaths 2011 ICD10.
United Kingdom - Scotland Vital Registration - Deaths 2012 ICD10.
United Kingdom - Scotland Vital Registration - Deaths 2013 ICD10.

United Kingdom - Scotland Vital Registration - Deaths 2014 ICD10.
United Kingdom - Scotland Vital Registration - Deaths 2017 ICD10.
United Kingdom - Scotland Vital Registration - Deaths 2018 ICD10.
United States Virgin Islands Vital Registration - Deaths 1980 ICD9.
Uruguay Vital Registration - Deaths 1980 ICD9.
Uruguay Vital Registration - Deaths 1981 ICD9.
Uruguay Vital Registration - Deaths 1982 ICD9.
Uruguay Vital Registration - Deaths 1983 ICD9.
Uruguay Vital Registration - Deaths 1984 ICD9.
Uruguay Vital Registration - Deaths 1985 ICD9.
Uruguay Vital Registration - Deaths 1986 ICD9.
Uruguay Vital Registration - Deaths 1987 ICD9.
Uruguay Vital Registration - Deaths 1988 ICD9.
Uruguay Vital Registration - Deaths 1989 ICD9.
Uruguay Vital Registration - Deaths 1990 ICD9.
Uruguay Vital Registration - Deaths 1993 ICD9.
Uruguay Vital Registration - Deaths 1994 ICD9.
Uruguay Vital Registration - Deaths 1995 ICD9.
Uruguay Vital Registration - Deaths 1996 ICD9.
Uruguay Vital Registration - Deaths 1997 ICD10.
Uruguay Vital Registration - Deaths 1998 ICD10.
Uruguay Vital Registration - Deaths 1999 ICD10.
Uruguay Vital Registration - Deaths 2000 ICD10.
Uruguay Vital Registration - Deaths 2001 ICD10.
Uruguay Vital Registration - Deaths 2002 ICD10.
Uruguay Vital Registration - Deaths 2003 ICD10.
Uruguay Vital Registration - Deaths 2004 ICD10.
Uruguay Vital Registration - Deaths 2005 ICD10.
Uruguay Vital Registration - Deaths 2006 ICD10.
Uruguay Vital Registration - Deaths 2007 ICD10.
Uruguay Vital Registration - Deaths 2008 ICD10.
Uruguay Vital Registration - Deaths 2009 ICD10.
Uruguay Vital Registration - Deaths 2010 ICD10.
Uruguay Vital Registration - Deaths 2012 ICD10.
Uruguay Vital Registration - Deaths 2013 ICD10.
Uruguay Vital Registration - Deaths 2014 ICD10.
Uruguay Vital Registration - Deaths 2015 ICD10.
Uruguay Vital Registration - Deaths 2016 ICD10.
Uruguay Vital Registration - Deaths 2017 ICD10.
Uzbekistan Vital Registration - Deaths 1981 ICD9.
Uzbekistan Vital Registration - Deaths 1982 ICD9.
Uzbekistan Vital Registration - Deaths 1985 ICD9.
Uzbekistan Vital Registration - Deaths 1986 ICD9.
Uzbekistan Vital Registration - Deaths 1987 ICD9.
Uzbekistan Vital Registration - Deaths 1988 ICD9.
Uzbekistan Vital Registration - Deaths 1989 ICD9.
Uzbekistan Vital Registration - Deaths 1990 ICD9.
Uzbekistan Vital Registration - Deaths 1991 ICD9.
Uzbekistan Vital Registration - Deaths 1992 ICD9.
Uzbekistan Vital Registration - Deaths 1993 ICD9.
Uzbekistan Vital Registration - Deaths 1994 ICD9.
Uzbekistan Vital Registration - Deaths 1995 ICD9.
Uzbekistan Vital Registration - Deaths 1996 ICD9.
Uzbekistan Vital Registration - Deaths 1997 ICD9.
Uzbekistan Vital Registration - Deaths 1998 ICD9.
Uzbekistan Vital Registration - Deaths 1999 ICD9.
Uzbekistan Vital Registration - Deaths 2000 ICD9.
Uzbekistan Vital Registration - Deaths 2001 ICD9.
Uzbekistan Vital Registration - Deaths 2002 ICD9.
Uzbekistan Vital Registration - Deaths 2003 ICD9.
Uzbekistan Vital Registration - Deaths 2004 ICD10.

Uzbekistan Vital Registration - Deaths 2005 ICD10.
Uzbekistan Vital Registration - Deaths 2009 ICD10.
Uzbekistan Vital Registration - Deaths 2010 ICD10.
Uzbekistan Vital Registration - Deaths 2011 ICD10.
Uzbekistan Vital Registration - Deaths 2012 ICD10.
Uzbekistan Vital Registration - Deaths 2013 ICD10.
Uzbekistan Vital Registration - Deaths 2014 ICD10.
Uzbekistan Vital Registration - Deaths 2015 ICD10.
Uzbekistan Vital Registration - Deaths 2016 ICD10.
Venezuela Vital Registration - Deaths 1980 ICD9.
Venezuela Vital Registration - Deaths 1981 ICD9.
Venezuela Vital Registration - Deaths 1982 ICD9.
Venezuela Vital Registration - Deaths 1983 ICD9.
Venezuela Vital Registration - Deaths 1985 ICD9.
Venezuela Vital Registration - Deaths 1986 ICD9.
Venezuela Vital Registration - Deaths 1987 ICD9.
Venezuela Vital Registration - Deaths 1988 ICD9.
Venezuela Vital Registration - Deaths 1989 ICD9.
Venezuela Vital Registration - Deaths 1990 ICD9.
Venezuela Vital Registration - Deaths 1992 ICD9.
Venezuela Vital Registration - Deaths 1993 ICD9.
Venezuela Vital Registration - Deaths 1994 ICD9.
Venezuela Vital Registration - Deaths 1996 ICD10.
Venezuela Vital Registration - Deaths 1997 ICD10.
Venezuela Vital Registration - Deaths 1998 ICD10.
Venezuela Vital Registration - Deaths 1999 ICD10.
Venezuela Vital Registration - Deaths 2000 ICD10.
Venezuela Vital Registration - Deaths 2001 ICD10.
Venezuela Vital Registration - Deaths 2002 ICD10.
Venezuela Vital Registration - Deaths 2003 ICD10.
Venezuela Vital Registration - Deaths 2004 ICD10.
Venezuela Vital Registration - Deaths 2005 ICD10.
Venezuela Vital Registration - Deaths 2006 ICD10.
Venezuela Vital Registration - Deaths 2007 ICD10.
Venezuela Vital Registration - Deaths 2008 ICD10.
Venezuela Vital Registration - Deaths 2009 ICD10.
Venezuela Vital Registration - Deaths 2010 ICD10.
Venezuela Vital Registration - Deaths 2011 ICD10.
Venezuela Vital Registration - Deaths 2012 ICD10.
Venezuela Vital Registration - Deaths 2013 ICD10.
Venezuela Vital Registration - Deaths 2014 ICD10.
World Health Organization (WHO). Armenia Vital Registration - Deaths 2019 ICD10.
World Health Organization (WHO). Austria Vital Registration - Deaths 2019 ICD10.
World Health Organization (WHO). Bahamas Vital Registration - Deaths 2015 ICD10.
World Health Organization (WHO). Brunei Darussalam Vital Registration - Deaths 2019 ICD10.
World Health Organization (WHO). Canada Vital Registration - Deaths 2018 ICD10.
World Health Organization (WHO). Canada Vital Registration - Deaths 2019 ICD10.
World Health Organization (WHO). Czech Republic Vital Registration - Deaths 2019 ICD10.
World Health Organization (WHO). Egypt Vital Registration - Deaths 2019 ICD10.
World Health Organization (WHO). Estonia Vital Registration - Deaths 2019 ICD10.
World Health Organization (WHO). Georgia Vital Registration - Deaths 2019 ICD10.
World Health Organization (WHO). Germany Vital Registration - Deaths 2019 ICD10.
World Health Organization (WHO). Greece Vital Registration - Deaths 2018 ICD10.
World Health Organization (WHO). Hungary Vital Registration - Deaths 2019 ICD10.
World Health Organization (WHO). Kyrgyzstan Vital Registration - Deaths 2018 ICD10.
World Health Organization (WHO). Kyrgyzstan Vital Registration - Deaths 2019 ICD10.
World Health Organization (WHO). Lebanon Vital Registration - Deaths 2019 ICD10.
World Health Organization (WHO). Lithuania Vital Registration - Deaths 2019 ICD10.
World Health Organization (WHO). Mauritius Vital Registration - Deaths 2019 ICD10.
World Health Organization (WHO). Panama Vital Registration - Deaths 2018 ICD10.
World Health Organization (WHO). Qatar Vital Registration - Deaths 2019 ICD10.

World Health Organization (WHO). Singapore Vital Registration - Deaths 2019 ICD10.
World Health Organization (WHO). Slovenia Vital Registration - Deaths 2019 ICD10.
World Health Organization (WHO). South Korea Vital Registration - Deaths 2019 ICD10.
World Health Organization (WHO). Sri Lanka Vital Registration - Deaths 2004 ICD10.
World Health Organization (WHO). Sri Lanka Vital Registration - Deaths 2005 ICD10.
World Health Organization (WHO). Sri Lanka Vital Registration - Deaths 2007 ICD10.
World Health Organization (WHO). Sri Lanka Vital Registration - Deaths 2011 ICD10.
World Health Organization (WHO). Sri Lanka Vital Registration - Deaths 2012 ICD10.
World Health Organization (WHO). Sri Lanka Vital Registration - Deaths 2013 ICD10.
World Health Organization (WHO). Sri Lanka Vital Registration - Deaths 2014 ICD10.
World Health Organization (WHO). Tajikistan Vital Registration - Deaths 2017 ICD10.
World Health Organization (WHO). Thailand Vital Registration - Deaths 2019 ICD10.
World Health Organization (WHO). Turkey Vital Registration - Deaths 2019 ICD10.
World Health Organization (WHO). United Kingdom - Northern Ireland Vital Registration - Deaths 2017 ICD10.
World Health Organization (WHO). United Kingdom - Scotland Vital Registration - Deaths 2019 ICD10.
World Health Organization (WHO). Uzbekistan Vital Registration - Deaths 2017 ICD10.
World Health Organization (WHO). Uzbekistan Vital Registration - Deaths 2018 ICD10.
World Health Organization (WHO). Uzbekistan Vital Registration - Deaths 2019 ICD10.
Yugoslavia - Bosnia and Herzegovina Vital Registration - Deaths 1985 ICD9.
Yugoslavia - Bosnia and Herzegovina Vital Registration - Deaths 1986 ICD9.
Yugoslavia - Bosnia and Herzegovina Vital Registration - Deaths 1987 ICD9.
Yugoslavia - Bosnia and Herzegovina Vital Registration - Deaths 1988 ICD9.
Yugoslavia - Bosnia and Herzegovina Vital Registration - Deaths 1989 ICD9.
Yugoslavia - Bosnia and Herzegovina Vital Registration - Deaths 1990 ICD9.
Yugoslavia - Croatia Vital Registration - Deaths 1985 ICD9.
Yugoslavia - Croatia Vital Registration - Deaths 1986 ICD9.
Yugoslavia - Croatia Vital Registration - Deaths 1987 ICD9.
Yugoslavia - Croatia Vital Registration - Deaths 1988 ICD9.
Yugoslavia - Croatia Vital Registration - Deaths 1989 ICD9.
Yugoslavia - Croatia Vital Registration - Deaths 1990 ICD9.
Yugoslavia - Slovenia Vital Registration - Deaths 1985 ICD9.
Yugoslavia - Slovenia Vital Registration - Deaths 1986 ICD9.
Yugoslavia - Slovenia Vital Registration - Deaths 1987 ICD9.
Yugoslavia - Slovenia Vital Registration - Deaths 1988 ICD9.
Yugoslavia - Slovenia Vital Registration - Deaths 1990 ICD9.
Yugoslavia, Federal Republic - Montenegro Vital Registration - Deaths 2000 ICD10.
Yugoslavia, Federal Republic - Serbia Vital Registration - Deaths 1998 ICD10.
Yugoslavia, Federal Republic - Serbia Vital Registration - Deaths 1999 ICD10.
Yugoslavia, Federal Republic - Serbia Vital Registration - Deaths 2000 ICD10.
Yugoslavia, Federal Republic - Serbia Vital Registration - Deaths 2001 ICD10.
Yugoslavia, Federal Republic - Serbia Vital Registration - Deaths 2002 ICD10.
Zimbabwe Vital Registration - Deaths 1990 ICD9.

Note that this table includes all data sources for carbon monoxide poisoning deaths available in the GBD, from 1980 onward.

Section 5 Collaborating Authors and Authors' Contributions

Section 5.1 GBD 2021 Carbon Monoxide Poisoning Collaborators

Madeline E Moberg, Erin B Hamilton, Scott M Zeng, Dana Bryazka, Jeff T Zhao, Rachel Feldman, Yohannes Habtegiorgis Abate, Mohsen Abbasi-Kangevari, Ame Mehadi Abdurehman, Aidin Abedi, Eman Abu-Gharbieh, Isaac Yeboah Addo, Abiola Victor Adepoju, Qorinah Estiningtyas Sakilah Adnani, Saira Afzal, Bright Opoku Ahinkorah, Sajjad Ahmad, Danial Ahmed, Haroon Ahmed, Dejene Tsegaye Alem, Adel Ali Saeed Al-Gheethi, Yousef Alimohamadi, Edward Kwabena Ameyaw, Mohammad Amrollahi-Sharifabadi, Tadele Fentabil Anagaw, Anayochukwu Edward Anyasodor, Jalal Arabloo, Aleksandr Y Aravkin, Seyyed Shamsadin Athari, Alok Atreya, Amirhossein Azari Jafari, Ashish D Badiye, Nayereh Baghcheghi, Sara Bagherieh, Hansi Bansal, Amadou Barrow, Azadeh Bashiri, Nebiyou Simegnew Bayileyegn, Alemshet Yirga Berhie, Akshaya Srikanth Bhagavathula, Pankaj Bhardwaj, Archith Boloor, Luis Alberto Cámera, Felix Carvalho, Márcia Carvalho, Eeshwar K Chandrasekar, Jung-Chen Chang, Vijay Kumar Chattu, Dinh-Toi Chu, Kaleb Coberly, Natália Cruz-Martins, Omid Dadras, Xiaochen Dai, Reza Darvishi Cheshmeh Soltani, Saswati Das, Subasish Das, Sisay Abebe Debela, Berecha Hundessa Demessa, Xinlei Deng, Abebaw Alemayehu Desta, Belay Desye, Meghnath Dhimal, Mahmoud Dibas, Haneil Larson Dsouza, Michael Ekholuenetale, Iman El Sayed, Waseem El-Huneidi, Daniel Berhanie Enyew, Adeniyi Francis Fagbamigbe, Ali Fatehizadeh, Syeda Anum Fatima Fatima, Florian Fischer, Richard Charles Franklin, Tushar Garg, Tilaye Gebru Gebi, Urge Gerema, Melaku Getachew, Motuma Erena Getachew, Farhad Ghamari, Mahaveer Golechha, Pouya Goleij, Sapna Gupta, Veer Bala Gupta, Vivek Kumar Gupta, Mehdi Harorani, Hamidreza Hasani, Abbas M Hassan, Hossein Hassanian-Moghaddam, Mohammed Bheser Hassen, Simon I Hay, Khezar Hayat, Mohammad Heidari, Mahsa Heidari-Foroozan, Demisu Zenbaba Heyi, Ramesh Holla, Praveen Hoogar, Md Shakhaoat Hossain, Mohammad-Salar Hosseini, Sorin Hostiuc, Soodabeh Hoveidamanesh, Olayinka Stephen Ilesanmi, Irena M Ilic, Mustapha Immurana, Chidozie C D Iwu, Umesh Jayarajah, Nitin Joseph, Charity Ehimwenma Joshua, Vidya Kadashetti, Tanuj Kanchan, Himal Kandel, Rami S Kantar, Neeti Kapoor, Ibraheem M Karaye, Patrick DMC Katoto, Himanshu Khajuria, Ejaz Ahmad Khan, Sorour Khateri, Farzad Khodamoradi, Moein Khormali, Jagdish Khubchandani, Grace Kim, Adnan Kisa, Hamid Reza Koohestani, Kewal Krishan, Naveen Kumar, Lucie Laflamme, Iván Landires, Bagher Larijani, Paolo Lauriola, Thao Thi Thu Le, Caterina Ledda, Seung Won Lee, Stephen S Lim, Stany W Lobo, Raimundas Lunevicius, Sandeep B Maharaj, Ritesh G Menezes, Alexios-Fotios A Mentis, Tomislav Mestrovic, Ted R Miller, Seyyedmohammadsadeq Mirmoeeni, Awoke Misganaw, Manish Mishra, Sanjeev Misra, Chaitanya Mittal, Esmaeil Mohammadi, Ali H Mokdad, Mohammad Ali Moni, Ebrahim Mostafavi, Sumaira Mubarik, Francesk Mulita, Jember Azanaw Mulualem, Temesgen Mulugeta, Christopher J L Murray, Isabella Myers, Biswa Prakash Nayak, Vinod C Nayak, Seyed Aria Nejadghaderi, Huong Lan Thi Nguyen, Van Thanh Nguyen, Hasti Nouraei, Ogochukwu Janet Nzoputam, Hassan Okati-Aliabad, Isaac Iyinoluwa Olufadewa, Michal Ordak, Alicia Padron-Monedero, Jagadish Rao Padubidri, Ashok Pandey, Suman Pant, Utsav Parekh, Shrikant Pawar, Amy E Peden, Ionela-Roxana Petcu, Frédéric B Piel, Zahra Zahid Piracha, Ghazaleh Pourali, Ibrahim Qattea, Maryam Faiz Qureshi, Pankaja Raghav Raghav, Mosiur Rahman, Shayan Rahmani, Premkumar Ramasubramani, Sheena Ramazanu, Salman Rawaf, Nazila Rezaei, Negar Rezaei, Mohsen Rezaeian, Basema Saddik, Malihe Sadeghi, Farideh Sadeghian, Umar Saeed, Amirhossein Sahebkar, Zahra Saif, Joseph W Sakshaug, Saina Salahi, Payman Salamati, Abdallah M Samy, Rodrigo Sarmiento-Suárez, David C Schwebel, Subramanian Senthilkumaran, Allen Seylani, Masood Ali Shaikh, Sunder Sham, Bereket Beyene Shashamo, Rahim Ali Sheikhi, B Suresh Kumar Shetty, Pavanchand H Shetty, Migbar Mekonnen

Sibhat, Harpreet Singh, Paramdeep Singh, Eskinder Ayalew Sisay, Yonatan Solomon, Majid Taheri, Irfan Ullah, Sana Ullah, Francesco S Violante, Linh Gia Vu, Nuwan Darshana Wickramasinghe, Arzu Yigit, Naohiro Yonemoto, Zabihollah Yousefi, Muhammad Zaman, Mikhail Sergeevich Zastrozhin, Zhi-Jiang Zhang, Peng Zheng, Mohammad Zoladl, Jaimie D Steinmetz, Theo Vos, Mohsen Naghavi, and Kanyin Liane Ong.

Section 5.2 Affiliations

Institute for Health Metrics and Evaluation (M E Moberg MS, E B Hamilton MPH, S M Zeng MSc, D Bryazka BA, J T Zhao BA, R Feldman BS, A Y Aravkin PhD, K Coberly BS, X Dai PhD, M Hassen BSc, Prof S I Hay FMedSci, Prof S S Lim PhD, T Mestrovic PhD, A H Mokdad PhD, Prof C J L Murray DPhil, P Zheng PhD, J D Steinmetz PhD, Prof T Vos PhD, Prof M Naghavi PhD, K L Ong PhD), Department of Applied Mathematics (A Y Aravkin PhD), Department of Health Metrics Sciences, School of Medicine (A Y Aravkin PhD, X Dai PhD, Prof S I Hay FMedSci, Prof S S Lim PhD, A Misganaw PhD, A H Mokdad PhD, Prof C J L Murray DPhil, P Zheng PhD, Prof T Vos PhD, Prof M Naghavi PhD), University of Washington, Seattle, WA, USA; Department of Clinical Governance and Quality Improvement (Y H Abate MSc), Aleta Wondo Hospital, Aleta Wondo, Ethiopia; Non-Communicable Diseases Research Center (NCDRC) (M Abbasi-Kangevari MD, S Rahmani MD, N Rezaei MD, N Rezaei PhD), Sina Trauma and Surgery Research Center (M Khormali MD, F Sadeghian PhD, Prof P Salamati MD), Endocrinology and Metabolism Research Institute (Prof B Larijani FACE, N Rezaei PhD), Faculty of Medicine (E Mohammadi MD), Tehran University of Medical Sciences, Tehran, Iran (E Mohammadi MD); Department of Emergency and Critical Care Nursing (A M Abdurehman MSc), Department of Health Informatics (D B Enyew MSc), Health Sciences Department of Oncology Nursing (T G Gebi MSc), Department of Emergency and Critical Care Medicine (M Getachew MD), Haramaya University, Harar, Ethiopia; Department of Neurosurgery (A Abedi MD), Keck School of Medicine (A Abedi MD), University of Southern California, Los Angeles, CA, USA; Clinical Sciences Department (E Abu-Gharbieh PhD), Department of Basic Medical Sciences (W El-Huneidi PhD), Sharjah Institute for Medical Research (B Saddik PhD), University of Sharjah, Sharjah, United Arab Emirates; Centre for Social Research in Health (I Y Addo PhD), School of Public Health and Community Medicine (A E Peden PhD), University of New South Wales, Sydney, NSW, Australia; Quality and Systems Performance Unit (I Y Addo PhD), Cancer Institute NSW, Sydney, NSW, Australia; HIV and Infectious Diseases Department (A V Adepoju MD), Jhpiego, Abuja, Nigeria; Adolescent Research and Care (A V Adepoju MD), Adolescent Friendly Research Initiative and Care, Ado Ekiti, Nigeria; Department of Faculty of Medicine (Q E S Adnani PhD), Universitas Padjadjaran (Padjadjaran University), Bandung, Indonesia; Department of Community Medicine (Prof S Afzal PhD), King Edward Memorial Hospital, Lahore, Pakistan; Department of Public Health (Prof S Afzal PhD), Public Health Institute, Lahore, Pakistan; School of Public Health (B O Ahinkorah MPhil), University of Technology Sydney, Sydney, NSW, Australia; Department of Health and Biological Sciences (S Ahmad PhD), Abasyn University, Peshawar, Pakistan; Department of Life Sciences (D Ahmed BS), NYIT College Of Osteopathic Medicine, Old Westbury, NY, USA; Department of Biosciences (H Ahmed PhD), COMSATS Institute of Information Technology, Islamabad, Pakistan; Department of Nursing (D T Alem MSc), Debre Markos University, Debremarkos, Ethiopia; Micropollutant Research Centre (MPRC) (A A S Al-Gheethi PhD), Universiti Tun Hussein Onn Malaysia (UTHM) (Tun Hussein Onn University of Malaysia), Batu Pahat, Malaysia; Camborne School of Mines (A A S Al-Gheethi PhD), University of Exeter, Penryn, UK; Pars Advanced and Minimally Invasive Medical Manners Research Center (Y Alimohamadi PhD), Health Management and

Economics Research Center (J Arabloo PhD), Trauma and Injury Research Center (M Taheri PhD), Iran University of Medical Sciences, Tehran, Iran; School of Graduate Studies (E K Ameyaw MPhil), Lingnan University, Hong Kong, China; Faculty of Veterinary Medicine (M Amrollahi-Sharifabadi PhD), Lorestan University, Khorramabad, Iran; Department of Health Promotion (T F Anagaw MPH), School of Health Science (A Y Berhie MSc), Bahir Dar University, Bahir Dar, Ethiopia; School of Dentistry and Medical Sciences (A E Anyasodor PhD), Charles Sturt University, Orange, NSW, Australia; Department of Immunology (S Athari PhD), Zanjan University of Medical Sciences, Zanjan, Iran; Department of Forensic Medicine (A Atreya MD), Lumbini Medical College, Palpa, Nepal; School of Medicine (A Azari Jafari MD, S Mirmoeeni MD), Center for Health Related Social and Behavioral Sciences Research (F Sadeghian PhD), Shahroud University of Medical Sciences, Shahroud, Iran; Department of Forensic Science (A D Badiye PhD, H Bansal MSc, N Kapoor PhD), Government Institute of Forensic Science, Nagpur, India; Department of Nursing (N Baghcheghi PhD), Social Determinants of Health Research Center (H Koohestani PhD), Saveh University of Medical Sciences, Saveh, Iran; School of Medicine (S Bagherieh BSc), Department of Environmental Health Engineering (A Fatehizadeh PhD), Isfahan University of Medical Sciences, Isfahan, Iran; Department of Public & Environmental Health (A Barrow MPH), University of The Gambia, Brikama, The Gambia; Epidemiology and Disease Control Unit (A Barrow MPH), Ministry of Health, Kotu, The Gambia; Health Information Management (A Bashiri PhD), Department of Medical Mycology and Parasitology (H Nouraei MSc), Shiraz University of Medical Sciences, Shiraz, Iran; Department of Surgery (N S Bayileyegn MD), Department of Public Health (U Gerema MSc, M E Getachew MPH), Department of Clinical Pharmacy (T Mulugeta MSc), Jimma University, Jimma, Ethiopia; Department of Health, Human Performance and Recreation (A S Bhagavathula PhD), University of Arkansas, Fayetteville, AR, USA; Department of Community Medicine and Family Medicine (P Bhardwaj MD, Prof P R Raghav MD), School of Public Health (P Bhardwaj MD), Department of Forensic Medicine and Toxicology (T Kanchan MD), Department of Surgical Oncology (Prof S Misra MCh), All India Institute of Medical Sciences, Jodhpur, India; Department of Internal Medicine (A Boloor MD), Department of Forensic Medicine and Toxicology (H L Dsouza MD, Prof B K Shetty MD, P H Shetty MD), Department of Community Medicine (N Joseph MD), Manipal Academy of Higher Education, Mangalore, India; Internal Medicine Department (Prof L A Cámera MD), Hospital Italiano de Buenos Aires (Italian Hospital of Buenos Aires), Buenos Aires, Argentina; Board of Directors (Prof L A Cámera MD), Argentine Society of Medicine, Buenos Aires, Argentina; Research Unit on Applied Molecular Biosciences (UCIBIO) (Prof F Carvalho PhD, M Carvalho PhD), Institute for Research and Innovation in Health (Prof N Cruz-Martins PhD), University of Porto, Porto, Portugal; Faculty of Health Sciences (M Carvalho PhD), University Fernando Pessoa, Porto, Portugal; Department of Anesthesiology and Perioperative Medicine (E K Chandrasekar MD), University of Rochester, Rochester, NY, USA; College of Medicine (J Chang PhD), National Taiwan University, Taipei, Taiwan; Department of Nursing (J Chang PhD), National Taiwan University Hospital, Taipei, Taiwan; Temerty Faculty of Medicine (V Chattu MD), University of Toronto, Toronto, ON, Canada; Saveetha Dental College, SIMATS (V Chattu MD), Saveetha University, Chennai, India; Center for Biomedicine and Community Health (D Chu PhD), VNU-International School, Hanoi, Viet Nam; Therapeutic and Diagnostic Technologies (Prof N Cruz-Martins PhD), Cooperativa de Ensino Superior Politécnico e Universitário (Polytechnic and University Higher Education Cooperative), Gandra, Portugal; Section Global Health and Rehabilitation (O Dadras DrPH), Western Norway University of Applied Sciences, Bergen, Norway; Department of Global Public Health and Primary Care (O Dadras DrPH), University of Bergen, Bergen, Norway; Department of Environmental Health (R Darvishi Cheshmeh Soltani PhD), Occupational Health Department (F Ghamari PhD),

Department of Nursing (M Harorani MSc), Arak University of Medical Sciences, Arak, Iran; Department of Biochemistry (S Das MD), Ministry of Health and Welfare, New Delhi, India; Ingram School of Engineering (S Das PhD), Texas State University, San Marcos, TX, USA; School of Public Health (S Debela MPH), Salale University, Fiche, Ethiopia; The United States Agency for International Development's (USAID) JSI (B H Demessa MPH), Jimma University, Addis Ababa, Ethiopia; Epidemiology Branch (X Deng PhD), National Institute of Health, Durham, NC, USA; Department of Surgical Nursing (A A Desta MSc), Department of Environmental and Occupational Health and Safety (J A Mulualem MSc), University of Gondar, Gondar, Ethiopia; Department of Public Health (B Desye MSc), Adigrat University, Adigrat, Ethiopia; Health Research Section (M Dhimal PhD), Research Department (A Pandey MPH), Department of Clinical Research (S Pant MPH), Nepal Health Research Council, Kathmandu, Nepal; Research Unit (M Dibas MD), Sulaiman Al Rajhi University, Qassim, Saudi Arabia; Forensic Medicine and Toxicology (H L Dsouza MD), Kasturba Medical College Mangalore, Mangalore, Dakshina Kannada District, Karnataka State, India; Department of Epidemiology and Medical Statistics (M Ekholuenetale MSc, A F Fagbamigbe PhD), Faculty of Public Health (M Ekholuenetale MSc, I I Olufadewa MHS), Department of Community Medicine (O S Ilesanmi PhD), University of Ibadan, Ibadan, Nigeria; Biomedical Informatics and Medical Statistics Department (I El Sayed PhD), Alexandria University, Alexandria, Egypt; Institute of Applied Health Sciences (A F Fagbamigbe PhD), University of Aberdeen, Aberdeen, UK; Department of Community Medicine and Global Health (S A F Fatima MPhil), University of Oslo, Oslo, Norway; Department of Bacteriologi (S A F Fatima MPhil), Norwegian Institute of Public Health, Oslo, Norway; Institute of Public Health (F Fischer PhD), Charité Universitätsmedizin Berlin (Charité Medical University Berlin), Berlin, Germany; School of Public Health, Medical, and Veterinary Sciences (R C Franklin PhD), James Cook University, Douglas, QLD, Australia; Department of Radiology (T Garg MBBS), King Edward Memorial Hospital, Mumbai, India; Department of Public Health (M E Getachew MPH), Wollega University, Nekemte, Ethiopia; Health Systems and Policy Research Department (M Golechha PhD), Indian Institute of Public Health, Gandhinagar, India; Department of Genetics (P Goleij MSc), Sana Institute of Higher Education, Sari, Iran; Toxicology Department (S Gupta MSc), Shriram Institute for Industrial Research, Delhi, India; School of Medicine (V Gupta PhD), Deakin University, Geelong, VIC, Australia; Faculty of Medicine Health and Human Sciences (Prof V K Gupta PhD), Macquarie University, Sydney, NSW, Australia; Department of Ophthalmology (H Hasani MD), Iran University of Medical Sciences, Karaj, Iran; Department of Plastic Surgery (A M Hassan MD), University of Texas, Houston, TX, USA; Social Determinants of Health Research Center (Prof H Hassanian-Moghaddam MD), Department of Medicine (M Heidari-Foroozan BMedSc), School of Medicine (S Nejadghaderi MD, S Rahmani MD), Medical Ethics and Law Research Center (M Taheri PhD), Shahid Beheshti University of Medical Sciences, Tehran, Iran; Chapter of Addiction Medicine (Prof H Hassanian-Moghaddam MD), Save Sight Institute (H Kandel PhD), University of Sydney, Sydney, NSW, Australia; National Data Management Center for Health (NDMC) (M Hassen BSc), National Data Management Center for Health (A Misganaw PhD), Ethiopian Public Health Institute, Addis Ababa, Ethiopia; Institute of Pharmaceutical Sciences (K Hayat MS), University of Veterinary and Animal Sciences, Lahore, Pakistan; Department of Pharmacy Administration and Clinical Pharmacy (K Hayat MS), Xian Jiaotong University, Xian, China; Community-Oriented Nursing Midwifery Research Center (M Heidari PhD), Department of Health in Disasters and Emergencies (R Sheikhi BHlthSci), Shahrekord University of Medical Sciences, Shahrekord, Iran; Department of Epidemiology (M Heidari-Foroozan BMedSc, S Nejadghaderi MD), Non-Communicable Diseases Research Center (NCDRC), Tehran, Iran; Department of Public Health (D Z Heyi MPH), Madda Walabu University, Robe, Ethiopia; Kasturba Medical College, Mangalore (R Holla MD), Department of

Forensic Medicine and Toxicology (Prof V C Nayak MD), Manipal Academy of Higher Education, Manipal, India; School of Social Sciences (P Hoogar PhD), The Apollo University, Chittoor, India; Department of Public Health and Informatics (M S Hossain PhD), Jahangirnagar University, Dhaka, Bangladesh; Research Center for Evidence-Based Medicine (M Hosseini MD), Tabriz University of Medical Sciences, Tabriz, Iran; Department of Legal Medicine and Bioethics (S Hostiuc PhD), Carol Davila University of Medicine and Pharmacy, Bucharest, Romania; Clinical Legal Medicine Department (S Hostiuc PhD), National Institute of Legal Medicine Mina Minovici, Bucharest, Romania; Burn Research Center (S Hoveidamanesh MD), Shahid Motahari Hospital, Tehran, Iran; Department of Community Medicine (O S Ilesanmi PhD), University College Hospital, Ibadan, Ibadan, Nigeria; Faculty of Medicine (I M Ilic PhD), University of Belgrade, Belgrade, Serbia; Institute of Health Research (M Immurana PhD), University of Health and Allied Sciences, Ho, Ghana; School of Health Systems and Public Health (C C D Iwu MPH), University of Pretoria, Pretoria, South Africa; Postgraduate Institute of Medicine (U Jayarajah MBBS), University of Colombo, Colombo, Sri Lanka; Department of Surgery (U Jayarajah MBBS), National Hospital, Colombo, Sri Lanka; Department of Economics (C E Joshua BSc), National Open University, Benin City, Nigeria; Department of Oral and Maxillofacial Pathology (V Kadashetti MDS), Krishna institute of Medical Sciences Deemed to be University, Karad, India; Sydney Eye Hospital (H Kandel PhD), South Eastern Sydney Local Health District, Sydney, NSW, Australia; The Hansjörg Wyss Department of Plastic and Reconstructive Surgery (R S Kantar MD), Nab'a Al-Hayat Foundation for Medical Sciences and Health Care, New York, NY, USA; Department of Cleft Lip and Palate Surgery (R S Kantar MD), Global Smile Foundation, Norwood, MA, USA; School of Health Professions and Human Services (I M Karaye MD), Hofstra University, Hempstead, NY, USA; Centre for Tropical Diseases and Global Health (P D Katoto PhD), Catholic University of Bukavu, Bukavu, Democratic Republic of the Congo; Department of Global Health (P D Katoto PhD), Stellenbosch University, Cape Town, South Africa; Amity Institute of Forensic Sciences (H Khajuria PhD, B P Nayak PhD), Amity University, Noida, India; Department of Epidemiology and Biostatistics (E A Khan MPH), Health Services Academy, Islamabad, Pakistan; School of Medicine (S Khateri MD), Kurdistan University of Medical Sciences, Sanandaj, Iran; Department of Social Medicine (F Khodamoradi PhD), Ahvaz Jundishapur University of Medical Sciences, Ahvaz, Iran; Department of Public Health (Prof J Khubchandani PhD), New Mexico State University, Las Cruces, NM, USA; Department of Pediatrics (G Kim MD), Department of Neonatology (I Qattea MD), Case Western Reserve University, Cleveland, OH, USA; Division of Pediatric Hospital Medicine (G Kim MD), UH Rainbow Babies and Children's Hospital, Cleveland, OH, USA; School of Health Sciences (Prof A Kisa PhD), Kristiania University College, Oslo, Norway; Department of International Health and Sustainable Development (Prof A Kisa PhD), Tulane University, New Orleans, LA, USA; Department of Anthropology (Prof K Krishan PhD), Panjab University, Chandigarh, India; Amity Institute of Biotechnology (N Kumar PhD), Amity University Rajasthan, Jaipur, India; Department of Global Public Health (Prof L Laflamme PhD), Karolinska Institute, Stockholm, Sweden; Institute for Social and Health Sciences (Prof L Laflamme PhD), University of South Africa, Pretoria, South Africa; Unit of Genetics and Public Health (Prof I Landires MD), Institute of Medical Sciences, Las Tablas, Panama; Ministry of Health, Herrera, Panama (Prof I Landires MD); International Society Doctors for the Environment, Arezzo, Italy (P Lauriola MD); Department of General Medicine (V T Nguyen MD), University of Medicine and Pharmacy at Ho Chi Minh City, Ho Chi Minh City, Viet Nam (T T Le MD); Clinical and Experimental Medicine (C Ledda PhD), University of Catania, Catania, Italy; Department of Precision Medicine (Prof S W Lee MD), Sungkyunkwan University, Suwon-si, South Korea; Department of Professional and Medical Education (S W Lobo PhD), Meharry Medical College, Nashville, TN, USA; Department of Biomedical Sciences (S W Lobo PhD), Mercer University, Macon, GA,

USA; Department of General Surgery (Prof R Lunevicius DSc), Liverpool University Hospitals NHS Foundation Trust, Liverpool, UK; Department of Surgery (Prof R Lunevicius DSc), University of Liverpool, Liverpool, UK; School of Pharmacy (S B Maharaj DBA), University of the West Indies, St. Augustine, Trinidad and Tobago; Fellow (S B Maharaj DBA), Planetary Health Alliance, Boston, MA, USA; Forensic Medicine Division (Prof R G Menezes MD), Imam Abdulrahman Bin Faisal University, Dammam, Saudi Arabia; International Dx Department (A A Mentis MD), BGI Genomics, Copenhagen, Denmark; University Centre Varazdin (T Mestrovic PhD), University North, Varazdin, Croatia; Pacific Institute for Research & Evaluation, Calverton, MD, USA (T R Miller PhD); School of Public Health (T R Miller PhD), Curtin University, Perth, WA, Australia; Department of Biomedical Sciences (M Mishra PhD), Mercer University School of Medicine, Macon, GA, USA; Department of Forensic Medicine and Toxicology (C Mittal MD), Dr. B. C. Roy Multi-Specialty Medical Research Centre, IIT Kharagpur, Kharagpur, India; School of Health & Rehabilitation Sciences (M Moni PhD), The University of Queensland, Brisbane, QLD, Australia; Department of Medicine (E Mostafavi PhD), Stanford Cardiovascular Institute (E Mostafavi PhD), Stanford University, Palo Alto, CA, USA; Department of Epidemiology and Biostatistics (S Mubarik MS), School of Medicine (Z Zhang PhD), Wuhan University, Wuhan, China; Department of Surgery (F Mulita PhD), General University Hospital of Patras, Patras, Greece; Faculty of Medicine (F Mulita PhD), University of Thessaly, Larissa, Greece; Independent Consultant, Rake, UK (I Myers MSc); Institute for Global Health Innovations (H L T Nguyen MPH), Duy Tan University, Hanoi, Viet Nam; Department of Physiology (O J Nzoputam PhD), University of Benin, Edo, Nigeria; Department of Physiology (O J Nzoputam PhD), Benson Idahosa University, Benin City, Nigeria; Health Promotion Research Center (H Okati-Aliabad PhD), Zahedan University of Medical Sciences, Zahedan, Iran; Slum and Rural Health Initiative Research Academy (I I Olufadewa MHS), Slum and Rural Health Initiative, Ibadan, Nigeria; Department of Pharmacotherapy and Pharmaceutical Care (M Ordak PhD), Medical University of Warsaw, Warsaw, Poland; National School of Public Health (A Padron-Monedero PhD), Institute of Health Carlos III, Madrid, Spain; Department of Forensic Medicine and Toxicology (J Padubidri MD), Kasturba Medical College, Mangalore, Mangalore, India; Research Department (A Pandey MPH), Public Health Research Society Nepal, Kathmandu, Nepal; Department of Forensic Medicine and Toxicology (U Parekh MD), All India Institute of Medical Sciences, Rajkot, India; Department of Genetics (S Pawar PhD), Yale University, New Haven, CT, USA; College of Public Health, Medical, and Veterinary Sciences (A E Peden PhD), James Cook University, Townsville, NSW, Australia; Department of Statistics and Econometrics (I Petcu PhD), Bucharest University of Economic Studies, Bucharest, Romania; School of Public Health (F B Piel PhD), I.M. Sechenov First Moscow State Medical University, London, UK; International Center of Medical Sciences Research, Islamabad, Pakistan (Z Z Piracha PhD); Metabolic Syndrome Research Center (G Pourali MD), International UNESCO Center for Health-related Basic Sciences and Human Nutrition (G Pourali MD), Applied Biomedical Research Center (A Sahebkar PhD), Biotechnology Research Center (A Sahebkar PhD), Mashhad University of Medical Sciences, Mashhad, Iran; Department of Molecular Biology and Biochemistry (M Qureshi MS), University of California Irvine, Irvine, CA, USA; Department of Population Science and Human Resource Development (M Rahman DrPH), University of Rajshahi, Rajshahi, Bangladesh; Department of Community Medicine (P Ramasubramani MD), Mahatma Gandhi Medical College and Research Institute, Puducherry, India; Leadership Institute for Global Health Transformation (LIGHT) (S Ramazanu PhD), National University of Singapore, Singapore; Department of Primary Care and Public Health (Prof S Rawaf MD), Imperial College London, London, UK; Academic Public Health England (Prof S Rawaf MD), Public Health England, London, UK; Department of Epidemiology and Biostatistics (Prof M Rezaeian PhD), Rafsanjan University

of Medical Sciences, Rafsanjan, Iran; Health Information Management (M Sadeghi PhD), Semnan University of Medical Sciences, Semnan, Iran; Multidisciplinary Laboratory Foundation University School of Health Sciences (FUSH) (Prof U Saeed PhD), Foundation University, Islamabad, Pakistan; International Center of Medical Sciences Research (ICMSR), Islamabad, Pakistan (Prof U Saeed PhD); Department of Psychiatry (Z Saif MBA), Ministry of Health, Manama, Bahrain; Institute for Employment Research (J W Sakshaug PhD), University of Warwick, Coventry, UK; Department of Statistics (J W Sakshaug PhD), Ludwig Maximilians University, Munich, Germany; Medical Laboratory (S Salahi BMedSc), Azad University of Medical Sciences, Tehran, Iran; Department of Entomology (A M Samy PhD), Medical Ain Shams Research Institute (MARSI) (A M Samy PhD), Ain Shams University, Cairo, Egypt; Department of Health and Society, Faculty of Medicine (Prof R Sarmiento-Suárez MPH), University of Applied and Environmental Sciences, Bogota, Colombia; National School of Public Health (Prof R Sarmiento-Suárez MPH), Carlos III Health Institute, Madrid, Spain; Department of Psychology (D C Schwebel PhD), University of Alabama at Birmingham, Birmingham, AL, USA; Emergency Department (S Senthilkumaran MD), Manian Medical Centre, Erode, India; National Heart, Lung, and Blood Institute (A Seylani BS), National Institute of Health, Rockville, MD, USA; Independent Consultant, Karachi, Pakistan (M A Shaikh MD); Department of Pathology and Laboratory Medicine (S Sham MBBS), Northwell Health, New York, NY, USA; Department of Nursing (B B Shashamo MSc), Arba Minch University, Arbaminch, Ethiopia; Department of Pediatrics and Child Health Nursing (M M Sibhat MSc), Dilla University, Dilla, Ethiopia; Department of Pulmonary and Critical Care Medicine (H Singh MD), Medical College of Wisconsin, Milwaukee, WI, USA; Department of Radiodiagnosis (P Singh MD), All India Institute of Medical Sciences, Bathinda, India; Department of Clinical Pharmacy (E A Sisay MSc), Addis Ababa University, Addis Ababa, Ethiopia; Department of Pharmacy (E A Sisay MSc), Tikur Anbessa Specialized Hospital, Addis Ababa, Ethiopia; Department of Nursing (Y Solomon MSc), Dire Dawa University, Dire Dawa, Ethiopia; Department of Rehabilitation and Health Sciences (I Ullah PhD), Iqra University, Islamabad, Pakistan; Department of Zoology (S Ullah PhD), University of Education, Lahore, Pakistan; Division of Science and Technology (S Ullah PhD), University of Education, Lahore, Lahore, Pakistan; Department of Medical and Surgical Sciences (Prof F S Violante MD), University of Bologna, Bologna, Italy; Occupational Health Unit (Prof F S Violante MD), Sant'Orsola Malpighi Hospital, Bologna, Italy; Institute for Global Health Innovations (L G Vu MSc), Faculty of Medicine (L G Vu MSc), Duy Tan University, Da Nang, Viet Nam; Department of Community Medicine (N D Wickramasinghe MD), Rajarata University of Sri Lanka, Anuradhapura, Sri Lanka; Department of Health Management (A Yigit PhD), Süleyman Demirel Üniversitesi (Süleyman Demirel University), Isparta, Türkiye; Department of Neuropsychopharmacology (N Yonemoto PhD), National Center of Neurology and Psychiatry, Kodaira, Japan; Department of Public Health (N Yonemoto PhD), Juntendo University, Tokyo, Japan; Department of Environmental Health (Prof Z Yousefi PhD), Mazandaran University of Medical Sciences, Sari, Iran; Faculty of Pharmacy (M Zaman PhD), University of Central Punjab, Lahore, Pakistan; Department of Bioengineering and Therapeutic Sciences (Prof M S Zastrozhin PhD), University of California San Francisco, San Francisco, CA, USA; Addictology Department (Prof M S Zastrozhin PhD), Russian Medical Academy of Continuous Professional Education, Moscow, Russia; Department of Nursing (M Zoladl PhD), Yasuj University of Medical Sciences, Yasuj, Iran.

Section 5.3 Authors' Contributions

Managing the overall research enterprise

Erin Hamilton, Jaimie Steinmetz, Theo Vos, Mohsen Naghavi, Liane Ong.

Writing the first draft of the manuscript

Madeline Moberg

Primary responsibility for applying analytical methods to produce estimates

Madeline Moberg, Dana Bryazka, Jeff Zhao

Primary responsibility for seeking, cataloguing, extracting, or cleaning data; designing or coding figures and tables

Madeline Moberg, Scott M Zeng, Dana Bryazka, Jeff Zhao, Rachel Feldman

Providing data or critical feedback on data sources

Yohannes Habtegiorgis Abate, Ame Mehadi Abdurehman, Aidin Abedi, Abiola Victor Adepoju, Qorinah Estiningtyas Adnani, Saira Afzal, Bright Opoku Ahinkorah, Sajjad Ahmad, Haroon Ahmed, Dejene Tsegaye Alem, Yousef Alimohamadi, Edward Kwabena Ameyaw, Mohammad Amrollahi-Sharifabadi, Jalal Arabloo, Seyyed Shamsadin Athari, Alok Atreya, Ashish D Badiye, Sara Bagherieh, Amadou Barrow, Nebiyou Simegnew Bayileyegn, Akshaya Srikanth Bhagavathula, Archith Boloor, Eeshwar K Chandrasekar, Jung-Chen Chang, Vijay Kumar Chattu, Dinh-Toi Chu, Natália Cruz-Martins, Xiaochen Dai, Meghnath Dhimal, Haneil Larson Dsouza, Michael Ekholuenetale, Iman El Sayed, Adeniyi Francis Fagbamigbe, Ali Fatehizadeh, Richard Charles Franklin, Tushar Garg, Tilaye Gebru Gebi, Melaku Getachew, Mahaveer Golechha, Pouya Goleij, Sapna Gupta, Veer Bala Gupta, Vivek Kumar Gupta, Hossein Hassanian-Moghaddam, Mahsa Heidari-Foroozan, Praveen Hoogar, Olayinka Stephen Ilesanmi, Charity Ehimwenma Joshua, Vidya Kadashetti, Himal Kandel, Rami S Kantar, Neeti Kapoor, Patrick DMC Katoto, Himanshu Khajuria, Sorour Khateri, Jagdish Khubchandani, Grace Kim, Adnan Kisa, Kewal Krishan, Thao Thi Thu Le, Caterina Ledda, Seung Won Lee, Stephen S Lim, Ritesh G Menezes, Ted R Miller, Awoke Misganaw, Madeline E Moberg, Ali H Mokdad, Mohammad Ali Moni, Ebrahim Mostafavi, Sumaira Mubarik, Francesk Mulita, Jember Azanaw Mulualem, Christopher J L Murray, Mohsen Naghavi, Biswa Prakash Nayak, Huong Lan Nguyen, Van Thanh Nguyen, Hasti Nouraei, Ogochukwu Janet Nzoputam, Kanyin Liane Ong, Jagadish Rao Padubidri, Ashok Pandey, Shrikant Pawar, Zahra Zahid Piracha, Ghazaleh Pourali, Shayan Rahmani, Sheena Ramazanu, Salman Rawaf, Basema Saddik, Umar Saeed, Zahra Saif, Abdallah M Samy, David C Schwebel, Subramanian Senthilkumaran, Allen Seylani, Masood Ali Shaikh, Sunder Sham, B Suresh Kumar Shetty, Pavanchand H Shetty, Yonatan Solomon, Jaimie D Steinmetz, Irfan Ullah, Sana Ullah, Theo Vos, Linh Gia Vu, Naohiro Yonemoto, and Mohammad Zoladl.

Developing methods or computational machinery

Qorinah Estiningtyas Adnani, Saira Afzal, Aleksandr Y Aravkin, Akshaya Srikanth Bhagavathula, Kaleb Coberly, Xiaochen Dai, Ali Fatehizadeh, Tilaye Gebru Gebi, Farhad Ghamari, Mohammad Heidari, Vidya Kadashetti, Sorour Khateri, Adnan Kisa, Thao Thi Thu Le, Chaitanya Mittal, Madeline E Moberg, Ali H

Mokdad, Mohammad Ali Moni, Francesk Mulita, Christopher J L Murray, Mohsen Naghavi, Van Thanh Nguyen, Michal Ordak, Umar Saeed, Abdallah M Samy, Theo Vos, Mikhail Sergeevich Zastrozhin, and Peng Zheng.

Providing critical feedback on methods or results

Yohannes Habtegiorgis Abate, Ame Mehadi Abdurehman, Aidin Abedi, Eman Abu-Gharbieh, Isaac Yeboah Addo, Abiola Victor Adepoju, Qorinah Estiningtyas Adnani, Saira Afzal, Bright Opoku Ahinkorah, Sajjad Ahmad, Danial Ahmed, Haroon Ahmed, Dejene Tsegaye Alem, Adel Ali Al-Gheethi, Edward Kwabena Ameyaw, Tadele Fentabil Anagaw, Anayochukwu Edward Anyasodor, Jalal Arabloo, Seyyed Shamsadin Athari, Alok Atreya, Amirhossein Azari Jafari, Nayereh Baghcheghi, Sara Bagherieh, Hansi Bansal, Amadou Barrow, Nebiyou Simegnew Bayileyegn, Alemshet Yirga Berhie, Akshaya Srikanth Bhagavathula, Pankaj Bhardwaj, Archith Boloor, Luis Alberto Cámera, Márcia Carvalho, Eeshwar K Chandrasekar, Jung-Chen Chang, Vijay Kumar Chattu, Dinh-Toi Chu, Natália Cruz-Martins, Omid Dadras, Xiaochen Dai, Reza Darvishi Cheshmeh Soltani, Saswati Das, Subasish Das, Sisay Abebe Debela, Berecha Hundessa Demessa, Xinlei Deng, Abebaw Alemayehu Desta, Belay Desye, Meghnath Dhimal, Mahmoud Dibas, Haneil Larson Dsouza, Michael Ekholuenetale, Iman El Sayed, Daniel Berhanie Enyew, Adeniyi Francis Fagbamigbe, Ali Fatehizadeh, Syeda Anum Fatima, Florian Fischer, Richard Charles Franklin, Tushar Garg, Tilaye Gebru Gebi, Urge Gerema, Melaku Getachew, Motuma Erena Getachew, Mahaveer Golechha, Sapna Gupta, Veer Bala Gupta, Vivek Kumar Gupta, Erin B Hamilton, Mehdi Harorani, Hamidreza Hasani, Abbas M Hassan, Hossein Hassanian-Moghaddam, Mohammed Bheser Hassen, Simon I Hay, Khezar Hayat, Mohammad Heidari, Mahsa Heidari-Foroozan, Demisu Zenbaba Heyi, Ramesh Holla, Praveen Hoogar, Md Shakhaoat Hossain, Mohammad-Salar Hosseini, Soodabeh Hoveidamanesh, Olayinka Stephen Ilesanmi, Irena M Ilic, Mustapha Immurana, Chidozie C Iwu, Umesh Jayarajah, Nitin Joseph, Charity Ehimwenma Joshua, Vidya Kadashetti, Tanuj Kanchan, Himal Kandel, Rami S Kantar, Neeti Kapoor, Ibraheem M Karaye, Patrick DMC Katoto, Himanshu Khajuria, Ejaz Ahmad Khan, Sorour Khateri, Farzad Khodamoradi, Moein Khormali, Jagdish Khubchandani, Grace Kim, Adnan Kisa, Hamid Reza Koohestani, Kewal Krishan, Naveen Kumar, Lucie Laflamme, Bagher Larijani, Paolo Lauriola, Thao Thi Thu Le, Caterina Ledda, Seung Won Lee, Stephen S Lim, Stany W Lobo, Ritesh G Menezes, Alexios-Fotios A Mentis, Tomislav Mestrovic, Ted R Miller, Seyyedmohammadsadeq Mirmoeeni, Awoke Misganaw, Manish Mishra, Sanjeev Misra, Chaitanya Mittal, Madeline E Moberg, Esmaeil Mohammadi, Ali H Mokdad, Mohammad Ali Moni, Ebrahim Mostafavi, Sumaira Mubarik, Francesk Mulita, Jember Azanaw Mulualem, Temesgen Mulugeta, Christopher J L Murray, Mohsen Naghavi, Biswa Prakash Nayak, Vinod C Nayak, Seyed Aria Nejadghaderi, Huong Lan Nguyen, Van Thanh Nguyen, Ogochukwu Janet Nzoputam, Hassan Okati-Aliabad, Isaac Iyinoluwa Olufadewa, Kanyin Liane Ong, Michal Ordak, Jagadish Rao Padubidri, Ashok Pandey, Suman Pant, Utsav Parekh, Shrikant Pawar, Amy E Peden, Ionela-Roxana Petcu, Frédéric B Piel, Zahra Zahid Piracha, Ghazaleh Pourali, Ibrahim Qattea, Maryam Faiz Qureshi, Pankaja Raghav, Mosiur Rahman, Shayan Rahmani, Premkumar Ramasubramani, Sheena Ramazanu, Salman Rawaf, Nazila Rezaei, Negar Rezaei, Mohsen Rezaeian, Basema Saddik, Malihe Sadeghi, Umar Saeed, Zahra Saif, Joseph W Sakshaug, Payman Salamati, Abdallah M Samy, Rodrigo Sarmiento-SuÃ¡rez, David C Schwebel, Subramanian Senthilkumaran, Masood Ali Shaikh, Bereket Beyene Shashamo, Rahim Ali Sheikhi, Migbar Mekonnen Sibhat, Paramdeep Singh, Yonatan Solomon, Jaimie D Steinmetz, Majid Taheri, Irfan Ullah, Sana Ullah, Francesco S Violante, Theo Vos, Linh Gia Vu, Nuwan Darshana Wickramasinghe, Arzu Yigit, Naohiro Yonemoto, Zabihollah Yousefi, Muhammad Zaman, and Mohammad Zoladl.

Drafting the work or revising it critically for important intellectual content

Yohannes Habtegiorgis Abate, Mohsen Abbasi-Kangevari, Ame Mehadi Abdurehman, Aidin Abedi, Eman Abu-Gharbieh, Isaac Yeboah Addo, Abiola Victor Adepoju, Qorinah Estiningtyas Adnani, Saira Afzal, Bright Opoku Ahinkorah, Danial Ahmed, Haroon Ahmed, Mohammad Amrollahi-Sharifabadi, Tadele Fentabil Anagaw, Anayochukwu Edward Anyasodor, Jalal Arabloo, Seyyed Shamsadin Athari, Amirhossein Azari Jafari, Ashish D Badiye, Sara Bagherieh, Hansi Bansal, Amadou Barrow, Azadeh Bashiri, Akshaya Srikanth Bhagavathula, Dana Bryazka, Felix Carvalho, Márcia Carvalho, Eeshwar K Chandrasekar, Vijay Kumar Chattu, Dinh-Toi Chu, Natália Cruz-Martins, Subasish Das, Berecha Hundessa Demessa, Meghnath Dhimal, Mahmoud Dibas, Haneil Larson Dsouza, Iman El Sayed, Adeniyi Francis Fagbamigbe, Ali Fatehizadeh, Syeda Anum Fatima, Rachel Feldman, Florian Fischer, Richard Charles Franklin, Tushar Garg, Tilaye Gebru Gebi, Melaku Getachew, Motuma Erena Getachew, Farhad Ghamari, Sapna Gupta, Veer Bala Gupta, Vivek Kumar Gupta, Erin B Hamilton, Hamidreza Hasani, Abbas M Hassan, Simon I Hay, Khezar Hayat, Demisu Zenbaba Heyi, Ramesh Holla, Md Shakhaoat Hossain, Mohammad-Salar Hosseini, Sorin Hostiuc, Olayinka Stephen Ilesanmi, Irena M Ilic, Mustapha Immurana, Chidozie C Iwu, Umesh Jayarajah, Nitin Joseph, Vidya Kadashetti, Himal Kandel, Rami S Kantar, Neeti Kapoor, Patrick DMC Katoto, Himanshu Khajuria, Ejaz Ahmad Khan, Farzad Khodamoradi, Jagdish Khubchandani, Grace Kim, Adnan Kisa, Kewal Krishan, Lucie Laflamme, Iván Landires, Bagher Larijani, Thao Thi Thu Le, Caterina Ledda, Seung Won Lee, Raimundas Lunevicius, Ritesh G Menezes, Alexios-Fotios A Mentis, Tomislav Mestrovic, Ted R Miller, Seyyedmohammadsadeq Mirmoeeni, Awoke Misganaw, Manish Mishra, Chaitanya Mittal, Madeline E Moberg, Esmaeil Mohammadi, Ali H Mokdad, Mohammad Ali Moni, Ebrahim Mostafavi, Christopher J L Murray, Isabella Myers, Mohsen Naghavi, Biswa Prakash Nayak, Vinod C Nayak, Seyed Aria Nejadghaderi, Huong Lan Nguyen, Van Thanh Nguyen, Ogochukwu Janet Nzoputam, Kanyin Liane Ong, Michal Ordak, Alicia Padron-Monedero, Jagadish Rao Padubidri, Suman Pant, Shrikant Pawar, Amy E Peden, Ionela-Roxana Petcu, Zahra Zahid Piracha, Ghazaleh Pourali, Pankaja Raghav, Shayan Rahmani, Premkumar Ramasubramani, Salman Rawaf, Nazila Rezaei, Basema Saddik, Malihe Sadeghi, Farideh Sadeghian, Umar Saeed, Amirhossein Sahebkar, Zahra Saif, Saina Salahi, Abdallah M Samy, Rodrigo Sarmiento-SuÃ¡rez, David C Schwebel, Allen Seylani, Bereket Beyene Shashamo, Pavanchand H Shetty, Migbar Mekonnen Sibhat, Harpreet Singh, Paramdeep Singh, Eskinder Ayalew Sisay, Yonatan Solomon, Jaimie D Steinmetz, Majid Taheri, Irfan Ullah, Sana Ullah, Francesco S Violante, Theo Vos, Linh Gia Vu, Nuwan Darshana Wickramasinghe, Arzu Yigit, Naohiro Yonemoto, Scott M Zeng, Zhi-Jiang Zhang, Jeff T Zhao, and Mohammad Zoladl.

Managing the estimation or publications process

Saira Afzal, Ali Fatehizadeh, Tilaye Gebru Gebi, Melaku Getachew, Erin B Hamilton, Simon I Hay, Thao Thi Thu Le, Madeline E Moberg, Ali H Mokdad, Christopher J L Murray, Mohsen Naghavi, Van Thanh Nguyen, Abdallah M Samy, and Mikhail Sergeevich Zastrozhin.
